# Aziridination of a Single Carbon Atom in Alkenes via Energy Transfer Catalysis

**DOI:** 10.1002/anie.9774889

**Published:** 2026-05-07

**Authors:** Fritz Paulus, Corinna Heusel, Felix H. Wessels, Bünyamin Sikora, Maik G. Niedziella, Kilian van der Beck, Constantin G. Daniliuc, Frank Glorius

**Affiliations:** ^1^ Organisch‐Chemisches Institut Universität Münster Münster Germany

**Keywords:** alkenyl boronates, aziridination, energy transfer catalysis, NH‐aziridines, regioselective

## Abstract

Traditional alkene aziridination relies on the alkene's reaction with a (formal) nitrene species, whereby the alkene provides both carbon atoms for the formed heterocycle. In contrast to this paradigm, we herein report a divergent manifold consisting of intermolecular aziridination at a single alkene site with concomitant functionalization of the second alkene site. This process unlocks alternative chemical space from alkenes through energy transfer‐catalyzed difunctionalization of alkenyl boronates and silanes toward spring‐loaded imine intermediates and their subsequent facile conversion to an aziridine involving a 1,2‐aza‐(bora‐)Brook rearrangement. The use of visible light and the absence of metals, additional bases, and external heating allow for mild reaction conditions that furnish unprotected and highly substituted aziridines.

## Graphical abstract

Contrary to traditional alkene aziridination via (formal) nitrene transfer, we herein report a strategy that achieves intermolecular aziridination of a single alkene carbon atom and installs an additional group at the other. Enabled by metal‐free energy transfer (EnT) catalysis, alkenyl boronates and silanes are transformed into reactive intermediates which rearrange in situ through an aza‐(bora‐)Brook rearrangement.

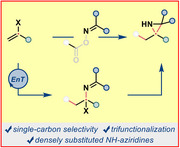

## 1 Introduction

Aziridines are characterized by a significant ring strain (27 kcal/mol) [1] governing their synthesis and reactivity. Opening these frameworks is a key strategy *en route* to functionalized amines, enabling efficient access to valuable structures—including 1,2‐amino alcohols, 1,2‐diamines, and various heterocycles [2]—and granting them an important role in many disciplines such as polymer chemistry [3] and drug design [4]. Due to their broad utility, aziridines stimulate ongoing efforts toward new synthetic methodologies [2, 5].

**FIGURE 1:**
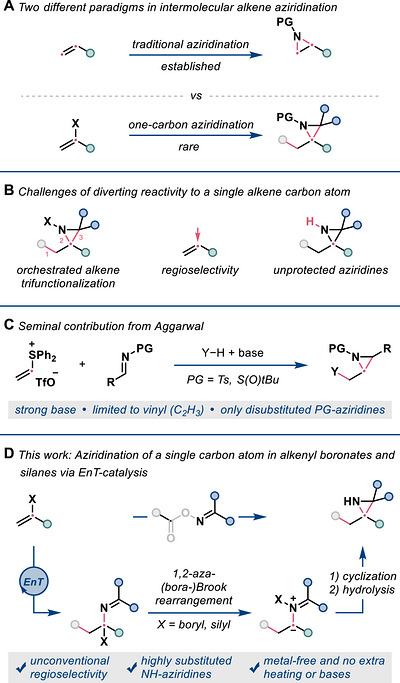
(A) Different paradigms in intermolecular alkene aziridination. (B) Challenges of diverting reactivity to a single alkene carbon atom. (C) Aggarwal's aziridination of a single carbon atom in strongly electrophilic diphenyl(vinyl)sulfonium. (D) This work: Intermolecular aziridination of a single carbon atom in alkenyl boronates and silanes via EnT‐catalysis. PG = protecting group. EnT = energy transfer.

Aziridination of abundantly available alkenes via nitrogen installation is a straight‐forward and widely applied approach [2, 5, 6]. Such reactions traditionally rely on the coupling with a (formal) nitrene species, resulting in the incorporation of both alkene carbon atoms into the aziridine ring (Figure 1A). This chemistry still represents an active area of research with many notable contributions in recent years [7, 8, 9, 10, 11, 12, 13, 14, 15, 16, 17, 18, 19, 20, 21]. Other intermolecular alkene aziridination paradigms, however, are largely unexplored, even though they offer unique opportunities to access divergent products from similar substrates [2, 22, 23, 24]. In that sense, intermolecular aziridine formation at a single alkene carbon atom—leaving the second alkene position available for additional functionalization—is a powerful approach to rapidly construct complex scaffolds in a distinctive manner (Figure 1A). The appeal of this transformation comes at the cost of high synthetic difficulty (Figure 1B). Precise orchestration is necessary to construct three bonds at the alkene in this process and, unlike classical (formal) nitrene transfer reactions, forming both aziridine bonds from a single alkene carbon atom requires bypassing the alkene's innate two‐carbon reactivity and offers no built‐in regiocontrol. Further difficulty arises when seeking to avoid assistance from nitrogen protecting groups in order to target desirable NH‐aziridines which permit facile downstream modification without prior deprotection [1, 2, 6].

A seminal solution to achieve such intermolecular aziridinations at a single alkene site was reported by Aggarwal, who demonstrated that nucleophilic attack onto a strongly electrophilic vinyl sulfonium salt generates a sulfur ylide that can undergo aziridination with an imine (Figure 1C) [22, 23]. While very elegant, this approach has been tailored for the synthesis of low‐substituted aziridines from aldimines and vinyl (C_2_H_3_) fragments, relying on strong bases and nitrogen protecting groups. Developing complementary reactions toward alternative substitution patterns from different types of alkenes, and under less harsh conditions, is consequently highly desirable to advance this field and to make intermolecular aziridinations of a single alkene carbon atom a strategically useful synthetic tool.

We aimed to establish a catalytic strategy capable of engaging less activated alkenes, installing diverse functional groups at the terminal position, and delivering structurally complex, highly substituted NH‐aziridines. Selecting an appropriate traceless directing group is critical to achieving this goal. Inspiration came from the ability of certain α‐borylated or α‐silylated amines—and nitrogen functional groups derived thereof—to undergo 1,2‐aza‐(bora‐)Brook rearrangements [25, 26, 27, 28, 29]. For imine substrates, this rearrangement can generate azomethine ylides which are common intermediates *en route* to aziridines and also play a role in certain aziridinations of imines with carbenoids [26, 30, 31]. We reasoned that the use of alkenes substituted with a boryl or silyl group could lead to such a rearrangement, followed by cyclization, upon regioselective imination [26]. Our concrete reaction design initiates with energy transfer‐catalyzed [32, 33, 34] σ‐bond cleavage of an imine‐based bifunctional reagent [35, 36, 37, 38, 39, 40, 41, 42, 43, 44, 45, 46, 47, 48, 49, 50, 51] (Figure 1D). Controlled by the reactivity of the two formed radicals, subsequent radical 1,2‐difunctionalization regioselectively functionalizes the terminal alkene position and places an imine group at the silyl's or boryl's α‐position. Rearrangement of this intermediate via a key 1,2‐aza‐Brook‐type migration then gives the desired aziridine which is set free by cleaving the shifted group (hydrolytically highly sensitive once migrated) during work‐up.

Herein, we describe the successful implementation of the outlined strategy for the intermolecular aziridination of a single alkene carbon atom. The developed reaction mildly couples borylated and silylated alkenes with a broad selection of bifunctional reagents, catalyzed by a metal‐free photosensitizer without additional heating or bases. Notably, the obtained tetrasubstituted aziridines are rare and represent a strongly underexplored substitution pattern [24]. Investigations into the reaction mechanism substantiate the proposed pathway and shed light on the facile rearrangement step.

## 2 Results and Discussion

### 2.1 Reaction Development

**FIGURE 2:**
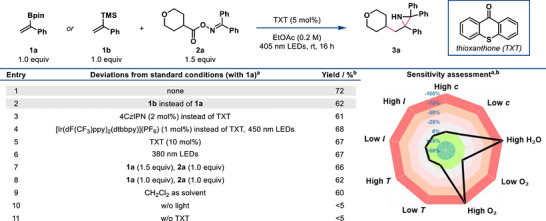
Reaction condition evaluation. ^a^Standard reaction conditions: **1a** (0.1 mmol, 1.0 equiv), **2a** (0.15 mmol, 1.5 equiv), TXT (5 mol%), EtOAc (0.2 M), 405 nm LEDs (18 W), rt, 16 h. ^b^Yields were determined by ^1^H NMR spectroscopy with an internal standard (Bpin or TMS is likely still associated with the nitrogen atom of **3a** in the crude). 4CzIPN = 1,2,3,5‐tetrakis(carbazol‐9‐yl)‐4,6‐dicyanobenzene.

We initiated our investigation with borylated alkene **1a** (1.0 equiv) and bifunctional oxime ester [36] **2a** (1.5 equiv) [52]. Using thioxanthone (TXT, 5 mol%) as metal‐free photocatalyst under irradiation with 405 nm LEDs in EtOAc (0.2 M) for 16 h delivered the desired aziridine **3a** in 72% NMR yield (Figure 2, entry 1). Silylated alkene **1b** gave **3a** in 62% under the same reaction conditions (Figure 2, entry 2). Notably, the proposed aza‐(bora‐)Brook rearrangement occurred smoothly and without additional heating. It should be mentioned that after silica gel column chromatography, free NH‐aziridines **3** were obtained. While the hydrolytically labile boryl or silyl moiety is likely still associated with the nitrogen atom in the crude, we systematically draw and describe aziridines **3** as the free NH‐aziridines for consistency throughout the manuscript. Changing the photocatalyst to 1,2,3,5‐tetrakis(carbazol‐9‐yl)‐4,6‐dicyanobenzene (4CzIPN, 2 mol%) or [Ir(dF(CF_3_)ppy)_2_(dtbbpy)](PF_6_) (1 mol%, 450 nm LEDs) led to slightly reduced NMR yields of 61% and 68%, respectively (entries 3 and 4). A higher loading of TXT (10 mol%) or using 380 nm LEDs caused an NMR yield of 67% in both cases (entries 5 and 6). Reversing the stoichiometry (66%, entry 7) or using equimolar amounts of **1a** and **2a** (62%, entry 8) led to slightly lower NMR yields as well. A 60% NMR yield was obtained when replacing EtOAc with CH_2_Cl_2_ (entry 9). Control experiments demonstrated that both light and photocatalyst are necessary for the reaction to proceed efficiently (entries 10 and 11).

Furthermore, we carried out a reaction condition‐based sensitivity assessment to identify critical reaction parameters (Figure 2) [53]. The screening showed little to no effect on the obtained yield when small deviations in concentration or light intensity were applied. Higher reaction temperature or degassing led to no changes in yield. On the other hand, a lower reaction temperature caused a small increase in yield while high levels of oxygen or water almost completely shut down the reaction. As the consequence of this assessment, our standard aziridination procedure relied on dry solvents and an argon atmosphere as important factors for optimal and reproducible results, while additional freeze–pump–thaw cycles were not implemented.

### 2.2 Substrate Scope

We then investigated the reaction's substrate scope (Table 1). First, we varied the functional group installed at the terminal alkene position by employing different reagents **2**. Product **3a**, resulting from a secondary alkyl radical, was obtained in 64% yield from borylated alkene **1a**—silylated alkene **1b** gave the same product in 54% yield. Various tertiary (**3b** and **3c**) and primary (**3d**–**g**) alkyl radicals led to the desired products in 45%–70% yield, further demonstrating the tolerance of furane (**3e**), phthalimide (**3f**), and terminal alkyne (**3g**) moieties as well as the applicability of a 2,2,2‐trifluoroethyl radical (**3d**). An unactivated alkene was also well tolerated, delivering aziridine **3h** in 51% yield. Fragments other than alkyl moieties were successfully introduced as well, including carboranyl (**3i**, 65%), [40] sulfide (**3j**, 24%), [54] and different sulfone (**3k**–**m**, 37%–43%) [39, 47, 50] groups.

**TABLE 1:** Substrate scope^a^

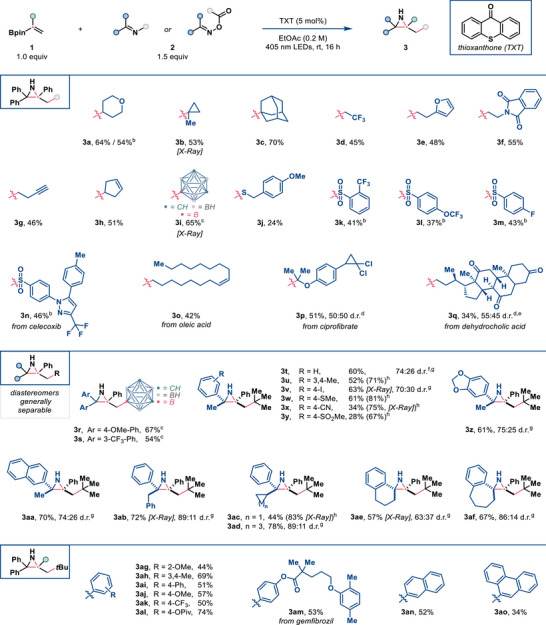

^a^ Isolated yields are given. Reactions were performed on 0.1–0.4 mmol scale.

^b^ From trimethyl(1‐phenylvinyl)silane (**1b**).

^c^ Conditions for carborane‐containing products: **1a** (2.0 equiv), **2** (1.0 equiv), 2‐isopropylthioxanthone (5 mol%), EtOAc (50 mM), 380 nm LEDs.

^d^ Isolated d.r. is given.

^e^ 1:1 stoichiometry of **1** and **2**.

^f^ [Ir(dF(CF_3_)ppy)_2_(dtbbpy)](PF_6_) (2 mol%) used instead of TXT. Irradiation with 450 nm LEDs.

^g^ Isolated yield of the depicted diastereomer and crude d.r. (determined by ^1^H NMR spectroscopy) given.

^h^ Both diastereomers were isolated separately. Given yield is the isolated yield of the depicted diastereomer. Yield in brackets is sum of the individual isolated yields of both diastereomers.

Furthermore, we prepared several bifunctional reagents **2** directly from prominent bioactive sulfonamides or carboxylic acids and used them to synthesize products derived from celecoxib (**3n**), oleic acid (**3o**), ciprofibrate (**3p**), and dehydrocholic acid (**3q**) in 34%–51% yield. Pyrazole (**3n**), unactivated alkene (**3o**), chlorinated cyclopropyl (**3p**), and ketone (**3q**) groups were well tolerated in the process. The α‐oxyalkyl radical formed during the synthesis of **3p** remained active and smoothly formed the desired aziridine.

Next, we examined different substituents at the reagent's (**2**) imine fragment. Electron‐rich (**3r**, 67%) and electron‐deficient (**3s**, 54%) aryl rings were both tolerated well. Unsymmetrical substitution with a methyl and an aryl group was feasible too, delivering compounds **3t**–**aa** in 60%–81% yield and further demonstrating the compatibility of iodide (**3v**), thioether (**3w**), cyano (**3x**), sulfone (**3y**), and acetal (**3z**) groups. Notably, for aziridines from unsymmetrical imine fragments, the resulting diastereomers could usually be easily separated by column chromatography, granting access to both isomers which would be desirable for medicinal chemical endeavors. Interestingly, the diastereomeric ratio was dependent on the electronics of the imine's aryl ring, and inversion of the diastereoselectivity was observed for aziridines **3x** and **3y**. Replacing the methyl group with a benzyl (**3ab**, 72%), cyclopropyl (**3ac**, 83%) or cyclopentyl (**3ad**, 78%) group gave the desired products as well. The cyclopropyl ring of **3ac** remained intact. Furthermore, we were particularly pleased to discover that cyclic imines of two different ring sizes gave spirocyclic aziridines **3ae** and **3af** in 57% and 67% yield, respectively.

Lastly, we investigated the scope of alkenes. As mentioned before, both boronic ester (**1a**) and trimethylsilyl (**1b**) substituted alkenes gave the desired aziridines **3**, allowing to select the appropriate lynchpin group for the given radical philicity. Furthermore, the alkene's phenyl group could be decorated in *ortho*‐ (**3ag**), *meta*‐ (**3ah**), and *para*‐(**3ah**–**am**) position—with electron‐donating as well as electron‐withdrawing substituents—delivering aziridines **3ag**–**am** in 44%–74% yield. Aziridine **3am** contained a motif derived from gemfibrozil. Naphthyl (**3an**, 52%) and phenanthrenyl (**3ao**, 34%) groups were tolerated as well.

A preliminary study on downstream reactivity of the prepared tetrasubstituted aziridine motifs **3** can be found in the Supporting Information.

### 2.3 Mechanistic Study

**FIGURE 3:**
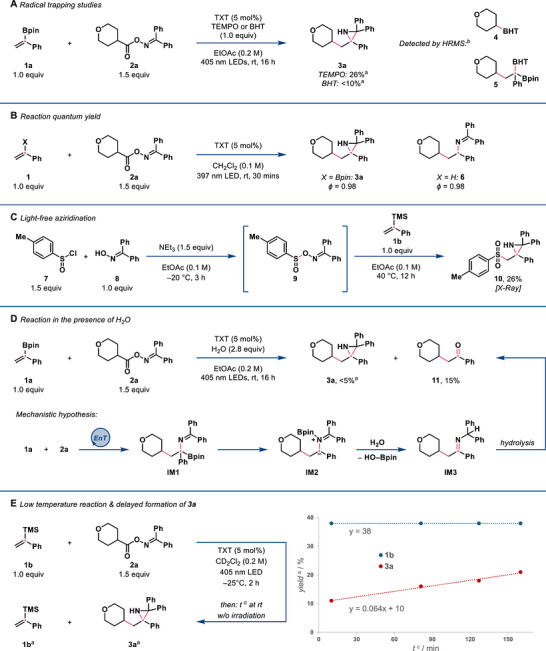
Mechanistic experiments. Isolated yields are given, unless otherwise noted. See the Supplementary Information for the experimental details. (A) Radical trapping studies. (B) Reaction quantum yield. (C) Light‐free aziridination. (D) Reaction in the presence of H_2_O. (E) Low temperature reaction & delayed formation of **3a**. [a] Yield determined by ^1^H NMR spectroscopy with mesitylene or CH_2_Br_2_ as internal standard (Bpin or TMS is likely still associated with the nitrogen atom of **3a** in the crude). [b] Given structures are reasonable proposals based on the HRMS results. [c] t refers to the time between interruption of irradiation and start of the NMR measurement. TEMPO = (2,2,6,6‐tetramethylpiperidin‐1‐yl)oxyl. BHT = butylated hydroxytoluene.

To establish a detailed mechanistic picture, we carried out a series of experiments (Figure 3). We first focused on the initial alkene difunctionalization step. Based on previous work on imine‐based bifunctional reagents, [36, 37, 38, 39, 40] the reagent's homolytic cleavage and the subsequent radical difunctionalization of alkene **1** is proposed. Adding the radical scavengers (2,2,6,6‐tetramethylpiperidin‐1‐yl)oxyl (TEMPO) or butylated hydroxytoluene (BHT) to the standard reaction mixture led to reduced NMR yields of 26% or <10%, respectively, substantiating the involvement of radicals in the formation of **3a** (Figure 3A). The adducts of both expected carbon‐centered radicals with BHT (**4** and **5**) were additionally detected, further supporting the radical nature of the initial alkene difunctionalization process.

Comparing the reaction quantum yield toward **3a** with the reaction quantum yield toward 1,2‐difunctionalized [36] product **6** gave the same value of *ϕ* = 0.98 (Figure 3B). This is in line with photochemical alkene difunctionalization constituting the first stage of product formation and the only photo‐involved reaction step. Reacting sulfinyl oxime **9** [55] with alkene **1b** in the absence of irradiation and photocatalyst led to aziridine **10** in 26% yield (Figure 3C), additionally confirming that the aziridination step does not require light and proceeds under mild heating.

The polar nature of the rearrangement was supported by a standard reaction of **1a** and **2a** with added water (Figure 3D). Formation of product **3a** was strongly suppressed and ketone **11** was isolated instead. We reason that **IM1** forms **IM2** upon 1,2‐boron shift which can then be protonated by the added H_2_O instead of cyclizing toward **3a**. Hydrolysis of imine **IM3** (presumably during column chromatography) would then give ketone **11**. This experiment also explains the high water sensitivity of the aziridination reaction which we noted when carrying out the reaction condition‐based sensitivity assessment [53]. We believe that trapping of imine **IM3** with different nucleophiles might be utilized to develop follow‐up methodologies.

Lastly, we were interested in the reaction outcome at low temperatures, hoping that we could temporally separate the photochemical 1,2‐difunctionalization and the thermal rearrangement. For this reason, the standard photoreaction with alkene **1b** was performed at a temperature of –25 °C and interrupted by turning off the irradiation after 2 h (Figure 3E). A low NMR yield of **3a** was initially observed which then linearly increased with time while the reaction mixture was kept at room temperature without irradiation. On the other hand, the amount of alkene **1b** remained constant during the examined time frame. This supports the presence of an intermediate that is photochemically generated from **1b** and thermally converted to **3a** (see the Supporting Information for further details).

## 3 Conclusion

In conclusion, we have developed a new method for the unconventional aziridination of a single alkene carbon atom. The regioselective imination of alkenyl boronates and silanes was identified as an effective synthetic means to access reactive intermediates in situ, which underwent aziridination upon facile rearrangement. Relying on energy transfer catalysis led to particularly mild and metal‐free reaction conditions. A broad array of radical precursors enabled versatile functionalizations of the second alkene position, constructing highly substituted motifs. We anticipate that this report will pave the way for further alkene aziridination reactions with unconventional regioselectivity. Additionally, intermolecular trapping of the intermediates after 1,2‐boryl or ‐‍silyl migration is expected to enable other types of functionalization reactions at a single alkene site.

## Conflicts of Interest

The authors declare no conflicts of interest.

## Supporting information




**Supporting File**: anie72358‐sup‐0001‐SuppMat.pdf.

## Data Availability

The data that supports the findings of this study are available in the Supporting Information of this article. Deposition numbers CCDC‐2485003 (for **3b**), CCDC‐2485004 (for **3i**), CCDC‐2485005 (for **3v‐major**), CCDC‐2487697 (for **3x‐major**), CCDC‐2485006 (for **3ab‐major**), CCDC‐2485007 (for **3ac‐minor**), CCDC‐2485008 (for **3ae‐major**), and CCDC‐2485009 (for **10**) contain the supplementary crystallographic data for this paper. These data are provided free of charge by the joint Cambridge Crystallographic Data Centre and Fachinformationszentrum Karlsruhe Access Structures service.
