# Supplementary material for: Aziridination of a Single Carbon Atom in Alkenes via Energy Transfer Catalysis
Source: Angew Chem Int Ed Engl. 2026 May 7;65(26):e9774889. doi: 10.1002/anie.9774889 (PMC13285470; doi:10.1002/anie.9774889)
Supplement: Supplementary file 1 — Supporting File: anie72358‐sup‐0001‐SuppMat.pdf. [file ANIE-65-e9774889-s001.pdf]

Aziridination of a Single Carbon Atom in Alkenes via Energy Transfer Catalysis

Fritz Paulus,† Corinna Heusel,† Felix H. Wessels,† Bünyamin Sikora, Maik G. Niedziella, Kilian van der Beck, Constantin G. Daniliuc, and Frank Glorius*

Organisch-Chemisches Institut, Universität Münster, Corrensstraße 36, 48149 Münster, Germany.

*glorius@uni-muenster.de;
†These authors contributed equally.

SUPPORTING INFORMATION

Contents

1. General information 3

   1.1 Experimental conditions, reagents, and solvent 3

   1.2 Analytical techniques 3

   1.3 Purification techniques 4

   1.4 Photochemical set-up 4

2. Starting materials 6

   2.1 Alkenes 1 6

   2.2 Bifunctional reagents 2 18

3. Reaction development 30

   3.1 Reaction optimization 30

   3.2 Sensitivity assessment 30

4. Substrate scope 33

   4.1 Characterization data 33

   4.2 Limitations 60

5. Mechanistic experiments 61

   5.1 Radical trapping studies 61

   5.2 Reaction quantum yield 62

   5.3 Light-free aziridination 65

   5.4 Reaction in the presence of H_2O 65

   5.5 Low temperature reaction & delayed formation of 3a 66

6. Preliminary downstream modifications 68

7. X-Ray analysis 70

8. NMR spectra 80

   8.1 Starting materials 80

   8.2 Substrate scope 108

8.3 Other 163

9. References 169

1. General information

1.1 Experimental conditions, reagents, and solvent

Reactions were carried out in oven-dried glassware under argon, using standard Schlenk techniques, unless otherwise stated. Given reaction temperatures are the ones of the heating/cooling media and the reactions were stirred using PTFE-coated magnetic stirring bars. Removal of low boiling solvents was achieved by rotary evaporation under reduced pressure, using a water bath at 40 °C.

Commercially available chemicals were purchased from commercial suppliers and were used without further purification, unless otherwise noted. Used photocatalysts (thioxanthone, 1,2,3,5-tetrakis(carbazol-9-yl)-4,6-dicyanobenzene (4CzIPN), and [Ir(dF(CF_3)ppy)$_2$(dtbbpy)](PF_6) ([Ir-F])) are commercially available.

The following solvents were purchased from ACROS Organics, Fischer Scientific, and Sigma-Aldrich (HPLC grade), dried using a solvent purification system (SPS) with activated alumina columns – custom-built by the "Feinmechanische Werkstatt des Organisch-Chemischen Instituts, Universität Münster" – and collected under positive argon pressure: dichloromethane (CH_2Cl_2), acetonitrile (MeCN), tetrahydrofuran (THF), diethylether (Et_2O), *N*,*N*-dimethylformamide (DMF), toluene, and methanol (MeOH). Other dry solvents were bought from ACROS Organics (ACROS ExtraDry solvents with ACROSeal® cap), stored over 3 or 4 Å molecular sieves, and collected under positive argon pressure.

n-Pentane, CH_2Cl_2, and ethyl acetate (EtOAc) used for column chromatography, extractions, etc., were purchased of technical grade and purified by atmospheric pressure distillation.

1.2 Analytical techniques

GC samples were filtered over a short plug of silica eluting with ethyl acetate prior to analysis, if not stated otherwise. GC-MS spectra were recorded on an Agilent Technologies 7890A GC-system (Agilent 5975C VL MSD or an Agilent 5975 MSD) with a HP-5MS column (0.25 mm · 30 m, film: 0.25 μm).

NMR spectra were recorded at room temperature in deuterated solvents using a Bruker Avance II 400, Bruker Avance Neo 400, Agilent DD2 500 or an Agilent DD2 600 spectrometer. Chemical shifts (δ) for ^{1}H and ^{13}C NMR spectra are reported in parts per million (ppm) relative to tetramethylsilane (TMS) using the residual solvent signals as references for ^{1}H and ^{13}C NMR spectra ($CDCl_3$: δ_H = 7.26 ppm, δ_C = 77.16 ppm; CD_2Cl_2: δ_H = 5.32 ppm, δ_C = 53.84 ppm). ^{11}B and ^{19}F NMR spectra are not referenced. NMR-signal multiplicities are reported using the following abbreviations (or combination thereof): s = singlet, d = doublet, t = triplet, q = quartet, p = quintet,

h = sextet; hept = heptet; m = multiplet, br = broad signal. Coupling constants (*J*) are quoted in Hz. The spectra were processed using MestReNova 15, typically applying standard phase and baseline correction.

ESI high-resolution mass spectra (HRMS) were recorded by the mass spectrometry department of the Organisch-Chemisches Institut, University of Münster, on a MicroToF spectrometer (Bruker Daltronics) or on an Orbitap LTQ XL (Thermo-Fisher Scientific).

1.3 Purification techniques

Thin layer chromatography (TLC) was performed using Merck silica gel 60 F254 aluminum plates. Visualization was achieved with UV light (254 nm) and/or staining with basic $KMnO_4$ solution (4 g $KMnO_4$, 10 g K_2CO_3, 1 g NaOH in 200 mL of distilled water), acidic $PdCl_2$ solution (1 wt.% $PdCl_2$ in 6 M aq. HCl; used for carborane-containing products) or Dragendorff's stain (2 g Bi(III) nitrate pentahydrate and 40 g of KI in 180 mL of distilled water and 20 mL of glacial acetic acid; used for aziridine-containing products). Flash column chromatography was carried out manually using standard techniques with silica gel (40-63 mesh) by Merck under 0.3-0.5 bar overpressure or by using a Biotage Isolera™ flash chromatography system equipped with Biotage® Sfär Silica D Duo 60 µm columns. The used solvent system is specified in the described procedures.

In some cases, employing deactivated silica improved the isolation. Deactivated silica was prepared by adding 250 mL of silica to a 500 mL round-bottom flask, adding *n*-pentane to make a slurry, and then adding 7 mL of triethylamine. After mixing well, the volatiles were removed under reduced pressure to obtain the basified silica.

1.4 Photochemical set-up

Unless otherwise noted, photochemical reactions were performed in a Hepatochem EvoluChem™ PhotoRedOx Box Duo device and irradiated with two EvoluChem™ HCK1012-01-008 LEDs (30 W, λ_{max} = 450 nm), two EvoluChem™ HCK1012-01-010 LEDs (18 W, λ_{max} = 405 nm), or two EvoluChem™ HCK1012-01-013 LEDs (18 W, λ_{max} = 380 nm). The reaction temperature in this setup was measured to be between 30 °C and 35 °C.

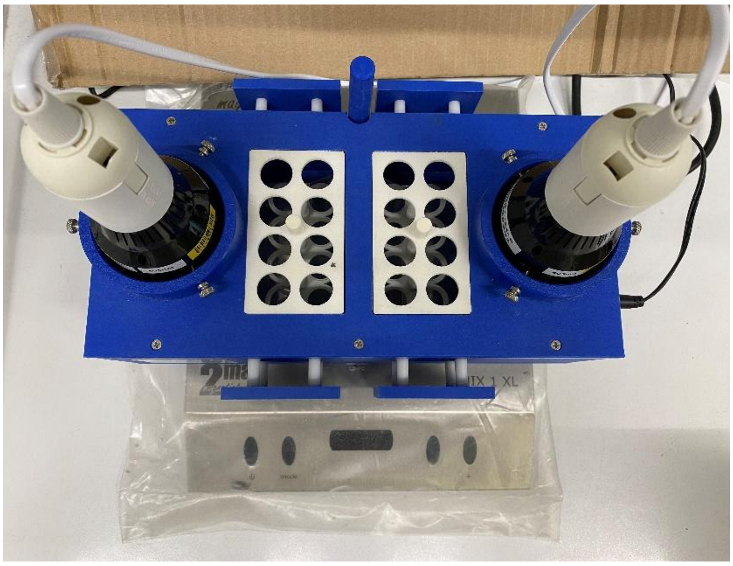

Supplementary Figure 1. Hepatochem EvoluChem™ PhotoRedOx Box Duo (with two light sources and vial holders) on a stirring plate.

2. Starting materials

2.1 Alkenes 1

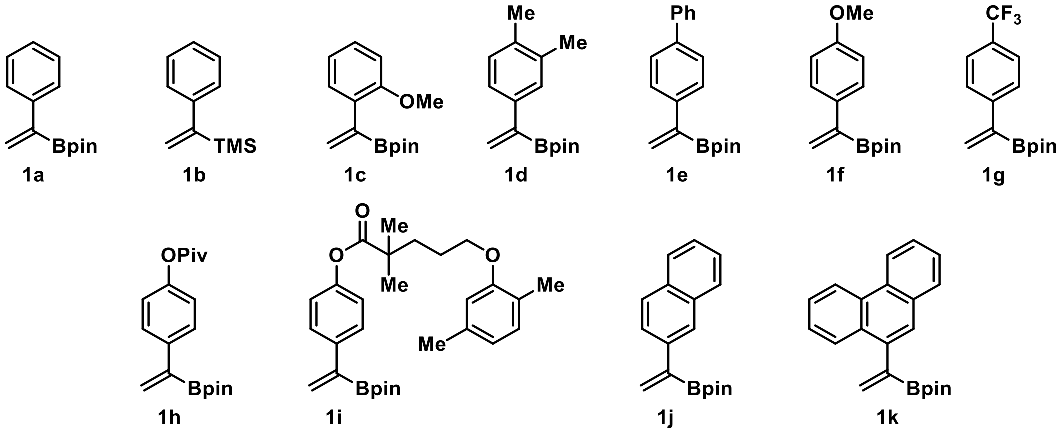

Supplementary Figure 2. Used alkenes **1**.

Alkene **1a** is commercially available. All other alkenes were prepared according to the following procedures:

Trimethyl(1-phenylvinyl)silane (1b)

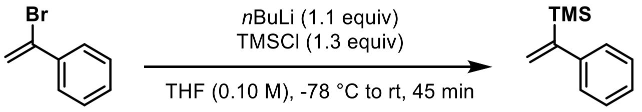

An oven-dried Schlenk flask was charged with a magnetic stir bar and (1-bromovinyl)benzene (1.83 g, 10.0 mmol, 1.0 equiv). THF (100 ml, 0.10 M) was added followed by dropwise *n*BuLi (1.6 M in hexanes; 7.1 mL, 11.4 mmol, 1.1 equiv) at -78 °C. After 15 minutes, TMSCl (1.41 g, 13.0 mmol, 1.3 equiv) was added dropwise, and the reaction mixture was stirred at room temperature for 30 minutes. The reaction was then quenched with MeOH (5.0 mL) and concentrated under reduced pressure. Purification by flash column chromatography (100% *n*-pentane) gave the title compound as a colorless oil (1.53 g, 8.68 mmol, 87 %).

^{1}H NMR (400 MHz, $CDCl_3$) δ = 7.34 – 7.27 (m, 2H), 7.25 – 7.16 (m, 3H), 5.83 (d, *J* = 3.0 Hz, 1H), 5.61 (d, *J* = 3.0 Hz, 1H), 0.18 (s, 9H).

^{13}C{^{1}H} NMR (101 MHz, $CDCl_3$) δ = 153.6, 144.9, 128.3, 127.3, 126.8, 126.4, -0.8.

The spectroscopic data is in agreement with the literature.[1]

2-(1-(2-Methoxyphenyl)vinyl)-4,4,5,5-tetramethyl-1,3,2-dioxaborolane (1c)

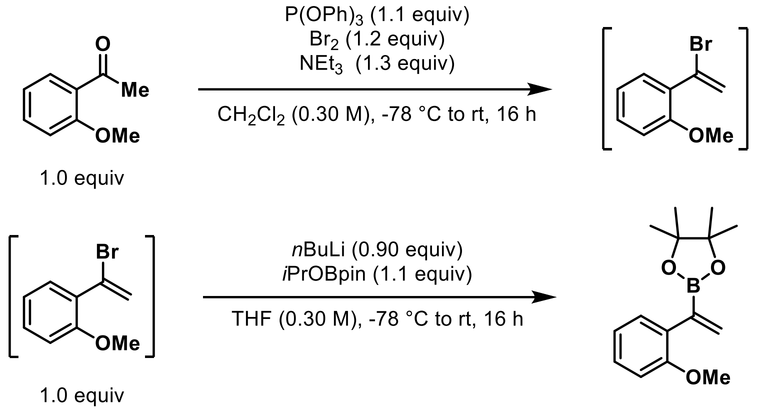

The first step was carried out according to a modified literature procedure.[2] An oven-dried Schlenk flask was charged with a magnetic stir bar, $P(OPh)_3$ (2.87 mL, 11.0 mmol, 1.1 equiv) was dissolved in CH_2Cl_2 (33 mL, 0.30 M) and cooled to -78 °C. Br_2 (0.615 mL, 12.0 mmol, 1.2 equiv) and NEt_3 (1.80 mL, 13.0 mmol, 1.3 equiv) was added dropwise. 2-methoxy acetophenone (1.38 mL, 10.0 mmol, 1.0 equiv) was added and the reaction mixture was stirred at room temperature for 16 hours. The solvent was removed under reduced pressure and the crude product was filtered over a small silica plug which was flushed with *n*-pentane to afford a dark yellow oil (after concentration) that was used without further purification.

This step was carried out according to a modified literature procedure.[3] An oven-dried Schlenk flask was charged with a magnetic stir bar and parts of the crude vinyl bromide (426 mg, 2.00 mmol, 1.0 equiv). THF (6.7 mL, 0.30 M) was added and the solution was cooled to -78 °C. *n*BuLi (1.12 mL, 1.60 M in hexanes, 1.79 mmol, 0.90 equiv) was added dropwise and the reaction mixture was stirred for 15 minutes. 2-Isopropoxy-4,4,5,5-tetramethyl-1,3,2-dioxaborolane (0.449 mL, 2.20 mmol, 1.1 equiv) was added dropwise and the reaction mixture was stirred at room temperature for 16 hours. The reaction was quenched with water and extracted with ethyl acetate (3 x 10 mL). The combined organic layers were dried over $MgSO_4$ and the solvent was removed under reduced pressure. Purification by column chromatography (*n*-pentane/diethyl ether = 100:1 to 50:1) gave the product as a colorless liquid (304 mg, 1.17 mmol, 58%).

^{1}H NMR (599 MHz, $CDCl_3$) δ 7.25 – 7.20 (m, 2H), 6.92 (td, J = 7.4, 1.1 Hz, 1H), 6.84 (dd, J = 8.7, 1.1 Hz, 1H), 5.89 (d, J = 3.3 Hz, 1H), 5.86 (d, J = 3.3 Hz, 1H), 3.80 (s, 3H), 1.30 (s, 12H).

^{13}C{^{1}H} NMR (151 MHz, $CDCl_3$) δ 156.5, 132.1, 129.2, 128.6, 128.6, 121.1, 110.3, 83.6, 55.3, 24.9.

^{11}B NMR (192 MHz, $CDCl_3$) δ 30.6.

HRMS (ESI): *m/z* calculated for $[C_{15}H_{21}BO_3Na]^+$ $[M+Na]^+$: 283.1476; found: 283.1475.

2-(1-(3,4-Dimethylphenyl)vinyl)-4,4,5,5-tetramethyl-1,3,2-dioxaborolane (1d)

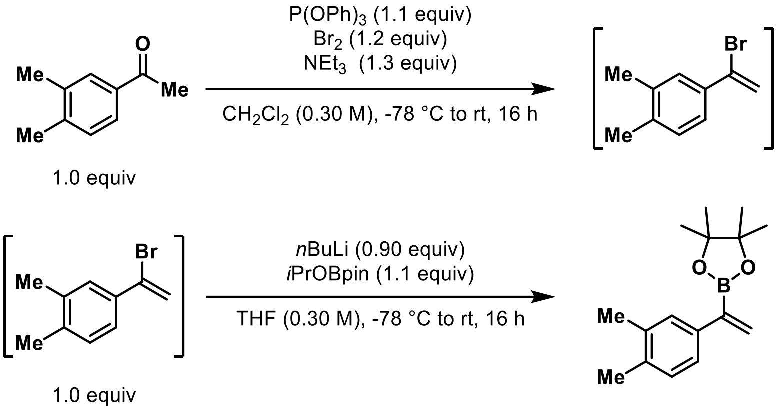

The first step was carried out according to a modified literature procedure.[2] An oven-dried Schlenk flask was charged with a magnetic stir bar, $P(OPh)_3$ (2.87 mL, 11.0 mmol, 1.1 equiv) was dissolved in CH_2Cl_2 (33 mL, 0.30 M) and cooled to -78 °C. Br_2 (0.615 mL, 12.0 mmol, 1.2 equiv) and NEt_3 (1.80 mL, 13.0 mmol, 1.3 equiv) was added dropwise. 3,4-Dimethyl acetophenone (1.48 mL, 10.0 mmol, 1.0 equiv) was added and the reaction mixture was stirred at room temperature for 16 hours. The solvent was removed under reduced pressure and the crude product was filtered over a small silica plug which was flushed with *n*-pentane to afford a yellow oil (after concentration) that was used without further purification.

This step was carried out according to a modified literature procedure.[3] An oven-dried Schlenk flask was charged with a magnetic stir bar and parts of the crude vinyl bromide (422 mg, 2.00 mmol, 1.0 equiv). THF (6.7 mL, 0.30 M) was added and the solution was cooled to -78 °C. *n*BuLi (1.12 mL, 1.6 M in hexanes, 1.79 mmol, 0.90 equiv) was added dropwise and the reaction mixture was stirred for 15 minutes. 2-Isopropoxy-4,4,5,5-tetramethyl-1,3,2-dioxaborolane (0.449 mL, 2.20 mmol, 1.1 equiv) was added dropwise and the reaction mixture was stirred at room temperature for 16 hours. The reaction was quenched with water and extracted with ethyl acetate (3x 10 mL). The combined organic layers were dried over $MgSO_4$ and the solvent was removed under reduced pressure. Purification by column chromatography (*n*-pentane/diethyl ether = 500:1 to 100:1) gave the product as a colorless liquid (242 mg, 0.937 mmol, 47%).

^{1}H NMR (599 MHz, $CDCl_3$) δ 7.24 – 7.20 (m, 2H), 7.10 – 7.06 (m, 1H), 6.03 (d, J = 3.1 Hz, 1H), 5.98 (d, J = 3.0 Hz, 1H), 2.26 (s, 3H), 2.24 (s, 3H), 1.32 (s, 12H).

^{13}C{^{1}H} NMR (151 MHz, $CDCl_3$) δ 139.2, 136.3, 135.5, 130.0, 129.6, 128.5, 124.9, 83.9, 24.9, 20.0, 19.6.

^{11}B NMR (192 MHz, $CDCl_3$) δ 30.6.

HRMS (ESI): *m/z* calculated for $[C_{16}H_{23}BO_2Na]^+$ $[M+Na]^+$: 281.1683; found: 281.1681.

2-(1-([1,1'-Biphenyl]-4-yl)vinyl)-4,4,5,5-tetramethyl-1,3,2-dioxaborolane (1e)

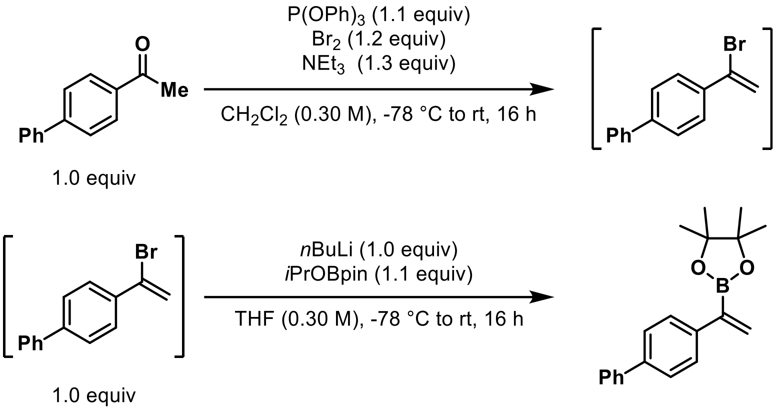

1.0 equiv

1.0 equiv

The first step was carried out according to a modified literature procedure.[2] An oven-dried Schlenk flask was charged with a magnetic stir bar, $P(OPh)_3$ (2.87 mL, 11.0 mmol, 1.1 equiv) was dissolved in CH_2Cl_2 (33 mL, 0.30 M) and cooled to -78 °C. Br_2 (0.615 mL, 12.0 mmol, 1.2 equiv) and NEt_3 (1.80 mL, 13.0 mmol, 1.3 equiv) was added dropwise. 4-Phenyl acetophenone (1.96 g, 9.99 mmol, 1.0 equiv) was added and the reaction mixture was stirred at room temperature for 16 hours. The solvent was removed under reduced pressure and the crude product was filtered over a small silica plug which was flushed with *n*-pentane to afford a white solid (after concentration) that was used without further purification.

This step was carried out according to a modified literature procedure.[3] An oven-dried Schlenk flask was charged with a magnetic stir bar and parts of the crude vinyl bromide (518 mg, 2.00 mmol, 1.0 equiv). THF (6.7 mL, 0.30 M) was added and the solution was cooled to -78 °C. *n*BuLi (1.25 mL, 1.60 M in hexanes, 2.00 mmol, 1.0 equiv) was added dropwise and the reaction mixture was stirred for 30 minutes. 2-Isopropoxy-4,4,5,5-tetramethyl-1,3,2-dioxaborolane (0.449 mL, 2.20 mmol, 1.1 equiv) was added dropwise and the reaction mixture was stirred at room temperature for 16 hours. The reaction was quenched with water and extracted with ethyl acetate (3x 10 mL). The combined organic layers were dried over $MgSO_4$ and the solvent was removed under reduced pressure. Purification by column chromatography (*n*-pentane/diethyl ether = 99:1) gave the product as a colorless liquid (128 mg, 0.418 mmol, 21%).

^{1}H NMR (400 MHz, $CDCl_3$) δ 7.64 – 7.52 (m, 6H), 7.48 – 7.39 (m, 2H), 7.37 – 7.29 (m, 1H), 6.17 – 6.06 (m, 2H), 1.35 (s, 12H).

^{13}C{^{1}H} NMR (101 MHz, $CDCl_3$) δ 141.2, 140.6, 140.0, 131.0, 128.9, 127.7, 127.2, 127.2, 127.1, 84.0, 25.0.

^{11}B NMR (128 MHz, $CDCl_3$) δ 30.5.

The spectroscopic data is in agreement with the literature.[4]

2-(1-(4-Methoxyphenyl)vinyl)-4,4,5,5-tetramethyl-1,3,2-dioxaborolane (1f)

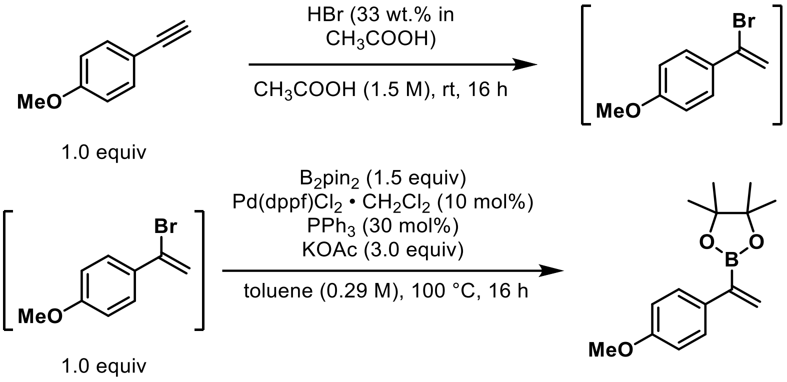

The first step was carried out according to a modified literature procedure.[5] A round-bottom flask was charged with a magnetic stir bar, 1-ethynyl-4-methoxybenzene (1.3 mL, 10 mmol, 1.0 equiv) was dissolved in acetic acid (5.0 mL). HBr (33% in AcOH, 1.5 mL) was added dropwise and the reaction mixture was stirred at room temperature for 16 hours. The reaction was quenched with sat. aq. $NaHCO_3$ and the aqueous layer was extracted with dichloromethane (2 x 20 mL). The combined organic layers were dried over $MgSO_4$, filtered and the solvent was removed under reduced pressure. The crude product was filtered over a small silica plug which was flushed with dichloromethane (5 vol%) in *n*-pentane. The crude product was used without further purification.

This step was carried out according to a modified reported procedure.[6] An oven-dried Schlenk flask was charged with a magnetic stir bar, vinyl bromide (1.0 g, 4.7 mmol, 1.0 equiv), B_2pin_2 (1.8 g, 7.1 mmol, 1.5 equiv), Pd(dppf)Cl_2 · CH_2Cl_2 (0.38 g, 0.47 mmol, 10 mol%), PPh_3 (0.37 g, 1.4 mmol, 30 mol%) and KOAc (1.4 g, 14 mmol, 3.0 equiv). The flask was evacuated and refilled with argon three times. Toluene (16 mL, 0.29 M) was added and the reaction mixture was stirred at 100 °C for 16 hours. After cooling to room temperature, the reaction mixture was filtered over a celite plug and the solvent was removed. The residue was dissolved in water and ethyl acetate, extracted in ethyl acetate (3x 10 mL) and washed with brine (1x 10 mL). The combined organic layers were dried over $MgSO_4$, filtered and the solvent was removed. Purification by column chromatography (100% *n*-pentane to *n*-pentane/diethyl ether = 19:1) gave the product as a yellow oil (0.17 mg, 0.65 mmol, 14%).

^{1}H NMR (599 MHz, $CDCl_3$) δ 7.48 – 7.42 (m, 2H), 6.91 – 6.83 (m, 2H), 6.01 (d, J = 3.0 Hz, 1H), 5.96 (d, J = 2.9 Hz, 1H), 3.80 (s, 3H), 1.32 (s, 12H).

^{13}C{^{1}H} NMR (151 MHz, $CDCl_3$) δ 159.0, 134.1, 129.1, 128.4, 113.8, 83.9, 55.4, 25.0.

^{11}B NMR (128 MHz, $CDCl_3$) δ 30.7.

HRMS (ESI): *m/z* calculated for $[C_{15}H_{21}BO_3Na]^+$ $[M+Na]^+$: 283.1476; found: 283.1475.

4,4,5,5-Tetramethyl-2-(1-(4-(trifluoromethyl)phenyl)vinyl)-1,3,2-dioxaborolane (1g)

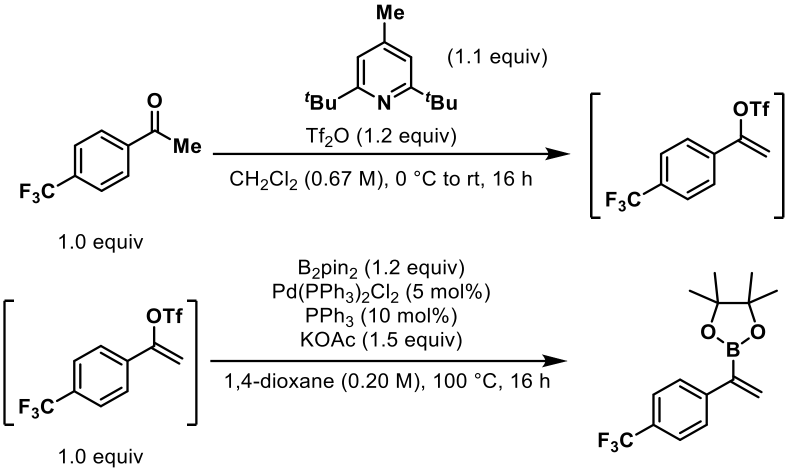

The first step was carried out according to a modified literature procedure.[7] An oven-dried Schlenk flask charged with a magnetic stir bar, the 1-(4-(trifluoromethyl)phenyl)ethan-1-one (1.88 g, 10.0 mmol, 1.0 equiv) was dissolved in CH_2Cl_2 (15.0 mL, 0.67 M) and the solution was cooled to 0 °C. 2,6-di-*tert*-butyl-4-methylpyridine (2.26 g, 11.0 mmol, 1.1 equiv) and Tf_2O (1.99 mL, 11.8 mmol, 1.2 equiv) were added and the reaction mixture was stirred at room temperature for 16 hours. The solvent was removed and pentane was added. The suspension was filtered and washed with pentane. The filtrate was washed with water (3x 20 mL) and the organic layer was dried over $MgSO_4$, filtered and the solvent was removed under reduced pressure. The crude product was filtered over a short silica plug which was flushed with ethyl acetate (2 vol%) in *n*-pentane. The crude product was used without further purification.

This step was carried out according to a modified reported procedure.[6] An oven-dried Schlenk flask was charged with a magnetic stir bar, vinyl triflate (1.00 g, 3.12 mmol, 1.0 equiv), B_2pin_2 (952 mg, 3.75 mmol, 1.2 equiv), $Pd(PPh_3)Cl_2$ (110 mg, 0.157 mmol, 5 mol%), PPh_3 (81.9 mg, 0.312 mmol, 10 mol%) and KOAc (460 mg, 4.68 mmol, 1.5 equiv). The flask was evacuated and refilled with argon three times. 1,4-dioxane (16 mL, 0.20 M) was added and the reaction mixture was stirred at 100 °C for 16 hours. After cooling to room temperature, the reaction mixture was filtered over silica and the filtrate was concentrated under reduced pressure. Purification by column chromatography (*n*-pentane/diethyl ether = 500:1) gave the product as a red oil (356 mg, 1.19 mmol, 38%).

^{1}H NMR (400 MHz, $CDCl_3$) δ 7.57 (s, 4H), 6.17 (d, J = 2.7 Hz, 1H), 6.13 (d, J = 2.8 Hz, 1H), 1.33 (s, 12H).

^{13}C{^{1}H} NMR (126 MHz, $CDCl_3$) δ 145.2, 133.1, 129.1 (q, J = 32.4 Hz), 127.6, 125.3 (q, J = 3.8 Hz), 124.5 (q, J = 271.8 Hz), 84.2, 24.9.

^{19}F NMR (376 MHz, $CDCl_3$) δ -62.5.

^{11}B NMR (128 MHz, $CDCl_3$) δ 30.4.

The spectroscopic data is in agreement with the literature.[8]

4-(1-(4,4,5,5-Tetramethyl-1,3,2-dioxaborolan-2-yl)vinyl)phenyl pivalate (1h)

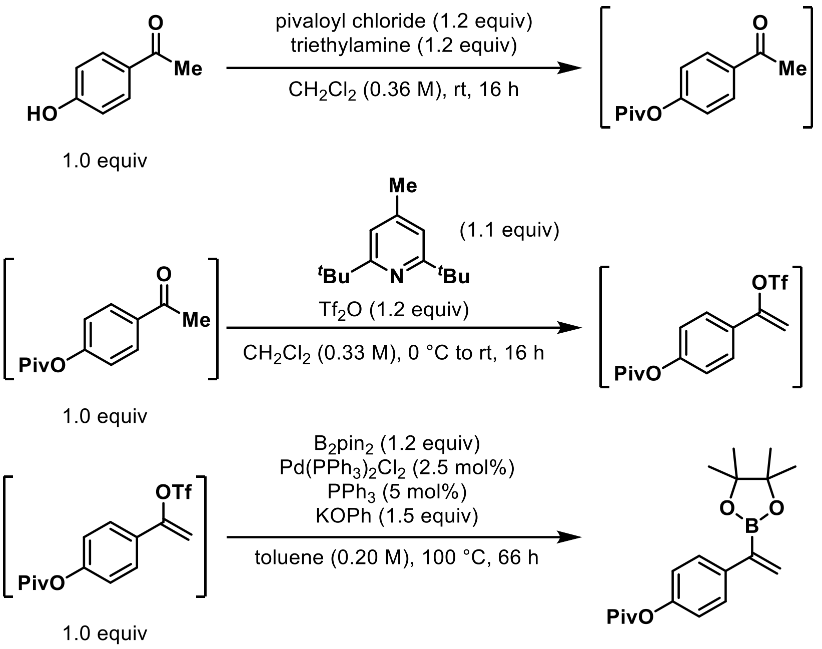

An oven-dried Schlenk flask was charged with a magnetic stir bar. 4-Hydroxyacetophenone (2.72 g, 20.0 mmol, 1.0 equiv) and pivaloyl chloride (2.89 g, 24.0 mmol, 1.2 equiv) were dissolved in CH_2Cl_2 (56 mL, 0.36 M). Triethylamine (2.42 g, 23.9 mmol, 1.2 equiv) was added and the reaction mixture was stirred at rt for 16 h. The reaction mixture was quenched with sat. aq. NH_4Cl (40 mL) and the layers were separated. The aqueous layer was extracted with CH_2Cl_2 (3x 30 mL) and the combined organic layers were dried over $MgSO_4$ and the solvent was removed under reduced pressure to afford the product as a white solid which was used without further purification.

This step was carried out according to a modified literature procedure.[7] An oven-dried Schlenk flask was charged with a magnetic stir bar and parts of the ketone (2.20 g, 10.0 mmol, 1.0 equiv). CH_2Cl_2 (30.0 mL, 0.33 M) was added and the solution was cooled to 0 °C. 2,6-Di-*tert*-butyl-4-methylpyridine (2.26 g, 11.0 mmol, 1.1 equiv) and Tf_2O (3.39 g, 12.0 mmol, 1.2 equiv) were added and the reaction mixture was stirred at room temperature for 16 hours. The solvent was removed under reduced pressure and pentane (30 mL) was added. The suspension was filtered and the solid was washed with pentane (20 mL). The filtrate was washed with 1 M aq. HCl solution (30 mL) and brine (30 mL) and the organic layer was dried over $MgSO_4$. The solvent was removed

under reduced pressure to afford the product as a dark red oil which was used without further purification.

This step was carried out according to a modified reported procedure.[6] An oven-dried Schlenk flask was charged with a magnetic stir bar, vinyl triflate (3.00 g, 8.51 mmol, 1.0 equiv), B_2pin_2 (2.59 g, 10.2 mmol, 1.2 equiv), $Pd(PPh_3)Cl_2$ (150 mg, 0.214 mmol, 2.5 mol%), PPh_3 (112 mg, 0.427 mmol, 5.0 mol%) and KOPh (1.69 g, 12.8 mmol, 1.5 equiv). The flask was evacuated and refilled with argon three times. Toluene (43 mL, 0.20 M) was added and the reaction mixture was stirred at 100 °C for 66 hours. After cooling to room temperature, the reaction mixture was filtered over silica and the filtrate was concentrated under reduced pressure. Purification by column chromatography (*n*-pentane/ethyl acetate = 20:1) gave the product as a white solid (1.24 g, 3.75 mmol, 44%).

^{1}H NMR (599 MHz, $CDCl_3$) δ 7.50 – 7.47 (m, 2H), 7.01 – 6.98 (m, 2H), 6.07 – 6.03 (m, 2H), 1.35 (s, 9H), 1.32 (s, 12H).

^{13}C{^{1}H} NMR (151 MHz, $CDCl_3$) δ 177.2, 150.4, 139.0, 131.1, 128.3, 121.2, 110.2, 84.0, 39.2, 27.3, 25.0.

^{11}B NMR (192 MHz, $CDCl_3$) δ 30.5.

HRMS (ESI): *m/z* calculated for $[C_{19}H_{27}BO_4Na]^+$ $[M+Na]^+$: 353.1895; found: 353.1896.

4-(1-(4,4,5,5-Tetramethyl-1,3,2-dioxaborolan-2-yl)vinyl)phenyl 5-(2,5-dimethylphenoxy)-2,2-dimethylpentanoate (1i)

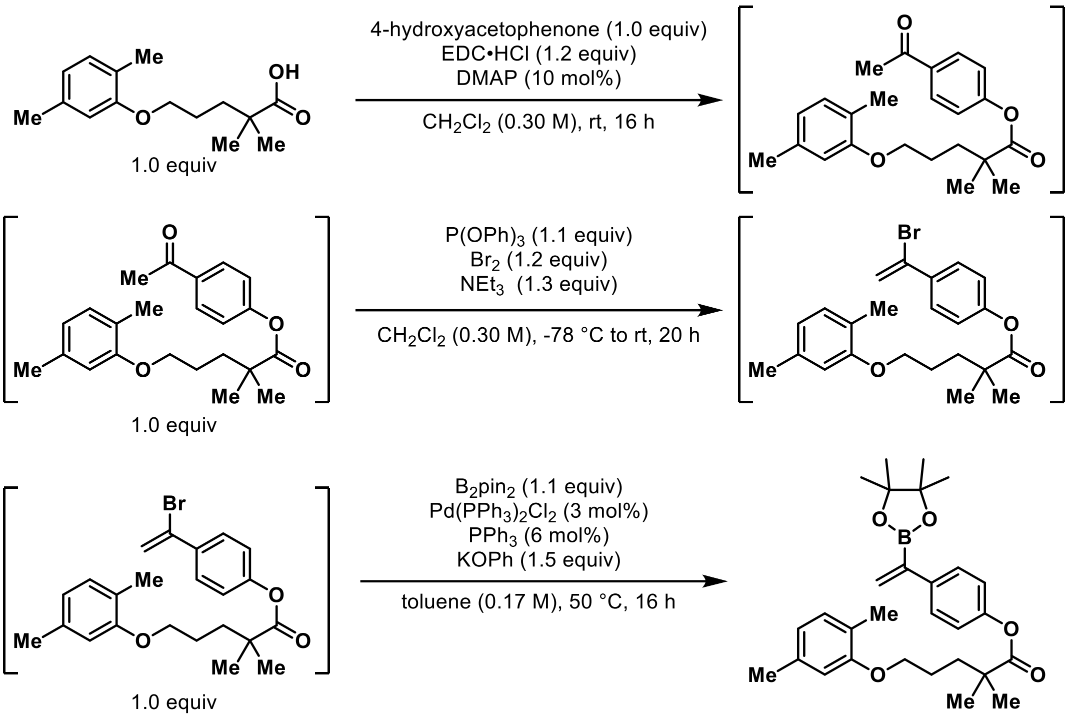

An oven-dried Schlenk flask was charged with a magnetic stir bar, gemfibrozil (2.50 g, 9.99 mmol, 1.0 equiv), EDC·HCl (2.30 g, 12.0 mmol, 1.2 equiv) and DMAP (122 mg, 0.999 mmol, 10 mol%). CH_2Cl_2 (33 mL, 0.30 M) was added and the solution was stirred for 5 minutes. 4-Hydroxyacetophenone (1.36 g, 9.99 mmol, 1.0 equiv) was added and the reaction mixture was stirred at room temperature for 16 hours. The reaction was quenched with water (15 mL) and the phases were separated. The aqueous layer was extracted with CH_2Cl_2 (3x 20 mL) and the combined organic layers were washed with brine (20 mL) and dried over $MgSO_4$. The solvent was removed under reduced pressure to afford the product as a colorless oil which was used without further purification.

This step was carried out according to a modified literature procedure.[2] An oven-dried Schlenk flask was charged with a magnetic stir bar and set under an argon atmosphere. $P(OPh)_3$ (2.87 mL, 11.0 mmol, 1.1 equiv) was dissolved in CH_2Cl_2 (33 mL, 0.30 M) and cooled to -78 °C. Br_2 (0.615 mL, 12.0 mmol, 1.2 equiv) and NEt_3 (1.80 mL, 13.0 mmol, 1.3 equiv) were added dropwise to afford a yellow solution. The ketone (3.68 g, 9.99 mmol, 1.0 equiv) was dissolved in CH_2Cl_2 (2 mL) and added to the yellow solution. The reaction mixture was stirred at room temperature for 20 hours. The solvent was removed under reduced pressure and the crude product was filtered over a small silica plug and was flushed with a solution of ethyl acetate in *n*-pentane (2%) to afford the product as a yellow oil that was used without further purification.

This step was carried out according to a modified reported procedure.[6] An oven-dried Schlenk flask was charged with a magnetic stir bar, parts of the bromide (863 mg, 2.00 mmol, 1.0 equiv), B_2pin_2 (559 mg, 2.20 mmol, 1.1 equiv), $Pd(PPh_3)Cl_2$ (42.1 mg, 0.0600 mmol, 3 mol%), PPh_3 (31.5 mg, 0.120 mmol, 6 mol%) and KOPh (397 mg, 3.00 mmol, 1.5 equiv). The Schlenk flask was evacuated and backfilled with argon three times and toluene (12 mL, 0.17 M) was added. The reaction mixture was stirred at 50 °C for 16 hours. After cooling to room temperature, the reaction mixture was quenched with water (10 mL) and the phases were separated. The aqueous layer was extracted with ethyl acetate (3x 20 mL) and the combined organic layers were dried over $MgSO_4$. The solvent was removed under reduced pressure. Purification by column chromatography (*n*-pentane/ethyl acetate = 20:1) gave the product as a colourless oil (396 mg, 0.828 mmol, 41%).

^{1}H NMR (599 MHz, $CDCl_3$) δ 7.50 – 7.45 (m, 2H), 7.01 (d, J = 7.5 Hz, 1H), 6.99 – 6.96 (m, 2H), 6.68 – 6.65 (m, 1H), 6.64 – 6.62 (m, 1H), 6.07 – 6.04 (m, 2H), 4.02 – 3.95 (m, 2H), 2.33 – 2.29 (m, 3H), 2.18 (s, 3H), 1.90 – 1.84 (m, 4H), 1.37 (s, 6H), 1.32 (s, 12H).

^{13}C{^{1}H} NMR (151 MHz, $CDCl_3$) δ 176.5, 157.1, 150.3, 139.1, 136.6, 131.1, 130.5, 128.3, 123.8, 121.2, 120.9, 112.1, 84.0, 68.0, 42.6, 37.3, 25.4, 25.3, 25.0, 21.6, 15.9.

^{11}B NMR (192 MHz, $CDCl_3$) δ 30.5.

HRMS (ESI): *m/z* calculated for $[C_{29}H_{39}BO_5Na]^+$ $[M+Na]^+$: 501.2783; found: 501.2787.

4,4,5,5-Tetramethyl-2-(1-(naphthalen-2-yl)vinyl)-1,3,2-dioxaborolane (1j)

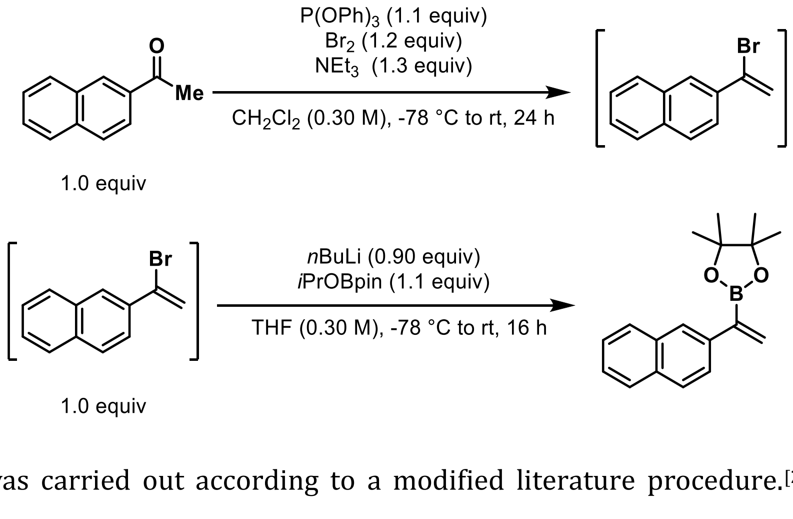

The first step was carried out according to a modified literature procedure.[2] An oven-dried Schlenk flask was charged with a magnetic stir bar, $P(OPh)_3$ (2.87 mL, 11.0 mmol, 1.1 equiv) was dissolved in CH_2Cl_2 (33 mL, 0.30 M) and cooled to -78 °C. Br_2 (0.615 mL, 12.0 mmol, 1.2 equiv) and NEt_3 (1.80 mL, 13.0 mmol, 1.3 equiv) was added dropwise. 2-Acetonaphthone (1.70 g, 10.0 mmol, 1.0 equiv) was added and the reaction mixture was stirred at room temperature for 24 hours. The solvent was removed under reduced pressure and the crude product was filtered over a small

silica plug and was flushed with *n*-pentane to afford a red solid that was used without further purification.

This step was carried out according to a modified literature procedure.[3] An oven-dried Schlenk flask was charged with a magnetic stir bar and parts of the crude vinyl bromide (466 mg, 2.00 mmol, 1.0 equiv). THF (6.7 mL, 0.30 M) was added and the solution was cooled to -78 °C. *n*BuLi (1.12 mL, 1.60 M in hexanes, 1.79 mmol, 0.90 equiv) was added dropwise and the reaction mixture was stirred for 15 minutes. 2-Isopropoxy-4,4,5,5-tetramethyl-1,3,2-dioxaborolane (0.449 mL, 2.20 mmol, 1.1 equiv) was added dropwise and the reaction mixture was stirred at room temperature for 16 hours. The reaction was quenched with water and extracted with ethyl acetate (3 x 10 mL). The combined organic layers were dried over $MgSO_4$ and the solvent was removed under reduced pressure. Purification by column chromatography (*n*-pentane/diethyl ether = 250:1 to 100:1) gave the product as a white solid (156 mg, 0.557 mmol, 28%).

^{1}H NMR (400 MHz, $CDCl_3$) δ 7.97 – 7.92 (m, 1H), 7.86 – 7.76 (m, 3H), 7.63 (dd, *J* = 8.5, 1.8 Hz, 1H), 7.49 – 7.39 (m, 2H), 6.21 (d, *J* = 2.9 Hz, 1H), 6.15 (d, *J* = 2.9 Hz, 1H), 1.36 (s, 12H).

^{13}C{^{1}H} NMR (151 MHz, $CDCl_3$) δ 139.0, 133.7, 132.8, 131.3, 128.4, 127.8, 127.6, 126.3, 126.0, 125.7, 125.7, 84.0, 25.0.

^{11}B NMR (192 MHz, $CDCl_3$) δ 30.6.

HRMS (ESI): *m/z* calculated for $[C_{18}H_{21}BO_2Na]^+$ $[M+Na]^+$: 303.1527; found: 303.1524.

4,4,5,5-Tetramethyl-2-(1-(phenanthren-9-yl)vinyl)-1,3,2-dioxaborolane (1k)

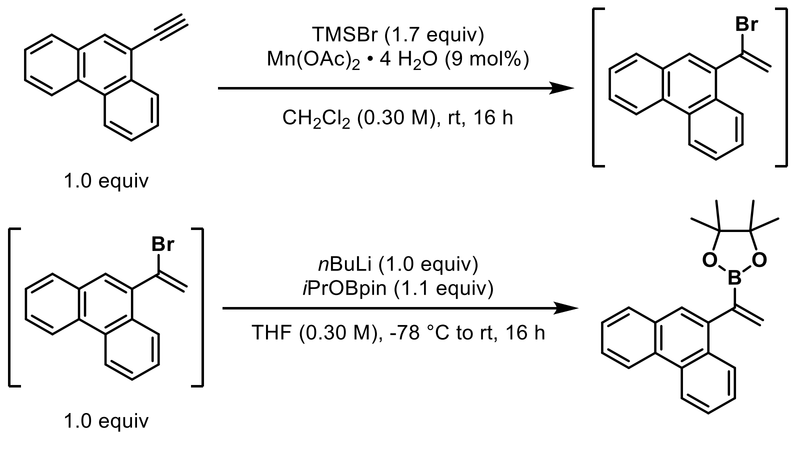

The first step was carried out according to a modified literature procedure.[9] An oven-dried Schlenk flask was charged with a magnetic stir bar, 9-ethynylphenanthrene (1.01 g, 4.99 mmol, 1.0 equiv) and $Mn(OAc)_2 \cdot 4\,H_2O$ (107 mg, 0.437 mmol, 9 mol%). The flask was evacuated and backfilled with argon three times. CH_2Cl_2 (16.7 mL, 0.30 M) was added and bromotrimethylsilane

(1.10 mL, 8.33 mmol, 1.7 equiv) was added dropwise. The reaction mixture was stirred at room temperature for 16 hours. The reaction mixture was filtered over silica and flushed with CH_2Cl_2. The solvent was removed under reduced pressure and the crude product was used without further purification.

This step was carried out according to a modified literature procedure.[3] An oven-dried Schlenk flask was charged with a magnetic stir bar and crude vinyl bromide (1.42 g, 5.01 mmol, 1.0 equiv). THF (16.7 mL, 0.30 M) was added and the solution was cooled to -78 °C. *n*BuLi (3.12 mL, 1.60 M in hexanes, 4.99 mmol, 1.0 equiv) was added dropwise and the reaction mixture was stirred for 15 minutes. 2-Isopropoxy-4,4,5,5-tetramethyl-1,3,2-dioxaborolane (1.12 mL, 5.49 mmol, 1.1 equiv) was added dropwise and the reaction mixture was stirred at room temperature for 16 hours. The reaction was quenched with water and extracted with ethyl acetate (3 x 20 mL). The combined organic layers were dried over $MgSO_4$ and the solvent was removed under reduced pressure. Purification by column chromatography (*n*-pentane/diethyl ether = 500:1 to 25:1) gave the product as a yellow solid (250 mg, 0.757 mmol, 15%).

^{1}H NMR (599 MHz, $CDCl_3$) δ 8.75 – 8.71 (m, 1H), 8.69 – 8.66 (m, 1H), 7.94 – 7.91 (m, 1H), 7.89 – 7.87 (m, 1H), 7.66 (ddd, *J* = 8.3, 6.9, 1.4 Hz, 1H), 7.62 (ddd, *J* = 8.3, 7.0, 1.6 Hz, 1H), 7.60 – 7.56 (m, 3H), 6.37 (d, *J* = 3.6 Hz, 1H), 6.11 (d, *J* = 3.7 Hz, 1H), 1.30 (s, 12H).

^{13}C{^{1}H} NMR (151 MHz, $CDCl_3$) δ 140.0, 133.6, 132.2, 131.3, 130.5, 130.2, 128.7, 126.8, 126.6, 126.4, 126.2, 126.1, 125.5, 123.0, 122.6, 84.0, 24.8.

^{11}B NMR (128 MHz, $CDCl_3$) δ 30.7.

HRMS (ESI): *m/z* calculated for $[C_{22}H_{23}BO_2Na]^+$ $[M+Na]^+$: 353.1683; found: 353.1682.

2.2 Bifunctional reagents 2

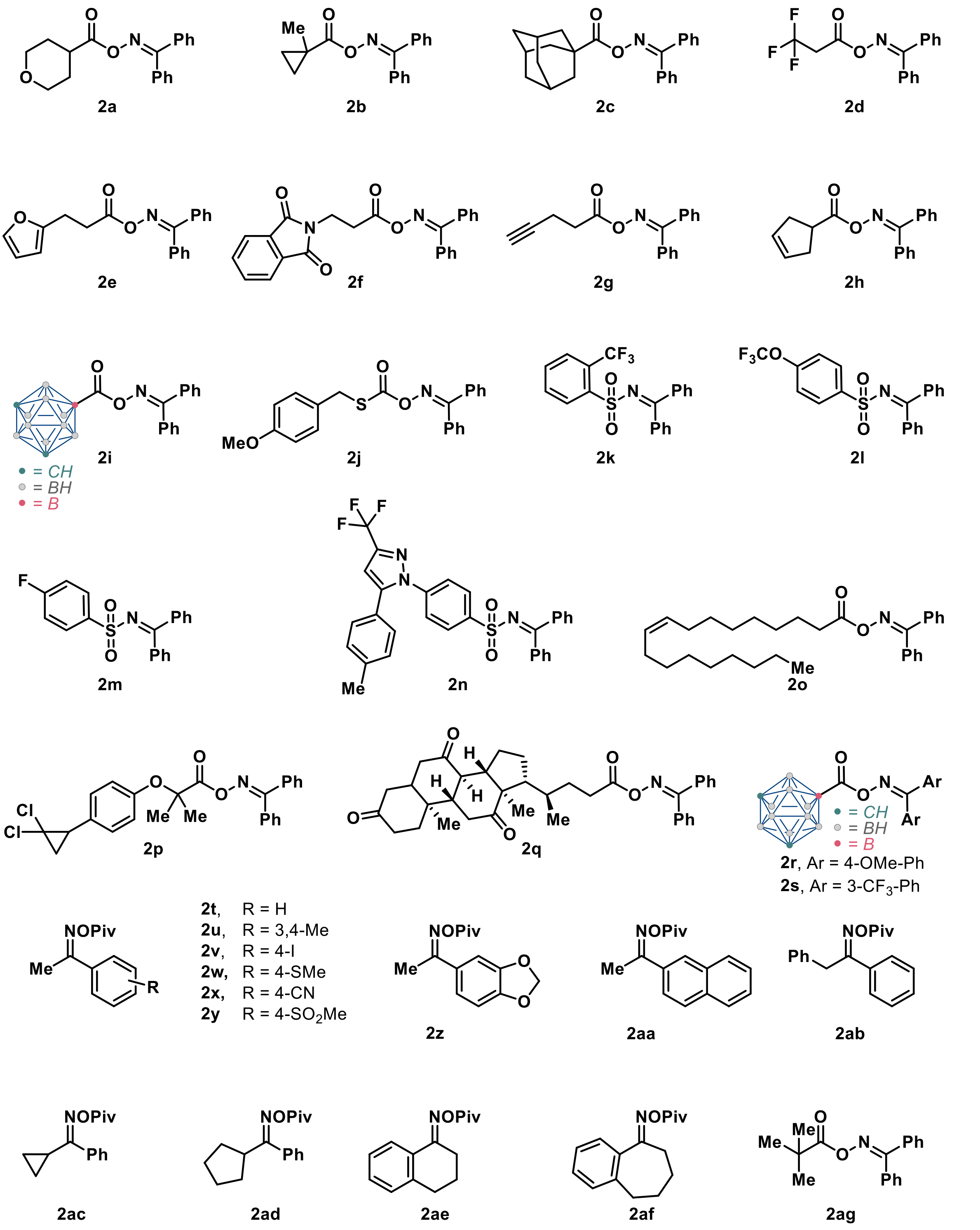

Supplementary Figure 3. Used bifunctional reagents **2**.

Reagents **2a–2c**,[10] **2g**,[10] **2i**,[11] **2j**,[12] **2k–2n**,[13] **2o–2q**,[10] **2r–2s**,[11] **2t**,[10] and **2ag**[10] as well as diphenylmethanone oxime **7**[10] were used as previously prepared by us. All other reagents were prepared according to the following procedures:

General procedure GP1 for the synthesis of bifunctional reagents 2 from different carboxylic acids

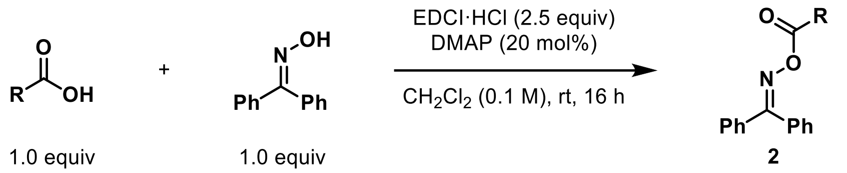

The procedure was carried out similar to a reported procedure.[10] A Teflon-coated stirring bar and diphenylmethanone oxime **7** (1.0 equiv) were placed in an oven-dried Schlenk tube along with EDCI·HCl (2.5 equiv) and the carboxylic acid (1.0 equiv), if solid. The tube was then evacuated and refilled with argon three times. Dry CH_2Cl_2 (0.10 M) was introduced next, followed by the addition of the carboxylic acid, if liquid. Then, DMAP (20 mol%) was added to the mixture.

The reaction was allowed to stir at room temperature for 16 h. Then, water (50 mL) was added, the layers were separated, and the aqueous layer was extracted with CH_2Cl_2 (2 x 30 mL). The organic layers were combined, dried over Na_2SO_4, filtered, and evaporated. Purification by column chromatography using the specified solvent system gave the desired reagent **2**.

General procedure GP2 for the synthesis of bifunctional reagents 2 from different ketones

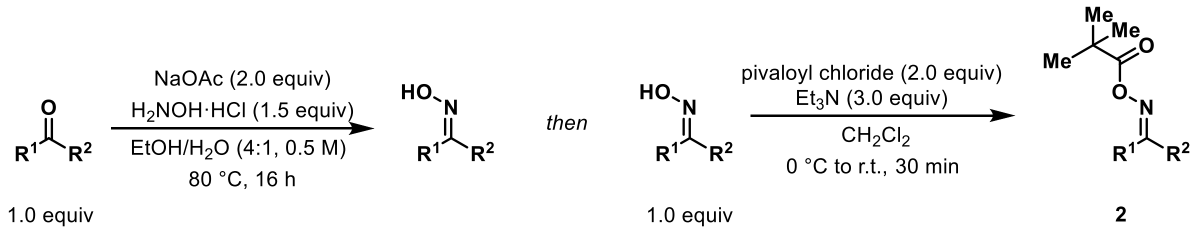

The procedure was carried out similar to a reported procedure.[10] An oven-dried Schlenk tube was charged (under air) with a stirring bar, sodium acetate (2.0 equiv), hydroxylamine hydrochloride (1.5 equiv), and the ketone (1.0 equiv), if solid. A mixture of EtOH/H_2O (4:1, 0.5 M) was then added, followed by ketone **1**, if liquid. The reaction mixture was stirred at 80 °C for 16 h. After completion, the ethanol was evaporated under reduced pressure, and H_2O (10 mL) was added. The aqueous phase was extracted with ethyl acetate (3 × 20 mL), and the combined organic layers were washed with H_2O (2 × 10 mL) and brine (1 × 10 mL). The organic phase was dried over $MgSO_4$, filtered, and the solvent was evaporated to yield the oxime which was taken to the next step without further purification.

The oxime (1.0 equiv) was then transferred to an oven-dried Schlenk tube equipped with a stirring bar. The tube was evacuated and backfilled with argon three times. Under argon counterflow, dry CH_2Cl_2 and triethylamine (3.0 equiv) were added while stirring. The reaction mixture was cooled to 0 °C, pivaloyl chloride (2.0 equiv) was injected, and the mixture was stirred

at room temperature for 30 min. The reaction was quenched by adding H_2O (10 mL), and the aqueous layers were extracted with CH_2Cl_2 (3 × 10 mL). The combined organic layers were washed with sat. aq. $NaHCO_3$ (1 × 10 mL) and brine (1 × 10 mL) and were then dried over $MgSO_4$. After filtration and solvent removal, the oxime ester **2** was purified by column chromatography using the specified solvent system.

Diphenylmethanone *O*-(3,3,3-trifluoropropanoyl) oxime (2d)

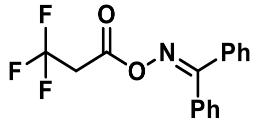

Following general procedure **GP1**, the title compound was prepared from diphenylmethanone oxime **7** (986 mg, 5.00 mmol, 1.0 equiv), 3,3,3-trifluoropropanoic acid (640 mg, 5.00 mmol, 1.0 equiv), EDCI·HCl (2.40 g, 12.5 mmol, 2.5 equiv), and DMAP (122 mg, 0.999 mmol, 20 mol%) in CH_2Cl_2 (50 mL, 0.10 M). The title compond was obtained as a white solid (713 mg, 2.32 mmol, 46%) after column chromatography (*n*-pentane/ethyl acetate = 20:1).

^{1}H NMR (400 MHz, $CDCl_3$) δ 7.62 – 7.56 (m, 2H), 7.53 – 7.44 (m, 4H), 7.41 – 7.35 (m, 2H), 7.34 – 7.28 (m, 2H), 3.21 (q, *J* = 10.0 Hz, 2H).

^{13}C{^{1}H} NMR (101 MHz, $CDCl_3$) δ 166.6, 161.7 (q, *J* = 4.4 Hz), 134.3, 132.2, 131.5, 130.0, 129.3, 128.8, 128.6, 128.4, 123.3 (q, *J* = 276.7 Hz), 38.9 (q, *J* = 31.5 Hz).

^{19}F{^{1}H} NMR (376 MHz, $CDCl_3$) δ -63.0.

HRMS (ESI): *m/z* calculated for $[C_{16}H_{12}F_3NO_2Na]^+$ $[M+Na]^+$: 330.0712; found: 330.0711.

Diphenylmethanone *O*-(3-(furan-2-yl)propanoyl) oxime (2e)

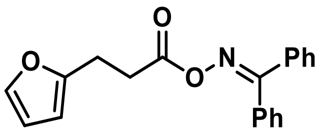

Following general procedure **GP1**, the title compound was prepared from diphenylmethanone oxime **7** (395 mg, 2.00 mmol, 1.0 equiv), 3-(furan-2-yl)propanoic acid (280 mg, 2.00 mmol, 1.0 equiv), EDCI·HCl (959 mg, 5.00 mmol, 2.5 equiv) and DMAP (48.9 mg, 0.400 mmol, 20 mol%) in CH_2Cl_2 (20 mL, 0.10 M). The title compound was obtained as a white solid (171 mg, 0.535 mmol, 27%) after column chromatography (*n*-pentane/ethyl acetate = 20:1 to 12:1).

^{1}H NMR (400 MHz, $CDCl_3$) δ 7.62 – 7.55 (m, 2H), 7.51 – 7.41 (m, 4H), 7.40 – 7.33 (m, 2H), 7.32 –

7.25 (m, 3H), 6.26 (dd, *J* = 3.2, 1.9 Hz, 1H), 6.01 – 5.95 (m, 1H), 2.98 – 2.90 (m, 2H), 2.73 – 2.65 (m, 2H).

^{13}C{^{1}H} NMR (101 MHz, $CDCl_3$) δ 170.2, 165.2, 153.9, 141.4, 134.8, 132.7, 131.1, 129.8, 129.2, 128.9, 128.5, 128.3, 110.4, 105.7, 31.7, 23.3.

HRMS (ESI): *m/z* calculated for $[C_{20}H_{17}NO_3Na]^+$ $[M+Na]^+$: 342.1101; found: 342.1096.

2-(3-(((Diphenylmethylene)amino)oxy)-3-oxopropyl)isoindoline-1,3-dione (2f)

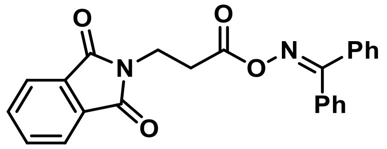

Following general procedure **GP1**, the title compound was prepared from diphenylmethanone oxime **7** (395 mg, 2.00 mmol, 1.0 equiv), 3-(1,3-dioxoisoindolin-2-yl)propanoic acid (438 mg, 2.00 mmol, 1.0 equiv), EDCI·HCl (959 mg, 5.00 mmol, 2.5 equiv) and DMAP (48.9 mg, 0.400 mmol, 20 mol%) in CH_2Cl_2 (20 mL, 0.10 M). The title compound was obtained as a white solid (647 mg, 1.62 mmol, 81%) after column chromatography (*n*-pentane/ethyl acetate = 5:1 to 2:1).

^{1}H NMR (400 MHz, $CDCl_3$) δ 7.87 – 7.78 (m, 2H), 7.75 – 7.66 (m, 2H), 7.60 – 7.53 (m, 2H), 7.48 – 7.39 (m, 4H), 7.38 – 7.28 (m, 4H), 3.97 (t, *J* = 7.4 Hz, 2H), 2.80 (t, *J* = 7.4 Hz, 2H).

^{13}C{^{1}H} NMR (101 MHz, $CDCl_3$) δ 168.6, 168.0, 165.2, 134.7, 134.2, 132.5, 132.2, 131.1, 129.8, 129.2, 128.9, 128.5, 128.3, 123.5, 33.6, 31.8.

HRMS (ESI): *m/z* calculated for $[C_{24}H_{18}N_2O_4Na]^+$ $[M+Na]^+$: 421.1159; found: 421.1134.

Diphenylmethanone *O*-cyclopent-3-ene-1-carbonyl oxime (2h)

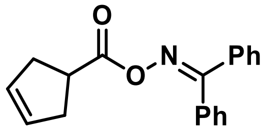

Following general procedure **GP1**, the title compound was prepared from diphenylmethanone oxime **7** (986 mg, 5.00 mmol, 1.0 equiv), cyclopent-3-ene-1-carboxylic acid (561 mg, 5.00 mmol, 1.0 equiv), EDCI·HCl (2.40 g, 12.5 mmol, 2.5 equiv), and DMAP (122 mg, 0.999 mmol, 20 mol%) in CH_2Cl_2 (50 mL, 0.10 M). Instead of column chromatography, the crude product was sonicated in *n*-pentane (5 mL) for 10 minutes and was obtained, after removal of the *n*-pentane with a pipette and drying, as a brown solid (1.02 g, 3.50 mmol, 70%).

^{1}H NMR (400 MHz, $CDCl_3$) δ 7.64 – 7.57 (m, 2H), 7.50 – 7.42 (m, 4H), 7.40 – 7.28 (m, 4H), 5.63 – 5.58 (m, 2H), 3.18 – 3.05 (m, 1H), 2.67 – 2.49 (m, 4H).

^{13}C{^{1}H} NMR (101 MHz, $CDCl_3$) δ 173.5, 165.4, 134.9, 132.8, 131.0, 129.7, 129.2, 129.0, 128.9, 128.5, 128.3, 40.6, 36.2.

HRMS (ESI): *m/z* calculated for $[C_{19}H_{17}NO_2Na]^+$ $[M+Na]^+$: 314.1152; found: 314.1129.

1-(3,4-Dimethylphenyl)ethan-1-one *O*-pivaloyl oxime (2u)

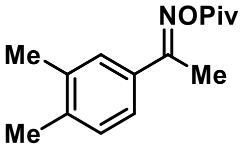

Following general procedure **GP2**, the title compound was prepared from 1-(3,4-dimethylphenyl)ethan-1-one (741 mg, 5.00 mmol, 1.0 equiv), NaOAc (820 mg, 10.0 mmol, 2.0 equiv), and hydroxylamine hydrochloride (521 mg, 7.50 mmol, 1.5 equiv) in ethanol/H_2O (4:1; 10 mL, 0.50 M).

The obtained oxime (815 mg, 4.99 mmol, 1.0 equiv) was reacted with triethylamine (1.52 g, 15.0 mmol, 3.0 equiv) and pivaloyl chloride (1.20 g, 9.95 mmol, 2.0 equiv) in CH_2Cl_2 (20 mL, 0.25 M). The title compound was obtained as an off-white solid (1.17 g, 4.73 mmol, 95%) after column chromatography (*n*-pentane/diethyl ether = 100:1 to 6:1).

^{1}H NMR (400 MHz, $CDCl_3$) δ 7.60 – 7.55 (m, 1H), 7.45 (dd, *J* = 7.8, 2.0 Hz, 1H), 7.15 (d, *J* = 7.9 Hz, 1H), 2.35 (s, 3H), 2.28 (s, 6H), 1.34 (s, 9H).

^{13}C{^{1}H} NMR (101 MHz, $CDCl_3$) δ 175.3, 163.4, 139.6, 137.0, 132.5, 129.9, 128.1, 124.7, 39.0, 27.5, 19.9, 19.8, 14.4.

HRMS (ESI): *m/z* calculated for $[C_{15}H_{21}NNaO_2]^+$ $[M+Na]^+$: 270.1464; found: 270.1458.

1-(4-Iodophenyl)ethan-1-one *O*-pivaloyl oxime (2v)

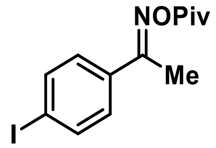

Following general procedure **GP2**, the title compound was prepared from 1-(4-iodophenyl)ethan-1-one (1.23 g, 5.00 mmol, 1.0 equiv), NaOAc (820 mg, 10.0 mmol, 2.0 equiv), and hydroxylamine hydrochloride (521 mg, 7.50 mmol, 1.5 equiv) in ethanol/H_2O (4:1; 10 mL, 0.50 M).

The obtained oxime (1.16 mg, 4.44 mmol, 1.0 equiv) was reacted with triethylamine (1.34 g, 13.2 mmol, 3.0 equiv) and pivaloyl chloride (1.07 g, 8.87 mmol, 2.0 equiv) in CH_2Cl_2 (18 mL, 0.25 M). The title compound was obtained as a white solid (1.28 g, 3.71 mmol, 74%) after column chromatography (*n*-pentane/ethyl acetate = 20:1).

^{1}H NMR (400 MHz, $CDCl_3$) δ 7.77 – 7.70 (m, 2H), 7.54 – 7.46 (m, 2H), 2.35 (s, 3H), 1.33 (s, 9H).

^{13}C{^{1}H} NMR (101 MHz, $CDCl_3$) δ 175.1, 162.3, 137.9, 134.6, 128.7, 97.3, 39.0, 27.4, 14.2.

HRMS (ESI): *m/z* calculated for $[C_{13}H_{16}INO_2Na]^+$ $[M+Na]^+$: 368.0118; found: 368.0100.

1-(4-(Methylthio)phenyl)ethan-1-one *O*-pivaloyl oxime (2w)

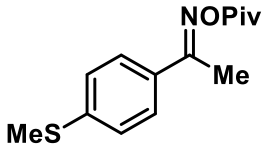

Following general procedure **GP2**, the title compound was prepared from 1-(4-(methylthio)phenyl)ethan-1-one (831 mg, 5.00 mmol, 1.0 equiv), NaOAc (820 mg, 10.0 mmol, 2.0 equiv), and hydroxylamine hydrochloride (521 mg, 7.50 mmol, 1.5 equiv) in ethanol/H_2O (4:1; 10 mL, 0.50 M).

The obtained oxime (899 mg, 4.96 mmol, 1.0 equiv) was reacted with triethylamine (1.51 g, 14.9 mmol, 3.0 equiv) and pivaloyl chloride (1.20 g, 9.95 mmol, 2.0 equiv) in CH_2Cl_2 (20 mL, 0.25 M). The title compound was obtained as an off-white solid (1.17 g, 4.41 mmol, 88%) after column chromatography (*n*-pentane/diethyl ether = 100:1 to 6:1).

^{1}H NMR (400 MHz, $CDCl_3$) δ 7.73 – 7.65 (m, 2H), 7.26 – 7.21 (m, 2H), 2.49 (s, 3H), 2.35 (s, 3H), 1.33 (s, 9H).

^{13}C{^{1}H} NMR (101 MHz, $CDCl_3$) δ 175.2, 162.6, 142.2, 131.3, 127.4, 125.8, 39.0, 27.4, 15.3, 14.2.

HRMS (ESI): *m/z* calculated for $[C_{14}H_{19}NNaO_2S]^+$ $[M+Na]^+$: 288.1029; found: 288.1021.

4-(1-((Pivaloyloxy)imino)ethyl)benzonitrile (2x)

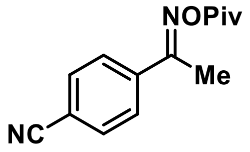

Following general procedure **GP2**, the title compound was prepared from 4-acetylbenzonitrile (435 mg, 3.00 mmol, 1.0 equiv), NaOAc (492 mg, 6.00 mmol, 2.0 equiv) and hydroxylamine hydrochloride (313 mg, 4.50 mmol, 1.5 equiv) in 4:1 ethanol/H_2O (6 mL, 0.50 M).

The obtained oxime (451 mg, 2.82 mmol, 1.0 equiv) was reacted with triethylamine (855 mg, 8.45 mmol, 3.0 equiv) and pivaloyl chloride (679 mg, 5.63 mmol, 2.0 equiv) in CH_2Cl_2 (11 mL, 0.26 M). The title compound was obtained as a white solid (476 mg, 1.95 mmol, 65%) after column chromatography (*n*-pentane/ethyl acetate = 100:1 to 9:1).

^{1}H NMR (400 MHz, $CDCl_3$) δ 7.92 – 7.84 (m, 2H), 7.75 – 7.66 (m, 2H), 2.40 (s, 3H), 1.34 (s, 9H).

^{13}C{^{1}H} NMR (101 MHz, $CDCl_3$) δ 174.9, 161.4, 139.4, 132.5, 127.8, 118.4, 114.2, 39.0, 27.4, 14.3.

HRMS (ESI): *m/z* calculated for $[C_{14}H_{16}N_2NaO_2]^+$ $[M+Na]^+$: 267.1104; found: 267.1099.

1-(4-(Methylsulfonyl)phenyl)ethan-1-one *O*-pivaloyl oxime (2y)

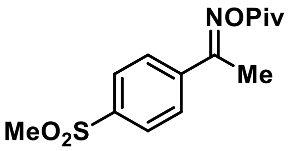

Following general procedure **GP2**, the title compound was prepared from 1-(4-(methylsulfonyl)phenyl)ethan-1-one (991 mg, 5.00 mmol, 1.0 equiv), NaOAc (820 mg, 10.0 mmol, 2.0 equiv), and hydroxylamine hydrochloride (521 mg, 7.50 mmol, 1.5 equiv) in ethanol/H_2O (4:1; 10 mL, 0.50 M).

The obtained oxime (1.03 g, 4.83 mmol, 1.0 equiv) was reacted with triethylamine (1.47 g, 14.5 mmol, 3.0 equiv) and pivaloyl chloride (1.17 g, 9.70 mmol, 2.0 equiv) in CH_2Cl_2 (19 mL, 0.25 M). The title compound was obtained as an off-white solid (639 mg, 2.15 mmol, 43%) after column chromatography (*n*-pentane/diethyl ether = 3:2 to 100% diethyl ether).

^{1}H NMR (400 MHz, $CDCl_3$) δ 8.01 – 7.92 (m, 4H), 3.06 (s, 3H), 2.42 (s, 3H), 1.34 (s, 9H).

^{13}C{^{1}H} NMR (101 MHz, $CDCl_3$) δ 174.9, 161.5, 142.2, 140.4, 128.1, 127.8, 44.6, 39.0, 27.4, 14.5.

HRMS (ESI): *m/z* calculated for $[C_{14}H_{19}NNaO_4S]^+$ $[M+Na]^+$: 320.0927; found: 320.0920.

1-(Benzo[*d*][1,3]dioxol-5-yl)ethan-1-one *O*-pivaloyl oxime (2z)

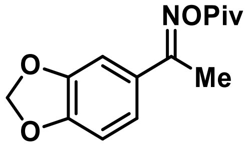

Following general procedure **GP2**, the title compound was prepared from 1-(benzo[*d*][1,3]dioxol-5-yl)ethan-1-one (821 mg, 5.00 mmol, 1.0 equiv), NaOAc (820 mg, 10.0 mmol, 2.0 equiv), and hydroxylamine hydrochloride (521 mg, 7.50 mmol, 1.5 equiv) in ethanol/H_2O (4:1; 10 mL, 0.50 M).

The obtained oxime (410 mg, 2.29 mmol, 1.0 equiv) was reacted with triethylamine (690 mg, 6.82 mmol, 3.0 equiv) and pivaloyl chloride (552 mg, 4.58 mmol, 2.0 equiv) in CH_2Cl_2 (9.2 mL, 0.25 M). The title compound was obtained as a white solid (498 mg, 1.89 mmol, 38%) after column chromatography (*n*-pentane/ethyl acetate = 5:1).

^{1}H NMR (400 MHz, $CDCl_3$) δ 7.34 (d, J = 1.7 Hz, 1H), 7.24 (dd, J = 8.2, 1.8 Hz, 1H), 6.82 (d, J = 8.2 Hz, 1H), 6.00 (s, 2H), 2.33 (s, 3H), 1.33 (s, 9H).

^{13}C{^{1}H} NMR (101 MHz, $CDCl_3$) δ 175.2, 162.5, 149.9, 148.1, 129.1, 121.9, 108.2, 107.3, 101.7, 39.0, 27.5, 14.4.

HRMS (ESI): *m/z* calculated for $[C_{14}H_{17}NO_4Na]^+$ $[M+Na]^+$: 286.1050; found: 286.1038.

1-(Naphthalen-2-yl)ethan-1-one *O*-pivaloyl oxime (2aa)

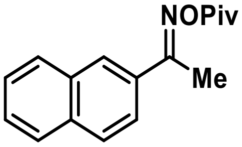

Following general procedure **GP2**, the title compound was prepared from 1-(naphthalen-2-yl)ethan-1-one (851 mg, 5.00 mmol, 1.0 equiv), NaOAc (820 mg, 10.0 mmol, 2.0 equiv), and hydroxylamine hydrochloride (521 mg, 7.50 mmol, 1.5 equiv) in ethanol/H_2O (4:1; 10 mL, 0.50 M).

The obtained oxime (890 mg, 4.80 mmol, 1.0 equiv) was reacted with triethylamine (1.45 g, 14.3 mmol, 3.0 equiv) and pivaloyl chloride (1.15 g, 9.54 mmol, 2.0 equiv) in CH_2Cl_2 (19 mL, 0.25 M). The title compound was obtained as a white solid (1.12 g, 4.16 mmol, 83%) after column chromatography (*n*-pentane/ethyl acetate = 40:1 to 20:1).

^{1}H NMR (400 MHz, $CDCl_3$) δ 8.20 – 8.14 (m, 1H), 8.01 – 7.96 (m, 1H), 7.92 – 7.81 (m, 3H), 7.59 –

7.46 (m, 2H), 2.50 (s, 3H), 1.37 (s, 9H).

$^{13}C\{^1H\}$ NMR (101 MHz, $CDCl_3$) δ 175.3, 163.0, 134.5, 133.0, 132.4, 128.9, 128.4, 127.8, 127.5, 127.4, 126.7, 124.0, 39.0, 27.5, 14.3.

HRMS (ESI): *m/z* calculated for $[C_{17}H_{19}NO_2Na]^+$ $[M+Na]^+$: 292.1308; found: 292.1293.

1,2-Diphenylethan-1-one *O*-pivaloyl oxime (2ab)

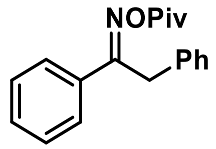

Following general procedure **GP2**, the title compound was prepared from 1,2-diphenylethan-1-one (981 mg, 5.00 mmol, 1.0 equiv), NaOAc (820 mg, 10.0 mmol, 2.0 equiv), and hydroxylamine hydrochloride (521 mg, 7.50 mmol, 1.5 equiv) in ethanol/H_2O (4:1; 10 mL, 0.50 M).

The obtained oxime (1.05 g, 4.97 mmol, 1.0 equiv) was reacted with triethylamine (1.51 g, 14.9 mmol, 3.0 equiv) and pivaloyl chloride (1.20 g, 9.95 mmol, 2.0 equiv) in CH_2Cl_2 (20 mL, 0.25 M). The title compound was obtained as an off-white solid (1.04 g, 3.52 mmol, 70%) after column chromatography (*n*-pentane/diethyl ether = 100:1 to 6:1).

^1H NMR (400 MHz, $CDCl_3$) δ 7.79 – 7.72 (m, 2H), 7.45 – 7.33 (m, 3H), 7.32 – 7.25 (m, 2H), 7.24 – 7.16 (m, 3H), 4.24 (s, 2H), 1.24 (s, 9H).

$^{13}C\{^1H\}$ NMR (101 MHz, $CDCl_3$) δ 175.1, 164.3, 135.4, 134.4, 130.7, 128.9, 128.7, 128.2, 127.6, 126.9, 38.8, 34.7, 27.3.

HRMS (ESI): *m/z* calculated for $[C_{19}H_{21}NNaO_2]^+$ $[M+Na]^+$: 318.1465; found: 318.1450.

Cyclopropyl(phenyl)methanone *O*-pivaloyl oxime (2ac)

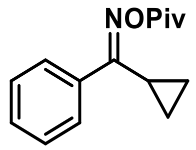

Following general procedure **GP2**, the title compound was prepared from cyclopropyl(phenyl)methanone (731 mg, 5.00 mmol, 1.0 equiv), NaOAc (820 mg, 10.0 mmol, 2.0 equiv), and hydroxylamine hydrochloride (521 mg, 7.50 mmol, 1.5 equiv) in ethanol/H_2O (4:1; 10 mL, 0.50 M).

The obtained oxime (790 mg, 4.90 mmol, 1.0 equiv) was reacted with triethylamine (1.49 g, 14.7 mmol, 3.0 equiv) and pivaloyl chloride (1.18 g, 9.79 mmol, 2.0 equiv) in CH_2Cl_2 (20 mL, 0.25 M). The title compound was obtained as a colorless oil (1.15 g, 4.69 mmol, 94%) after column chromatography (*n*-pentane/diethyl ether = 100:1 to 6:1) as a 73:27 mixture of geometrical isomers.

^{1}H NMR (599 MHz, $CDCl_3$) δ 7.45 – 7.33 (m, 5H^{major} + 3H^{minor}), 7.22 – 7.18 (m, 2H^{minor}), 2.29 (tt, J = 8.6, 5.4 Hz, 1H^{major}), 2.02 – 1.94 (m, 1H^{minor}), 1.35 (s, 9H^{major}), 1.03 – 0.98 (m, 2H^{major} + 9H^{minor}), 0.94 – 0.84 (m, 4H^{minor}), 0.72 – 0.68 (m, 2H^{major}).

^{13}C{^{1}H} NMR (151 MHz, $CDCl_3$) δ 175.3, 175.3, 170.5, 169.6, 132.6, 132.4, 129.7, 129.1, 129.0, 128.2, 128.1, 127.2, 39.0, 38.4, 27.5, 27.0, 15.7, 10.8, 6.4, 6.4.

HRMS (ESI): m/z calculated for $[C_{15}H_{19}NNaO_2]^+$ $[M+Na]^+$: 268.1308; found: 268.1302.

Cyclopentyl(phenyl)methanone *O*-pivaloyl oxime (2ad)

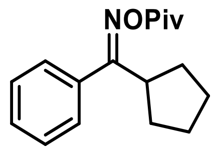

Following general procedure **GP2**, the title compound was prepared from cyclopentyl(phenyl)methanone (523 mg, 3.00 mmol, 1.0 equiv), NaOAc (492 mg, 6.00 mmol, 2.0 equiv) and hydroxylamine hydrochloride (313 mg, 4.50 mmol, 1.5 equiv) in 4:1 ethanol/H_2O (6 mL, 0.50 M).

The obtained oxime (565 mg, 2.99 mmol, 1.0 equiv) was reacted with triethylamine (906 mg, 8.95 mmol, 3.0 equiv) and pivaloyl chloride (720 mg, 5.97 mmol, 2.0 equiv) in CH_2Cl_2 (12 mL, 0.25 M). The title compound was obtained as a colorless oil (327 mg, 1.20 mmol, 40%) after column chromatography (*n*-pentane/ethyl acetate = 100:1 to 6:1).

^{1}H NMR (400 MHz, $CDCl_3$) δ 7.45 – 7.31 (m, 5H), 3.55 – 3.42 (m, 1H), 2.07 – 1.92 (m, 2H), 1.79 – 1.56 (m, 6H), 1.33 (s, 9H).

^{13}C{^{1}H} NMR (101 MHz, $CDCl_3$) δ 175.1, 171.7, 134.5, 129.4, 128.3, 128.2, 40.8, 38.9, 30.4, 27.4, 25.6.

HRMS (ESI): m/z calculated for $[C_{17}H_{23}NNaO_2]^+$ $[M+Na]^+$: 296.1621; found: 296.1608.

3,4-Dihydronaphthalen-1(2*H*)-one *O*-pivaloyl oxime (2ae)

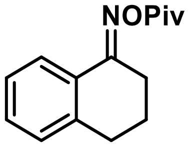

Following general procedure **GP2**, the title compound was prepared from 3,4-dihydronaphthalen-1(2*H*)-one (731 mg, 5.00 mmol, 1.0 equiv), NaOAc (820 mg, 10.0 mmol, 2.0 equiv), and hydroxylamine hydrochloride (521 mg, 7.50 mmol, 1.5 equiv) in ethanol/H_2O (4:1; 10 mL, 0.50 M).

The obtained oxime (743 mg, 4.61 mmol, 1.0 equiv) was reacted with triethylamine (1.40 g, 13.8 mmol, 3.0 equiv) and pivaloyl chloride (1.11 g, 9.21 mmol, 2.0 equiv) in CH_2Cl_2 (19 mL, 0.24 M). The title compound was obtained as a white solid (667 mg, 2.72 mmol, 54%) after column chromatography (*n*-pentane/ethyl acetate = 20:1).

^{1}H NMR (400 MHz, $CDCl_3$) δ 8.19 (dd, *J* = 7.9, 1.4 Hz, 1H), 7.33 (td, *J* = 7.4, 1.4 Hz, 1H), 7.23 (td, *J* = 7.6, 1.4 Hz, 1H), 7.16 (dd, *J* = 7.6, 1.2 Hz, 1H), 2.86 (t, *J* = 6.6 Hz, 2H), 2.82 – 2.73 (m, 2H), 1.94 – 1.84 (m, 2H), 1.33 (s, 9H).

^{13}C{^{1}H} NMR (101 MHz, $CDCl_3$) δ 175.2, 162.1, 140.9, 130.8, 129.2, 128.7, 126.7, 125.9, 39.0, 29.7, 27.5, 25.7, 21.5.

HRMS (ESI): *m/z* calculated for $[C_{15}H_{19}NO_2Na]^+$ $[M+Na]^+$: 268.1308; found: 268.1296.

6,7,8,9-Tetrahydro-5*H*-benzo[7]annulen-5-one *O*-pivaloyl oxime (2af)

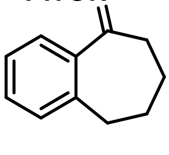

Following general procedure **GP2**, the title compound was prepared from 6,7,8,9-tetrahydro-5*H*-benzo[7]annulen-5-one (481 mg, 3.00 mmol, 1.0 equiv), NaOAc (492 mg, 6.00 mmol, 2.0 equiv) and hydroxylamine hydrochloride (313 mg, 4.50 mmol, 1.5 equiv) in 4:1 ethanol/H_2O (6 mL, 0.50 M).

The obtained oxime (467 mg, 2.67 mmol, 1.0 equiv) was reacted with triethylamine (806 mg, 7.96 mmol, 3.0 equiv) and pivaloyl chloride (640 mg, 5.31 mmol, 2.0 equiv) in CH_2Cl_2 (11 mL, 0.24 M). The title compound was obtained as a colorless oil (404 mg, 1.56 mmol, 52%) after column chromatography (*n*-pentane/ethyl acetate = 40:1 to 20:1).

^{1}H NMR (400 MHz, $CDCl_3$) δ 7.54 (dd, *J* = 7.6, 1.5 Hz, 1H), 7.33 (td, *J* = 7.5, 1.5 Hz, 1H), 7.27 – 7.21 (m, 1H), 7.17 – 7.09 (m, 1H), 2.84 – 2.72 (m, 4H), 1.85 – 1.74 (m, 2H), 1.73 – 1.62 (m, 2H), 1.34 (s, 9H).

^{13}C{^{1}H} NMR (101 MHz, $CDCl_3$) δ 175.2, 170.6, 139.7, 134.7, 130.3, 129.0, 128.5, 126.8, 39.0, 31.9, 28.0, 27.5, 25.9, 21.9.

HRMS (ESI): *m/z* calculated for $[C_{16}H_{21}NO_2Na]^+$ $[M+Na]^+$: 282.1465; found: 282.1450.

3. Reaction development

3.1 Reaction optimization

An oven-dried 10 mL Schlenk tube was charged with a Teflon-coated stirring bar, alkene **1a** (23.0 mg, 0.100 mmol, 1.0 equiv), reagent **2a** (46.4 mg, 0.150 mmol, 1.5 equiv), and thioxanthone (1.1 mg, 5.2 µmol, 5 mol%). The tube was evacuated and backfilled with argon three times. Dry ethyl acetate (0.50 mL, 0.20 M) was added and the mixture was then irradiated at 405 nm for 16 h. After that, the solvent was removed in vacuo and the residue was examined by ^{1}H NMR spectroscopy with mesitylene as internal standard. The tested variations of these conditions and the respective results are shown below.

Supplementary Table 1. Reaction condition optimization. Given yields were determined by ^{1}H NMR spectroscopy with mesitylene as internal standard.

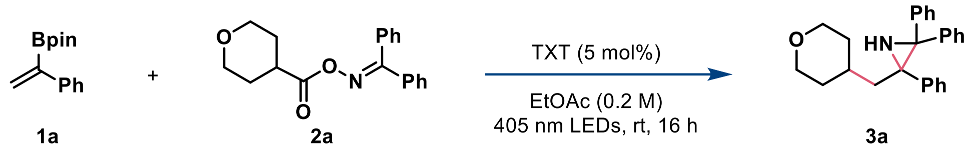

Entry	Deviations from standard conditions	Yield / %
1	none	72
2	4CzIPN (2 mol%) instead of TXT	61
3	[Ir(dF(CF$_3$)ppy)$_2$(dtbbpy)](PF$_6$) (1 mol%) instead of TXT, 450 nm LEDs	68
4	TXT (10 mol%)	67
5	380 nm LEDs	67
6	**1a** (1.5 equiv), **2a** (1.0 equiv)	66
7	**1a** (1.0 equiv), **2a** (1.0 equiv)	62
8	CH_2Cl_2 as solvent	60
9	w/o light	<5
10	w/o TXT	<5

3.2 Sensitivity assessment

The sensitivity assessment was conducted in a similar manner as reported by Glorius and coworkers.[14]

General procedure and results

An oven-dried 10 mL Schlenk tube was charged with a Teflon-coated stirring bar, alkene **1a** (23.0 mg, 0.100 mmol, 1.0 equiv), reagent **2a** (46.4 mg, 0.150 mmol, 1.5 equiv), and thioxanthone (1.1 mg, 5.2 µmol, 5 mol%). The tube was evacuated and backfilled with argon three times. Dry ethyl acetate (0.50 mL, 0.20 M) was added and the mixture was then irradiated at 405 nm for 16 h. After that, the solvent was removed in vacuo and the residue was examined by ^{1}H NMR

spectroscopy with an internal standard. The tested variations of these conditions and the respective results are shown below.

Supplementary Table 2. Deviations from the standard reaction conditions and corresponding relative deviations in yield from the control standard reaction.

Entry	Reaction	Deviation from the standard conditions	Relative deviation in yield from the standard reaction / %[a]
1	High *c*	0.45 mL EtOAc	0
2	Low *c*	0.55 mL EtOAc	-6
3	High H_2O	+5 µL H_2O	-100
4	Low O_2	Degassing by 3x freeze-pump-thaw	0
5	High O_2	Reaction prepared under air	-97
6	Low *T*	Water-cooled Schlenk tube	+9[b]
7	High *T*	Standard setup w/o fans turned on	0
8	Low *I*	One lamp 32 cm away from reaction vessel	-4
9	High *I*	One lamp 2 cm away from reaction vessel	-1

[a] Determined by ^{1}H NMR spectroscopy with an internal standard.

[b] Deviation from a control reaction in a water-cooled Schlenk tube w/o water flow.

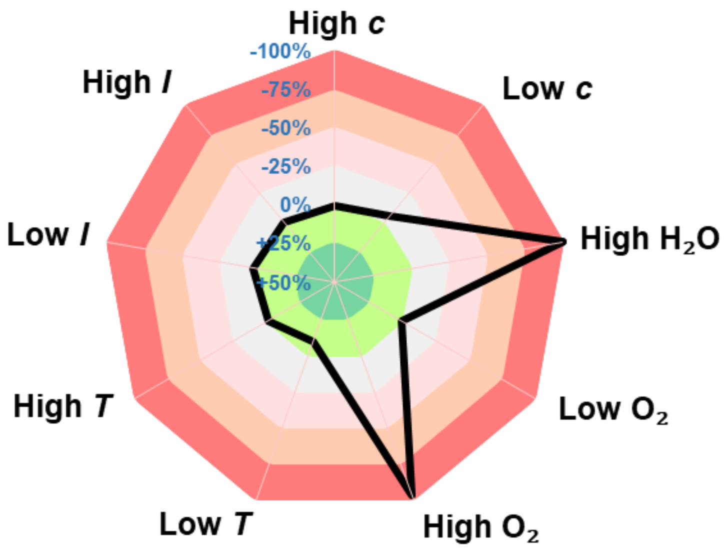

Supplementary Figure 4. Sensitivity assessment radar diagram.

<u>General lessons from the reaction development are described below:</u>

Note 1: In this assessment, the reaction was found to be particularly sensitive towards air and water. Consequently, the standard reaction conditions employed dry ethyl acetate, stored under argon over

molecular sieves, which was collected under positive argon pressure. To ensure appropriate exclusion of oxygen and moisture, the reactions were set up using the following procedure:

1. *The solids were added to the Schlenk tube under air*
2. *The atmosphere was exchanged with argon via three vacuum/argon cycles*
3. *The solvent was added under argon counterflow*
4. *Additional liquid components were added under argon counterflow, if applicable*
5. *The tube was tightly closed*

As additional degassing with freeze-pump-thaw cycles was found to be inconsequential, we did not degas the reaction mixtures.

Note 2: The hydrolytically labile Bpin (or TMS) group is likely still associated with the nitrogen atom of ***3a*** *in the crude but is cleaved during column chromatography (only free NH-aziridine* ***3a*** *was isolated). Throughout the SI,* ***3a*** *is depicted and described as the free NH-aziridine for consistency, even when only the crude was analyzed.*

Note 3: Crude 2D NMR analysis clearly proved that the aziridine ring of ***3a*** *was already formed in the crude.*

4. Substrate scope

General procedure GP3 for the synthesis of aziridines 3

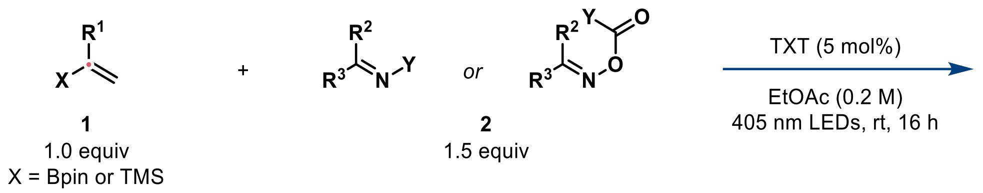

An oven-dried 10 mL Schlenk tube was charged with a Teflon-coated stirring bar, alkene **1** (1.0 equiv), reagent **2** (1.5 equiv), and thioxanthone (5 mol%). The tube was evacuated and backfilled with argon three times. Dry ethyl acetate (0.20 M) was added, and the mixture was then irradiated at 405 nm for 16 h. After that, the solvent was removed and the product was isolated by column chromatography on silica using the specified solvent system.

*Note: Liquid alkenes **1** were added to the tube after replacing the atmosphere with argon.*

4.1 Characterization data

Aziridine 3a

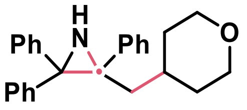

The title compound was prepared according to general procedure **GP3** from alkene **1a** (46.0 mg, 0.200 mmol, 1.0 equiv) and reagent **2a** (92.8 mg, 0.300 mmol, 1.5 equiv) with thioxanthone (2.2 mg, 10 µmol, 5 mol%) in ethyl acetate (1.0 mL, 0.20 M). Column chromatography (*n*-pentane/CH_2Cl_2/ethyl acetate = 88:10:2 to 75:10:15) gave the title compound as a white solid (47.5 mg, 0.129 mmol, 64%).

^{1}H NMR (400 MHz, $CDCl_3$) δ 7.58 – 7.48 (m, 2H), 7.41 – 7.34 (m, 2H), 7.32 – 7.27 (m, 3H), 7.24 – 7.19 (m, 2H), 7.17 – 7.11 (m, 2H), 7.08 – 7.00 (m, 1H), 7.00 – 6.94 (m, 2H), 6.93 – 6.86 (m, 1H), 3.83 (ddd, *J* = 11.4, 4.7, 1.9 Hz, 1H), 3.76 (ddd, *J* = 11.5, 4.7, 1.8 Hz, 1H), 3.16 (qd, *J* = 11.8, 2.3 Hz, 2H), 2.65 – 2.45 (m, 1H), 1.86 – 1.76 (m, 1H), 1.53 – 1.40 (m, 1H), 1.33 – 1.04 (m, 3H), 0.96 – 0.80 (m, 1H).

^{13}C{^{1}H} NMR (101 MHz, $CDCl_3$) δ 142.2, 140.9, 139.4, 128.8, 128.4, 128.4, 128.0, 127.9, 127.6, 127.3, 126.4, 126.1, 67.9, 67.8, 54.1, 51.8, 45.5, 33.8, 33.3, 33.0.

HRMS (ESI): *m/z* calculated for $[C_{26}H_{27}NONa]^+$ $[M+Na]^+$: 392.1985; found: 392.1986.

The same product **3a** was obtained from the reaction of **1b** (17.6 mg, 99.8 µmol, 1.0 equiv) and reagent **2a** (46.4 mg, 0.150 mmol, 1.5 equiv) with thioxanthone (1.1 mg, 5.2 µmol, 5 mol%) in ethyl acetate (0.5 mL, 0.20 M) to give a white solid (20.0 mg, 54.1 µmol, 54%).

Aziridine 3b

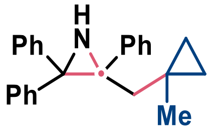

The title compound was prepared according to general procedure **GP3** from alkene **1a** (46.0 mg, 0.200 mmol, 1.0 equiv) and reagent **2b** (83.8 mg, 0.300 mmol, 1.5 equiv) with thioxanthone (2.2 mg, 10 µmol, 5 mol%) in ethyl acetate (1.0 mL, 0.20 M). Column chromatography (*n*-pentane/ethyl acetate = 200:1 to 25:1) gave the title compound as a white solid (36.0 mg, 0.106 mmol, 53%).

^{1}H NMR (400 MHz, $CDCl_3$) δ 7.57 – 7.52 (m, 2H), 7.42 – 7.35 (m, 2H), 7.35 – 7.32 (m, 2H), 7.30 – 7.21 (m, 3H), 7.15 – 7.08 (m, 2H), 7.06 – 6.94 (m, 3H), 6.92 – 6.84 (m, 1H), 2.13 (d, *J* = 14.7 Hz, 1H), 1.42 (dd, *J* = 14.6, 1.2 Hz, 1H), 1.01 (s, 3H), 0.07 – -0.02 (m, 2H), -0.08 – -0.16 (m, 1H), -0.17 – -0.28 (m, 1H).

^{13}C{^{1}H} NMR (151 MHz, $CDCl_3$) δ 142.4, 141.0, 140.2, 128.7, 128.7, 128.7, 128.4, 127.4, 127.4, 127.2, 126.1, 126.0, 54.0, 52.9, 47.8, 23.9, 14.3, 13.6, 12.5.

HRMS (ESI): *m/z* calculated for $[C_{25}H_{25}NNa]^+$ $[M+Na]^+$: 362.1879; found: 362.1879.

Note: This structure was additionally confirmed by single crystal X-Ray analysis.

Aziridine 3c

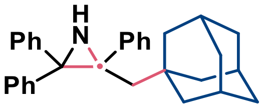

The title compound was prepared according to general procedure **GP3** from alkene **1a** (46.0 mg, 0.200 mmol, 1.0 equiv) and reagent **2c** (108 mg, 0.300 mmol, 1.5 equiv) with thioxanthone (2.2 mg, 10 µmol, 5 mol%) in ethyl acetate (1.0 mL, 0.20 M). Column chromatography (*n*-pentane/ethyl acetate = 500:1 to 150:1) gave the title compound as a white solid (58.4 mg, 0.139 mmol, 70%).

^{1}H NMR (400 MHz, $CDCl_3$) δ 7.58 – 7.51 (m, 2H), 7.41 – 7.33 (m, 4H), 7.30 – 7.22 (m, 3H), 7.09 (t,

J = 7.5 Hz, 2H), 7.01 – 6.92 (m, 3H), 6.86 (tt, J = 6.7, 1.2 Hz, 1H), 2.49 (d, J = 14.9 Hz, 1H), 1.82 – 1.71 (m, 3H), 1.60 – 1.52 (m, 3H), 1.51 – 1.42 (m, 3H), 1.41 – 1.33 (m, 3H), 1.29 – 1.20 (m, 3H), 0.92 (d, J = 14.9 Hz, 1H).

$^{13}C\{^1H\}$ NMR (101 MHz, $CDCl_3$) δ 142.5, 140.9, 140.8, 128.7, 128.6, 128.5, 128.4, 127.4, 127.4, 127.1, 126.0, 125.9, 54.4, 51.8, 51.0, 43.6, 36.9, 35.1, 28.7.

HRMS (ESI): *m/z* calculated for $[C_{31}H_{33}NNa]^+$ $[M+Na]^+$: 442.2505; found: 442.2505.

Aziridine 3d

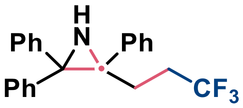

The title compound was prepared according to general procedure **GP3** from alkene **1a** (46.0 mg, 0.200 mmol, 1.0 equiv) and reagent **2d** (92.2 mg, 0.300 mmol, 1.5 equiv) with thioxanthone (2.2 mg, 10 µmol, 5 mol%) in ethyl acetate (1.0 mL, 0.20 M). Column chromatography (*n*-pentane/ethyl acetate = 50:1 to 15:1) gave the title compound as a white solid (32.9 mg, 89.5 µmol, 45%).

^1H NMR (400 MHz, $CDCl_3$) δ 7.62 – 7.54 (m, 2H), 7.45 – 7.35 (m, 2H), 7.32 – 7.24 (m, 3H), 7.23 – 7.16 (m, 4H), 7.14 – 7.07 (m, 1H), 7.04 – 6.98 (m, 2H), 6.97 – 6.91 (m, 1H), 2.53 (t, J = 12.9 Hz, 1H), 2.25 – 2.09 (m, 1H), 2.05 – 1.85 (m, 1H), 1.42 (td, J = 13.4, 4.9 Hz, 1H).

$^{13}C\{^1H\}$ NMR (101 MHz, $CDCl_3$) δ 141.1, 140.6, 137.9, 128.9, 128.4, 128.3, 128.2, 127.9, 127.8, 127.6, 127.2, 127.0 (q, J = 276.4 Hz), 126.6, 56.9, 52.3, 31.0 (q, J = 29.3 Hz), 30.5.

$^{19}F\{^1H\}$ NMR (376 MHz, $CDCl_3$) δ -66.3.

HRMS (ESI): *m/z* calculated for $[C_{23}H_{20}NF_3Na]^+$ $[M+Na]^+$: 390.1440; found: 390.1441.

Aziridine 3e

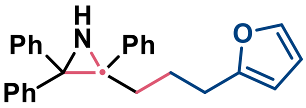

The title compound was prepared according to general procedure **GP3** from alkene **1a** (46.0 mg, 0.200 mmol, 1.0 equiv) and reagent **2e** (95.8 mg, 0.300 mmol, 1.5 equiv) with thioxanthone (2.2 mg, 10 µmol, 5 mol%) in ethyl acetate (1.0 mL, 0.20 M). Column chromatography (*n*-

pentane/ethyl acetate = 100:1 to 30:1) gave the title compound as a light-yellow sticky solid (36.6 mg, 96.4 µmol, 48%).

^{1}H NMR (400 MHz, $CDCl_3$) δ 7.59 – 7.53 (m, 2H), 7.42 – 7.34 (m, 2H), 7.33 – 7.27 (m, 3H), 7.24 – 7.19 (m, 3H), 7.18 – 7.12 (m, 2H), 7.09 – 7.03 (m, 1H), 7.02 – 6.96 (m, 2H), 6.95 – 6.89 (m, 1H), 6.19 (dd, J = 3.2, 1.8 Hz, 1H), 5.81 (dd, J = 3.0, 1.0 Hz, 1H), 2.58 – 2.33 (m, 3H), 1.77 – 1.50 (m, 2H), 1.34 – 1.14 (m, 1H).

^{13}C{^{1}H} NMR (101 MHz, $CDCl_3$) δ 155.9, 141.9, 140.9, 140.8, 139.1, 128.7, 128.6, 128.3, 128.3, 127.8, 127.6, 127.3, 126.5, 126.2, 110.1, 104.9, 56.2, 53.6, 37.5, 27.7, 25.0.

HRMS (ESI): m/z calculated for $[C_{27}H_{25}NONa]^+$ $[M+Na]^+$: 402.1828; found: 402.1826.

Aziridine 3f

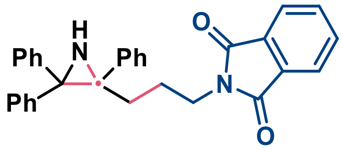

The title compound was prepared according to general procedure **GP3** from alkene **1a** (46.0 mg, 0.200 mmol, 1.0 equiv) and reagent **2f** (120 mg, 0.301 mmol, 1.5 equiv) with thioxanthone (2.2 mg, 10 µmol, 5 mol%) in ethyl acetate (1.0 mL, 0.20 M). Column chromatography (*n*-pentane/ethyl acetate = 10:1 to 3:1) gave the title compound as a white solid (50.0 mg, 0.109 mmol, 55%).

^{1}H NMR (400 MHz, $CDCl_3$) δ 7.79 – 7.73 (m, 2H), 7.69 – 7.63 (m, 2H), 7.58 – 7.53 (m, 2H), 7.35 – 7.26 (m, 4H), 7.23 – 7.16 (m, 3H), 7.15 – 7.09 (m, 2H), 7.06 – 6.95 (m, 3H), 6.94 – 6.87 (m, 1H), 3.53 (t, J = 7.2 Hz, 2H), 2.41 – 2.24 (m, 1H), 1.76 – 1.53 (m, 2H), 1.30 – 1.19 (m, 1H).

^{13}C{^{1}H} NMR (101 MHz, $CDCl_3$) δ 168.3, 141.6, 140.8, 138.8, 133.9, 132.1, 128.6, 128.6, 128.3, 128.3, 127.9, 127.6, 127.3, 126.6, 126.2, 123.2, 56.0, 53.3, 37.7, 35.4, 25.6.

HRMS (ESI): m/z calculated for $[C_{31}H_{26}N_2O_2Na]^+$ $[M+Na]^+$: 481.1887; found: 481.1888.

Aziridine 3g

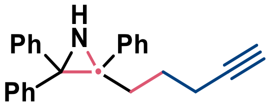

The title compound was prepared according to general procedure **GP3** from alkene **1a** (46.0 mg, 0.200 mmol, 1.0 equiv) and reagent **2g** (83.2 mg, 0.300 mmol, 1.5 equiv) with thioxanthone (2.2 mg, 10 µmol, 5 mol%) in ethyl acetate (1.0 mL, 0.20 M). Column chromatography (*n*-pentane/CH_2Cl_2/ethyl acetate = 50:50:1) gave the title compound as a colorless oil (31.1 mg, 92.2 µmol, 46%).

^{1}H NMR (599 MHz, $CDCl_3$) δ 7.59 – 7.55 (m, 2H), 7.40 – 7.34 (m, 2H), 7.31 – 7.26 (m, 3H), 7.23 – 7.19 (m, 2H), 7.17 – 7.12 (m, 2H), 7.07 – 7.02 (m, 1H), 7.01 – 6.96 (m, 2H), 6.94 – 6.87 (m, 1H), 2.47 (t, *J* = 10.2 Hz, 1H), 2.07 (td, *J* = 7.3, 3.1 Hz, 2H), 1.85 (t, *J* = 2.6 Hz, 1H), 1.56 – 1.41 (m, 2H), 1.29 – 1.23 (m, 1H).

^{13}C{^{1}H} NMR (151 MHz, $CDCl_3$) δ 142.2, 141.1, 139.1, 128.7, 128.6, 128.4, 128.2, 127.9, 127.6, 127.3, 126.5, 126.2, 84.1, 69.0, 55.8, 52.9, 36.8, 25.2, 18.2.

HRMS (ESI): *m/z* calculated for $[C_{25}H_{23}NNa]^+$ $[M+Na]^+$: 360.1723; found: 360.1703.

Aziridine 3h

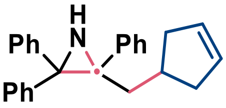

The title compound was prepared according to general procedure **GP3** from alkene **1a** (46.0 mg, 0.200 mmol, 1.0 equiv) and reagent **2h** (87.3 mg, 0.300 mmol, 1.5 equiv) with thioxanthone (2.2 mg, 10 µmol, 5 mol%) in ethyl acetate (1.0 mL, 0.20 M). Column chromatography (*n*-pentane/ethyl acetate = 200:1 to 25:1) gave the title compound as a white solid (35.9 mg, 0.102 mmol, 51%).

^{1}H NMR (500 MHz, $CDCl_3$) δ 7.59 – 7.55 (m, 2H), 7.41 – 7.35 (m, 2H), 7.34 – 7.30 (m, 2H), 7.30 – 7.25 (m, 1H), 7.25 – 7.20 (m, 2H), 7.16 – 7.10 (m, 2H), 7.06 – 7.01 (m, 1H), 7.00 – 6.95 (m, 2H), 6.92 – 6.86 (m, 1H), 5.60 – 5.54 (m, 1H), 5.53 – 5.47 (m, 1H), 2.70 (d, *J* = 14.3 Hz, 1H), 2.41 (dd, *J* = 16.0, 7.4 Hz, 1H), 2.26 – 2.11 (m, 2H), 2.06 – 1.98 (m, 1H), 1.73 – 1.61 (m, 1H), 1.19 – 1.11 (m, 1H).

^{13}C{^{1}H} NMR (126 MHz, $CDCl_3$) δ 142.2, 141.1, 139.8, 130.2, 130.1, 128.7, 128.6, 128.3, 128.3, 127.7, 127.5, 127.3, 126.3, 126.1, 54.6, 53.3, 44.4, 39.6, 38.4, 36.4.

HRMS (ESI): *m/z* calculated for $[C_{26}H_{25}NNa]^+$ $[M+Na]^+$: 374.1879; found: 374.1881.

Aziridine 3i

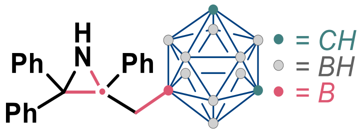

The title compound was prepared according to a modified general procedure **GP3** from alkene **1a** (46.0 mg, 0.200 mmol, 2.0 equiv) and reagent **2i** (36.7 mg, 99.9 µmol, 1.0 equiv) in ethyl acetate (2.0 mL, 50 mM). 2-Isopropylthioxanthone (1.3 mg, 5.1 µmol, 5 mol%) was used instead of thioxanthone and the reaction was irradiated at 380 nm. Column chromatography (*n*-pentane/ethyl acetate = 50:1 to 20:1) gave the title compound as a white solid (27.8 mg, 65.0 µmol, 65%).

^{1}H NMR (400 MHz, $CDCl_3$) δ 7.59 – 7.51 (m, 2H), 7.41 – 7.33 (m, 4H), 7.29 – 7.21 (m, 3H), 7.13 – 7.07 (m, 2H), 7.04 – 6.94 (m, 3H), 6.93 – 6.86 (m, 1H), 2.88 – 2.62 (m, 2H), 2.27 (d, *J* = 15.6 Hz, 1H), 0.89 (d, *J* = 15.6 Hz, 1H), 3.42 – 0.58 (m, 9H).

^{13}C{^{1}H} NMR (101 MHz, $CDCl_3$) δ 142.5, 141.4, 140.0, 128.8, 128.7, 128.6, 128.3, 127.5, 127.3, 127.1, 126.0, 126.0, 57.6, 54.4, 54.3, 53.8, 23.7.

^{11}B{^{1}H} NMR (128 MHz, $CDCl_3$) δ -1.1, -6.4, -9.8, -13.3, -14.1, -17.7, -19.8.

HRMS (ESI): *m/z* calculated for $[C_{23}H_{29}B_{10}NNa]^+$ $[M+Na]^+$: 450.3195; found: 450.3192.

Note: This structure was additionally confirmed by single crystal X-Ray analysis.

Aziridine 3j

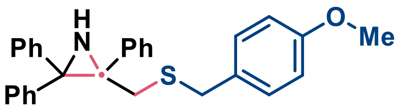

The title compound was prepared according to general procedure **GP3** from alkene **1a** (46.0 mg, 0.200 mmol, 2.0 equiv) and reagent **2j** (113 mg, 0.299 mmol, 1.5 equiv) with thioxanthone (2.2 mg, 10 µmol, 5 mol%) in ethyl acetate (1.0 mL, 0.20 M). Column chromatography (*n*-pentane/ethyl acetate = 30:1 to 12:1) gave the title compound as a light-yellow gum (20.9 mg, 47.8 µmol, 24%).

^{1}H NMR (400 MHz, $CDCl_3$) δ 7.53 (d, *J* = 7.5 Hz, 2H), 7.40 – 7.33 (m, 4H), 7.30 – 7.25 (m, 1H), 7.24

– 7.16 (m, 4H), 7.15 – 7.09 (m, 1H), 7.06 – 6.98 (m, 2H), 6.97 – 6.91 (m, 3H), 6.76 – 6.68 (m, 2H), 3.77 (s, 3H), 3.34 (d, *J* = 13.3 Hz, 1H), 3.27 – 3.14 (m, 2H), 2.49 (d, *J* = 13.9 Hz, 1H).

^{13}C{^{1}H} NMR (101 MHz, $CDCl_3$) δ 158.6, 140.5, 140.0, 138.3, 130.1, 130.0, 128.8, 128.8, 128.7, 128.1, 127.9, 127.7, 127.6, 127.1, 126.5, 113.9, 57.9, 55.4, 53.7, 39.4, 36.1.

HRMS (ESI): *m/z* calculated for $[C_{29}H_{27}NOSNa]^+$ $[M+Na]^+$: 460.1706; found: 460.1705.

Aziridine 3k

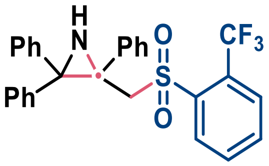

The title compound was prepared according to general procedure **GP3** from alkene **1b** (35.2 mg, 0.200 mmol, 1.0 equiv) and reagent **2k** (117 mg, 0.300 mmol, 1.5 equiv) with thioxanthone (2.2 mg, 10 µmol, 5 mol%) in ethyl acetate (1.0 mL, 0.20 M). Column chromatography (100% *n*-pentane to *n*-pentane/ethyl acetate = 9:1) gave the title compound as a light-yellow solid (40.2 mg, 81.5 µmol, 41%).

^{1}H NMR (400 MHz, $CDCl_3$) δ 8.09 – 8.03 (m, 1H), 7.52 – 7.47 (m, 5H), 7.43 – 7.37 (m, 2H), 7.34 – 7.28 (m, 1H), 7.20 – 7.14 (m, 2H), 6.98 – 6.86 (m, 5H), 6.84 – 6.74 (m, 3H), 4.64 (d, *J* = 14.9 Hz, 1H), 3.21 (d, *J* = 15.0 Hz, 1H).

^{13}C{^{1}H} NMR (126 MHz, $CDCl_3$) δ 140.3, 139.3, 137.7 (q, *J* = 1.2 Hz), 136.2, 134.0, 133.6, 132.3, 129.4, 128.8 (q, *J* = 33.2 Hz), 128.6, 128.2, 128.1, 127.9 (q, *J* = 6.2 Hz), 127.9, 127.6, 127.5, 126.6, 126.6, 122.6 (d, *J* = 273.8 Hz), 62.4 (q, *J* = 2.9 Hz), 56.9, 46.5.

^{19}F{^{1}H} NMR (470 MHz, $CDCl_3$) δ -56.8.

HRMS (ESI): *m/z* calculated for $[C_{28}H_{22}F_3NO_2SNa]^+$ $[M+Na]^+$: 516.1216; found: 516.1212.

Aziridine 3l

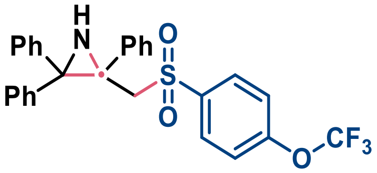

The title compound was prepared according to general procedure **GP3** from alkene **1b** (35.2 mg, 0.200 mmol, 1.0 equiv) and reagent **2l** (122 mg, 0.301 mmol, 1.5 equiv) with thioxanthone

(2.2 mg, 10 μmol, 5 mol%) in ethyl acetate (1.0 mL, 0.20 M). Column chromatography (*n*-pentane/CH_2Cl_2/ethyl acetate = 8:2:0.1 to 8:2:0.4) gave the title compound as a white solid (37.9 mg, 74.4 μmol, 37%).

^{1}H NMR (500 MHz, CD_2Cl_2) δ 7.60 – 7.55 (m, 2H), 7.53 – 7.50 (m, 2H), 7.46 – 7.40 (m, 2H), 7.36 – 7.30 (m, 1H), 7.24 – 7.18 (m, 2H), 7.08 – 6.83 (m, 10H), 4.38 (d, *J* = 15.1 Hz, 1H), 3.21 – 3.06 (m, 1H).

^{13}C{^{1}H} NMR (126 MHz, CD_2Cl_2) δ 152.9 (q, *J* = 1.7 Hz), 140.9, 139.8, 138.0, 136.9, 130.9, 129.6, 128.9, 128.5, 128.4, 128.3, 127.9, 127.9, 127.0, 126.9, 121.0 (q, *J* = 1.1 Hz), 120.6 (q, *J* = 259.2 Hz), 62.9, 56.7, 47.0.

^{19}F{^{1}H} NMR (377 MHz, CD2Cl$_2$) δ -58.0.

HRMS (ESI): *m/z* calculated for $[C_{28}H_{22}NO_3SF_3Na]^+$ $[M+Na]^+$: 532.1165; found: 532.1165.

Aziridine 3m

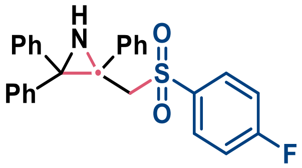

The title compound was prepared according to general procedure **GP3** from alkene **1b** (17.6 mg, 99.8 μmol, 1.0 equiv) and reagent **2m** (50.9 mg, 0.150 mmol, 1.5 equiv) with thioxanthone (1.1 mg, 5.2 μmol, 5 mol%) in ethyl acetate (0.50 mL, 0.20 M). Column chromatography (100% *n*-pentane to *n*-pentane/ethyl acetate = 9:1) gave the title compound as a yellowish solid (19.0 mg, 42.8 μmol, 43%).

^{1}H NMR (500 MHz, CD_2Cl_2) δ 7.57 – 7.49 (m, 4H), 7.44 – 7.39 (m, 2H), 7.35 – 7.30 (m, 1H), 7.22 – 7.18 (m, 2H), 7.05 – 7.00 (m, 2H), 6.99 – 6.94 (m, 2H), 6.94 – 6.88 (m, 6H), 4.34 (d, *J* = 15.1 Hz, 1H), 3.11 (d, *J* = 15.1 Hz, 1H).

^{13}C{^{1}H} NMR (126 MHz, CD_2Cl_2) δ 166.0 (d, *J* = 255.2 Hz), 140.9, 139.8, 137.1, 135.8, 131.5 (d, *J* = 9.9 Hz), 129.6, 128.9, 128.5, 128.4, 128.3, 127.9, 127.9, 127.0, 126.9, 116.4 (d, *J* = 22.8 Hz), 62.8, 56.7, 47.1.

^{19}F{^{1}H} NMR (470 MHz, CD_2Cl_2) δ -105.1.

HRMS (ESI): *m/z* calculated for $[C_{27}H_{22}NO_2SFNa]^+$ $[M+Na]^+$: 466.1248; found: 466.1261.

Aziridine 3n

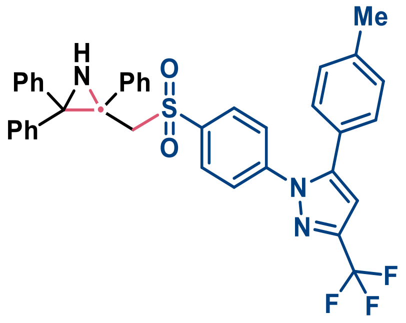

The title compound was prepared according to general procedure **GP3** from alkene **1b** (17.7 mg, 0.100 mmol, 1.0 equiv) and reagent **2n** (82.0 mg, 0.150 mmol, 1.5 equiv) with thioxanthone (1.1 mg, 5.2 µmol, 5 mol%) in ethyl acetate (0.50 mL, 0.20 M). Column chromatography (*n*-pentane/ethyl acetate = 10:1 to 5:1) gave the title compound as a white solid (30.2 mg, 46.5 µmol, 46%).

^{1}H NMR (400 MHz, $CDCl_3$) δ 7.58 – 7.46 (m, 4H), 7.41 – 7.35 (m, 2H), 7.33 – 7.27 (m, 1H), 7.23 – 7.13 (m, 6H), 7.06 – 7.02 (m, 4H), 7.00 – 6.86 (m, 6H), 6.71 (s, 1H), 4.29 (d, *J* = 15.1 Hz, 1H), 3.19 (d, *J* = 14.9 Hz, 1H), 2.38 (s, 3H).

^{13}C{^{1}H} NMR (101 MHz, $CDCl_3$) δ 145.2, 144.3 (q, *J* = 38.6 Hz), 143.2, 140.1, 139.9, 139.1, 138.7, 136.3, 129.8, 129.5, 129.3, 128.8, 128.6, 128.2, 127.9, 127.9, 127.7, 127.2, 126.8, 125.9, 124.9, 121.2 (d, *J* = 269.1 Hz), 106.6 (q, *J* = 1.9 Hz), 62.5, 56.5, 47.1, 21.5.

^{19}F{^{1}H} NMR (376 MHz, $CDCl_3$) δ -62.5.

HRMS (ESI): *m/z* calculated for $[C_{38}H_{30}N_3O_2SF_3Na]^+$ $[M+Na]^+$: 672.1903; found: 672.1904.

Aziridine 3o

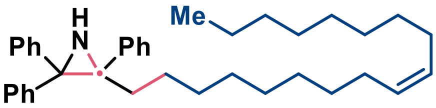

The title compound was prepared according to general procedure **GP3** from alkene **1a** (46.0 mg, 0.200 mmol, 1.0 equiv) and reagent **2o** (139 mg, 0.301 mmol, 1.5 equiv) with thioxanthone (2.2 mg, 10 µmol, 5 mol%) in ethyl acetate (1.0 mL, 0.20 M). Column chromatography (*n*-pentane/ethyl acetate = 200:1 to 25:1) gave the title compound as a colorless oil (43.8 mg, 83.9 µmol, 42%).

^{1}H NMR (400 MHz, $CDCl_3$) δ 7.60 – 7.52 (m, 2H), 7.42 – 7.34 (m, 2H), 7.30 – 7.23 (m, 3H), 7.22 – 7.17 (m, 2H), 7.16 – 7.10 (m, 2H), 7.07 – 7.01 (m, 1H), 7.00 – 6.93 (m, 2H), 6.93 – 6.84 (m, 1H),

5.46 – 5.24 (m, 2H), 2.33 (t, *J* = 10.8 Hz, 1H), 2.08 – 1.90 (m, 4H), 1.41 – 1.03 (m, 25H), 0.88 (t, *J* = 6.8 Hz, 3H).

$^{13}C\{^{1}H\}$ NMR (151 MHz, $CDCl_3$) δ 142.5, 141.4, 139.7, 130.0, 130.0, 128.7, 128.7, 128.4, 128.3, 127.7, 127.5, 127.2, 126.3, 126.1, 55.9, 53.6, 38.7, 32.1, 29.9, 29.8, 29.8, 29.7, 29.6, 29.5, 29.5, 29.5, 29.3, 27.3, 27.3, 26.4, 22.8, 14.3.

HRMS (ESI): *m/z* calculated for $[C_{38}H_{51}NNa]^+$ $[M+Na]^+$: 544.3914; found: 544.3917.

Aziridine 3p

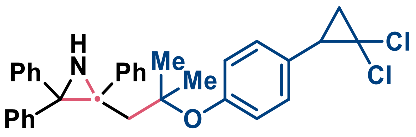

The title compound was prepared according to general procedure **GP3** from alkene **1a** (23.0 mg, 0.100 mmol, 1.0 equiv) and reagent **2p** (70.3 mg, 0.150 mmol, 1.5 equiv) with thioxanthone (1.1 mg, 5.2 µmol, 5 mol%) in ethyl acetate (0.50 mL, 0.20 M). Column chromatography (*n*-pentane/ethyl acetate = 200:1 to 25:1) gave the title compound as a colorless oil (26.9 mg, 50.9 µmol, 51%) in 50:50 d.r.

^{1}H NMR (599 MHz, $CDCl_3$) δ 7.56 – 7.52 (m, 2H), 7.43 – 7.40 (m, 2H), 7.39 – 7.35 (m, 2H), 7.29 – 7.25 (m, 3H), 7.11 (t, *J* = 7.5 Hz, 2H), 7.07 – 7.04 (m, 2H), 7.01 – 6.94 (m, 3H), 6.91 – 6.86 (m, 1H), 6.79 – 6.73 (m, 2H), 2.87 – 2.81 (m, 2H), 1.96 – 1.92 (m, 1H), 1.91 – 1.87 (m, 1H), 1.82 – 1.75 (m, 1H), 1.08 (s, 3H), 1.03 – 1.00 (m, 3H).

$^{13}C\{^{1}H\}$ NMR (101 MHz, $CDCl_3$) δ 154.3, 142.3, 141.2, 141.0, 129.7, 129.4, 128.8, 128.8, 128.5, 128.4, 127.6, 127.4, 127.2, 126.1, 126.0, 124.0, 82.4, 61.0, 55.5, 50.2, 47.8, 47.8, 35.1, 28.5, 28.5, 26.8, 26.0, 26.0.

HRMS (ESI): *m/z* calculated for $[C_{33}H_{31}NOCl_2Na]^+$ $[M+Na]^+$: 550.1675; found: 550.1643.

Aziridine 3q

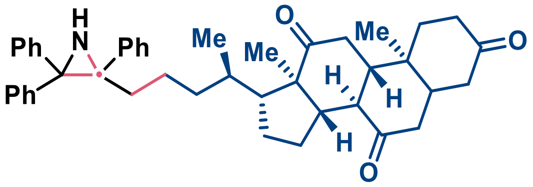

The title compound was prepared according to general procedure **GP3** from alkene **1a** (69.0 mg, 0.300 mmol, 1.0 equiv) and reagent **2q** (175 mg, 0.301 mmol, 1.0 equiv) with thioxanthone (3.2 mg, 15 µmol, 5 mol%) in ethyl acetate (1.5 mL, 0.20 M). Column chromatography (*n*-pentane/ethyl acetate = 7:1 to 1:1) gave the title compound as a light-yellow solid (64.6 mg, 0.101 mmol, 34%) in 55:45 d.r.

^{1}H NMR (400 MHz, $CDCl_3$) δ 7.59 – 7.55 (m, 2H), 7.40 – 7.34 (m, 2H), 7.30 – 7.24 (m, 3H), 7.22 – 7.18 (m, 2H), 7.16 – 7.10 (m, 2H), 7.06 – 7.01 (m, 1H), 7.00 – 6.94 (m, 2H), 6.93 – 6.86 (m, 1H), 2.96 – 2.73 (m, 3H), 2.38 – 1.71 (m, 16H), 1.65 – 1.52 (m, 1H), 1.36 (s, 3H), 1.34 – 0.99 (m, 5H), 0.97 – 0.94 (m, 3H), 0.91 – 0.77 (m, 1H), 0.71 – 0.60 (m, 3H).

^{13}C{^{1}H} NMR (101 MHz, $CDCl_3$) δ 212.1, 212.0, 209.1, 208.9, 208.9, 142.0, 141.0, 139.2, 128.7, 128.6, 128.6, 128.6, 128.3, 128.3, 128.2, 127.7, 127.7, 127.5, 127.5, 127.2, 127.2, 126.4, 126.3, 126.1, 126.1, 56.9, 56.9, 56.1, 53.8, 51.8, 49.1, 46.9, 46.0, 45.9, 45.6, 45.6, 45.1, 42.9, 38.9, 38.7, 36.6, 36.2, 36.2, 36.1, 35.5, 35.5, 35.3, 27.8, 25.2, 23.7, 23.6, 22.0, 19.1, 19.0, 11.9, 11.9.

HRMS (ESI): *m/z* calculated for $[C_{44}H_{51}NO_3Na]^+$ $[M+Na]^+$: 664.3761; found: 664.3764.

Aziridine 3r

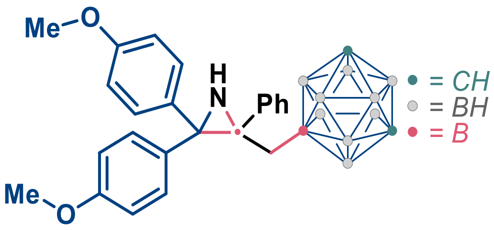

The title compound was prepared according to a modified general procedure **GP3** from alkene **1a** (46.0 mg, 0.200 mmol, 2.0 equiv) and reagent **2r** (42.8 mg, 0.100 mmol, 1.0 equiv) in ethyl acetate (2.0 mL, 50 mM). 2-Isopropylthioxanthone (1.3 mg, 5.1 µmol, 5 mol%) was used instead of thioxanthone and the reaction was irradiated at 380 nm. Column chromatography (*n*-pentane/ethyl acetate = 20:1 to 7:1) gave the title compound as a white solid (32.9 mg, 67.5 µmol, 67%).

^{1}H NMR (400 MHz, $CDCl_3$) δ 7.46 – 7.40 (m, 2H), 7.39 – 7.33 (m, 2H), 7.16 – 7.07 (m, 4H), 7.04 –

6.97 (m, 1H), 6.93 – 6.84 (m, 2H), 6.55 – 6.47 (m, 2H), 3.79 (s, 3H), 3.60 (s, 3H), 2.77 – 2.67 (m, 2H), 2.22 (d, *J* = 15.6 Hz, 1H), 0.91 (d, *J* = 15.5 Hz, 1H), 3.40 – 0.73 (m, 9H).

^{13}C{^{1}H} NMR (101 MHz, $CDCl_3$) δ 158.5, 157.5, 140.2, 135.2, 134.1, 129.6, 129.2, 128.6, 127.4, 126.0, 114.2, 113.0, 56.5, 55.4, 55.1, 54.4, 54.3, 54.1, 23.8.

^{11}B{^{1}H} NMR (128 MHz, $CDCl_3$) δ -1.1, -6.4, -9.8, -13.3, -14.1, -17.7, -19.6.

HRMS (ESI): *m/z* calculated for $[C_{25}H_{33}NO_2B_{10}Na]^+$ $[M+Na]^+$: 510.3419; found: 510.3408.

Aziridine 3s

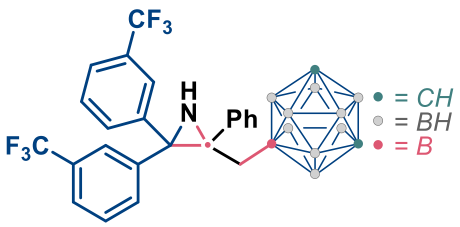

The title compound was prepared according to a modified general procedure **GP3** from alkene **1a** (46.0 mg, 0.200 mmol, 2.0 equiv) and reagent **2s** (50.3 mg, 99.9 µmol, 1.0 equiv) in ethyl acetate (2.0 mL, 50 mM). 2-Isopropylthioxanthone (1.3 mg, 5.1 µmol, 5 mol%) was used instead of thioxanthone and the reaction was irradiated at 380 nm. Column chromatography (*n*-pentane/ethyl acetate = 50:1 to 15:1) gave the title compound as a white solid (30.7 mg, 54.5 µmol, 54%).

^{1}H NMR (400 MHz, $CDCl_3$) δ 7.81 – 7.73 (m, 2H), 7.59 – 7.50 (m, 3H), 7.41 – 7.31 (m, 3H), 7.19 – 7.15 (m, 1H), 7.13 – 7.06 (m, 3H), 7.04 – 6.96 (m, 1H), 2.77 – 2.72 (m, 2H), 2.27 (d, *J* = 15.5 Hz, 1H), 0.80 (d, *J* = 15.5 Hz, 1H), 3.30 – 0.66 (m, 9H).

^{13}C{^{1}H} NMR (101 MHz, $CDCl_3$) δ 142.8, 141.8, 139.1, 132.1, 132.0, 131.3 (q, *J* = 32.5 Hz), 129.9 (q, *J* = 32.8 Hz), 129.5, 128.4, 128.1, 127.6, 126.5, 125.7 (q, *J* = 3.8 Hz), 125.2 (q, *J* = 3.7 Hz), 124.5 (q, *J* = 3.2 Hz), 124.1 (q, *J* = 272.5 Hz), 123.1 (q, *J* = 3.3 Hz), 56.2, 54.5, 54.4, 54.1, 23.5.

^{11}B{^{1}H} NMR (128 MHz, $CDCl_3$) δ -1.4, -6.4, -9.9, -13.3, -14.0, -17.7, -19.7.

^{19}F{^{1}H} NMR (376 MHz, $CDCl_3$) δ -62.6, -62.8.

HRMS (ESI): *m/z* calculated for $[C_{25}H_{27}NB_{10}F_6Na:]^+$ $[M+Na]^+$: 586.2943; found: 586.2949.

Aziridine 3t

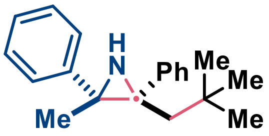

The title compound was prepared according to general procedure **GP3** from alkene **1a** (23.0 mg, 0.100 mmol, 1.0 equiv) and reagent **2t** (32.9 mg, 0.150 mmol, 1.5 equiv) in ethyl acetate (1.0 mL, 0.10 M). [Ir-F] (2.2 mg, 2.0 µmol, 2 mol%) was used instead of thioxanthone and the reaction was irradiated at 450 nm. Column chromatography (*n*-pentane/ethyl acetate = 30:1) gave the title compound as a brown oil (16.8 mg, 60.1 µmol, 60%).

^{1}H NMR (400 MHz, $CDCl_3$) δ 7.20 – 7.16 (m, 2H), 7.15 – 7.10 (m, 2H), 7.04 – 6.96 (m, 4H), 6.94 – 6.84 (m, 2H), 2.57 (d, *J* = 14.7 Hz, 1H), 1.82 (s, 3H), 1.75 (d, *J* = 14.8 Hz, 1H), 0.80 (s, 9H).

^{13}C{^{1}H} NMR (101 MHz, $CDCl_3$) δ 142.4, 141.3, 128.7, 127.5, 127.4, 127.2, 125.8, 125.8, 51.0, 47.7, 46.9, 32.8, 30.9, 23.5.

HRMS (ESI): *m/z* calculated for $[C_{20}H_{25}NH]^+$ $[M+H]^+$: 280.2060; found: 280.2060.

Note: The major diastereomer was isolated. Crude ^{1}H NMR analysis revealed a d.r. of 74:26.

Aziridine 3u

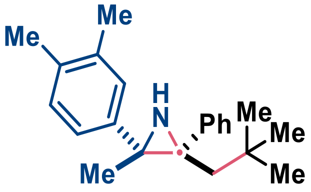

major diastereomer

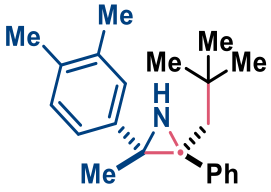

minor diastereomer

The title compound was prepared according to general procedure **GP3** from alkene **1a** (46.0 mg, 0.200 mmol, 1.0 equiv) and reagent **2u** (74.2 mg, 0.300 mmol, 1.5 equiv) with thioxanthone (2.2 mg, 10 µmol, 5 mol%) in ethyl acetate (1.0 mL, 0.20 M). Column chromatography (*n*-pentane/ethyl acetate = 100:1 to 50:1) gave the title compound as two separable diastereomers:

Major diastereomer: white solid (32.2 mg, 0.105 mmol, 52%)

^{1}H NMR (400 MHz, $CDCl_3$) δ 7.22 – 7.16 (m, 2H), 7.03 – 6.97 (m, 2H), 6.93 – 6.87 (m, 2H), 6.85 – 6.81 (m, 1H), 6.78 – 6.73 (m, 1H), 2.53 (d, *J* = 14.7 Hz, 1H), 2.05 (s, 3H), 2.03 (s, 3H), 1.79 (s, 3H), 1.75 (d, *J* = 14.8 Hz, 1H), 0.79 (s, 9H).

$^{13}C\{^1H\}$ NMR (101 MHz, $CDCl_3$) δ 141.4, 139.8, 135.3, 133.7, 128.8, 128.7, 128.7, 127.1, 125.7, 124.8, 51.1, 47.8, 46.8, 32.8, 30.9, 23.8, 19.7, 19.4.

HRMS (ESI): *m/z* calculated for $[C_{22}H_{29}NNa]^+$ $[M+Na]^+$: 330.2192; found: 330.2193.

Minor diastereomer: white solid (11.5 mg, 37.4 µmol, 19%)

^1H NMR (400 MHz, $CDCl_3$) δ 7.66 – 7.60 (m, 1H), 7.43 – 7.31 (m, 3H), 7.28 – 7.21 (m, 1H), 7.17 – 7.07 (m, 3H), 2.31 (s, 3H), 2.28 (s, 3H), 2.11 (d, *J* = 15.0 Hz, 1H), 1.04 (s, 3H), 0.80 (d, *J* = 15.1 Hz, 1H), 0.66 (s, 9H).

$^{13}C\{^1H\}$ NMR (101 MHz, $CDCl_3$) δ 141.4, 139.9, 136.9, 135.3, 129.9, 128.5, 128.4, 127.9, 126.6, 124.5, 51.2, 50.2, 47.7, 32.6, 30.7, 24.6, 20.1, 19.6.

HRMS (ESI): *m/z* calculated for $[C_{22}H_{29}NNa]^+$ $[M+Na]^+$: 330.2192; found: 330.2191.

Aziridine 3v

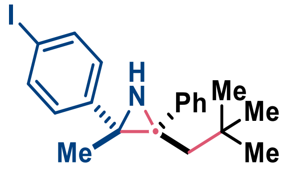

The title compound was prepared according to general procedure **GP3** from alkene **1a** (46.0 mg, 0.200 mmol, 1.0 equiv) and reagent **2v** (104 mg, 0.301 mmol, 1.5 equiv) with thioxanthone (2.2 mg, 10 µmol, 5 mol%) in ethyl acetate (1.0 mL, 0.20 M). Column chromatography (*n*-pentane/ethyl acetate = 200:1 to 25:1) gave the title compound as a white solid (51.2 mg, 0.126 mmol, 63%).

^1H NMR (500 MHz, $CDCl_3$) δ 7.34 – 7.29 (m, 2H), 7.16 (d, *J* = 7.5 Hz, 2H), 7.04 – 6.98 (m, 2H), 6.95 – 6.90 (m, 1H), 6.89 – 6.85 (m, 2H), 2.56 (d, *J* = 14.7 Hz, 1H), 1.78 (s, 3H), 1.69 (d, *J* = 14.7 Hz, 1H), 0.78 (s, 9H).

$^{13}C\{^1H\}$ NMR (126 MHz, $CDCl_3$) δ 142.6, 141.0, 136.4, 129.7, 128.6, 127.4, 126.0, 91.2, 51.0, 47.8, 46.2, 32.9, 30.9, 23.4.

HRMS (ESI): *m/z* calculated for $[C_{20}H_{24}NIH]^+$ $[M+H]^+$: 406.1026; found: 406.1027.

Note 1: The major diastereomer was isolated. Crude ^1H NMR analysis revealed a d.r. of 70:30.

Note 2: This structure was additionally confirmed by single crystal X-Ray analysis.

Aziridine 3w

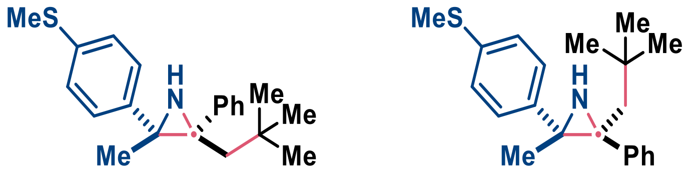

The title compound was prepared according to general procedure **GP3** from alkene **1a** (46.0 mg, 0.200 mmol, 1.0 equiv) and reagent **2w** (79.6 mg, 0.300 mmol, 1.5 equiv) with thioxanthone (2.2 mg, 10 µmol, 5 mol%) in ethyl acetate (1.0 mL, 0.20 M). Column chromatography (*n*-pentane/ethyl acetate = 100:1 to 15:1) gave the title compound as two separable diastereomers:

Major diastereomer: light-yellow solid (39.9 mg, 0.123 mmol, 61%)

^{1}H NMR (400 MHz, $CDCl_3$) δ 7.18 (d, J = 7.6 Hz, 2H), 7.08 – 7.03 (m, 2H), 7.02 – 6.97 (m, 2H), 6.93 – 6.86 (m, 3H), 2.54 (d, J = 14.9 Hz, 1H), 2.32 (s, 3H), 1.80 (s, 3H), 1.74 (d, J = 14.7 Hz, 1H), 0.78 (s, 9H).

^{13}C{^{1}H} NMR (101 MHz, $CDCl_3$) δ 140.8, 139.5, 135.3, 128.6, 128.0, 127.3, 125.9, 125.9, 51.5, 47.6, 46.8, 32.8, 30.8, 23.4, 16.0.

HRMS (ESI): *m/z* calculated for $[C_{21}H_{27}NSNa]^+$ $[M+Na]^+$: 348.1756; found: 348.1755.

Minor diastereomer: light-yellow solid (13.1 mg, 40.2 µmol, 20%)

^{1}H NMR (400 MHz, $CDCl_3$) δ 7.62 (s, 1H), 7.40 – 7.33 (m, 5H), 7.31 – 7.24 (m, 3H), 2.51 (s, 3H), 2.07 (d, J = 15.0 Hz, 1H), 1.07 (s, 3H), 0.81 (d, J = 15.0 Hz, 1H), 0.66 (s, 9H).

^{13}C{^{1}H} NMR (101 MHz, $CDCl_3$) δ 140.6, 138.9, 137.2, 128.4, 128.1, 127.8, 126.9, 126.8, 51.9, 50.0, 48.0, 32.5, 30.7, 24.4, 15.9.

HRMS (ESI): *m/z* calculated for $[C_{21}H_{27}NSNa]^+$ $[M+Na]^+$: 348.1756; found: 348.1756.

Aziridine 3x

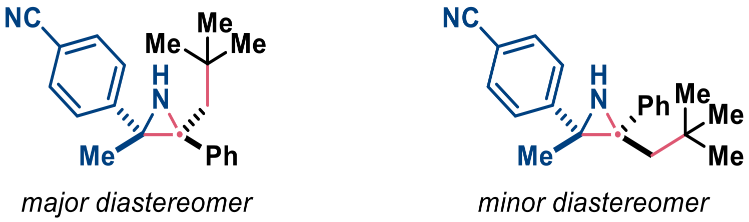

The title compound was prepared according to general procedure **GP3** from alkene **1a** (46.0 mg, 0.200 mmol, 1.0 equiv) and reagent **2x** (73.3 mg, 0.300 mmol, 1.5 equiv) with thioxanthone (2.2 mg, 10 µmol, 5 mol%) in ethyl acetate (1.0 mL, 0.20 M). Column chromatography (*n*-pentane/ethyl acetate = 40:1 to 9:1) gave the title compound as two separable diastereomers:

Major diastereomer: light-yellow solid (24.9 mg, 81.8 µmol, 41%)

^{1}H NMR (400 MHz, $CDCl_3$) δ 7.71 – 7.66 (m, 2H), 7.56 (d, J = 7.9 Hz, 2H), 7.52 – 7.33 (m, 4H), 7.31 – 7.26 (m, 1H), 1.97 (d, J = 14.7 Hz, 1H), 1.08 (s, 3H), 0.69 – 0.61 (m, 10H).

^{13}C{^{1}H} NMR (101 MHz, $CDCl_3$) δ 148.1, 140.7, 132.5, 128.6, 128.4, 127.8, 127.2, 118.9, 110.9, 51.7, 49.8, 47.3, 32.5, 30.6, 24.1.

HRMS (ESI): *m/z* calculated for $[C_{21}H_{24}N_2Na]^+$ $[M+Na]^+$: 327.1832; found: 327.1831.

Note: This structure was additionally confirmed by single crystal X-Ray analysis.

Minor diastereomer: white solid (20.5 mg, 67.3 µmol, 34%)

^{1}H NMR (400 MHz, $CDCl_3$) δ 7.30 – 7.22 (m, 4H), 7.14 (d, J = 7.6 Hz, 2H), 7.01 – 6.95 (m, 2H), 6.93 – 6.87 (m, 1H), 2.59 (d, J = 14.7 Hz, 1H), 1.83 (s, 3H), 1.70 (d, J = 14.7 Hz, 1H), 0.79 (s, 9H).

^{13}C{^{1}H} NMR (101 MHz, $CDCl_3$) δ 148.1, 140.2, 131.3, 128.5, 128.4, 127.5, 126.3, 119.2, 109.5, 51.7, 47.6, 46.5, 32.9, 30.8, 22.7.

HRMS (ESI): *m/z* calculated for $[C_{21}H_{24}N_2Na]^+$ $[M+Na]^+$: 327.1832; found: 327.1830.

Aziridine 3y

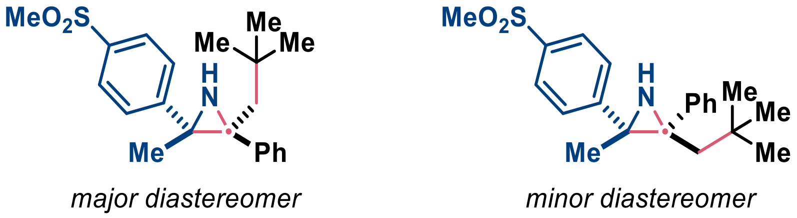

The title compound was prepared according to general procedure **GP3** from alkene **1a** (46.0 mg, 0.200 mmol, 1.0 equiv) and reagent **2y** (89.2 mg, 0.300 mmol, 1.5 equiv) with thioxanthone (2.2 mg, 10 µmol, 5 mol%) in ethyl acetate (1.0 mL, 0.20 M). Column chromatography (*n*-pentane/ethyl acetate = 20:1 to 2:1) gave the title compound as two separable diastereomers.

Major diastereomer: white solid (27.6 mg, 77.2 µmol, 39%)

^{1}H NMR (400 MHz, $CDCl_3$) δ 8.01 – 7.93 (m, 2H), 7.68 (d, J = 8.0 Hz, 2H), 7.58 – 7.27 (m, 5H), 3.10 (s, 3H), 2.00 (d, J = 14.7 Hz, 1H), 1.12 (s, 3H), 0.72 – 0.63 (m, 10H).

^{13}C{^{1}H} NMR (101 MHz, $CDCl_3$) δ 148.5, 140.1, 139.3, 128.7, 128.6, 128.4, 127.8, 127.3, 52.2, 49.7, 47.6, 44.6, 32.5, 30.6, 24.2.

HRMS (ESI): *m/z* calculated for $[C_{21}H_{27}NO_2SNa]^+$ $[M+Na]^+$: 380.1655; found: 380.1655.

Minor diastereomer: white solid (19.9 mg, 55.7 µmol, 28%)

^{1}H NMR (400 MHz, $CDCl_3$) δ 7.57 (d, J = 8.1 Hz, 2H), 7.38 (d, J = 8.1 Hz, 2H), 7.19 (d, J = 7.6 Hz, 2H), 6.99 (t, J = 7.6 Hz, 2H), 6.89 (t, J = 7.3 Hz, 1H), 2.86 (s, 3H), 2.58 (d, J = 14.7 Hz, 1H), 1.90 (s, 3H), 1.80 (d, J = 14.2 Hz, 1H), 0.80 (s, 9H).

^{13}C{^{1}H} NMR (101 MHz, $CDCl_3$) δ 148.5, 139.4, 138.0, 128.6, 128.4, 127.7, 126.6, 126.5, 52.5, 47.1, 46.9, 44.6, 32.8, 30.8, 22.4.

HRMS (ESI): *m/z* calculated for $[C_{21}H_{27}NO_2SNa]^+$ $[M+Na]^+$: 380.1655; found: 380.1654.

Aziridine 3z

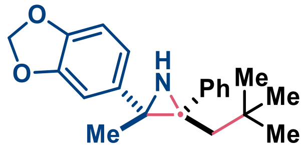

The title compound was prepared according to general procedure **GP3** from alkene **1a** (46.0 mg, 0.200 mmol, 1.0 equiv) and reagent **2z** (79.0 mg, 0.300 mmol, 1.5 equiv) with thioxanthone (2.2 mg, 10 µmol, 5 mol%) in ethyl acetate (1.0 mL, 0.20 M). Column chromatography (*n*-

pentane/ethyl acetate = 200:1 to 25:1) gave the title compound as a colorless oil (39.5 mg, 0.122 mmol, 61%).

^{1}H NMR (500 MHz, $CDCl_3$) δ 7.19 (d, J = 7.6 Hz, 2H), 7.03 (t, J = 7.5 Hz, 2H), 6.93 (t, J = 7.3 Hz, 1H), 6.63 (dd, J = 15.9, 4.7 Hz, 2H), 6.46 (d, J = 8.1 Hz, 1H), 5.76 (s, 2H), 2.54 (d, J = 14.7 Hz, 1H), 1.78 (s, 3H), 1.74 – 1.68 (m, 1H), 0.78 (s, 9H).

^{13}C{^{1}H} NMR (126 MHz, $CDCl_3$) δ 146.7, 145.2, 141.6, 137.1, 128.7, 127.2, 125.7, 120.8, 108.4, 107.3, 100.6, 50.8, 47.9, 46.3, 32.9, 30.9, 24.0.

HRMS (ESI): m/z calculated for $[C_{21}H_{25}NO_2Na]^+$ $[M+Na]^+$: 346.1778; found: 346.1777.

Note: The major diastereomer was isolated. Crude ^{1}H NMR analysis revealed a d.r. of 75:25.

Aziridine 3aa

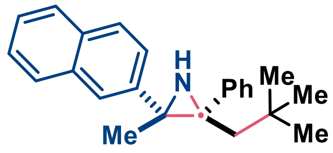

The title compound was prepared according to general procedure **GP3** from alkene **1a** (46.0 mg, 0.200 mmol, 1.0 equiv) and reagent **2aa** (80.8 mg, 0.300 mmol, 1.5 equiv) with thioxanthone (2.2 mg, 10 μmol, 5 mol%) in ethyl acetate (1.0 mL, 0.20 M). Column chromatography (n-pentane/ethyl acetate = 75:1 to 50:1) gave the title compound as a colorless sticky oil (46.0 mg, 0.140 mmol, 70%).

^{1}H NMR (400 MHz, $CDCl_3$) δ 7.71 – 7.59 (m, 3H), 7.49 (d, J = 8.5 Hz, 1H), 7.39 – 7.29 (m, 2H), 7.29 – 7.21 (m, 3H), 6.97 – 6.88 (m, 2H), 6.83 – 6.74 (m, 1H), 2.64 (d, J = 14.7 Hz, 1H), 1.89 (s, 3H), 1.79 (d, J = 14.7 Hz, 1H), 0.83 (s, 9H).

^{13}C{^{1}H} NMR (101 MHz, $CDCl_3$) δ 141.3, 140.3, 133.0, 131.9, 128.6, 127.8, 127.5, 127.2, 126.9, 126.2, 126.1, 125.8, 125.6, 125.2, 51.2, 47.9, 47.0, 32.9, 30.9, 23.7.

HRMS (ESI): m/z calculated for $[C_{24}H_{27}NNa]^+$ $[M+Na]^+$: 352.2036; found: 352.2035.

Note: The major diastereomer was isolated. Crude ^{1}H NMR analysis revealed a d.r. of 74:26.

Aziridine 3ab

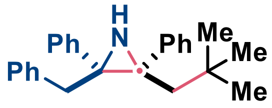

The title compound was prepared according to general procedure **GP3** from alkene **1a** (46.0 mg, 0.200 mmol, 1.0 equiv) and reagent **2ab** (88.6 mg, 0.300 mmol, 1.5 equiv) with thioxanthone (2.2 mg, 10 µmol, 5 mol%) in ethyl acetate (1.0 mL, 0.20 M). Column chromatography (*n*-pentane/ethyl acetate = 200:1 to 75:1) gave the title compound as a white solid (51.5 mg, 0.145 mmol, 72%).

^{1}H NMR (400 MHz, $CDCl_3$) δ 7.23 – 7.12 (m, 5H), 7.07 – 7.02 (m, 2H), 7.02 – 6.95 (m, 4H), 6.93 – 6.81 (m, 4H), 3.65 (d, *J* = 14.6 Hz, 1H), 3.32 (d, *J* = 14.7 Hz, 1H), 2.73 (d, *J* = 14.5 Hz, 1H), 1.95 (d, *J* = 14.6 Hz, 1H), 0.84 (s, 9H).

^{13}C{^{1}H} NMR (101 MHz, $CDCl_3$) δ 141.4, 140.7, 138.3, 129.6, 128.9, 128.5, 128.3, 127.2, 127.0, 126.4, 125.8, 125.7, 51.4, 51.3, 47.9, 41.8, 33.1, 31.0.

HRMS (ESI): *m/z* calculated for $[C_{26}H_{29}NNa]^+$ $[M+Na]^+$: 378.2192; found: 378.2184.

Note 1: The major diastereomer was isolated. Crude ^{1}H NMR analysis revealed a d.r. of 89:11.

Note 2: This structure was additionally confirmed by single crystal X-Ray analysis.

Aziridine 3ac

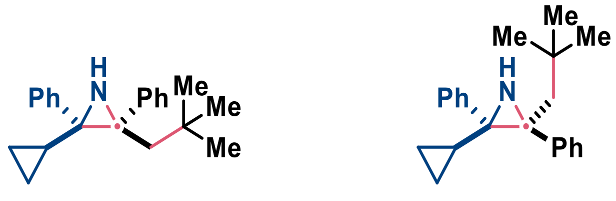

The title compound was prepared according to general procedure **GP3** from alkene **1a** (46.0 mg, 0.200 mmol, 1.0 equiv) and reagent **2ac** (73.6 mg, 0.300 mmol, 1.5 equiv) with thioxanthone (2.2 mg, 10 µmol, 5 mol%) in ethyl acetate (1.0 mL, 0.20 M). Column chromatography (*n*-pentane/ethyl acetate = 150:1 to 100:1) gave the title compound as two separable diastereomers:

Major diastereomer: white solid (26.9 mg, 88.1 µmol, 44%)

^{1}H NMR (400 MHz, $CDCl_3$) δ 7.22 – 7.17 (m, 2H), 7.11 – 7.04 (m, 2H), 7.03 – 6.94 (m, 4H), 6.94 – 6.85 (m, 2H), 2.59 (d, *J* = 14.7 Hz, 1H), 2.06 (d, *J* = 14.7 Hz, 1H), 1.83 – 1.71 (m, 1H), 0.81 (s, 9H), 0.77 – 0.68 (m, 1H), 0.66 – 0.54 (m, 1H), 0.49 – 0.38 (m, 1H), 0.25 – 0.14 (m, 1H).

$^{13}C\{^{1}H\}$ NMR (101 MHz, $CDCl_3$) δ 141.6, 141.1, 128.5, 128.3, 127.2, 127.1, 125.7, 125.7, 51.7, 50.8, 46.9, 32.9, 31.0, 16.5, 6.6, 5.1.

HRMS (ESI): *m/z* calculated for $[C_{22}H_{27}NNa]^+$ $[M+Na]^+$: 328.2036; found: 328.2034.

Minor diastereomer: white solid (23.7 mg, 77.6 µmol, 39%)

^{1}H NMR (400 MHz, $CDCl_3$) δ 7.63 – 7.46 (m, 2H), 7.40 – 7.27 (m, 7H), 7.25 – 7.19 (m, 1H), 2.16 (d, *J* = 14.8 Hz, 1H), 0.68 – 0.60 (m, 10H), 0.58 – 0.50 (m, 1H), 0.15 – -0.13 (m, 4H).

$^{13}C\{^{1}H\}$ NMR (101 MHz, $CDCl_3$) δ 141.8, 140.6, 128.9, 128.4, 128.3, 127.9, 127.0, 126.3, 51.7, 50.8, 50.7, 32.6, 30.7, 16.6, 3.8, 2.3.

HRMS (ESI): *m/z* calculated for $[C_{22}H_{27}NNa]^+$ $[M+Na]^+$: 328.2036; found: 328.2034.

Note: The minor diastereomer's structure was additionally confirmed by single crystal X-Ray analysis.

Aziridine 3ad

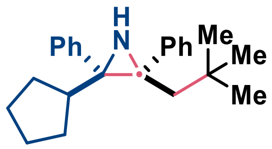

The title compound was prepared according to general procedure **GP3** from alkene **1a** (46.0 mg, 0.200 mmol, 1.0 equiv) and reagent **2ad** (82.0 mg, 0.300 mmol, 1.5 equiv) with thioxanthone (2.2 mg, 10 µmol, 5 mol%) in ethyl acetate (1.0 mL, 0.20 M). Column chromatography (*n*-pentane/ethyl acetate = 200:1 to 100:1) gave the title compound as a white solid (52.1 mg, 0.156 mmol, 78%).

^{1}H NMR (400 MHz, $CDCl_3$) δ 7.44 – 6.72 (m, 10H), 2.69 (d, *J* = 14.7 Hz, 1H), 2.16 – 1.93 (m, 2H), 1.87 – 1.70 (m, 2H), 1.60 – 1.27 (m, 5H), 1.09 – 0.98 (m, 1H), 0.80 (s, 9H).

$^{13}C\{^{1}H\}$ NMR (101 MHz, $CDCl_3$) δ 142.1, 138.1, 130.9, 128.1, 127.2, 126.1, 125.7, 125.6, 53.1, 50.7, 47.7, 45.8, 33.1, 31.2, 29.8, 29.4, 24.6, 24.4.

HRMS (ESI): *m/z* calculated for $[C_{24}H_{31}NNa]^+$ $[M+Na]^+$: 356.2349; found: 356.2350.

Note: The major diastereomer was isolated. Crude ^{1}H NMR analysis revealed a d.r. of 89:11.

Aziridine 3ae

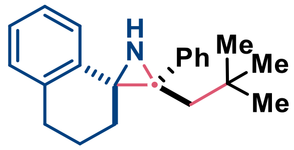

The title compound was prepared according to general procedure **GP3** from alkene **1a** (92.0 mg, 0.400 mmol, 1.0 equiv) and reagent **2ae** (147 mg, 0.600 mmol, 1.5 equiv) with thioxanthone (4.2 mg, 20 µmol, 5 mol%) in ethyl acetate (2.0 mL, 0.20 M). Column chromatography (*n*-pentane/ethyl acetate = 400:1 to 50:1) gave the title compound as a white solid (69.5 mg, 0.228 mmol, 57%).

^{1}H NMR (400 MHz, $CDCl_3$) δ 7.59 – 6.40 (m, 9H), 2.99 – 2.82 (m, 2H), 2.43 (d, *J* = 14.5 Hz, 1H), 2.20 – 2.01 (m, 4H), 1.73 (d, *J* = 14.5 Hz, 1H), 0.81 (s, 9H).

^{13}C{^{1}H} NMR (101 MHz, $CDCl_3$) δ 141.0, 137.8, 137.6, 129.3, 127.4, 127.2, 126.1, 125.8, 125.8, 124.2, 52.6, 48.5, 46.4, 32.8, 30.8, 30.0, 29.1, 22.6.

HRMS (ESI): *m/z* calculated for $[C_{22}H_{27}NNa]^+$ $[M+Na]^+$: 328.2036; found: 328.2036.

Note 1: The major diastereomer was isolated. Crude ^{1}H NMR analysis revealed a d.r. of 63:37.

Note 2: This structure was additionally confirmed by single crystal X-Ray analysis

Aziridine 3af

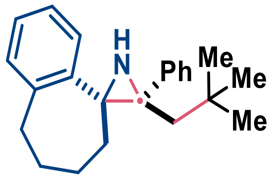

The title compound was prepared according to general procedure **GP3** from alkene **1a** (46.0 mg, 0.200 mmol, 1.0 equiv) and reagent **2af** (77.8 mg, 0.300 mmol, 1.5 equiv) with thioxanthone (2.2 mg, 10 µmol, 5 mol%) in ethyl acetate (1.0 mL, 0.20 M). Column chromatography (*n*-pentane/ethyl acetate = 400:1) followed by preparative TLC (*n*-pentane/ethyl acetate = 10:1) gave the title compound as a colorless oil (43.0 mg, 0.135 mmol, 67%).

^{1}H NMR (400 MHz, $CDCl_3$) δ 7.43 (d, *J* = 7.6 Hz, 1H), 7.31 (d, *J* = 7.6 Hz, 2H), 6.97 (t, *J* = 7.5 Hz, 2H), 6.94 – 6.80 (m, 3H), 6.72 (d, *J* = 7.5 Hz, 1H), 2.89 – 2.73 (m, 2H), 2.61 (dd, *J* = 13.9, 6.1 Hz, 1H), 2.26 – 2.13 (m, 1H), 2.10 – 1.84 (m, 4H), 1.75 (d, *J* = 14.6 Hz, 1H), 1.40 – 1.24 (m, 1H), 0.82 (s, 9H).

$^{13}C\{^1H\}$ NMR (101 MHz, $CDCl_3$) δ 141.0, 140.7, 140.5, 128.4, 128.3, 127.8, 126.9, 126.4, 125.7, 125.0, 50.7, 50.0, 47.3, 37.4, 36.8, 33.3, 31.2, 29.0, 27.3.

HRMS (ESI): *m/z* calculated for $[C_{23}H_{29}NNa]^+$ $[M+Na]^+$: 342.2192; found: 342.2192.

Note 1: The major diastereomer was isolated. Crude ^1H NMR analysis revealed a d.r. of 86:14.

Aziridine 3ag

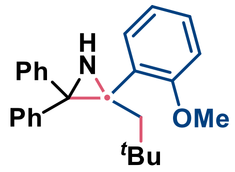

The title compound was prepared according to general procedure **GP3** from alkene **1c** (52.0 mg, 0.200 mmol, 1.0 equiv) and reagent **2ag** (84.4 mg, 0.300 mmol, 1.5 equiv) with thioxanthone (2.1 mg, 9.9 µmol, 5 mol%) in ethyl acetate (1.0 mL, 0.20 M). Column chromatography (*n*-pentane/diethyl ether = 250:1) gave the title compound as a white solid (33.0 mg, 88.8 µmol, 44%).

^1H NMR (599 MHz, $CDCl_3$) δ 7.64 – 7.59 (m, 2H), 7.49 – 7.42 (m, 1H), 7.41 – 7.33 (m, 2H), 7.31 – 7.25 (m, 3H), 6.99 (ddd, *J* = 8.1, 7.3, 1.8 Hz, 1H), 6.95 – 6.88 (m, 2H), 6.88 – 6.82 (m, 1H), 6.73 (td, *J* = 7.4, 1.1 Hz, 1H), 6.54 (dd, *J* = 8.2, 1.0 Hz, 1H), 3.87 (s, 3H), 2.84 (d, *J* = 14.7 Hz, 1H), 0.79 (d, *J* = 14.7 Hz, 1H), 0.69 (s, 9H).

$^{13}C\{^1H\}$ NMR (151 MHz, $CDCl_3$) δ 157.4, 142.9, 141.0, 130.6, 129.2, 128.9, 128.5, 127.9, 127.7, 127.0, 126.9, 125.7, 119.7, 109.6, 55.0, 52.9, 50.2, 50.0, 32.6, 30.4.

HRMS (ESI): *m/z* calculated for $[C_{26}H_{29}NONa]^+$ $[M+Na]^+$: 394.2141; found: 394.2142.

Aziridine 3ah

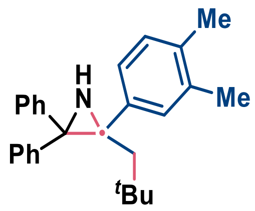

The title compound was prepared according to general procedure **GP3** from alkene **1d** (51.6 mg, 0.200 mmol, 1.0 equiv) and reagent **2ag** (84.4 mg, 0.300 mmol, 1.5 equiv) with thioxanthone (2.1 mg, 9.9 µmol, 5 mol%) in ethyl acetate (1.0 mL, 0.20 M). Column chromatography (*n*-

pentane/diethyl ether = 250:1) gave the title compound as a white solid (51.0 mg, 0.138 mmol, 69%).

^{1}H NMR (599 MHz, $CDCl_3$) δ 7.54 – 7.50 (m, 2H), 7.37 – 7.32 (m, 2H), 7.25 – 7.21 (m, 3H), 7.07 – 7.05 (m, 1H), 7.02 (d, J = 8.0 Hz, 1H), 6.97 – 6.92 (m, 2H), 6.88 – 6.84 (m, 1H), 6.82 (d, J = 7.7 Hz, 1H), 2.53 (d, J = 14.8 Hz, 1H), 2.11 (s, 3H), 2.08 (s, 3H), 0.98 (d, J = 14.7 Hz, 1H), 0.69 (s, 9H).

^{13}C{^{1}H} NMR (101 MHz, $CDCl_3$) δ 143.0, 141.4, 137.7, 135.2, 133.9, 129.8, 128.7, 128.7, 128.7, 128.5, 127.3, 127.0, 125.9, 125.8, 54.2, 51.5, 50.7, 32.9, 31.0, 19.8, 19.5.

HRMS (ESI): m/z calculated for $[C_{27}H_{31}NNa]^+$ $[M+Na]^+$: 392.2349; found: 392.2348.

Aziridine 3ai

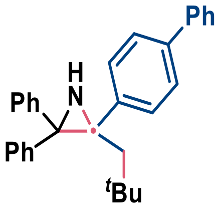

The title compound was prepared according to general procedure **GP3** from alkene **1e** (61.2 mg, 0.200 mmol, 1.0 equiv) and reagent **2ag** (84.4 mg, 0.300 mmol, 1.5 equiv) with thioxanthone (2.1 mg, 9.9 µmol, 5 mol%) in ethyl acetate (1.0 mL, 0.20 M). Column chromatography (*n*-pentane/diethyl ether = 50:1) and subsequent preparative TLC (*n*-pentane/ethyl acetate = 95:5) gave the title compound as a white solid (43.0 mg, 0.103 mmol, 51%).

^{1}H NMR (400 MHz, $CDCl_3$) δ 7.59 – 7.48 (m, 4H), 7.44 – 7.33 (m, 8H), 7.30 – 7.24 (m, 4H), 6.99 – 6.92 (m, 2H), 6.89 – 6.79 (m, 1H), 2.62 (d, J = 14.8 Hz, 1H), 1.07 (d, J = 14.7 Hz, 1H), 0.73 (s, 9H).

^{13}C{^{1}H} NMR (101 MHz, $CDCl_3$) δ 142.5, 140.9, 140.9, 139.6, 138.5, 129.0, 128.8, 128.7, 128.6, 128.5, 127.5, 127.2, 127.1, 126.9, 126.0, 126.0, 54.6, 51.7, 50.6, 33.0, 31.0.

HRMS (ESI): m/z calculated for $[C_{31}H_{31}NNa]^+$ $[M+Na]^+$: 440.2349; found: 440.2349.

Aziridine 3aj

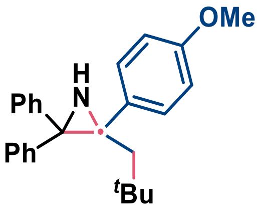

The title compound was prepared according to general procedure **GP3** from alkene **1f** (52.0 mg, 0.200 mmol, 1.0 equiv) and reagent **2ag** (84.4 mg, 0.300 mmol, 1.5 equiv) with thioxanthone (2.1 mg, 9.9 µmol, 5 mol%) in ethyl acetate (1.0 mL, 0.20 M). Column chromatography (*n*-pentane/ethyl acetate = 50:1) gave the title compound as a yellow solid (42.0 mg, 0.113 mmol, 57%).

^{1}H NMR (599 MHz, $CDCl_3$) δ 7.55 – 7.50 (m, 2H), 7.35 (t, *J* = 7.8 Hz, 2H), 7.29 – 7.22 (m, 5H), 6.96 (dd, *J* = 8.4, 7.0 Hz, 2H), 6.90 – 6.83 (m, 1H), 6.66 – 6.61 (m, 2H), 3.68 (s, 3H), 2.53 (d, *J* = 14.8 Hz, 1H), 1.01 (d, *J* = 14.8 Hz, 1H), 0.70 (s, 9H).

^{13}C{^{1}H} NMR (151 MHz, $CDCl_3$) δ 157.8, 142.9, 141.3, 132.7, 129.6, 128.7, 128.6, 128.5, 127.4, 127.0, 125.8, 112.9, 55.1, 54.3, 51.2, 50.9, 32.9, 30.9.

HRMS (ESI): *m/z* calculated for $[C_{26}H_{29}NONa]^+$ $[M+Na]^+$: 394.2141; found: 394.2142.

Aziridine 3ak

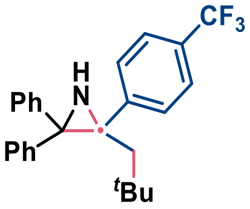

The title compound was prepared according to general procedure **GP3** from alkene **1g** (59.6 mg, 0.200 mmol, 1.0 equiv) and reagent **2ag** (84.4 mg, 0.300 mmol, 1.5 equiv) with thioxanthone (2.1 mg, 9.9 µmol, 5 mol%) in ethyl acetate (1.0 mL, 0.20 M). Column chromatography (*n*-pentane/diethyl ether = 500:1) gave the title compound as a white solid (41.0 mg, 0.100 mmol, 50%).

^{1}H NMR (500 MHz, $CDCl_3$) δ 7.55 – 7.45 (m, 4H), 7.41 – 7.25 (m, 5H), 7.24 – 7.18 (m, 2H), 6.98 – 6.91 (m, 2H), 6.89 – 6.83 (m, 1H), 2.64 (d, *J* = 14.9 Hz, 1H), 1.07 (d, *J* = 14.8 Hz, 1H), 0.69 (s, 9H).

^{13}C{^{1}H} NMR (126 MHz, $CDCl_3$) δ 145.1, 142.3, 140.4, 128.9, 128.9, 128.4, 128.4, 128.2 (q, *J* = 32.3 Hz), 127.6, 127.4, 126.2, 124.4, 124.4 (q, *J* = 271.9 Hz), 54.6, 51.2, 50.6, 33.1, 31.0.

$^{19}F\{^{1}H\}$ **NMR** (470 MHz, $CDCl_3$) δ -62.3.

HRMS (ESI): *m/z* calculated for $[C_{26}H_{26}NF_3H:]^+$ $[M+H]^+$: 410.2090; found: 410.2091.

Aziridine 3al

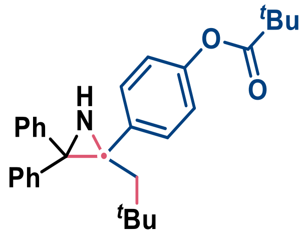

The title compound was prepared according to general procedure **GP3** from alkene **1h** (66.1 mg, 0.200 mmol, 1.0 equiv) and reagent **2ag** (84.4 mg, 0.300 mmol, 1.5 equiv) with thioxanthone (2.1 mg, 9.9 µmol, 5 mol%) in ethyl acetate (1.0 mL, 0.20 M). Column chromatography (*n*-pentane/ethyl acetate = 100:1) gave the title compound as a white solid (65.0 mg, 0.147 mmol, 74%).

^{1}H **NMR** (500 MHz, $CDCl_3$) δ 7.55 – 7.51 (m, 2H), 7.39 – 7.34 (m, 4H), 7.28 – 7.24 (m, 3H), 7.03 – 6.94 (m, 2H), 6.93 – 6.86 (m, 1H), 6.80 (d, *J* = 8.7 Hz, 2H), 2.59 (d, *J* = 14.8 Hz, 1H), 1.30 (s, 9H), 1.04 (d, *J* = 14.8 Hz, 1H), 0.71 (s, 9H).

$^{13}C\{^{1}H\}$ **NMR** (126 MHz, $CDCl_3$) δ 177.0, 149.3, 142.7, 140.9, 138.0, 129.4, 128.8, 128.5, 128.5, 127.5, 127.2, 126.0, 120.5, 54.4, 51.2, 50.9, 39.1, 33.0, 31.0, 27.2.

HRMS (ESI): *m/z* calculated for $[C_{30}H_{35}NO_2Na]^+$ $[M+Na]^+$: 464.2560; found: 464.2563.

Aziridine 3am

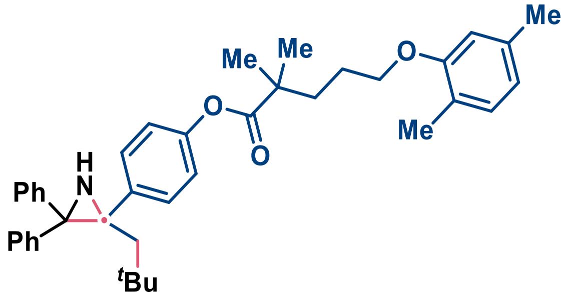

The title compound was prepared according to general procedure **GP3** from alkene **1i** (95.7 mg, 0.200 mmol, 1.0 equiv) and reagent **2ag** (84.4 mg, 0.300 mmol, 1.5 equiv) with thioxanthone (2.1 mg, 9.9 µmol, 5 mol%) in ethyl acetate (1.0 mL, 0.20 M). Column chromatography (*n*-

pentane/ethyl acetate = 50:1) gave the title compound as a colorless oil (63.0 mg, 0.107 mmol, 53%).

^{1}H NMR (599 MHz, $CDCl_3$) δ 7.55 – 7.49 (m, 2H), 7.39 – 7.33 (m, 4H), 7.28 – 7.24 (m, 3H), 7.03 – 6.95 (m, 3H), 6.91 – 6.88 (m, 1H), 6.77 (d, J = 8.4 Hz, 2H), 6.70 – 6.65 (m, 1H), 6.64 – 6.60 (m, 1H), 3.98 – 3.93 (m, 2H), 2.58 (d, J = 14.9 Hz, 1H), 2.31 (s, 3H), 2.15 (s, 3H), 1.86 – 1.81 (m, 4H), 1.31 (s, 6H), 1.04 (d, J = 14.9 Hz, 1H), 0.71 (s, 9H).

^{13}C{^{1}H} NMR (151 MHz, $CDCl_3$) δ 176.3, 157.0, 149.2, 142.7, 140.9, 138.1, 136.6, 130.5, 129.5, 128.8, 128.5, 128.5, 127.6, 127.2, 126.0, 123.8, 120.9, 120.5, 112.1, 67.9, 54.4, 51.2, 50.9, 42.5, 37.2, 33.0, 31.0, 25.4, 25.3, 21.6, 15.9.

HRMS (ESI): *m/z* calculated for $[C_{40}H_{47}NO_3Na]^+$ $[M+Na]^+$: 612.3448; found: 612.3440.

Aziridine 3an

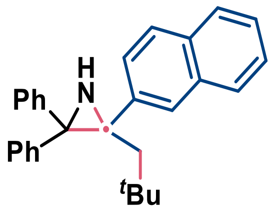

The title compound was prepared according to general procedure **GP3** from alkene **1j** (56.0 mg, 0.200 mmol, 1.0 equiv) and reagent **2ag** (84.4 mg, 0.300 mmol, 1.5 equiv) with thioxanthone (2.1 mg, 9.9 µmol, 5 mol%) in ethyl acetate (1.0 mL, 0.20 M). Column chromatography (*n*-pentane/diethyl ether = 250:1) gave the title compound as a white solid (41.0 mg, 0.105 mmol, 52%).

^{1}H NMR (500 MHz, $CDCl_3$) δ 7.81 – 7.77 (m, 1H), 7.73 – 7.64 (m, 2H), 7.61 – 7.56 (m, 3H), 7.53 (dd, J = 8.5, 1.7 Hz, 1H), 7.41 – 7.31 (m, 4H), 7.30 – 7.26 (m, 3H), 6.90 – 6.82 (m, 2H), 6.76 – 6.71 (m, 1H), 2.71 (d, J = 14.9 Hz, 1H), 1.10 (d, J = 14.9 Hz, 1H), 0.69 (s, 9H).

^{13}C{^{1}H} NMR (101 MHz, $CDCl_3$) δ 142.8, 140.9, 138.3, 133.1, 132.1, 128.8, 128.5, 128.5, 127.9, 127.6, 127.4, 127.3, 127.2, 127.0, 126.9, 125.9, 125.6, 125.3, 54.7, 51.8, 50.8, 33.0, 31.0.

HRMS (ESI): *m/z* calculated for $[C_{29}H_{29}NNa]^+$ $[M+Na]^+$: 414.2192; found: 414.2191.

Aziridine 3ao

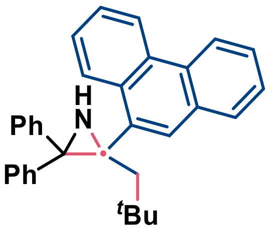

The title compound was prepared according to general procedure **GP3** from alkene **1k** (66.0 mg, 0.200 mmol, 1.0 equiv) and reagent **2ag** (84.4 mg, 0.300 mmol, 1.5 equiv) with thioxanthone (2.1 mg, 10 µmol, 5 mol%) in ethyl acetate (1.0 mL, 0.20 M). Column chromatography (*n*-pentane/diethyl ether = 100:1 to 20:1) gave the title compound as a white solid (30.0 mg, 67.9 µmol, 34%).

^{1}H NMR (500 MHz, $CDCl_3$) δ 8.60 – 8.50 (m, 3H), 8.12 (s, 1H), 7.90 – 7.86 (m, 1H), 7.79 – 7.75 (m, 2H), 7.73 – 7.68 (m, 1H), 7.61 – 7.52 (m, 3H), 7.48 (t, *J* = 7.7 Hz, 2H), 7.38 – 7.28 (m, 3H), 6.73 – 6.67 (m, 2H), 6.67 – 6.61 (m, 1H), 2.94 (d, *J* = 14.9 Hz, 1H), 1.22 (d, *J* = 14.9 Hz, 1H), 0.59 (s, 9H).

^{13}C{^{1}H} NMR (101 MHz, $CDCl_3$) δ 142.8, 140.1, 134.6, 131.5, 130.7, 130.5, 129.9, 129.5, 129.1, 128.9, 128.8, 128.1, 127.4, 126.9, 126.5, 126.3, 126.0, 125.9, 125.9, 125.8, 123.3, 122.4, 53.8, 51.9, 51.0, 33.1, 30.1.

HRMS (ESI): *m/z* calculated for $[C_{33}H_{31}NNa]^+$ $[M+Na]^+$: 464.2349; found: 464.2349.

4.2 Limitations

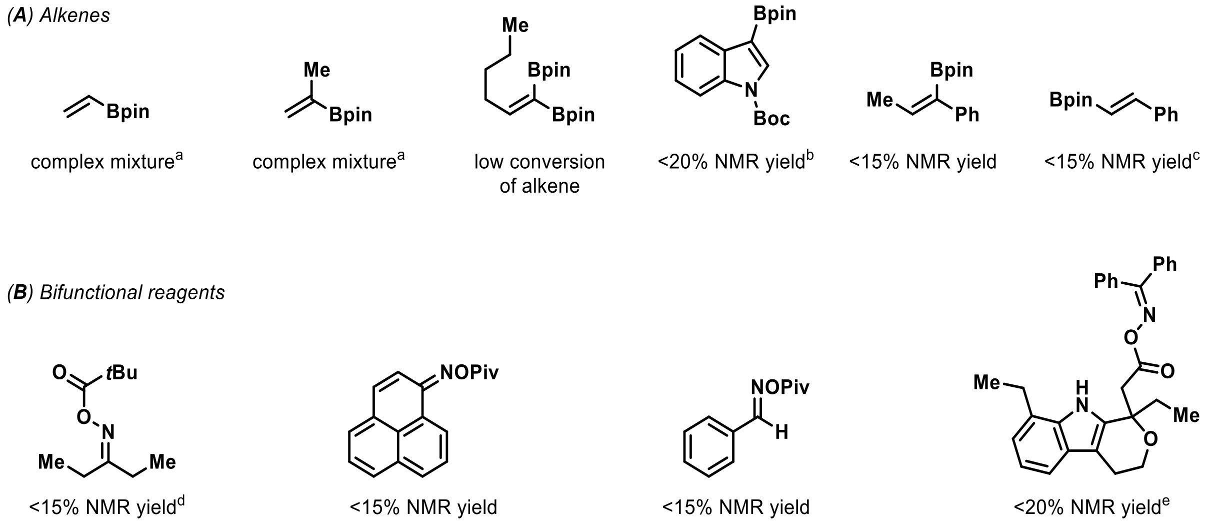

Supplementary Figure 5. Unsuccessful substrates. Reactions were performed on 0.1 or 0.2 mmol scale following the standard conditions with **2ag** as bifunctional reagent or **1a** as alkene. ^{1}H NMR yields were determined with mesitylene or CH_2Br_2 as internal standard. [a]Alkene (0.2 mmol, 2.0 equiv), **2i** (0.1 mmol, 1.0 equiv), 2-isopropylthioxanthone (5 mol%), EtOAc (50 mM), 380 nm LEDs (18 W), rt, 16 h. [b]Alkene (0.2 mmol, 1.0 equiv), **2ag** (0.4 mmol, 2.0 equiv), TXT (10 mol%), EtOAc (50 mM), 405 nm LEDs (18 W), rt, 16 h. [c]Reagent **2a** used. [d]0.3 mmol scale. [e]**1a** (0.2 mmol) and reagent (0.2 mmol) used.

5. Mechanistic experiments

5.1 Radical trapping studies

Trapping Experiment with BHT

An oven-dried 10 mL Schlenk tube was charged with a Teflon-coated stirring bar, alkene **1a** (23.0 mg, 0.100 mmol, 1.0 equiv), reagent **2a** (46.4 mg, 0.150 mmol, 1.5 equiv), thioxanthone (1.1 mg, 5.2 µmol, 5 mol%), and butylated hydroxytoluol (22.0 mg, 99.8 µmol, 1.0 equiv). The tube was evacuated and backfilled with argon three times. Dry ethyl acetate (0.50 mL, 0.20 M) was added and the mixture was then irradiated at 405 nm for 16 h. After that, the solvent was removed in vacuo and the residue was examined by ^{1}H NMR spectroscopy with mesitylene as internal standard.

The NMR yield of aziridine **3a** was determined to be below 10%. Crude ESI-HRMS was carried out:

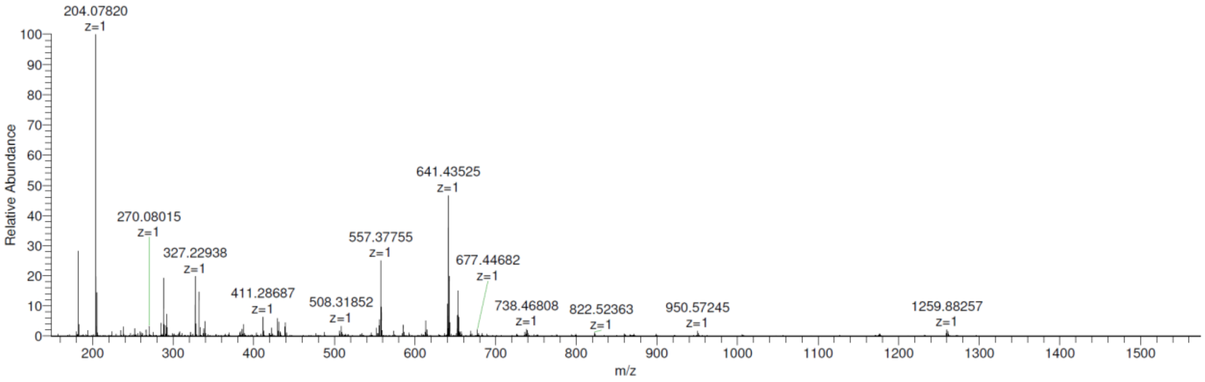

Supplementary Figure 6. Crude ESI-HRMS analysis of the BHT trapping experiment.

The *m/z*-ratios of the following structures were detected:

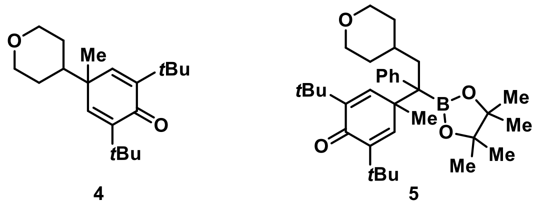

4
HRMS (ESI): m/z calculated for $[C_{20}H_{32}NaO_2]^+$ $[M+Na]^+$: 327.2295; found: 327.2294

5
HRMS (ESI): m/z calculated for $[C_{34}H_{51}BNaO_4]^+$ $[M+Na]^+$: 557.3773; found: 557.3776

Note: Given structures are reasonable structures based on the found m/z-ratio.

Trapping Experiment with TEMPO

An oven-dried 10 mL Schlenk tube was charged with a Teflon-coated stirring bar, alkene **1a** (23.0 mg, 0.100 mmol, 1.0 equiv), reagent **2a** (46.4 mg, 0.150 mmol, 1.5 equiv), thioxanthone (1.1 mg, 5.2 µmol, 5 mol%), and (2,2,6,6-tetramethylpiperidin-1-yl)oxyl (15.6 mg, 99.8 µmol, 1.0 equiv). The tube was evacuated and backfilled with argon three times. Dry ethyl acetate (0.50 mL, 0.20 M) was added and the mixture was then irradiated at 405 nm for 16 h. After that,

the solvent was removed in vacuo and the residue was examined by ^1H NMR spectroscopy with mesitylene as internal standard.
The NMR yield of aziridine **3a** was determined to be 26% and no TEMPO adducts were detected by HRMS analysis.

5.2 Reaction quantum yield

Measurement of the photon flux

The photon flux was determined by ferrioxalate actinometry similar to a procedure by Yoon.[15] A 3 W LED (λ_{max} = 397 nm) was used for the quantum yield measurement.

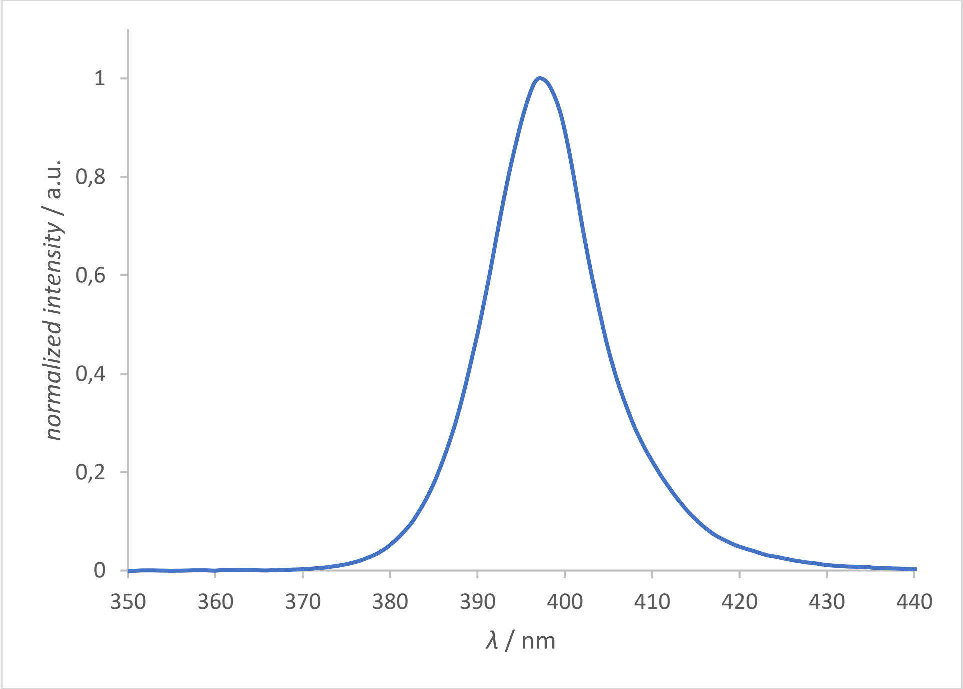

Supplementary Figure 7. Emission spectrum of the used 3 W LED (λ_{max} = 397 nm).

A solution (10 mL, 0.15 M) of potassium ferrioxalate hydrate (737 mg, 1.50 mmol) in aq. H_2SO_4 (50 mM) and a solution (20 mL, 5.0 mM) of 1,10-phenanthroline monohydrate (20 mg, 0.10 mmol) and sodium acetate (4.50 g) in aq. H_2SO_4 (500 mM) were prepared and kept in the dark. All following steps were conducted in the dark as well.

Four Schlenk tubes were charged with the prepared ferrioxalate solution (1.0 mL) and two of these tubes were successively irradiated with the described LED for 60 s at a distance of 5 cm, while two tubes were left in the dark. Then, to all four Schlenk tubes was added the prepared phenanthroline solution (175 μL each) and the reaction mixtures were stirred for 60 mins. For all

four solutions, the absorbance at 510 nm was measured and the difference between the average absorbance of the two irradiated samples and the average absorbance of the two control samples was determined ($\Delta A_{510\,\mathrm{nm}}$ = 2.189).

The formed amount of Fe(II) was calculated based on the Lambert–Beer law (equation 1) with V = 1.175 mL, l = 1.0 cm, and ε = 11100 L·mol^{-1}·cm^{-1}.[16,17]

$$n_{Fe(II)} = \frac{V \cdot \Delta A_{510\,\mathrm{nm}}}{l \cdot \varepsilon} \tag{1}$$

The fraction of light which was absorbed by the actinometer at λ = 397 nm (f) was determined with equation 2 with the absorbance of the ferrioxalate stock solution at λ = 397 nm being $A_{397\,\mathrm{nm}} > 3$ ($f > 0.999$).

$$f = 1 - 10^{-A_{397\,\mathrm{nm}}} \tag{2}$$

The photonflux ϕ_q was finally determined using equation 3 with ϕ_F = 1.13 (at λ = 392 nm) and t = 60 s, giving ϕ_q = 3.418·10^{-9} mol·s^{-1}.[10,18]

$$\phi_q = \frac{n_{Fe(II)}}{\phi_F \cdot t \cdot f} \tag{3}$$

Reaction quantum yield for the formation of 3a

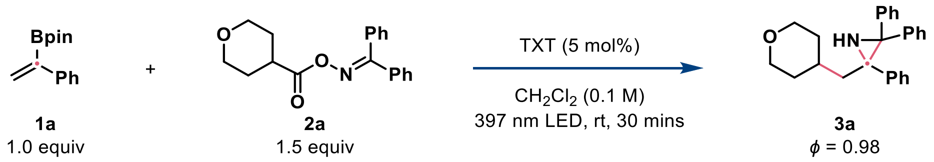

An oven-dried 10 mL Schlenk tube was charged with a Teflon-coated stirring bar, alkene **1a** (23.0 mg, 0.100 mmol, 1.0 equiv), reagent **2a** (46.4 mg, 0.150 mmol, 1.5 equiv), and thioxanthone (1.1 mg, 5.2 µmol, 5 mol%). The tube was evacuated and backfilled with argon three times. Dry CH_2Cl_2 (1.0 mL, 0.10 M) was added and the mixture was then irradiated at 397 nm for 1800 s using the described setup. After that, the solvent was removed in vacuo and the residue was examined by ^{1}H NMR spectroscopy with mesitylene as internal standard. The NMR yield was determined to be 6% (average of two reactions).

Note: The solvent was changed from ethyl acetate to CH_2Cl_2 to ensure better solubility and a better reproducibility of the resulting yield.

The reaction's quantum yield was determined using equation 4 with the determined photon flux ϕ_q, the irradiation time t = 1800 s, and the fraction of light absorbed ($f_R > 0.999$; determined according to equation 2 with $A_{397\,\mathrm{nm}} > 3$) by the reaction mixture.

$$\phi = \frac{n_{\text{product}}}{\phi_q \cdot t \cdot f_{\text{R}}} \quad (4)$$

The determined quantum yield was ϕ = 0.98.

Reaction quantum yield for the formation of 6

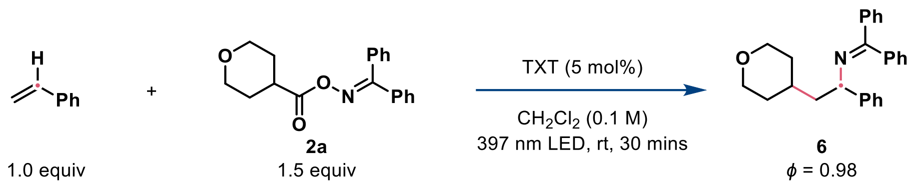

An oven-dried 10 mL Schlenk tube was charged with a Teflon-coated stirring bar, reagent **2a** (46.4 mg, 0.150 mmol, 1.5 equiv), and thioxanthone (1.1 mg, 5.2 µmol, 5 mol%). The tube was evacuated and backfilled with argon three times. Dry CH_2Cl_2 (1.0 mL, 0.10 M) and styrene (10.4 mg, 99.9 µmol, 1.0 equiv) were added and the mixture was then irradiated at 397 nm for 1800 s using the described setup. After that, the solvent was removed in vacuo and the residue was examined by ^{1}H NMR spectroscopy with mesitylene as internal standard. The NMR yield was determined to be 6% (average of two reactions).

The reaction's quantum yield was determined using equation 4 with the determined photon flux ϕ_q, the irradiation time t = 1800 s, and the fraction of light absorbed (f_R > 0.999; determined according to equation 2 with $A_{397\,nm}$ > 3) by the reaction mixture. The determined quantum yield was ϕ = 0.98.

To clearly identify product **6** in the reaction mixture, we isolated this compound using the following procedure.

Isolation of 6

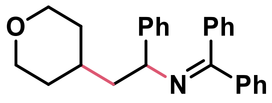

An oven-dried 10 mL Schlenk tube was charged with a Teflon-coated stirring bar, reagent **2a** (92.8 mg, 0.300 mmol, 1.5 equiv), and thioxanthone (2.2 mg, 10 µmol, 5 mol%). The tube was evacuated and backfilled with argon three times. Dry ethyl acetate (2.0 mL, 0.10 M) and styrene (20.8 mg, 0.200 mmol, 1.0 equiv) were added and the mixture was then irradiated at 405 nm for 16 h. After that, the solvent was removed in vacuo, and column chromatography (n-pentane/ethyl acetate = 24:1) gave the title compound as a colorless oil (59 mg, 0.160 mmol, 80%).

^{1}H NMR (400 MHz, $CDCl_3$) δ 7.73 – 7.66 (m, 2H), 7.47 – 7.17 (m, 11H), 7.10 – 7.03 (m, 2H), 4.53 – 4.44 (m, 1H), 3.92 – 3.80 (m, 2H), 3.35 – 3.19 (m, 2H), 2.00 – 1.88 (m, 1H), 1.79 – 1.67 (m, 1H), 1.56 – 1.36 (m, 2H), 1.35 – 1.06 (m, 3H).

^{13}C{^{1}H} NMR (101 MHz, $CDCl_3$) δ 166.6, 145.4, 140.0, 137.1, 130.0, 128.7, 128.5, 128.5, 128.4, 128.1, 127.9, 127.2, 126.8, 68.1, 68.0, 63.6, 47.2, 33.6, 33.0, 31.9.

HRMS (ESI): *m/z* calculated for $[C_{26}H_{27}NONa]^+$ $[M+Na]^+$: 392.1985; found: 392.1981.

5.3 Light-free aziridination

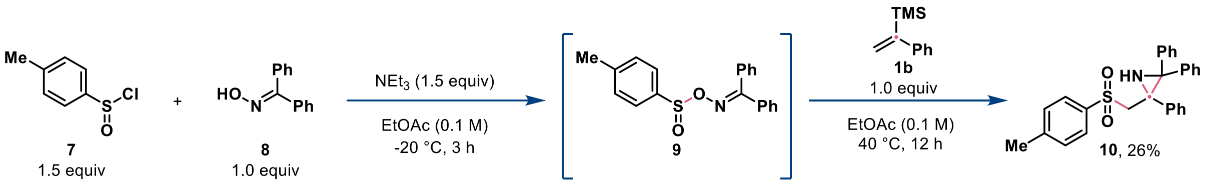

The light-free alkene functionalization reaction was carried out according to an adapted literature procedure, and compound **7** was prepared as described therein.[19] Compound **7** (52.4 mg, 0.300 mmol, 1.5 equiv) was dissolved in EtOAc (1.0 mL) and cooled to -20 °C. Oxime **8** (39.5 mg, 0.200 mmol, 1.0 equiv) in EtOAc (1.0 mL) was added at -20 °C followed by NEt_3 (30.4 mg, 0.300 mmol, 1.5 equiv). The reaction mixture was stirred for 3 h to form reagent **9**, to which alkene **1b** (35.3 mg, 0.200 mmol, 1.0 equiv) was added. The reaction was then heated up to 40 °C and was stirred for another 12 h. The solvent was removed and the residue was purified by column chromatography (100% *n*-pentane to *n*-pentane/ethyl acetate = 10:1) giving the desired aziridine **10** as an off-white solid (23.0 mg, 52.3 µmol, 26%).

^{1}H NMR (500 MHz, $CDCl_3$) δ = 7.50 – 7.47 (m, 2H), 7.45 – 7.42 (m, 2H), 7.41 – 7.36 (m, 2H), 7.33 – 7.27 (m, 1H), 7.18 – 7.14 (m, 2H), 7.00 – 6.91 (m, 6H), 6.91 – 6.86 (m, 1H), 6.86 – 6.81 (m, 3H), 4.29 (d, *J* = 14.9 Hz, 1H), 3.14 (d, *J* = 15.0 Hz, 1H), 2.28 (s, 3H).

^{13}C{^{1}H} NMR (126 MHz, $CDCl_3$) δ = 144.4, 140.5, 139.4, 136.9, 136.1, 129.4, 129.3, 128.7, 128.4, 128.2, 128.1, 127.9, 127.6, 127.5, 126.5, 126.3, 62.2, 56.4, 46.8, 21.6.

HRMS (ESI): *m/z* calculated for $[C_{28}H_{25}NNaO_2S]^+$ $[M+Na]^+$: 462.1498; found: 462.1499.

Note: This structure was additionally confirmed by single crystal X-Ray analysis.

5.4 Reaction in the presence of H_2O

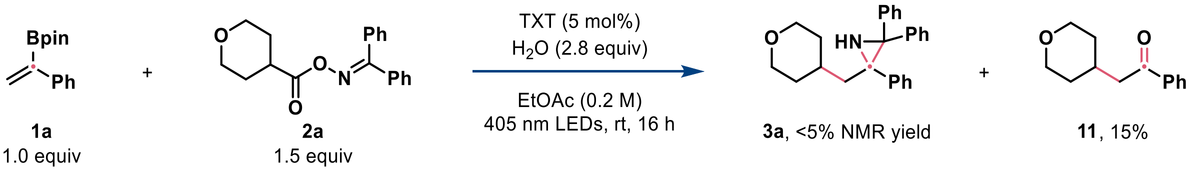

An oven-dried 10 mL Schlenk tube was charged with a Teflon-coated stirring bar, alkene **1a** (46.0 mg, 0.200 mmol, 1.0 equiv), reagent **2a** (92.8 mg, 0.300 mmol, 1.5 equiv), and thioxanthone (2.2 mg, 10 µmol, 5 mol%). The tube was evacuated and backfilled with argon three times. Dry ethyl acetate (1.0 mL, 0.20 M) was added, followed by H_2O (10.0 mg, 0.555 mmol, 2.8 equiv), and the mixture was then irradiated at 405 nm for 16 h. After that, the solvent was removed in vacuo and the residue was examined by ^{1}H NMR spectroscopy with mesitylene as internal standard. The NMR yield of **3a** was determined to be below 5%.

Purification of the crude mixture by column chromatography (100% *n*-pentane to *n*-pentane/ethyl acetate = 10:1), followed by preparative TLC (*n*-pentane/ethyl acetate = 9:1) gave ketone **11** as an off-white solid (6.0 mg, 29 µmol, 15%).

^{1}H NMR (400 MHz, $CDCl_3$) δ = 7.98 – 7.92 (m, 2H), 7.60 – 7.54 (m, 1H), 7.50 – 7.43 (m, 2H), 3.95 (ddd, *J* = 11.7, 3.9, 1.6 Hz, 2H), 3.45 (td, *J* = 11.8, 2.1 Hz, 2H), 2.90 (d, *J* = 6.6 Hz, 2H), 2.36 – 2.18 (m, 1H), 1.73 – 1.64 (m, 2H), 1.46 – 1.33 (m, 2H).
^{13}C{^{1}H} NMR (101 MHz, $CDCl_3$) δ = 199.4, 137.4, 133.3, 128.8, 128.2, 68.0, 45.5, 33.2, 31.5.
The spectroscopic data is in agreement with the literature.[20]

5.5 Low temperature reaction & delayed formation of 3a

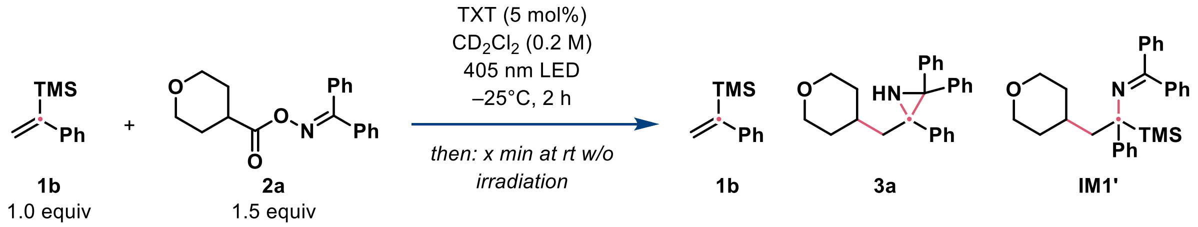

An oven-dried 10 mL Schlenk tube was charged with a Teflon-coated stirring bar, alkene **1b** (35.3 mg, 0.200 mmol, 1.0 equiv), reagent **2a** (92.8 mg, 0.300 mmol, 1.5 equiv), and thioxanthone (2.1 mg, 9.9 µmol, 5 mol%). The tube was evacuated and backfilled with argon three times and then, dry and degassed CD_2Cl_2 (1.0 mL, 0.20 M) was added. The mixture was irradiated for 2 h with a 405 nm LED in an ethanol bath cooled to -25 °C by a Julabo FT 401 immersion cooler. Subsequently, CH_2Br_2 was added as internal standard, and the sample was quickly transferred to an NMR tube under argon. The tube was kept at rt, and ^{1}H NMR spectra of this sample were acquired after the indicated times. The extracted data is displayed below:

Supplementary Table 3. Yields of **1b**, **3a**, and **IM1’** after the indicated times.

Entry	*t* / min [a]	yield (**1b**) / % [b]	yield (**3a**) / % [b]	yield (**IM1‘**) / % [b]
1	10	38	11	23
2	81	38	16	18
3	127	38	18	16
4	160	38	21	13

[a] *t* refers to the time between interruption of irradiation and start of the NMR measurement.

[b] Determined by ^{1}H NMR spectroscopy with CH_2Br_2 as internal standard.

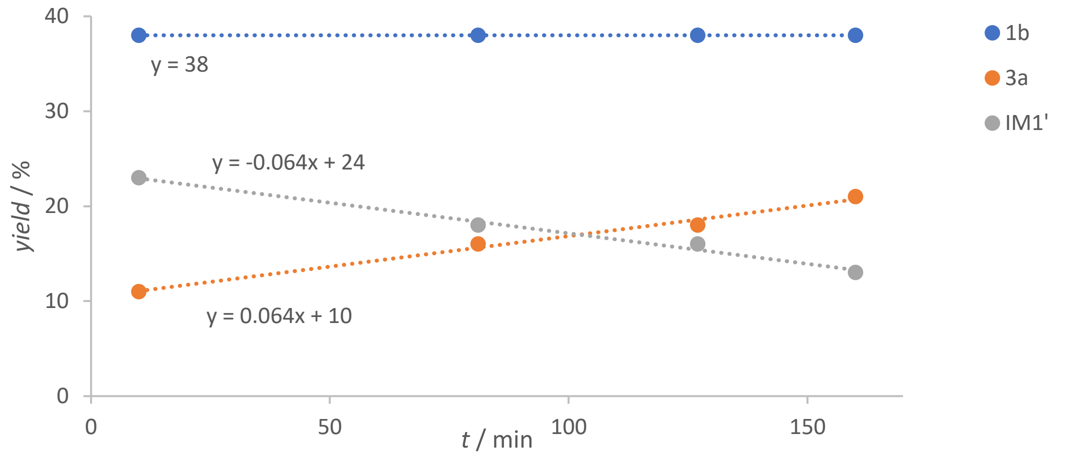

Supplementary Figure 8. Visualization of the data shown in **Supplementary Table 3**.

*Discussion: The amount of alkene **1b** remained constant throughout the observation period, suggesting that conversion of **1b** in the reaction mixture requires irradiation. The yield of **3a** increased linearly. Since **1b** was not further converted, the increase in yield needs to result from conversion of an intermediate in the mixture formed during the irradiation period. Another species (**IM1’**) in the crude NMR was observed and was found to decrease in the same way as the yield of **3a** increased. Consequently, the data suggests that **IM1’** is formed photochemically and is then thermally converted to **3a**.*

*Comment: Since **IM1’** could not be isolated due to its rapid decomposition (see above), the drawn structure of **IM1’** is a reasonable suggestion consistent with the proposed mechanism and the other mechanistic insights. **IM1’** was quantified using its distinct TMS signal at 0.04 ppm.*

6. Preliminary downstream modifications

Acidic treatment of aziridine 3t towards indene 12

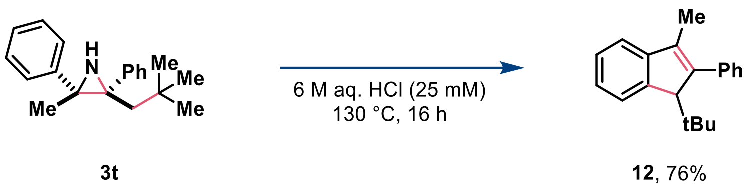

The conditions were adapted from a literature report.[21] A 10 mL Schlenk tube was charged with a Teflon-coated stirring bar and aziridine **3t** (27.9 mg, 99.8 µmol, 1.0 equiv). Aqueous HCl (6.0 M; 4.0 mL, 25 mM) was added, the tube was closed, and the reaction mixture was stirred at 130 °C for 16 h. After cooling to room temperature, the pH was adjusted to 8 using aq. sat. $NaHCO_3$. The aqueous layer was extracted with ethyl acetate (3x 20 mL) and the combined organic layers were dried over $MgSO_4$. The solvent was removed and the residue was purified by column chromatography (*n*-pentane/ethyl acetate = 19:1) giving indene **12** as a colourless oil (20.0 mg, 76.2 µmol, 76%).

^{1}H NMR (599 MHz, $CDCl_3$) δ = 7.56 (dq, *J* = 7.5, 0.8 Hz, 1H), 7.41 – 7.37 (m, 2H), 7.34 – 7.26 (m, 5H), 7.18 (ddd, *J* = 7.5, 5.2, 3.4 Hz, 1H), 3.76 (qd, *J* = 1.8, 0.7 Hz, 1H), 2.11 (d, *J* = 1.8 Hz, 3H), 0.81 (s, 9H).

^{13}C{^{1}H} NMR (151 MHz, $CDCl_3$) δ = 147.1, 145.8, 145.7, 139.8, 135.8, 129.5, 128.1, 126.7, 126.5, 125.3, 124.1, 118.9, 61.9, 35.4, 29.2, 11.3.

HRMS (EI): *m/z* calculated for $[C_{20}H_{22}]^+$ $[M]^+$: 262.1716; found: 262.1719.

Hydrogenation of aziridine 3d towards 13

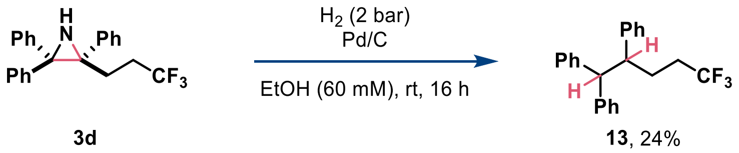

The conditions were adapted from a literature report.[21] An oven-dried 10 mL Schlenk tube was charged with a Teflon-coated stirring bar, aziridine **3d** (22.0 mg, 59.9 µmol, 1.0 equiv) and Pd/C (10 wt%; 22.0 mg). The tube was evacuated and backfilled with argon three times. Dry ethanol (1.0 mL, 60 mM) was added, and the tube was purged with hydrogen for 1 min, before the tube was closed and the hydrogen pressure was set to 2.0 bar. After stirring at room temperature for 16 h, the reaction mixture was passed through a pad of Celite eluting with ethyl acetate (2 mL).

The solvent was removed and the residue was purified by preparative TLC (*n*-pentane/ethyl acetate = 9:1) giving **13** as a colourless oil (5.0 mg, 14 µmol, 24%).

^{1}H NMR (400 MHz, $CDCl_3$) δ = 7.41 – 7.37 (m, 2H), 7.33 (t, *J* = 7.5 Hz, 2H), 7.24 – 7.13 (m, 3H), 7.12 – 7.00 (m, 7H), 6.97 – 6.90 (m, 1H), 4.09 (d, *J* = 11.5 Hz, 1H), 3.44 (td, *J* = 11.3, 3.4 Hz, 1H), 1.97 – 1.90 (m, 1H), 1.86 – 1.67 (m, 3H).

^{13}C{^{1}H} NMR (126 MHz, $CDCl_3$) δ = 143.4, 143.3, 141.9, 129.0, 128.5, 128.4, 128.2, 128.2, 128.1, 127.3 (q, *J* = 276.6 Hz), 126.8, 126.6, 126.0, 58.9, 49.3, 32.0 (q, *J* = 28.3 Hz), 27.2 (q, *J* = 2.9 Hz).

^{19}F{^{1}H} NMR (377 MHz, $CDCl_3$) *δ* -66.0.

HRMS (EI): *m/z* calculated for $[C_{23}H_{19}F_3]^+$ $[M-H_2]^+$: 352.1433; found: 352.1444.

7. X-Ray analysis

X-Ray diffraction: Data sets for compounds **3b**, **3i**, **3v-major**, **3x-major**, **3ab-major**, **3ac-minor**, **3ae-major** and **10** were collected with a Bruker D8 Venture Photon III Diffractometer. Programs used: data collection: *APEX6* Version 2024.9-0 [1] (Bruker AXS Inc., **2024**); cell refinement: *SAINT* Version 8.41 (Bruker AXS Inc., **2024**); data reduction: *SAINT* Version 8.41 (Bruker AXS Inc., **2024**); absorption correction, *SADABS* Version 2016/2 (Bruker AXS Inc., **2024**); structure solution *SHELXT*-Version 2018-3 [2] (Sheldrick, G. M. *Acta Cryst.,* **2015**, *A71*, 3-8); structure refinement *SHELXL*- Version 2019-2 [3] (Sheldrick, G. M. *Acta Cryst.,* **2015**, *C71* (1), 3-8) and graphics, *XP* [4] (Version 5.1, Bruker AXS Inc., Madison, Wisconsin, USA, **1998**). *R*-values are given for observed reflections, and wR^2 values are given for all reflections.
Exceptions and special features: For compound **3b** two phenyl rings, for compound **3v-major** the entire molecule and for compound **3ab-major** the *tert*-butyl group were found disordered over two positions in the asymmetric unit. Several restraints (SADI, SAME, ISOR, SUMP and SIMU) were used in order to improve refinement stability.

X-ray crystal structure analysis of 3b (glo10827): A colourless, needle shaped specimen of $C_{25}H_{25}N$, approximate dimensions 0.055×0.058×0.149 mm³, was used for the X-ray crystallographic analysis. The crystals were crystallised from heptane. The X-ray intensity data of **3b** were measured on a Bruker D8 VENTURE KAPPA diffractometer system equipped with a microfocus sealed tube (λ = 0.71073 Å) and a multilayer mirror monochromator. The specimen was held at 100(2) K during the measurement with an Oxford Cryostream 1000 low temperature device. A total of 1568 frames were collected. The total exposure time was 18.96 hours. The frames were integrated with the SAINT V8.41 package using a narrow-frame algorithm. The integration of the data using a monoclinic unit cell yielded a total of 39763 reflections to a maximum θ angle of 25.02° (0.84 Å resolution), of which 3335 were independent (average redundancy 11.92, completeness = 99.8%, Rint = 12.53%, Rsig = 5.99%) and 2463 (73.9%) were greater than $2\sigma(F^2)$. The final cell constants of a = 12.6653(12) Å, b = 6.1359(7) Å, c = 24.542(3) Å, volume = 1894.5(3) Å³, are based upon the refinement of the XYZ-centroids of 7218 reflections above 20 $\sigma(I)$ with 2.19° < 2θ < 25.34°. Data were corrected for absorption effects using the Multi-Scan method in SADABS 2016/2. The calculated minimum and maximum transmission coefficients (based on crystal size) are 0.990 and 0.996. The structure was solved by SHELXT 2018/2 and refined using the SHELXL-2019/2 Software, in the space group $P2_1/c$ (14), with Z = 4 for the formula unit $C_{25}H_{25}N$. The final anisotropic full-matrix least-squares refinement on F^2 with 350 variables against 3335 data points 338 and 338 restraints converged at R_1 = 7.55%, for the observed data and wR_2 = 20.70% for all data. The goodness-of-fit on F^2 was 1.10. The largest peak

in the final difference electron density synthesis was 0.56 e⁻/Å^3 and the deepest hole was −0.20 e⁻/Å^3 with an RMS deviation of 0.070 e⁻/Å^3. On the basis of the final model, the calculated density was 1.19 g/cm^3 and *F*(000), 728 e⁻. The hydrogen at N1 atom was refined freely. CCDC number: 2485003.

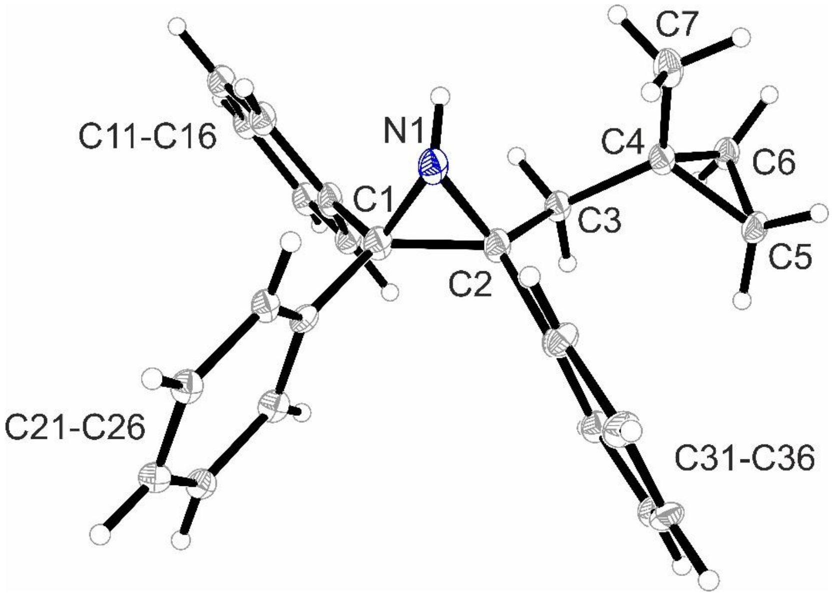

Supplementary Figure 9. Crystal structure of compound **3b**. Thermal ellipsoids are shown at 30% probability.

X-ray crystal structure analysis of 3i (glo10658): A colorless, prism-like specimen of $C_{23}H_{29}B_{10}N$, approximate dimensions 0.120 mm x 0.223 mm x 0.335 mm, was used for the X-ray crystallographic analysis. The X-ray intensity data were measured on a single crystal diffractometer Bruker D8 Venture Photon III system equipped with a micro focus tube Mo ImS (MoKα, λ = 0.71073 Å) and a MX mirror monochromator. A total of 1023 frames were collected. The total exposure time was 1.94 hours. The frames were integrated with the Bruker SAINT software package using a narrow-frame algorithm. The integration of the data using a triclinic unit cell yielded a total of 64474 reflections to a maximum θ angle of 27.47° (0.77 Å resolution), of which 11078 were independent (average redundancy 5.820, completeness = 99.8%, R_{int} = 6.87%, R_{sig} = 4.42%) and 9655 (87.15%) were greater than 2σ(F^2). The final cell constants of <u>a</u> = 9.2130(3) Å, <u>b</u> = 11.2557(5) Å, <u>c</u> = 23.6855(10) Å, α = 91.091(2)°, β = 96.8890(10)°, γ = 96.3710(10)°, volume = 2422.06(17) Å^3, are based upon the refinement of the XYZ-centroids of 9011 reflections above 20 σ(I) with 4.946° < 2θ < 54.93°. Data were corrected for absorption effects using the multi-scan method (SADABS). The ratio of minimum to maximum apparent transmission was 0.955. The calculated minimum and maximum transmission coefficients (based on crystal size) are 0.9800 and 0.9930. The structure was solved and refined using the Bruker SHELXTL Software Package, using the space group *P*-1, with Z = 4 for the formula

unit, $C_{23}H_{29}B_{10}N$. The final anisotropic full-matrix least-squares refinement on F^2 with 709 variables converged at R1 = 5.51%, for the observed data and wR2 = 12.59% for all data. The goodness-of-fit was 1.122. The largest peak in the final difference electron density synthesis was 0.362 $e^-/Å^3$ and the largest hole was -0.282 $e^-/Å^3$ with an RMS deviation of 0.046 $e^-/Å^3$. On the basis of the final model, the calculated density was 1.173 g/cm^3 and F(000), 896 e^-. The hydrogens at N1A, N1B and the carboranyl groups were refined freely. CCDC number: 2485004.

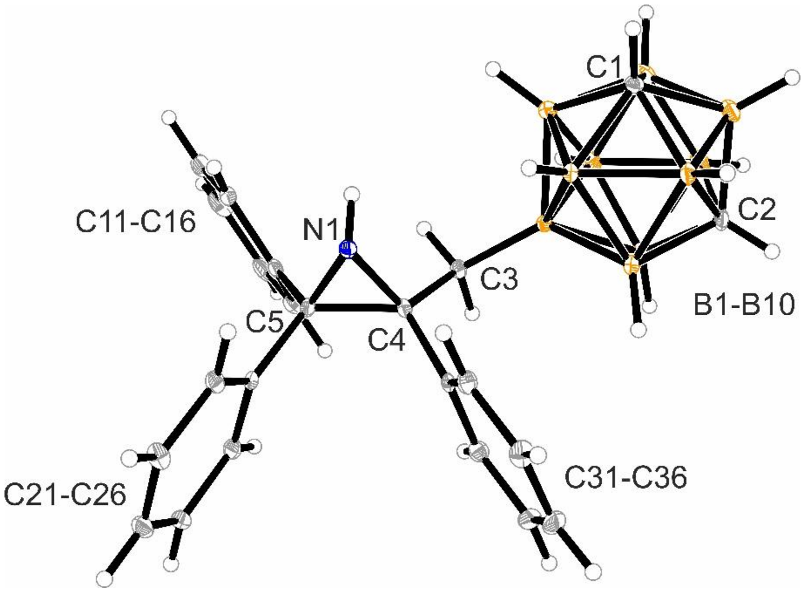

Supplementary Figure 10. Crystal structure of compound **3i**. Only one molecule (molecule named with suffix A) of two found in the asymmetric unit is shown. Thermal ellipsoids are shown at 30% probability.

X-ray crystal structure analysis of 3v-major (glo10824): A colourless, prism shaped specimen of $C_{20}H_{24}IN$, approximate dimensions 0.047×0.08×0.082 mm^3, was used for the X-ray crystallographic analysis. The crystals were crystallised from DCM, heptane and trichloromethane. The X-ray intensity data of **3v-major** were measured on a Bruker D8 VENTURE KAPPA diffractometer system equipped with a microfocus sealed tube (λ = 0.71073 Å) and a multilayer mirror monochromator. A total of 1047 frames were collected. The total exposure time was 3.06 hours. The frames were integrated with the SAINT V8.41 package using a narrow-frame algorithm. The integration of the data using a monoclinic unit cell yielded a total of 44573 reflections to a maximum θ angle of 26.73° (0.79 Å resolution), of which 3954 were independent (average redundancy 11.27, completeness = 99.9%, Rint = 10.75%, Rsig = 4.84%) and 3186 (80.6%) were greater than $2\sigma(F^2)$. The final cell constants of a = 6.0111(15) Å, b = 23.523(5) Å, c = 13.309(3) Å, volume = 1856.7(7) $Å^3$, are based upon the refinement of the XYZ-centroids of 5770 reflections above 20 $\sigma(I)$ with 2.32° < 2θ < 26.08°. Data were corrected for absorption effects using the Multi-Scan method in SADABS 2016/2. The calculated minimum and maximum

transmission coefficients (based on crystal size) are 0.872 and 0.923. The structure was solved by SHELXT 2018/2 and refined using the SHELXL-2019/2 Software, in the space group $P2_1/n$ (14), with Z = 4 for the formula unit $C_{20}H_{24}IN$. The final anisotropic full-matrix least-squares refinement on F^2 with 395 variables against 3954 data points 551 and 551 restraints converged at R_1 = 3.92%, for the observed data and wR_2 = 8.26% for all data. The goodness-of-fit on F^2 was 1.04. The largest peak in the final difference electron density synthesis was 0.53 e⁻/Å^3 and the deepest hole was −0.67 e⁻/Å^3 with an RMS deviation of 0.091 e⁻/Å^3. On the basis of the final model, the calculated density was 1.45 g/cm^3 and F(000), 816 e⁻. The hydrogen at N1 atom is refined freely. The hydrogen at N1A atom is refined freely, but with N-H distance restraints (DFIX) and U-fixed value. CCDC number: 2485005.

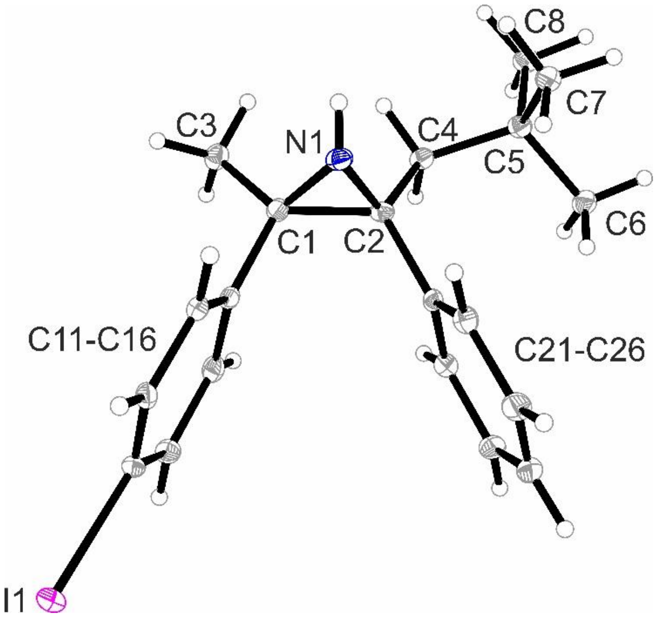

Supplementary Figure 11. Crystal structure of compound **3v-major**. Thermal ellipsoids are shown at 30% probability.

X-ray crystal structure analysis of 3x-major (glo10870): A colourless, prism shaped specimen of $C_{21}H_{24}N_2$, approximate dimensions 0.051×0.071×0.119 mm^3, was used for the X-ray crystallographic analysis. The crystals were crystallised from DCM and hexanol. The X-ray intensity data of **3x-major** were measured on a Bruker D8 VENTURE KAPPA diffractometer system equipped with a microfocus sealed tube (λ = 0.71073 Å) and a multilayer mirror monochromator. The specimen was held at 100(2) K during the measurement with an Oxford Cryostream 1000 low temperature device. A total of 2444 frames were collected. The total exposure time was 11.68 hours. The frames were integrated with the SAINT V8.41 package using a narrow-frame algorithm. The integration of the data using a monoclinic unit cell yielded a total of 59211 reflections to a maximum θ angle of 27.50° (0.77 Å resolution), of

which 3982 were independent (average redundancy 14.87, completeness = 99.9%, Rint = 8.90%, Rsig = 4.67%) and 2998 (75.3%) were greater than 2σ(F^2). The final cell constants of a = 16.7868(10) Å, b = 5.9910(4) Å, c = 17.4224(10) Å, volume = 1736.19(18) Å^3, are based upon the refinement of the XYZ-centroids of 6966 reflections above 20 σ(*I*) with 2.36° < 2θ < 25.55°. Data were corrected for absorption effects using the Multi-Scan method in SADABS 2016/2. The calculated minimum and maximum transmission coefficients (based on crystal size) are 0.992 and 0.997. The structure was solved by SHELXT 2018/2 and refined using the SHELXL-2019/2 Software, in the space group $P2_1/c$ (14), with Z = 4 for the formula unit $C_{21}H_{24}N_2$. The final anisotropic full-matrix least-squares refinement on F^2 with 221 variables against 3982 data points converged at R_1 = 4.26%, for the observed data and wR_2 = 10.92% for all data. The goodness-of-fit on F^2 was 1.03. The largest peak in the final difference electron density synthesis was 0.21 e$^-$/Å^3 and the deepest hole was −0.17 e$^-$/Å^3 with an RMS deviation of 0.037 e$^-$/Å^3. On the basis of the final model, the calculated density was 1.17 g/cm^3 and *F*(000), 656 e$^-$. The hydrogen at N1 atom is refined freely, but disordered over two positions. CCDC number: 2487697.

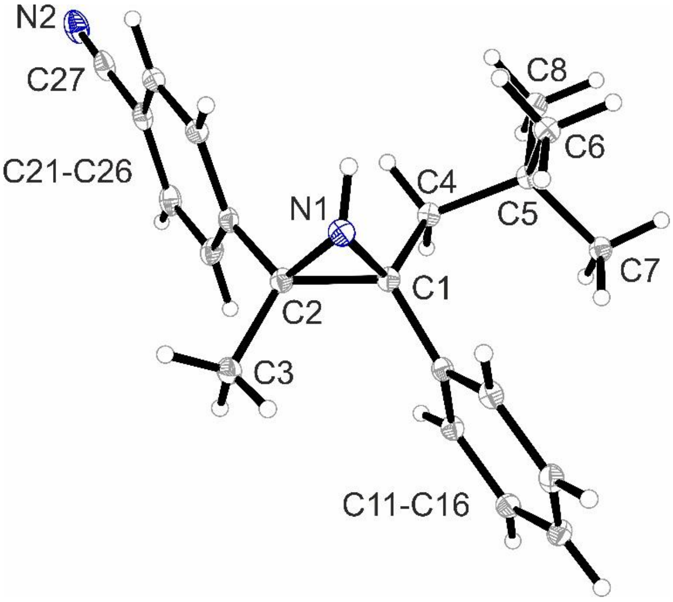

Supplementary Figure 12. Crystal structure of compound **3x-major**. Thermal ellipsoids are shown at 30% probability.

X-ray crystal structure analysis of 3ab-major (glo10833): A colourless, needle shaped specimen of $C_{26}H_{29}N$, approximate dimensions 0.026×0.05×0.268 mm^3, was used for the X-ray crystallographic analysis. The crystals were crystallised from ethanol and heptane. The X-ray

intensity data of **3ab-major** were measured on a Bruker D8 VENTURE KAPPA diffractometer system equipped with a microfocus sealed tube (λ = 1.54178 Å) and a multilayer mirror monochromator. The specimen was held at 100(2) K during the measurement with an Oxford Cryostream 1000 low temperature device. A total of 2264 frames were collected. The total exposure time was 22.88 hours. The frames were integrated with the SAINT V8.41 package using a narrow-frame algorithm. The integration of the data using an orthorhombic unit cell yielded a total of 29318 reflections to a maximum θ angle of 70.36° (0.82 Å resolution), of which 3969 were independent (average redundancy 7.39, completeness = 100.0%, Rint = 7.30%, Rsig = 4.06%) and 3377 (85.1%) were greater than 2σ(F^2). The final cell constants of a = 5.9240(2) Å, b = 15.1270(4) Å, c = 23.2042(6) Å, volume = 2079.38(10) $Å^3$, are based upon the refinement of the XYZ-centroids of 8680 reflections above 20 σ(I) with 3.49° < 2θ < 69.88°. Data were corrected for absorption effects using the Multi-Scan method in SADABS 2016/2. The calculated minimum and maximum transmission coefficients (based on crystal size) are 0.881 and 0.987. The structure was solved by SHELXT 2018/2 and refined using the SHELXL-2019/2 Software, in the space group $P2_12_12_1$ (19), with Z = 4 for the formula unit $C_{26}H_{29}N$. The final anisotropic full-matrix least-squares refinement on F^2 with 264 variables against 3969 data points 75 and 75 restraints converged at R_1 = 4.56%, for the observed data and wR_2 = 12.75% for all data. The goodness-of-fit on F^2 was 1.07. The largest peak in the final difference electron density synthesis was 0.23 $e^-/Å^3$ and the deepest hole was −0.20 $e^-/Å^3$ with an RMS deviation of 0.039 $e^-/Å^3$. On the basis of the final model, the calculated density was 1.14 g/cm^3 and F(000), 768 e^-. The t-Bu group is disordered over two positions. Several restraints (SIMU, SAME, SADI, ISOR and EADP) were used in order to improve refinement stability. The hydrogen at N1 atom was refined freely. CCDC number: 2485006.

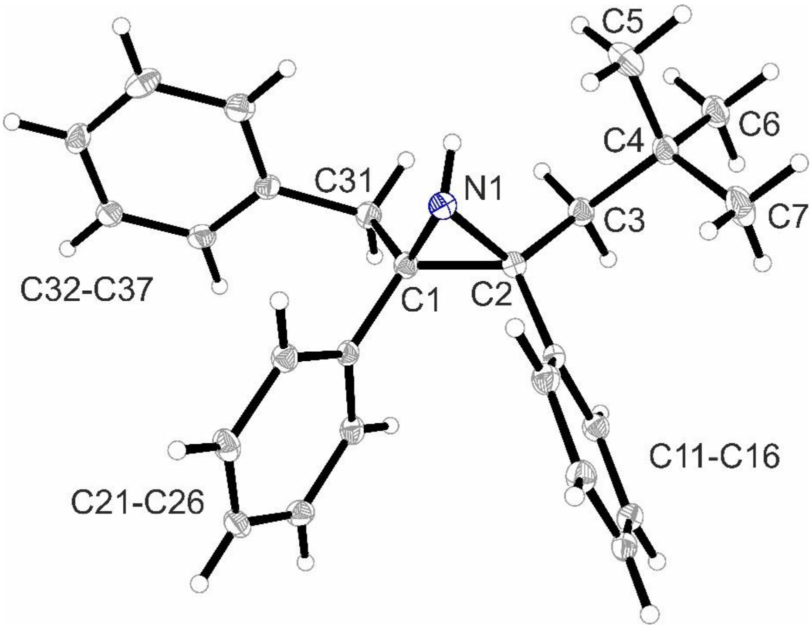

Supplementary Figure 13. Crystal structure of compound **3ab-major**. Thermal ellipsoids are shown at 30% probability.

X-ray crystal structure analysis of 3ac-minor (glo10865): A colourless, plate shaped specimen of $C_{22}H_{27}N$, approximate dimensions 0.031×0.041×0.104 mm^3, was used for the X-ray crystallographic analysis. The crystals were crystallised from DCM and deuterated trichloromethane. The X-ray intensity data of **3ac-minor** were measured on a Bruker D8 VENTURE KAPPA diffractometer system equipped with a microfocus sealed tube (λ = 0.71073 Å) and a multilayer mirror monochromator. The specimen was held at 100(2) K during the measurement with an Oxford Cryostream 1000 low temperature device. A total of 1612 frames were collected. The total exposure time was 21.37 hours. The frames were integrated with the SAINT V8.41 package using a narrow-frame algorithm. The integration of the data using a monoclinic unit cell yielded a total of 45411 reflections to a maximum θ angle of 25.35° (0.83 Å resolution), of which 3263 were independent (average redundancy 13.92, completeness = 99.9%, Rint = 13.92%, Rsig = 7.89%) and 2111 (64.7%) were greater than 2σ(F^2). The final cell constants of a = 6.0083(7) Å, b = 16.527(2) Å, c = 18.017(2) Å, volume = 1779.9(4) Å^3, are based upon the refinement of the XYZ-centroids of 5227 reflections above 20 σ(I) with 2.46° < 2θ < 22.98°. Data were corrected for absorption effects using the Multi-Scan method in SADABS 2016/2. The calculated minimum and maximum transmission coefficients (based on crystal size) are 0.993 and 0.998. The structure was solved by SHELXT 2018/2 and refined using the SHELXL-2019/2 Software, in the space group $P2_1/n$ (14), with Z = 4 for the formula unit $C_{22}H_{27}N$. The final anisotropic full-matrix least-squares refinement on F^2 with 215 variables against 3263 data points 200 and 200 restraints converged at R_1 = 7.29%, for the observed data and wR_2 = 21.91% for all data. The goodness-of-fit on F^2 was 1.03. The largest peak in the final difference electron

density synthesis was 0.64 e⁻/Å^3 and the deepest hole was −0.25 e⁻/Å^3 with an RMS deviation of 0.063 e⁻/Å^3. On the basis of the final model, the calculated density was 1.14 g/cm^3 and F(000), 664 e⁻. The hydrogen at N1 atom was refined freely but with N-H distance restraints (DFIX). CCDC number: 2485007.

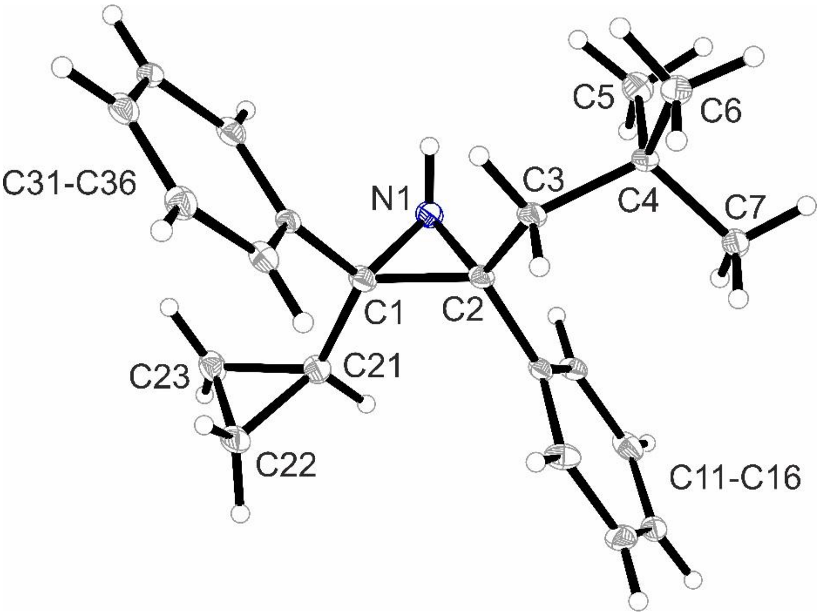

Supplementary Figure 14. Crystal structure of compound **3ac-minor**. Thermal ellipsoids are shown at 30% probability.

X-ray crystal structure analysis of 3ae-major (glo10825): A colourless, prism shaped specimen of $C_{22}H_{27}N$, approximate dimensions 0.046×0.047×0.147 mm^3, was used for the X-ray crystallographic analysis. The crystals were crystallised from DCM, ethyl acetate and pentane. The X-ray intensity data of **3ae-major** were measured on a Bruker D8 VENTURE KAPPA diffractometer system equipped with a microfocus sealed tube (λ = 0.71073 Å) and a multilayer mirror monochromator. A total of 1087 frames were collected. The total exposure time was 6.46 hours. The frames were integrated with the SAINT V8.41 package using a narrow-frame algorithm. The integration of the data using an orthorhombic unit cell yielded a total of 24340 reflections to a maximum θ angle of 26.72° (0.79 Å resolution), of which 3727 were independent (average redundancy 6.53, completeness = 99.7%, Rint = 6.96%, Rsig = 4.17%) and 3287 (88.2%) were greater than 2σ(F^2). The final cell constants of a = 5.9402(2) Å, b = 14.7255(5) Å, c = 20.0572(8) Å, volume = 1754.45(11) Å^3, are based upon the refinement of the XYZ-centroids of 6146 reflections above 20 σ(I) with 2.46° < 2θ < 26.61°. Data were corrected for absorption effects using the Multi-Scan method in SADABS 2016/2. The calculated minimum and maximum transmission coefficients (based on crystal size) are 0.990 and 0.997. The structure was solved by SHELXT 2018/2 and refined using the SHELXL-2019/2 Software, in the space group $P2_12_12_1$ (19), with Z = 4 for the formula unit $C_{22}H_{27}N$.[3, 4] The final anisotropic full-matrix least-squares

refinement on F^2 with 215 variables against 3727 data points converged at R_1 = 3.73%, for the observed data and wR_2 = 8.66% for all data. The goodness-of-fit on F^2 was 1.04. The largest peak in the final difference electron density synthesis was 0.16 e⁻/Å^3 and the deepest hole was −0.15 e⁻/Å^3 with an RMS deviation of 0.034 e⁻/Å^3. On the basis of the final model, the calculated density was 1.16 g/cm^3 and F(000), 664 e⁻. The hydrogen at N1 atom was refined freely. CCDC number: 2485008.

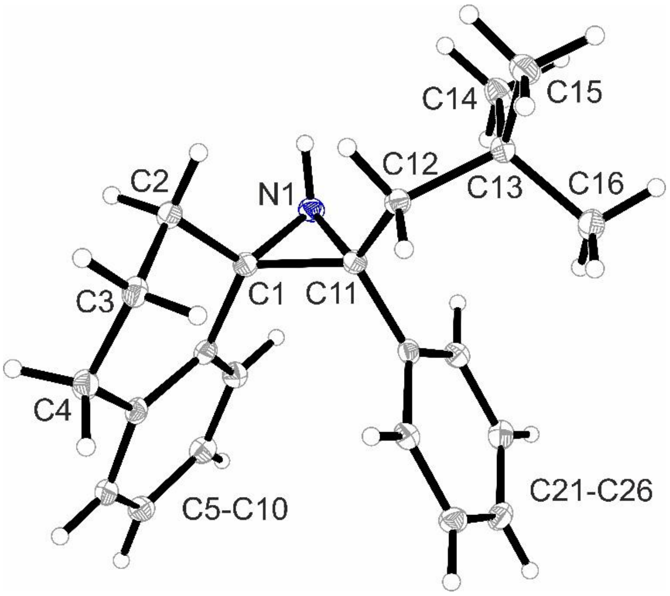

Supplementary Figure 15. Crystal structure of compound **3ae-major**. Thermal ellipsoids are shown at 30% probability.

X-ray crystal structure analysis of 10 (glo10883): A colourless, prism shaped specimen of $C_{28}H_{25}NO_2S$, approximate dimensions 0.044×0.062×0.135 mm^3, was used for the X-ray crystallographic analysis. The crystals were crystallised from DCM and hexane. The X-ray intensity data of **10** were measured on a Bruker D8 VENTURE KAPPA diffractometer system equipped with a microfocus sealed tube (λ = 0.71073 Å) and a multilayer mirror monochromator. A total of 1290 frames were collected. The total exposure time was 12.03 hours. The frames were integrated with the SAINT V8.41 package using a narrow-frame algorithm. The integration of the data using a triclinic unit cell yielded a total of 62582 reflections to a maximum θ angle of 25.04° (0.84 Å resolution), of which 4029 were independent (average redundancy 7.92, completeness = 99.9%, Rint = 20.04%, Rsig = 10.16%) and 2645 (65.6%) were greater than 2σ(F^2). The final cell constants of a = 6.5545(18) Å, b = 12.182(3) Å, c = 15.516(4) Å, volume = 1141.3(5) Å^3, are based upon the refinement of the XYZ-centroids of 2944 reflections above 20 σ(I) with 2.84° < 2θ < 23.76°. Data were corrected for absorption effects using the Multi-Scan method in SADABS 2016/2. The calculated minimum and maximum transmission coefficients (based on crystal size) are 0.978 and 0.993. The structure was solved by SHELXT 2018/2 and refined using the SHELXL-

2019/2 Software, in the space group *P*-1 (2), with Z = 2 for the formula unit $C_{28}H_{25}NO_2S$. The final anisotropic full-matrix least-squares refinement on F^2 with 295 variables against 4029 data points 1 and 1 restraints converged at R_1 = 8.55%, for the observed data and wR_2 = 23.48% for all data. The goodness-of-fit on F^2 was 1.04. The largest peak in the final difference electron density synthesis was 0.74 e⁻/Å^3 and the deepest hole was −0.44 e⁻/Å^3 with an RMS deviation of 0.126 e⁻/Å^3. On the basis of the final model, the calculated density was 1.28 g/cm^3 and *F*(000), 464 e⁻. The hydrogen at N1 atom was refined freely, but with N-H distance restraints (DFIX). CCDC number: 2485009.

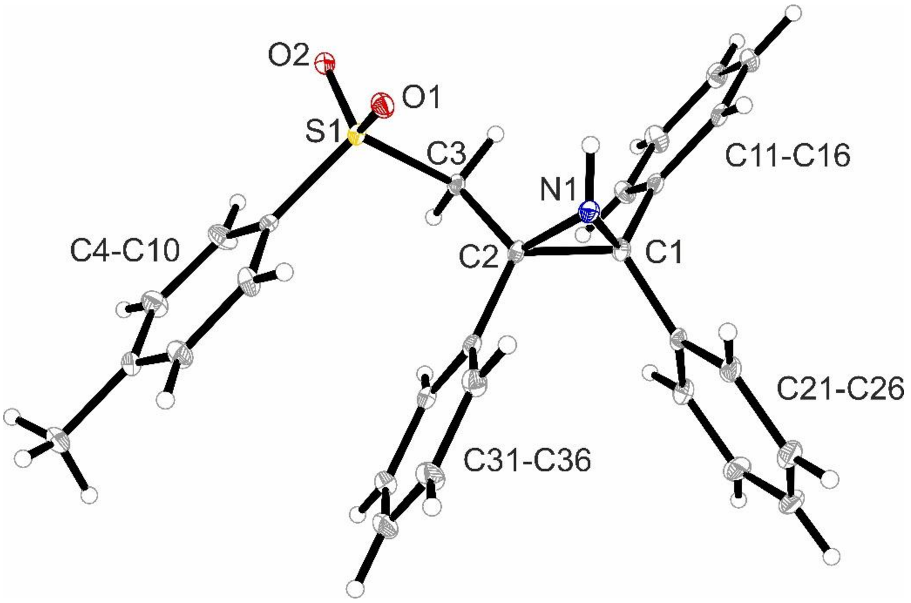

Supplementary Figure 16. Crystal structure of compound **10**. Thermal ellipsoids are shown at 30% probability.

References for the X-Ray analysis:

1. Bruker AXS (**2024**) *APEX6 Version 2024.9-0*, *SAINT Version 8.41* and *SADABS Bruker AXS area detector scaling and absorption correction Version 2016/2*, Bruker AXS Inc., Madison, Wisconsin, USA.
2. Sheldrick, G. M., *SHELXT – Integrated space-group and crystal-structure determination*, *Acta Cryst.*, **2015**, *A71*, 3-8.
3. Sheldrick, G.M., *Crystal structure refinement with SHELXL*, *Acta Cryst.*, **2015**, *C71 (1)*, 3-8.
4. Bruker AXS (**1998**) *XP – Interactive molecular graphics, Version 5.1,* Bruker AXS Inc., Madison, Wisconsin, USA.

8. NMR spectra

8.1 Starting materials

^{1}H NMR ($CDCl_3$, 400 MHz) for **1b**

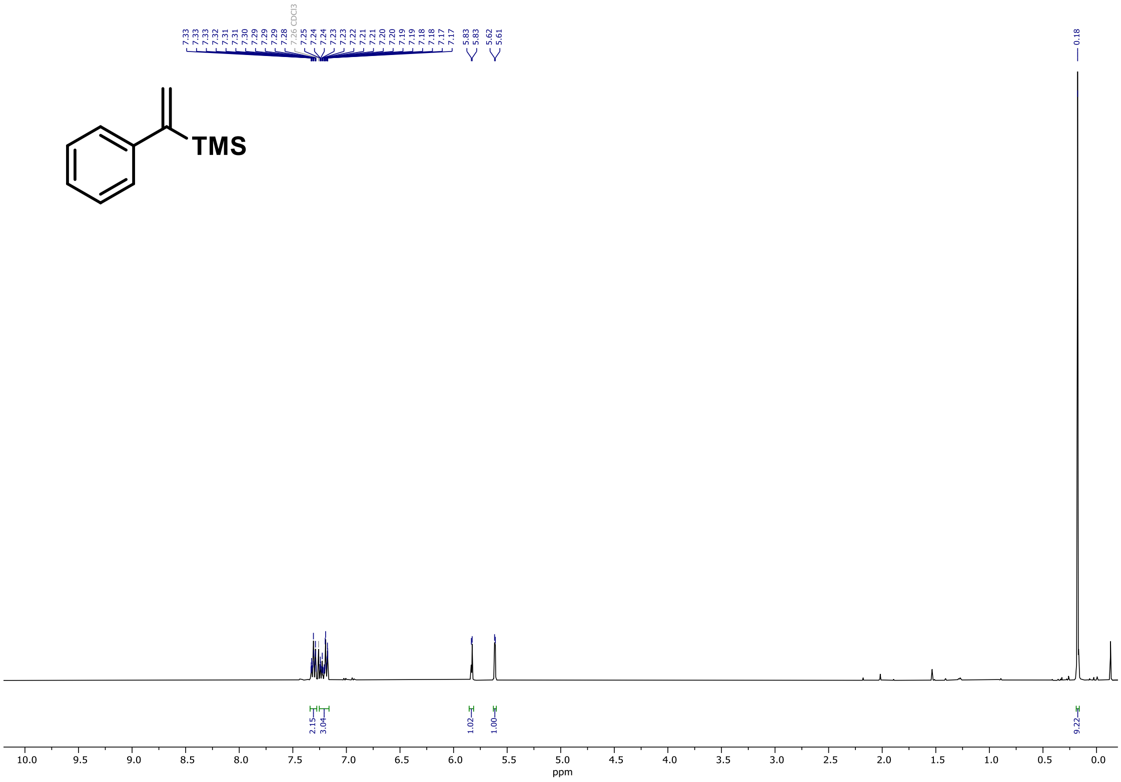

^{13}C{^{1}H} NMR ($CDCl_3$, 101 MHz) for **1b**

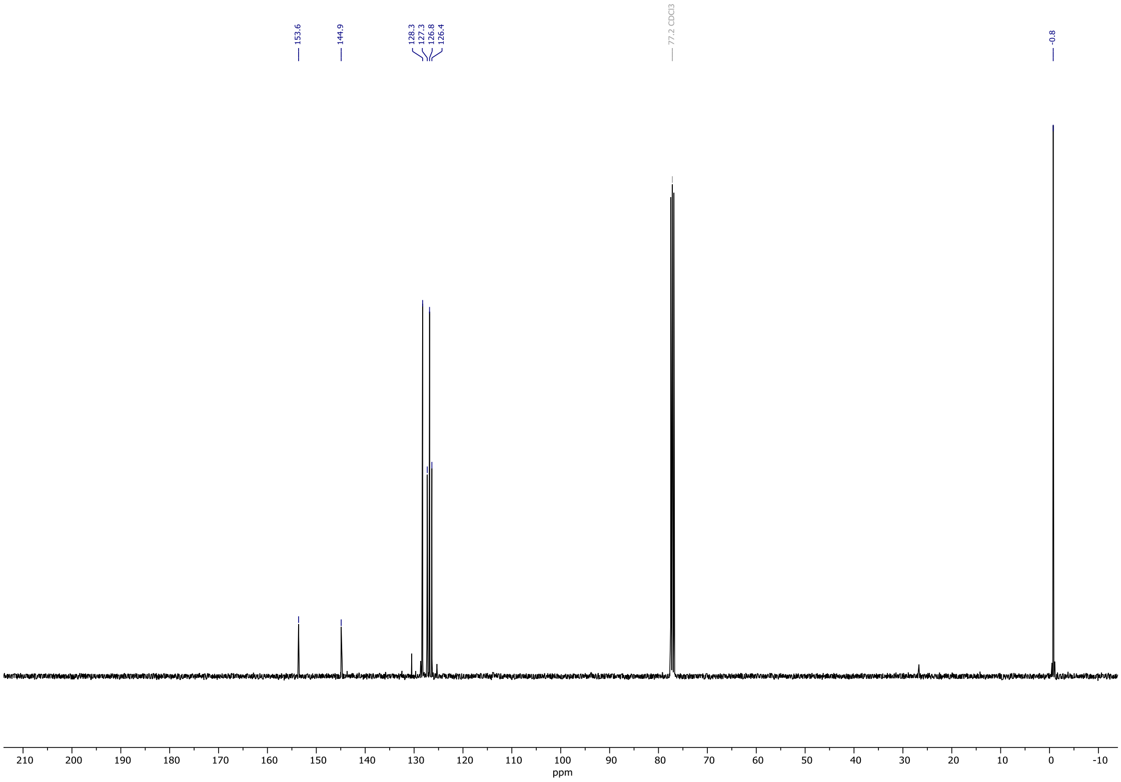

^{1}H NMR ($CDCl_3$, 599 MHz) for **1c**

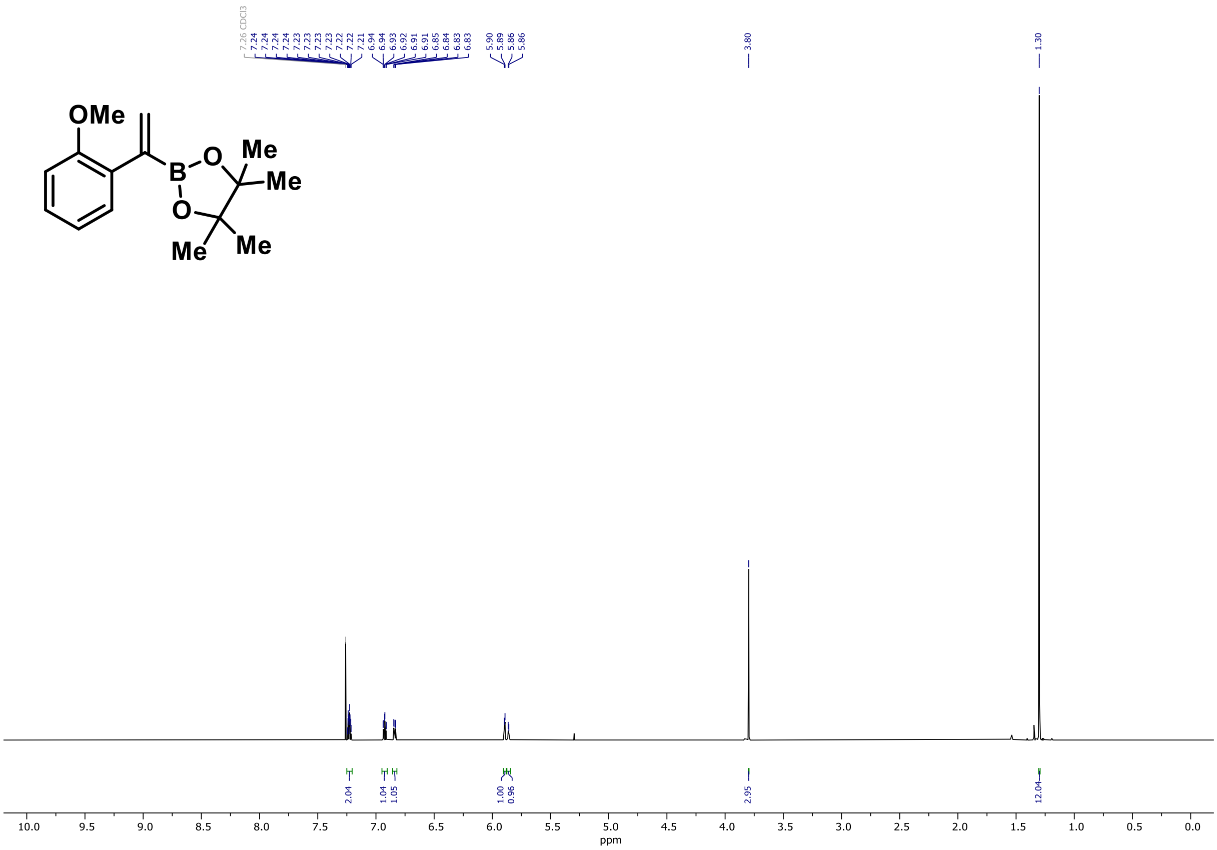

^{13}C{^{1}H} NMR ($CDCl_3$, 151 MHz) for **1c**

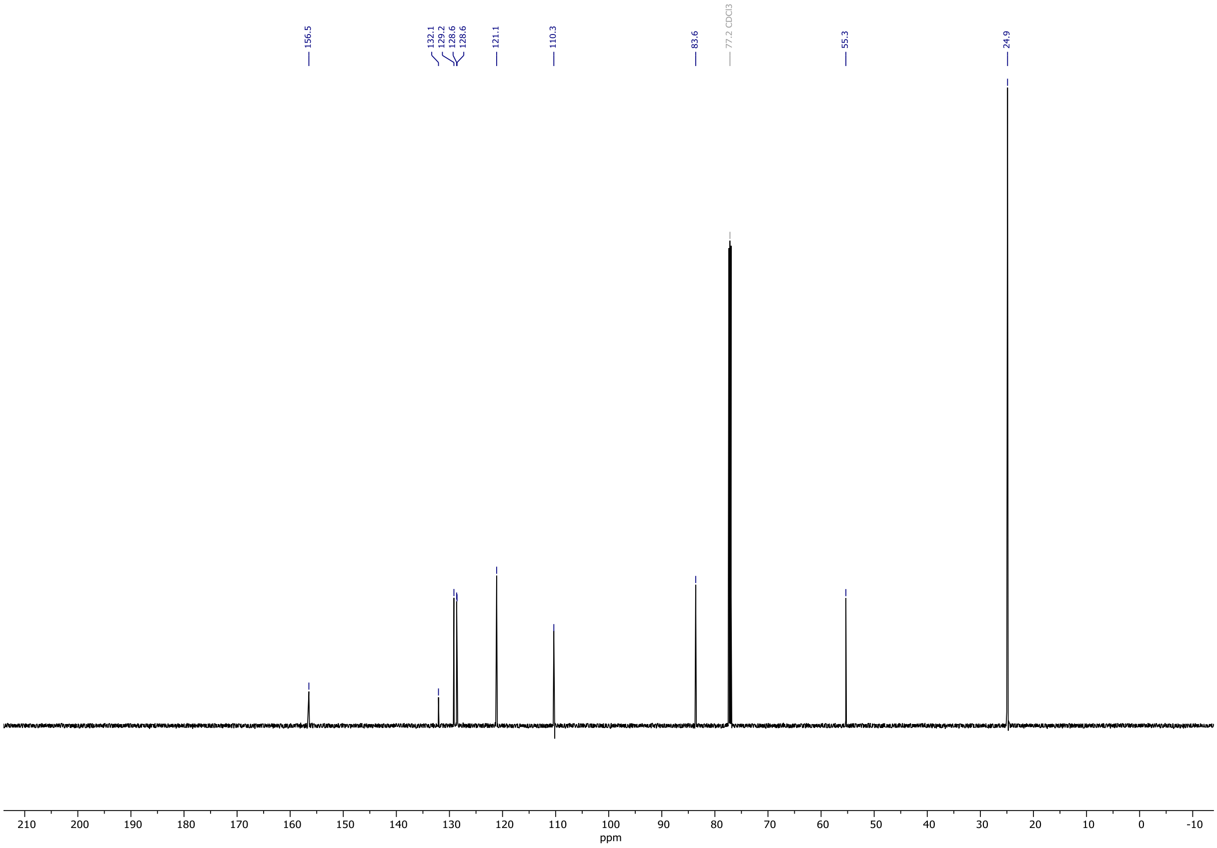

^{1}H NMR ($CDCl_3$, 599 MHz) for **1d**

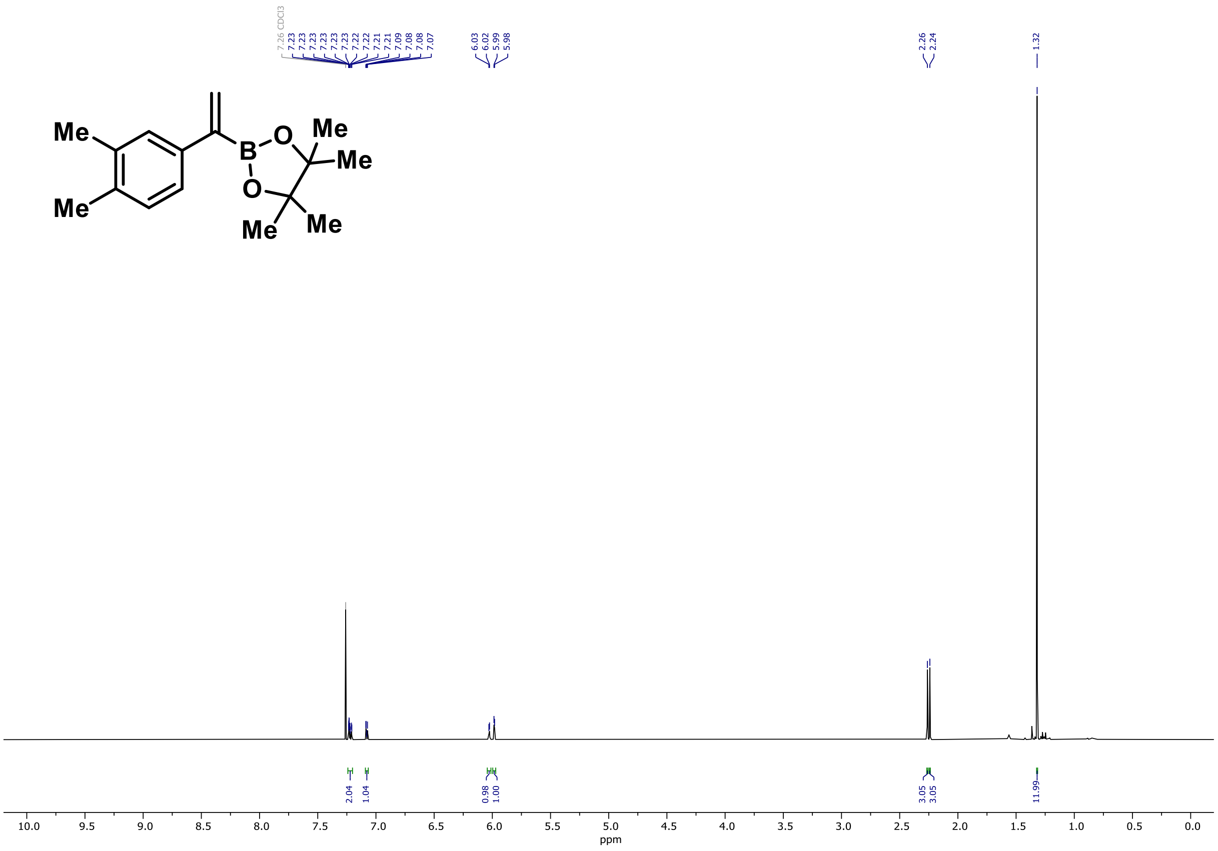

^{13}C{^{1}H} NMR ($CDCl_3$, 151 MHz) for **1d**

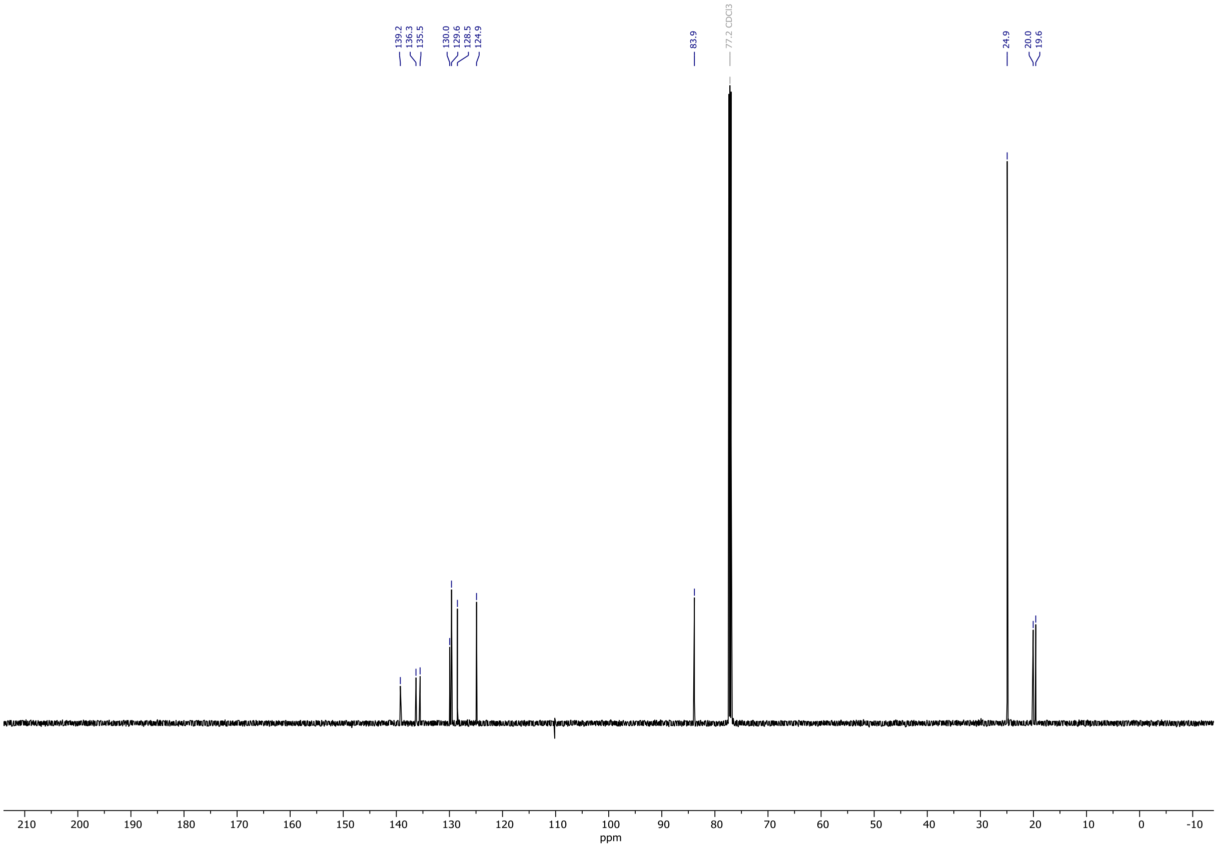

^{1}H NMR ($CDCl_3$, 400 MHz) for **1e**

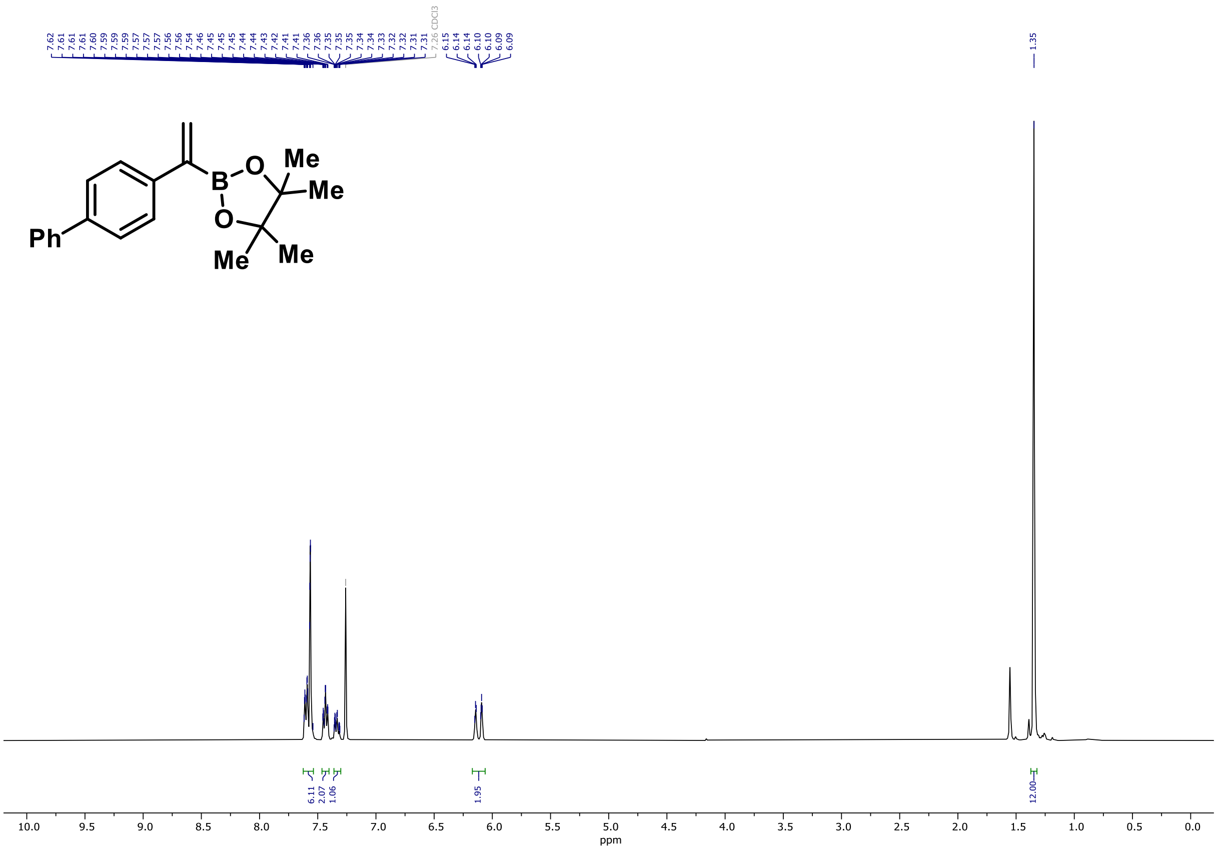

^{13}C{^{1}H} NMR ($CDCl_3$, 101 MHz) for **1e**

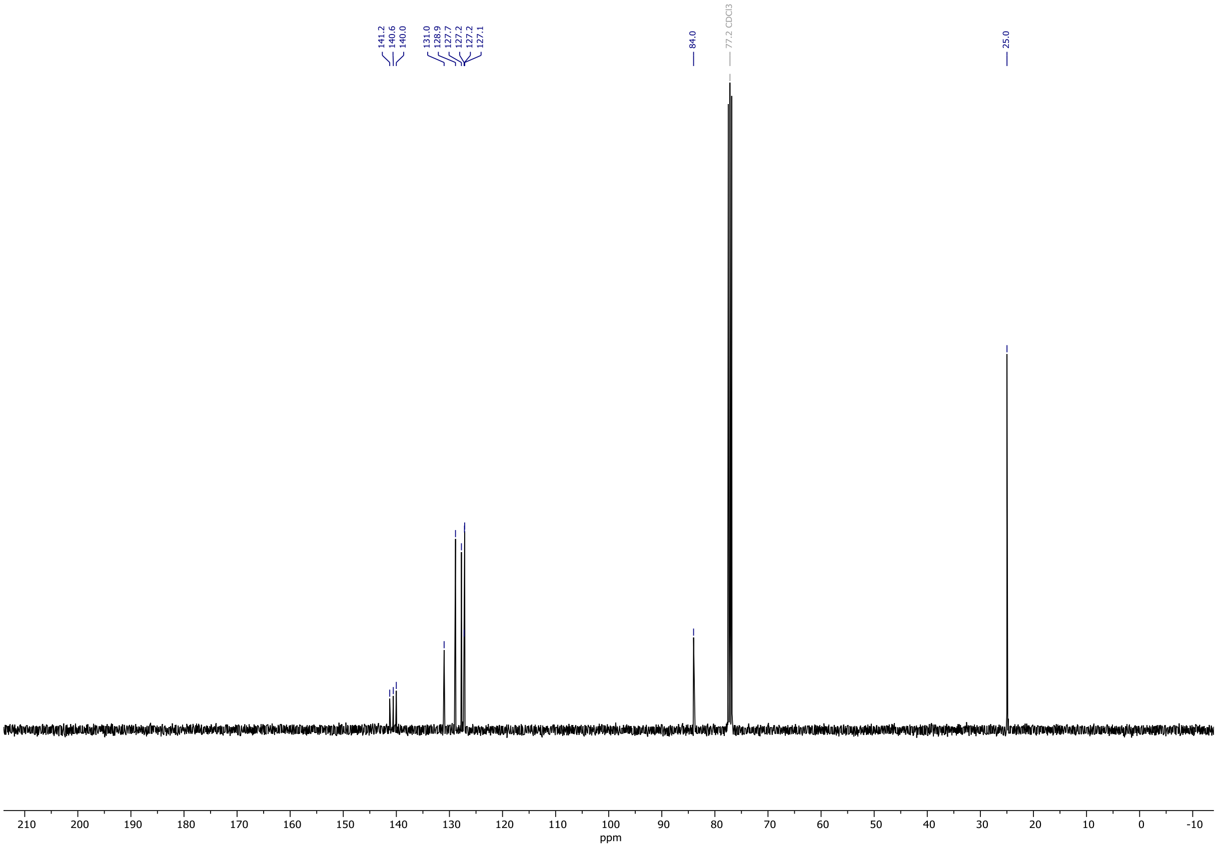

^{1}H NMR ($CDCl_3$, 599 MHz) for **1f**

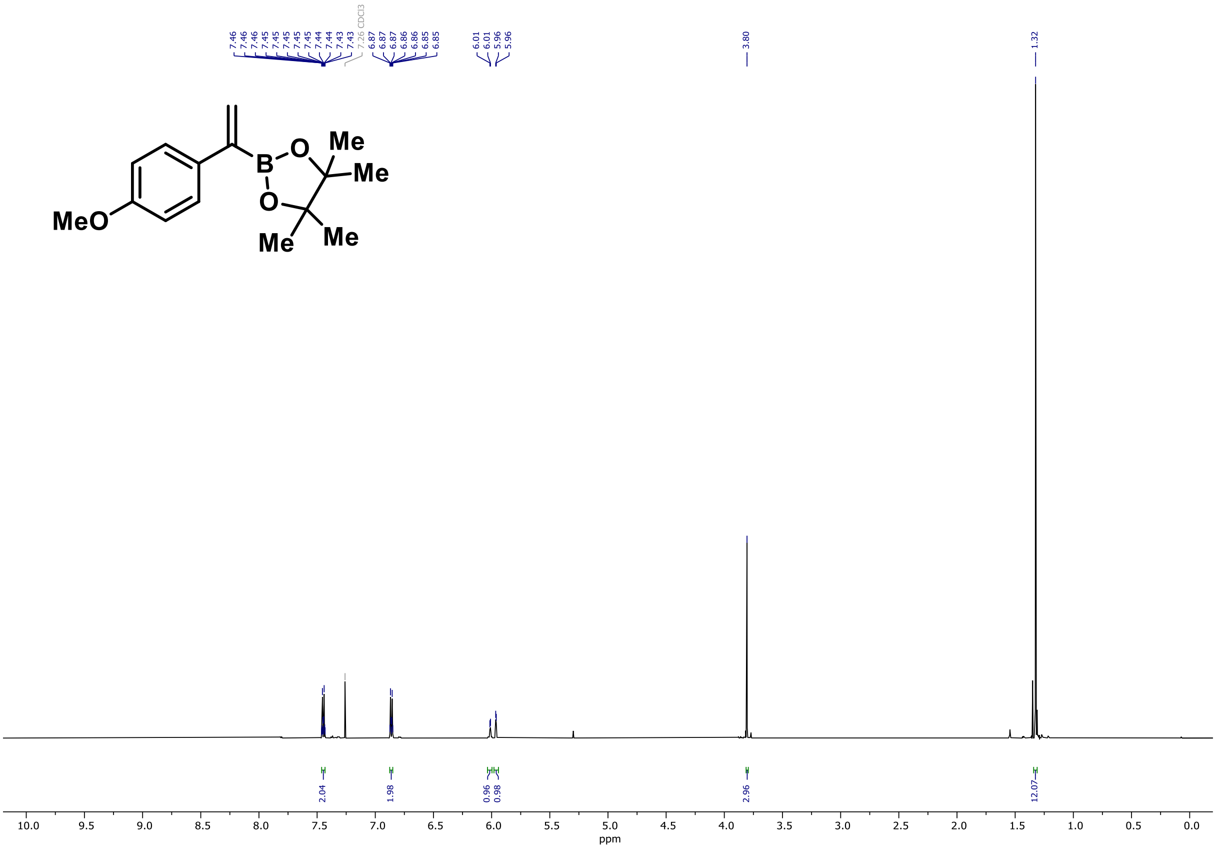

$^{13}C\{^{1}H\}$ NMR ($CDCl_3$, 151 MHz) for **1f**

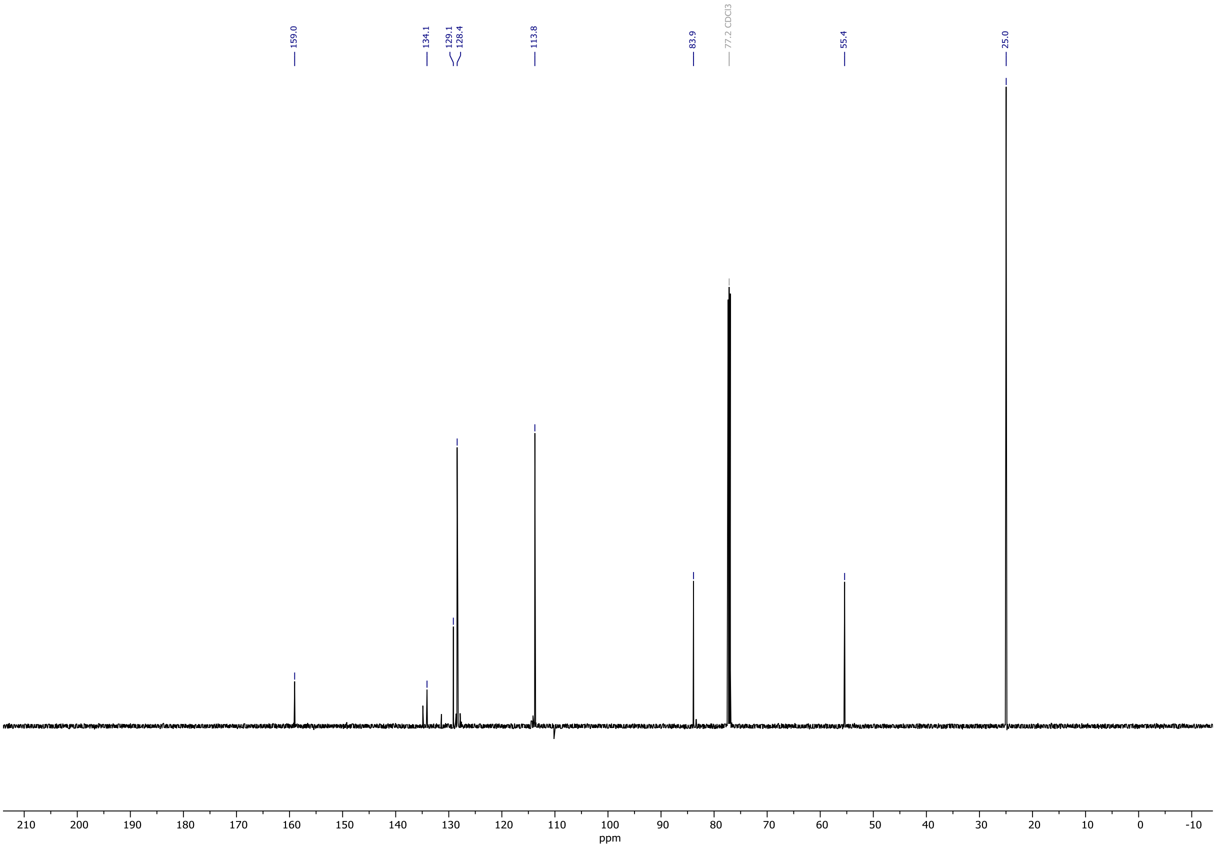

^{1}H NMR (CDCl$_3$, 400 MHz) for **1g**

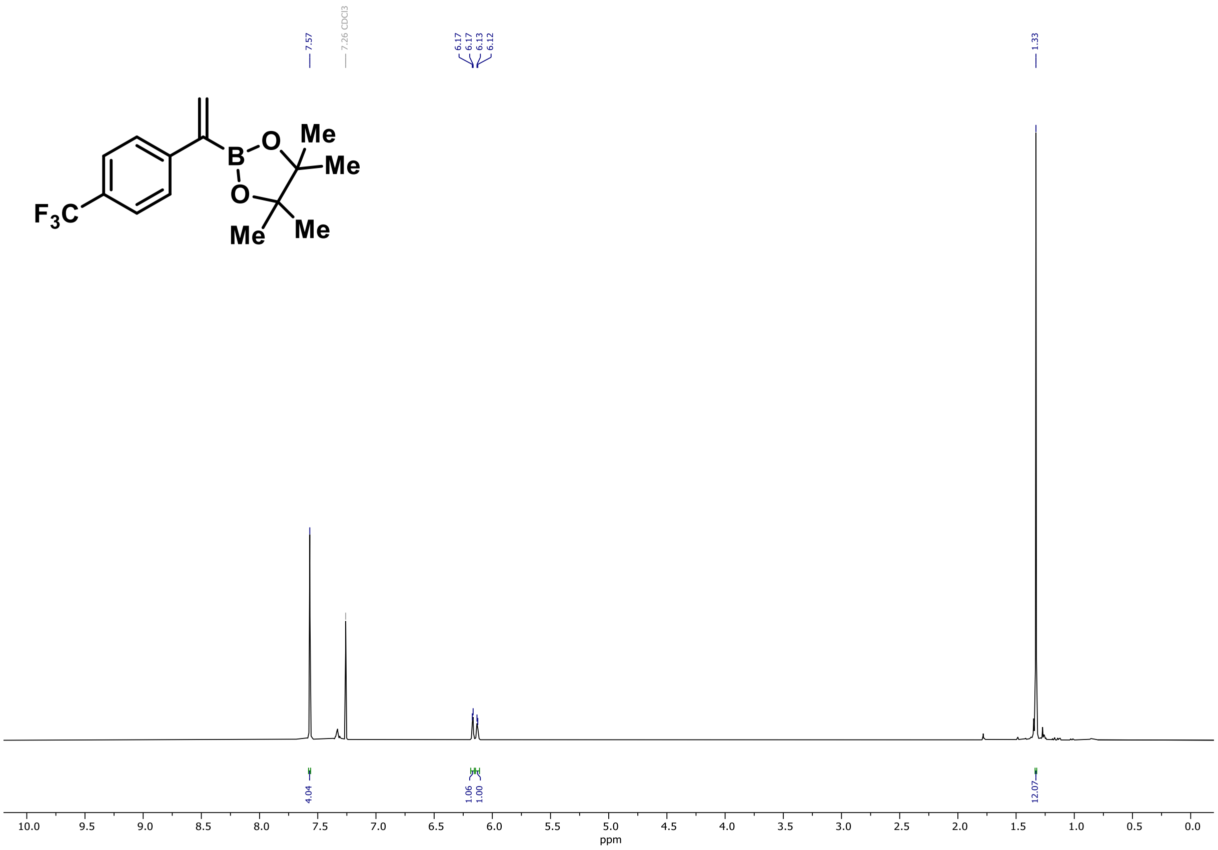

^{13}C{^{1}H} NMR (CDCl$_3$, 126 MHz) for **1g**

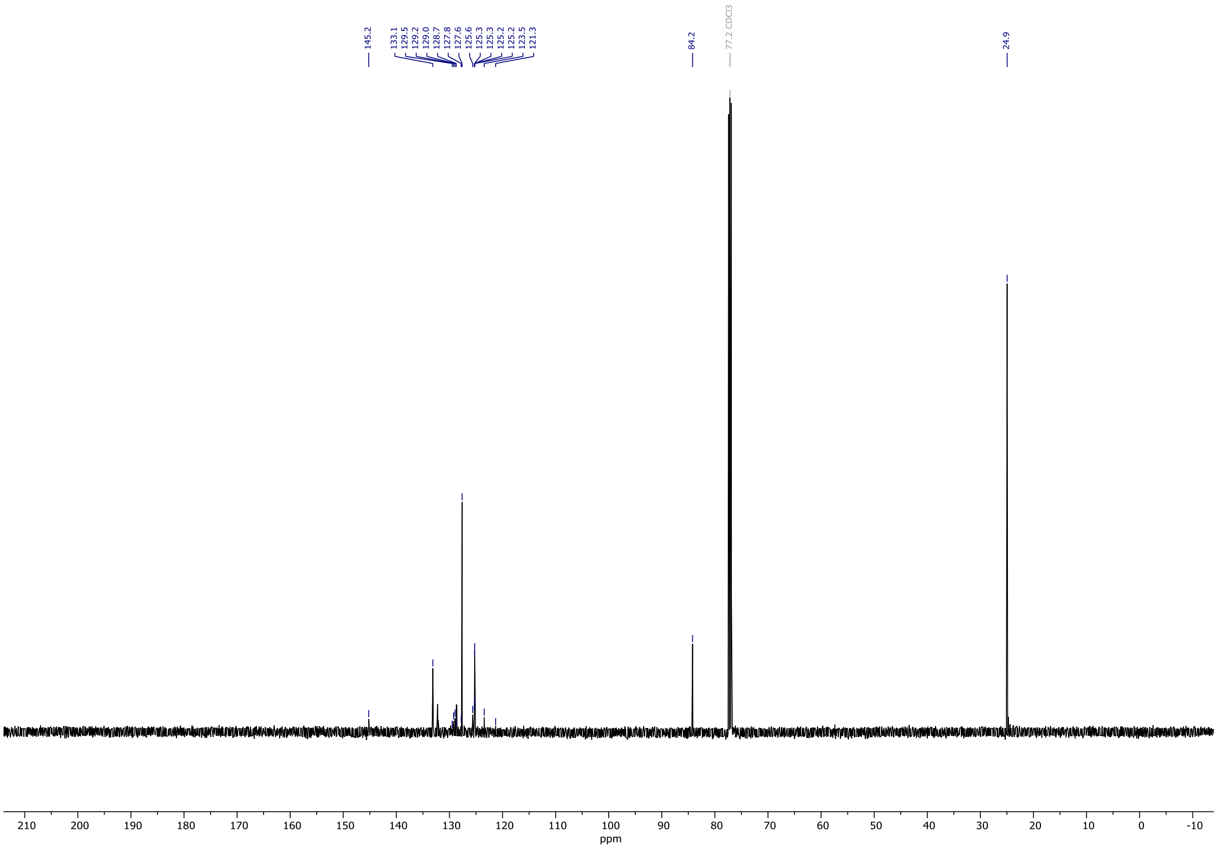

^{19}F{^{1}H} NMR (CDCl$_3$, 376 MHz) for **1g**

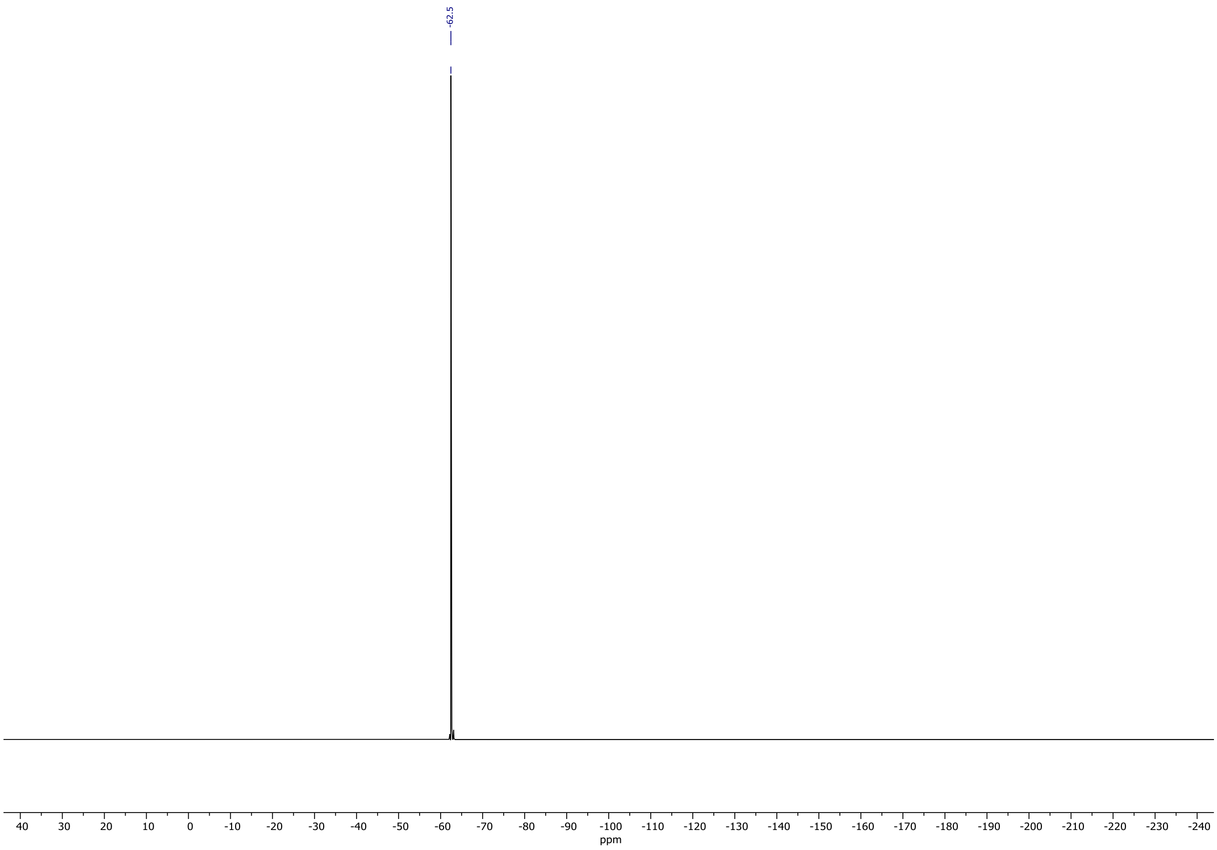

^{1}H NMR (CDCl$_3$, 599 MHz) for **1h**

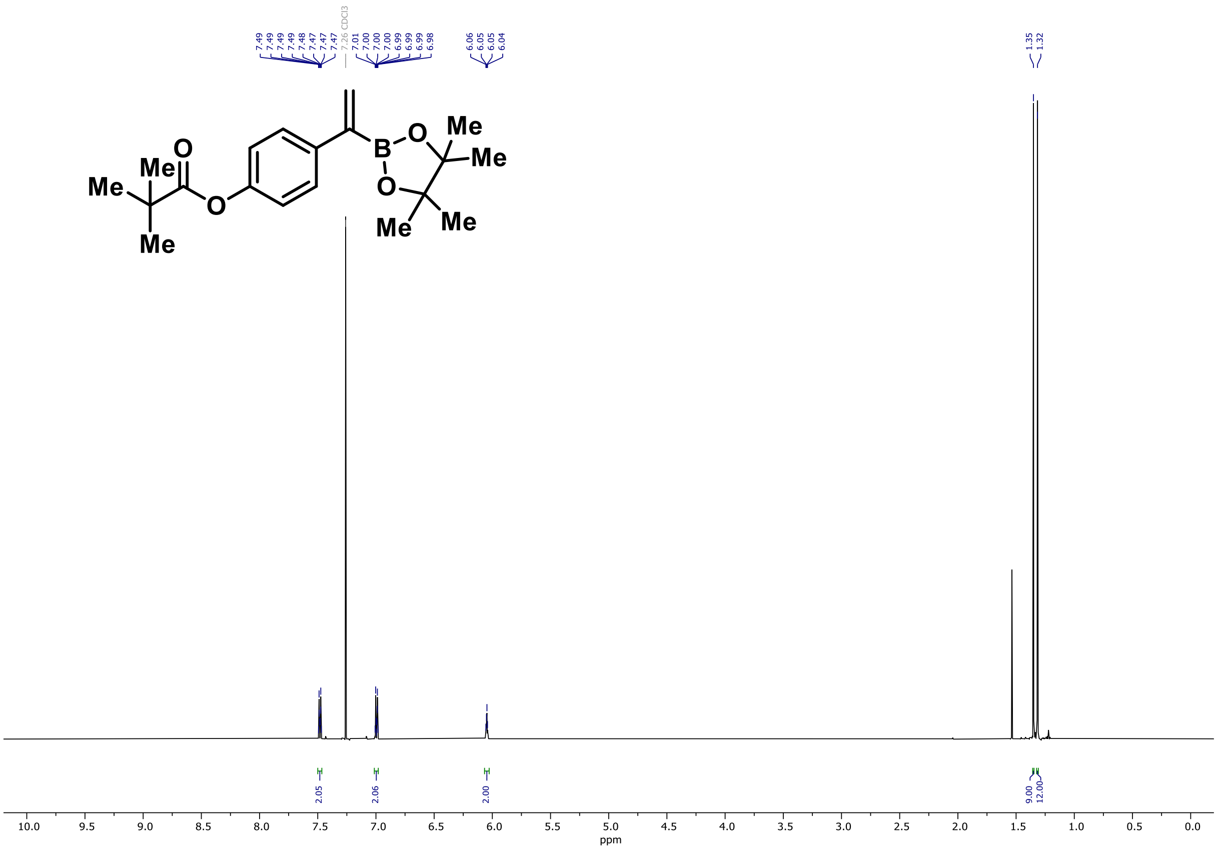

^{13}C{^{1}H} NMR (CDCl$_3$, 151 MHz) for **1h**

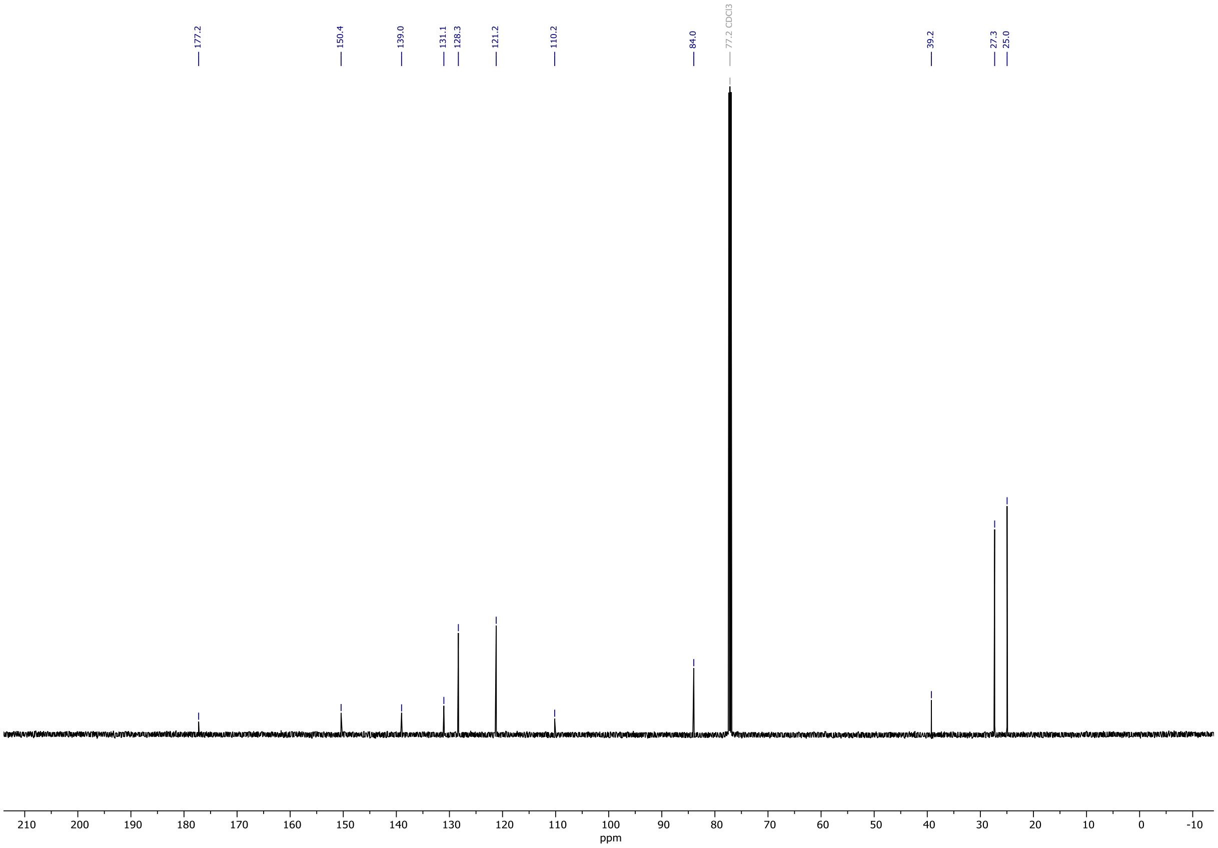

^{1}H NMR ($CDCl_3$, 599 MHz) for **1i**

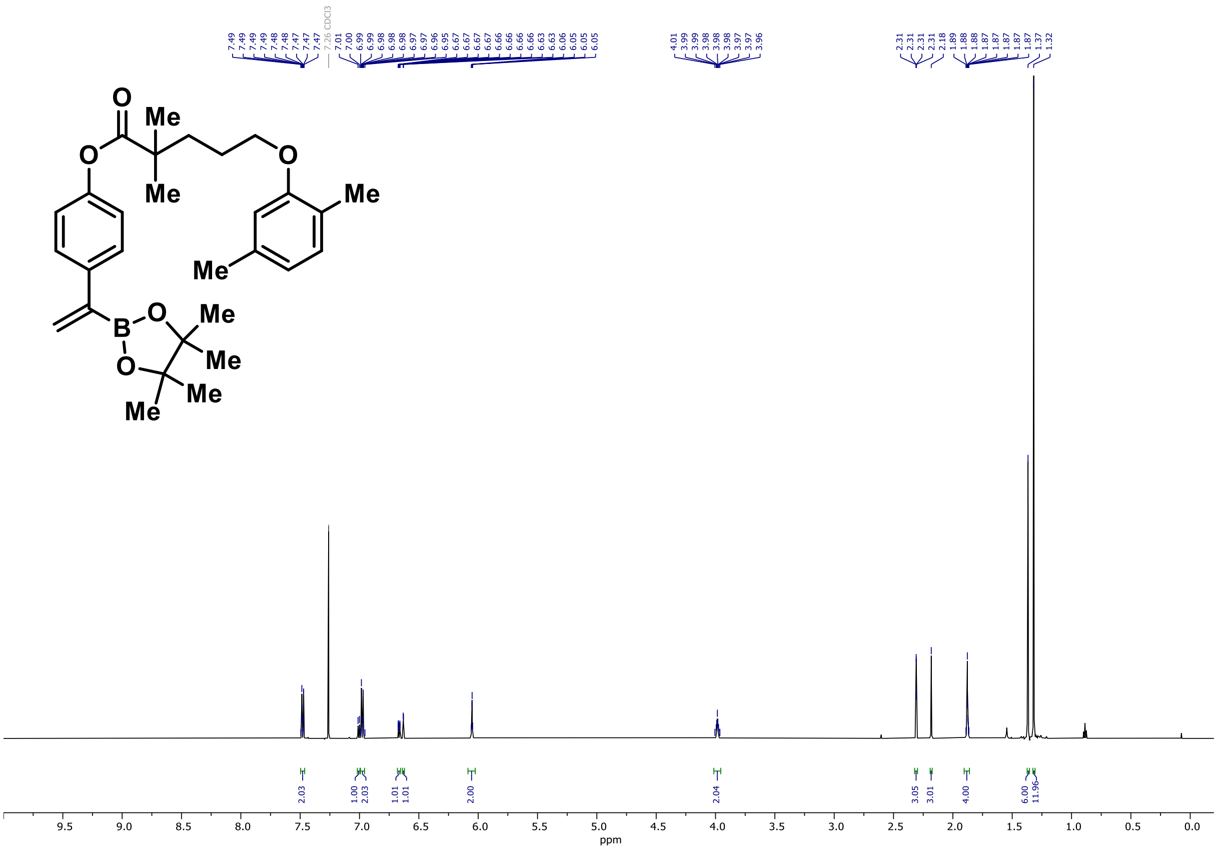

^{13}C{^{1}H} NMR ($CDCl_3$, 151 MHz) for **1i**

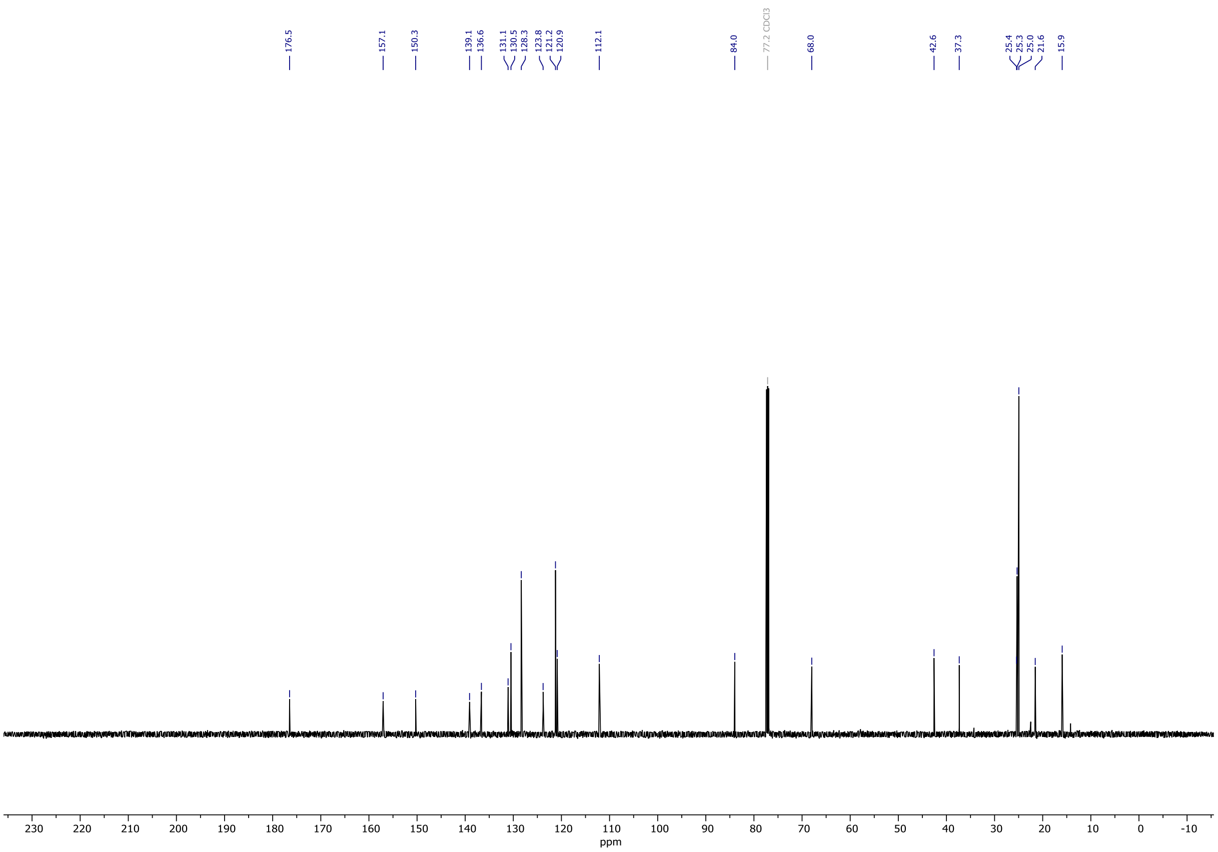

^{1}H NMR ($CDCl_3$, 400 MHz) for **1j**

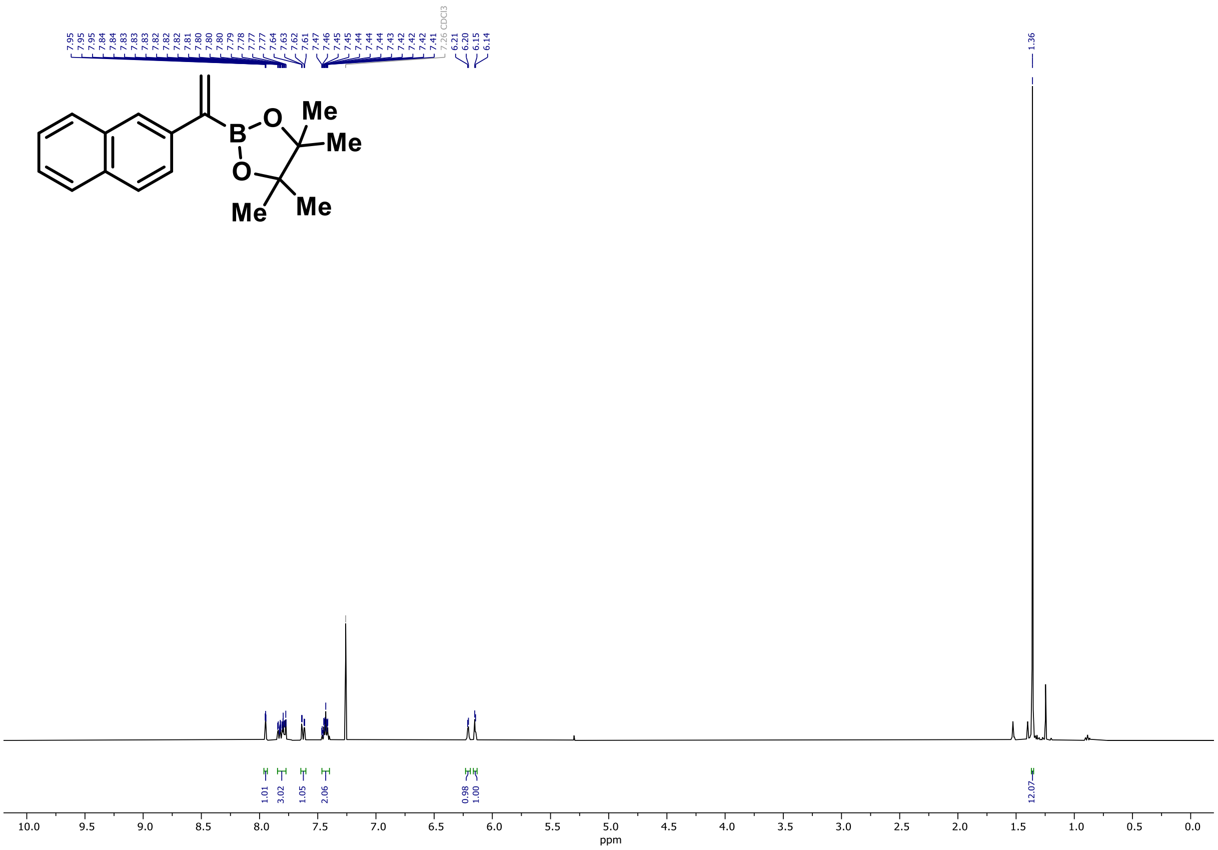

^{13}C{^{1}H} NMR ($CDCl_3$, 151 MHz) for **1j**

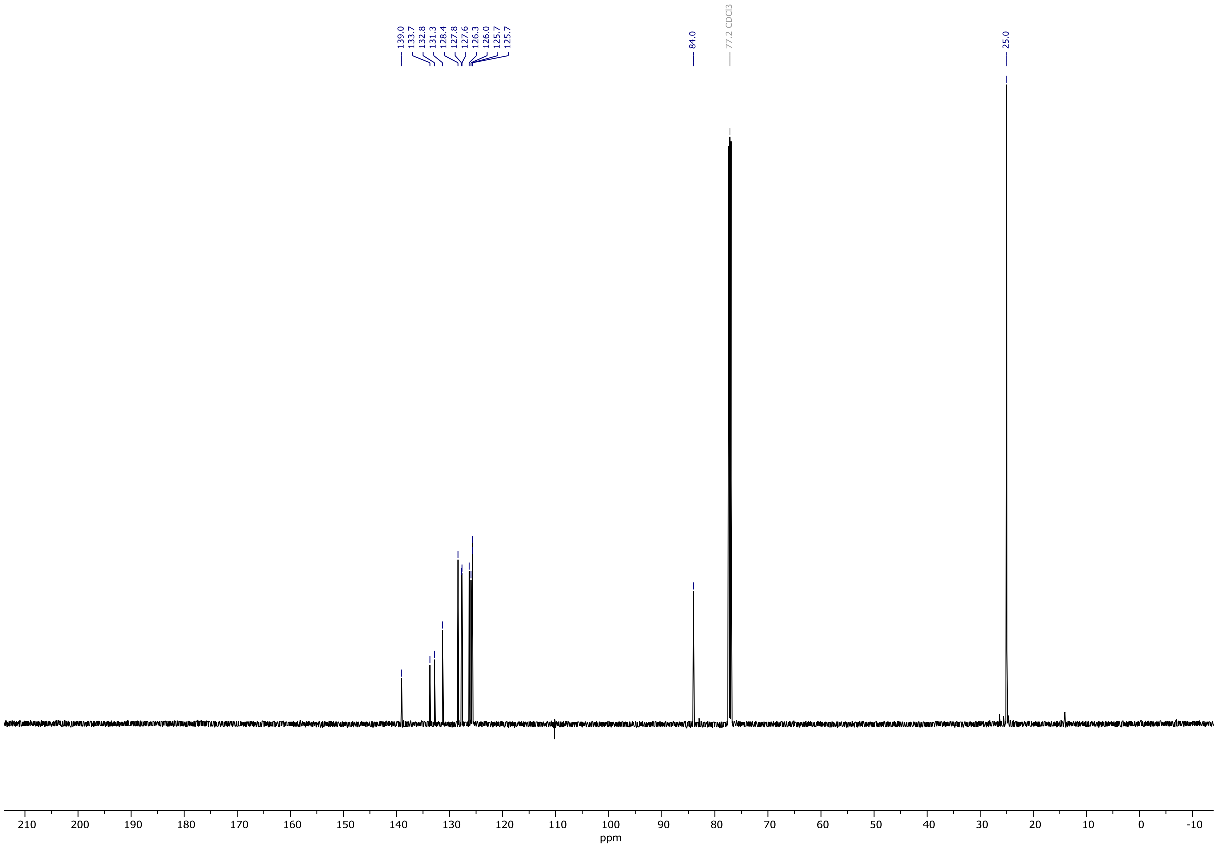

^{1}**H NMR** ($CDCl_3$, 400 MHz) for **1k**

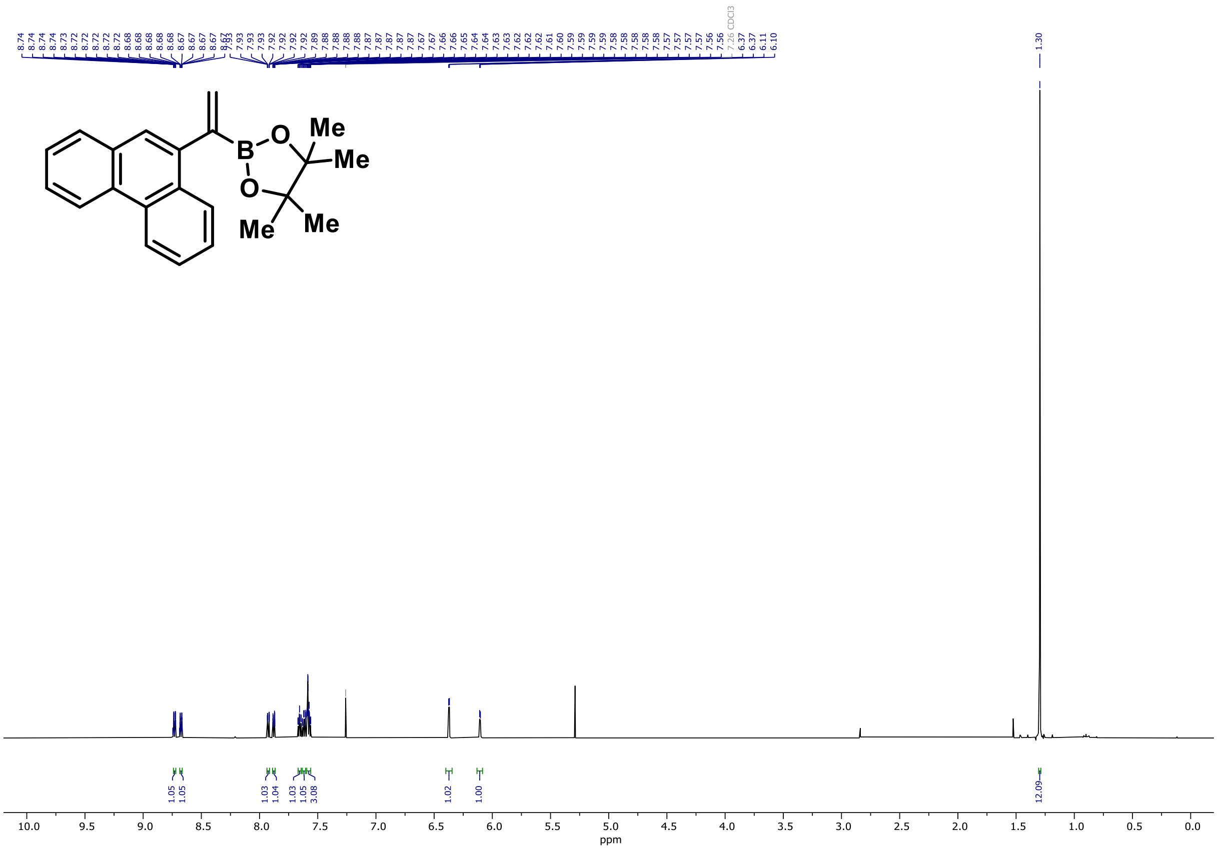

^{13}**C{^{1}H} NMR** ($CDCl_3$, 101 MHz) for **1k**

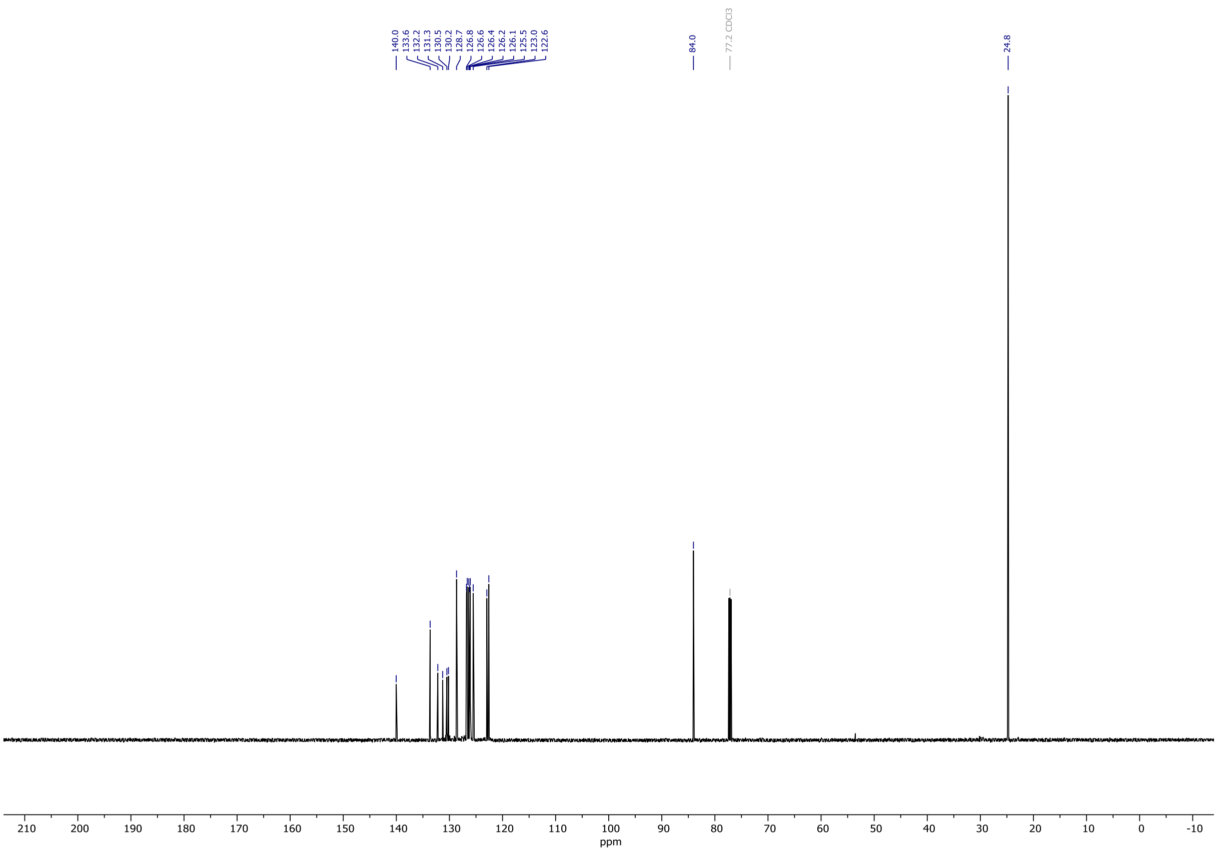

^{1}H NMR ($CDCl_3$, 400 MHz) for **2d**

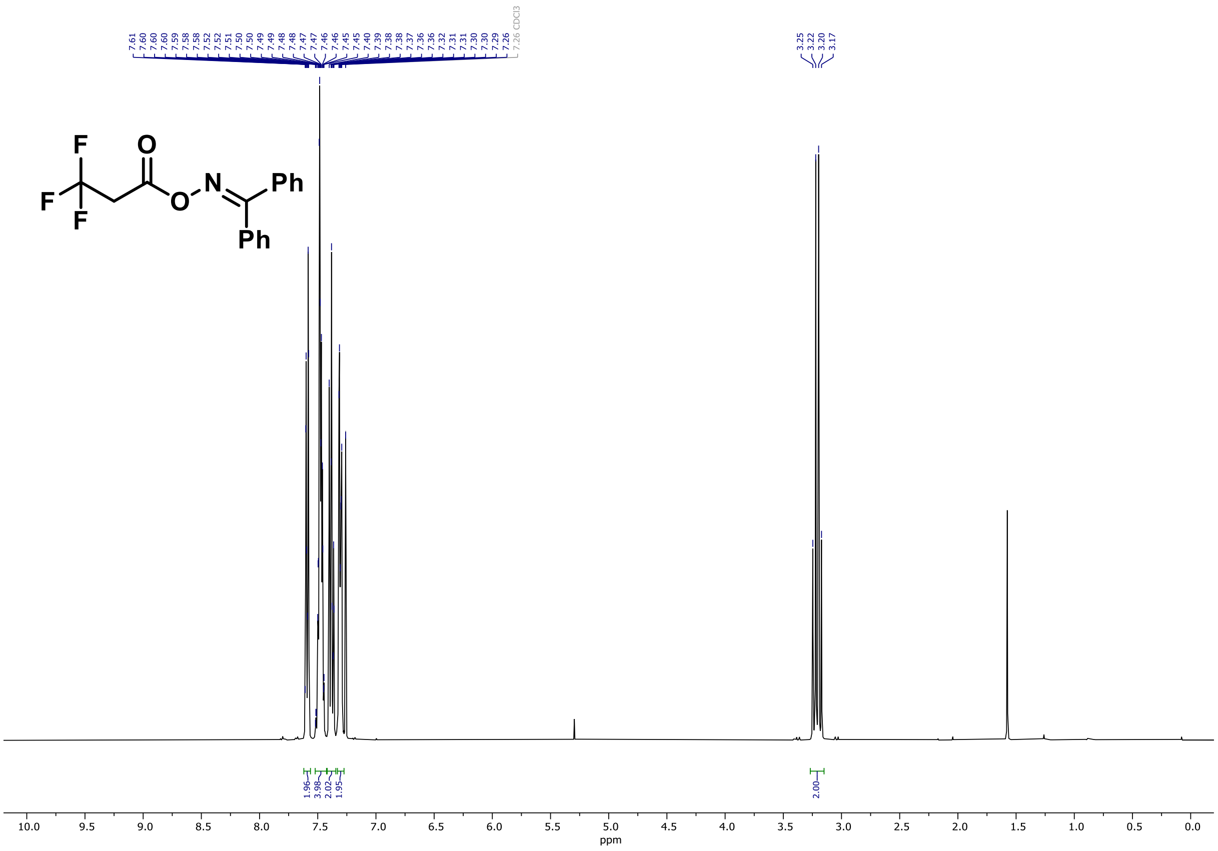

^{13}C{^{1}H} NMR ($CDCl_3$, 101 MHz) for **2d**

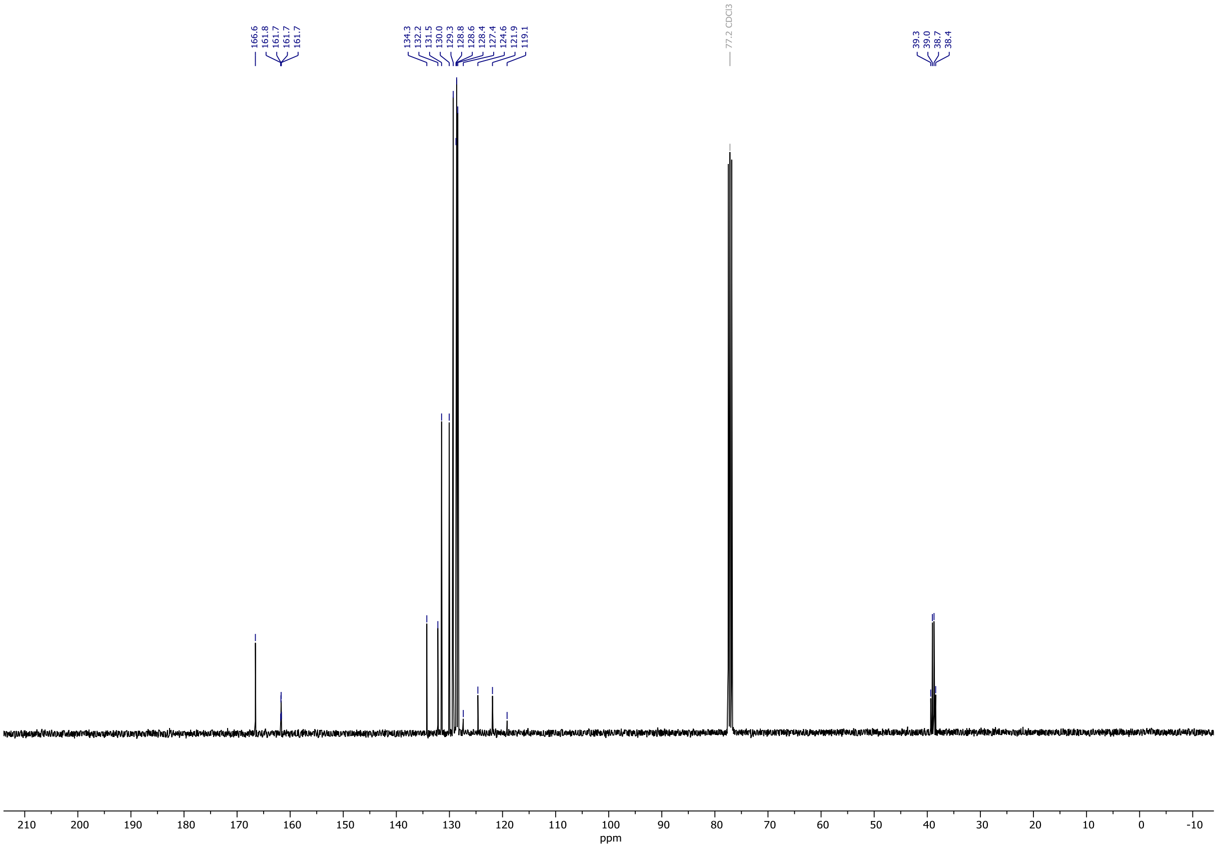

^{19}F{^{1}H} NMR (CDCl$_3$, 376 MHz) for **2d**

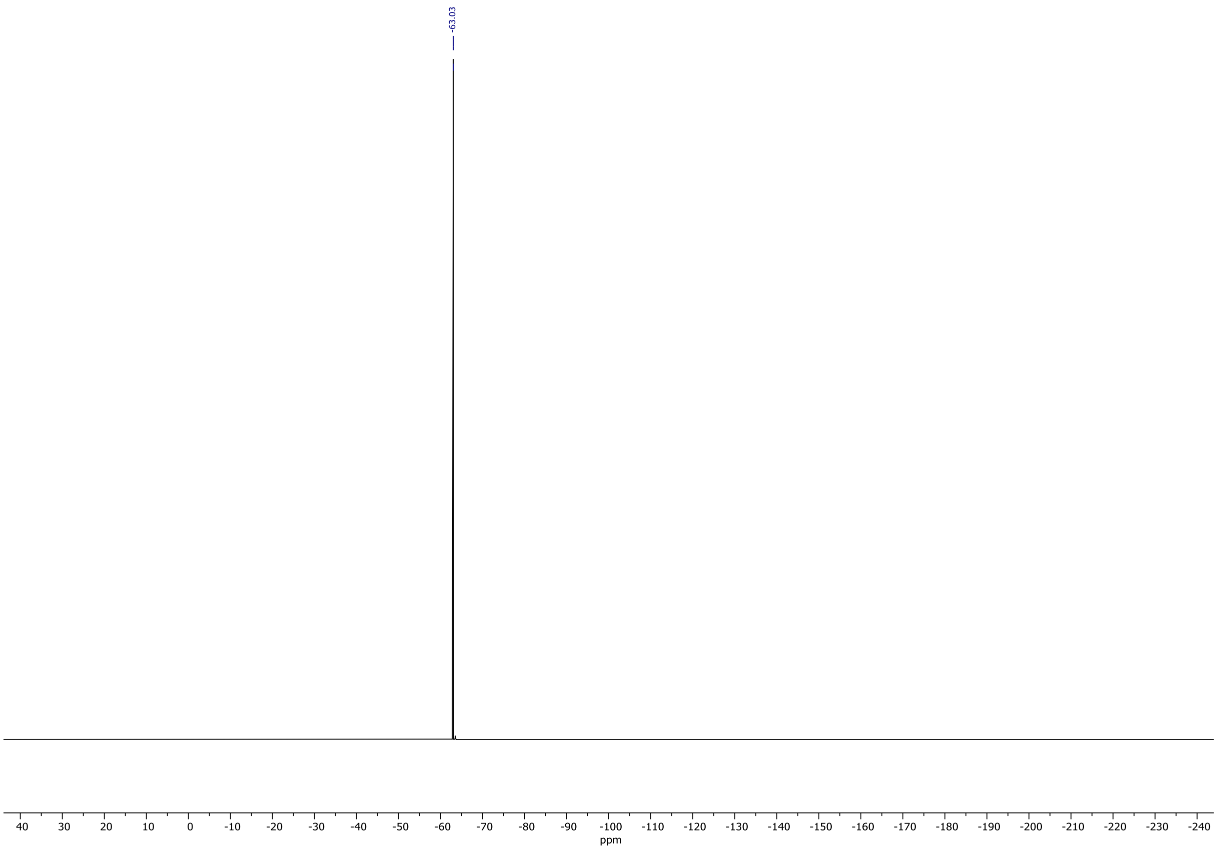

^{1}H NMR (CDCl$_3$, 400 MHz) for **2e**

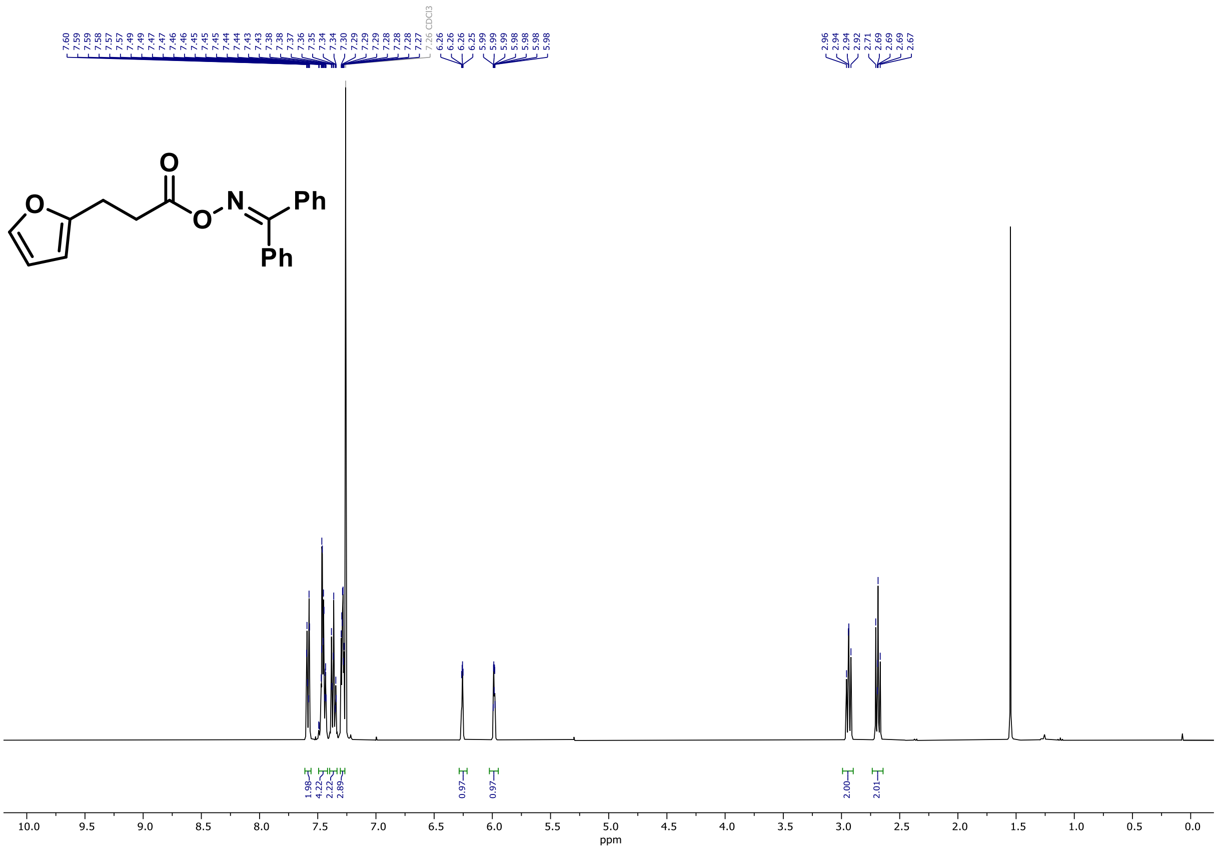

^{13}C{^{1}H} NMR (CDCl$_3$, 101 MHz) for **2e**

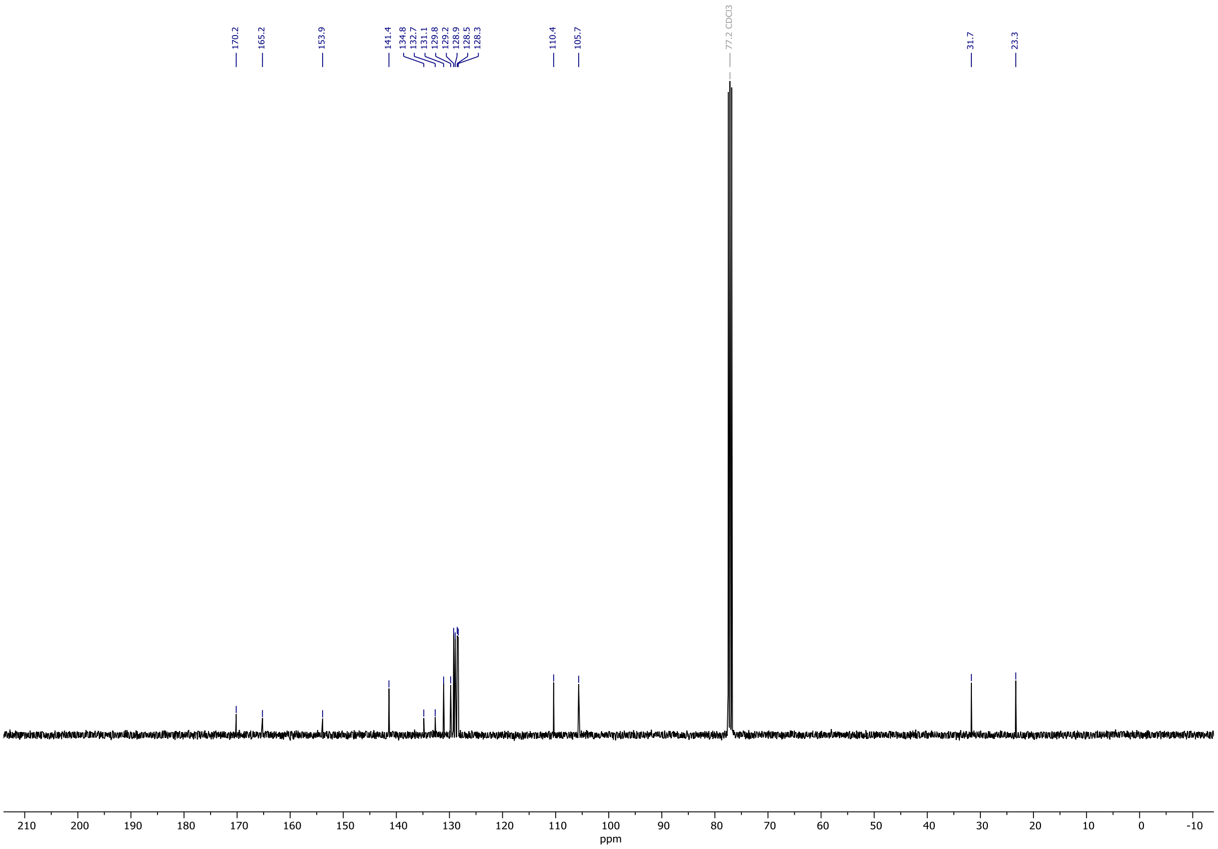

¹H NMR (CDCl$_3$, 400 MHz) for **2f**

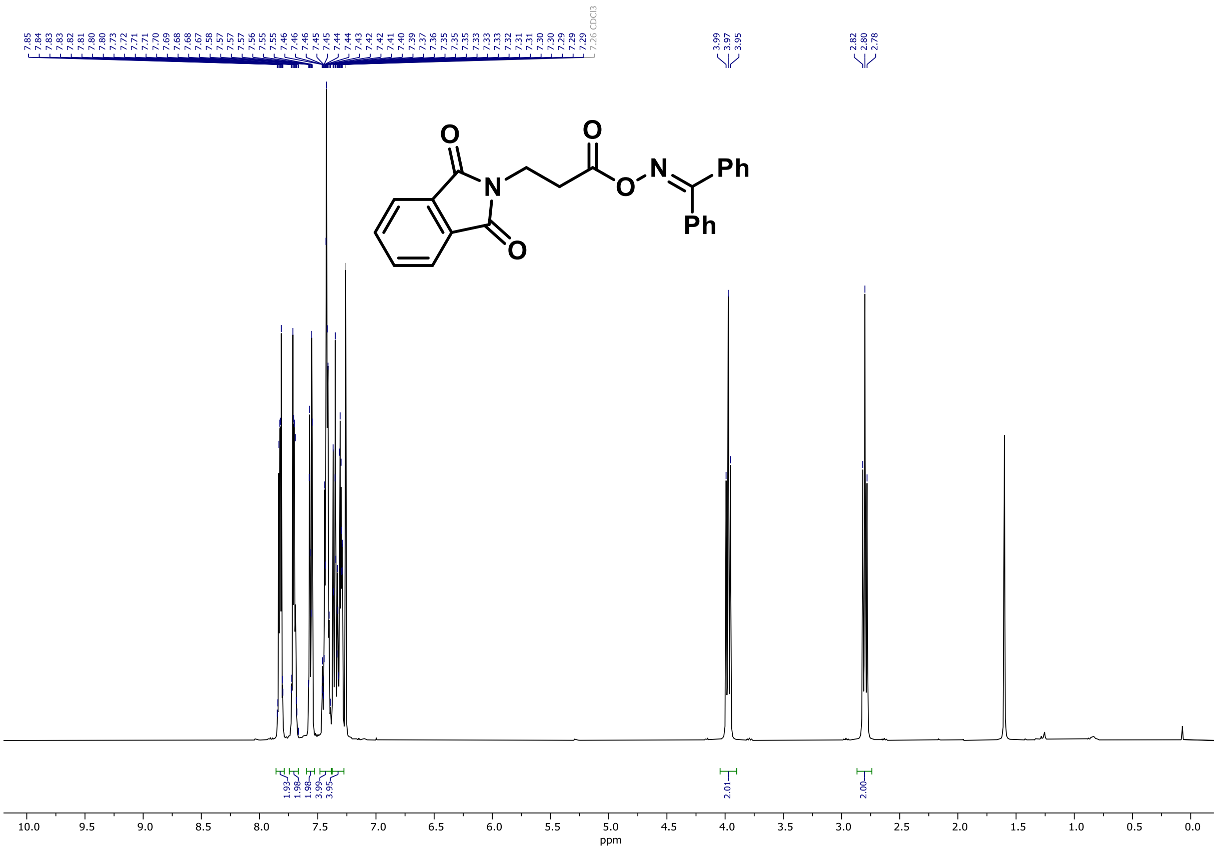

¹³C{¹H} NMR (CDCl$_3$, 101 MHz) for **2f**

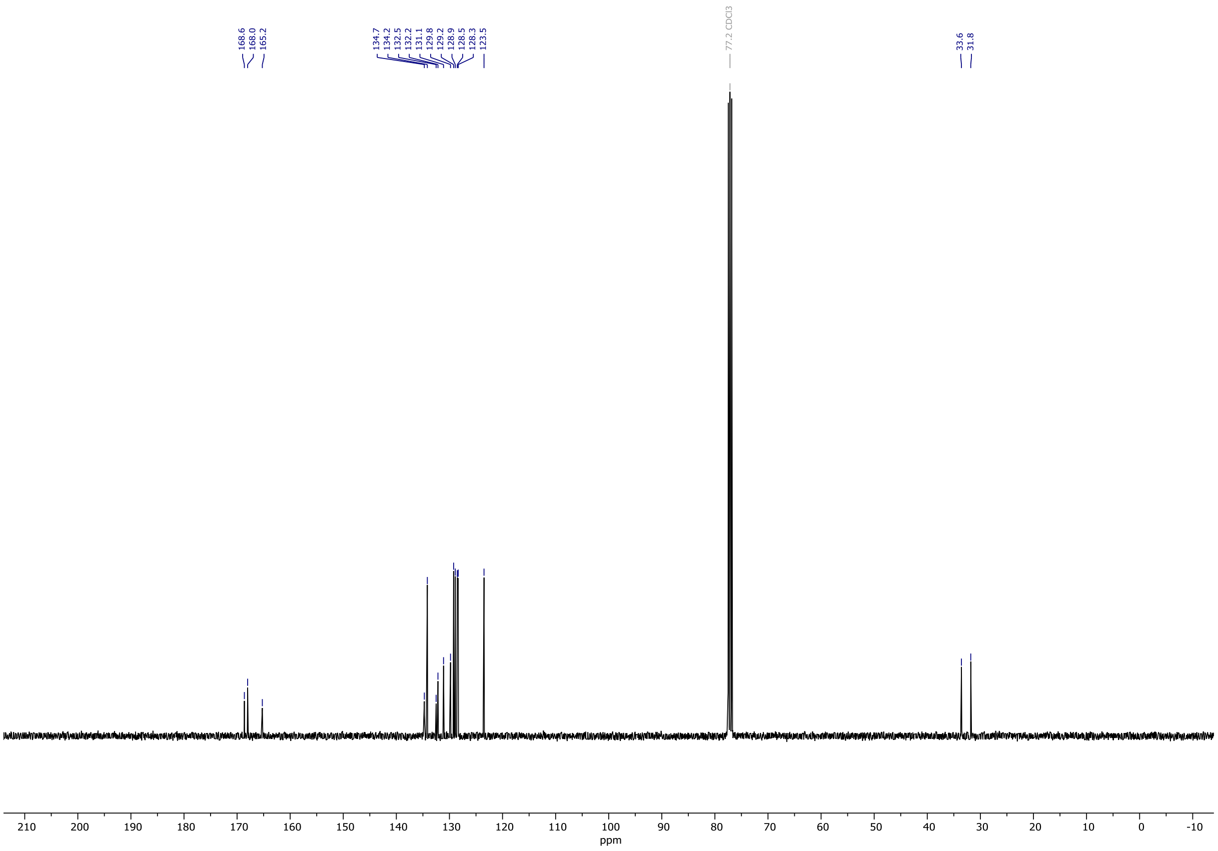

^{1}H NMR (CDCl$_3$, 400 MHz) for **2h**

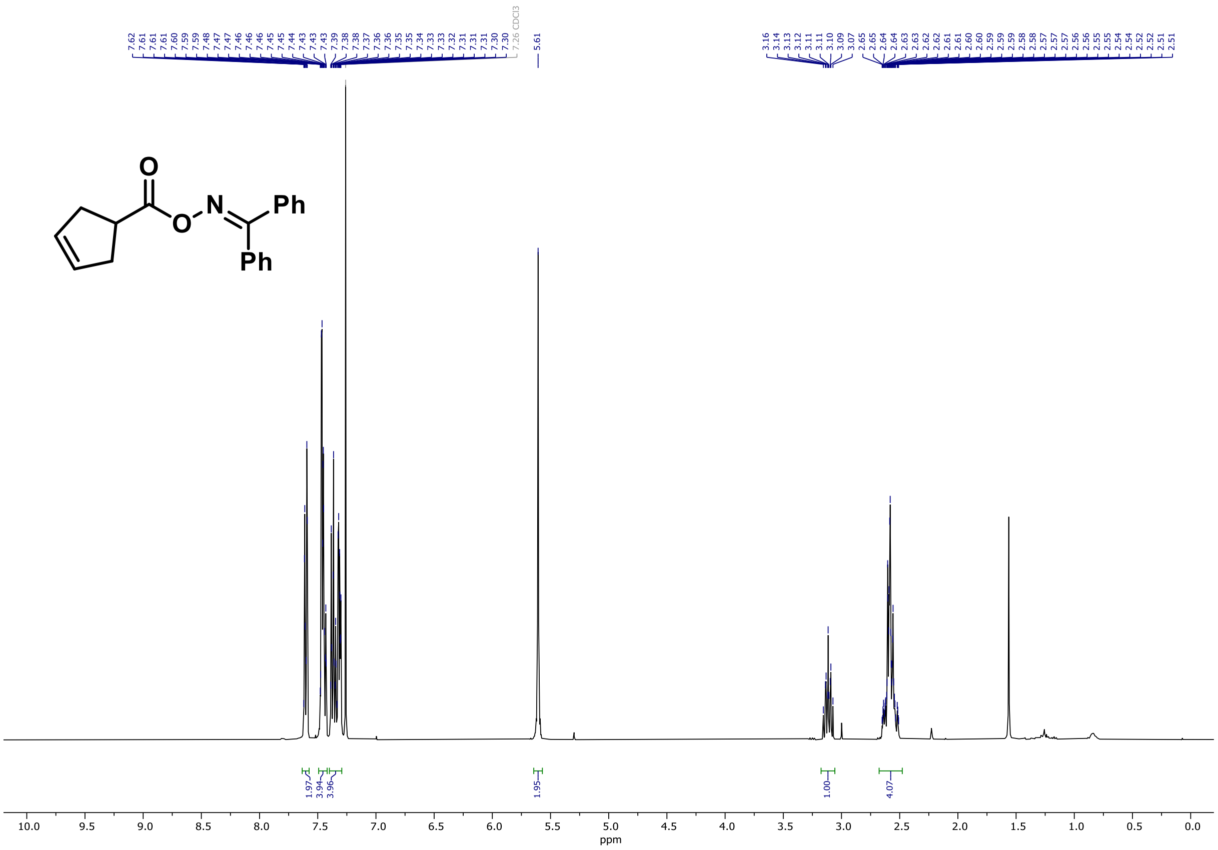

^{13}C{^{1}H} NMR (CDCl$_3$, 101 MHz) for **2h**

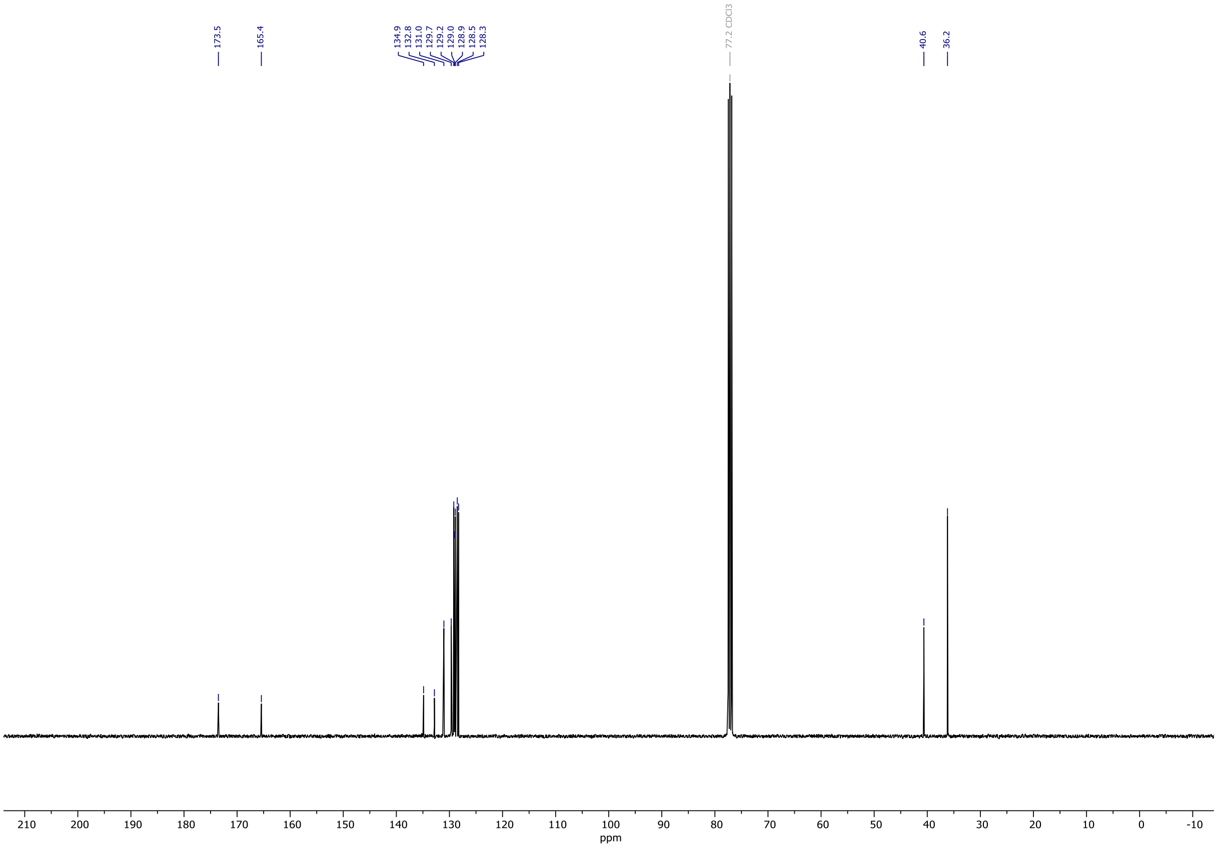

¹H NMR (CDCl$_3$, 400 MHz) for **2u**

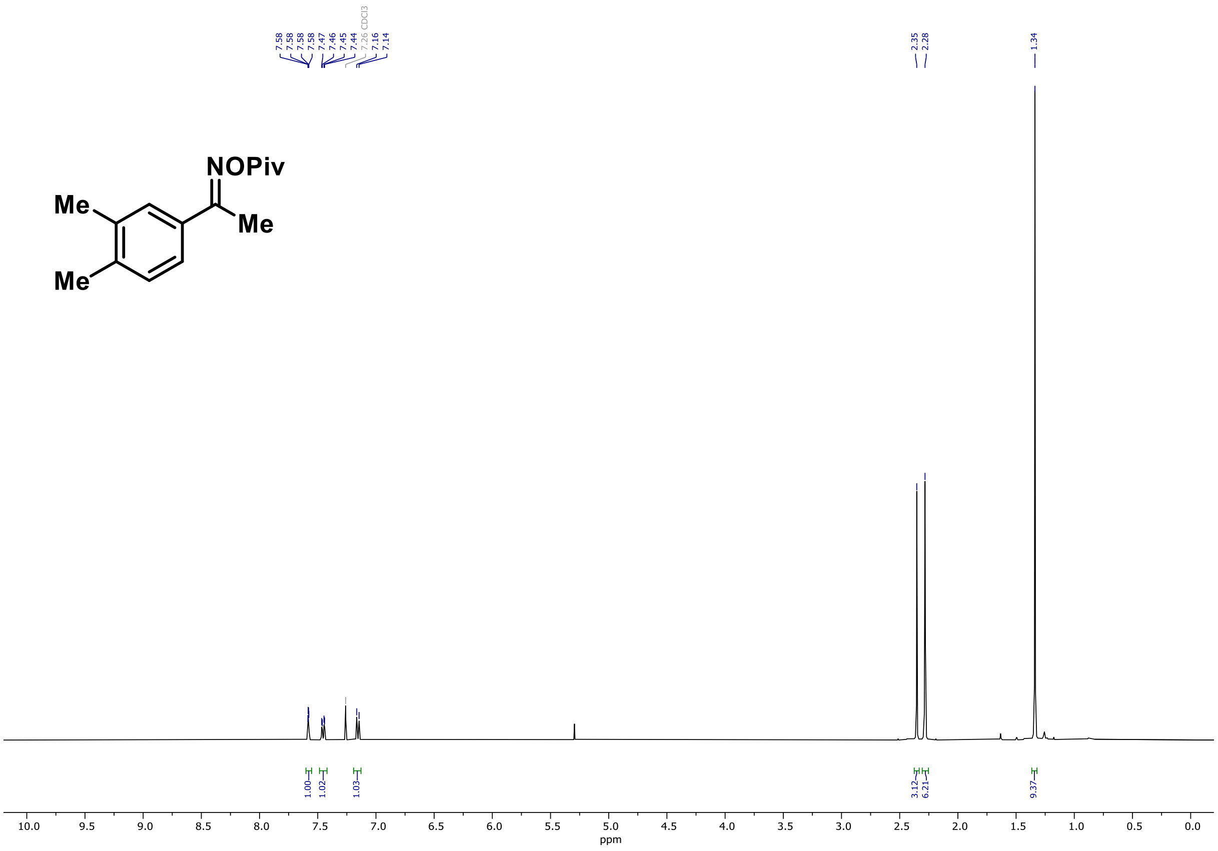

¹³C{¹H} NMR (CDCl$_3$, 101 MHz) for **2u**

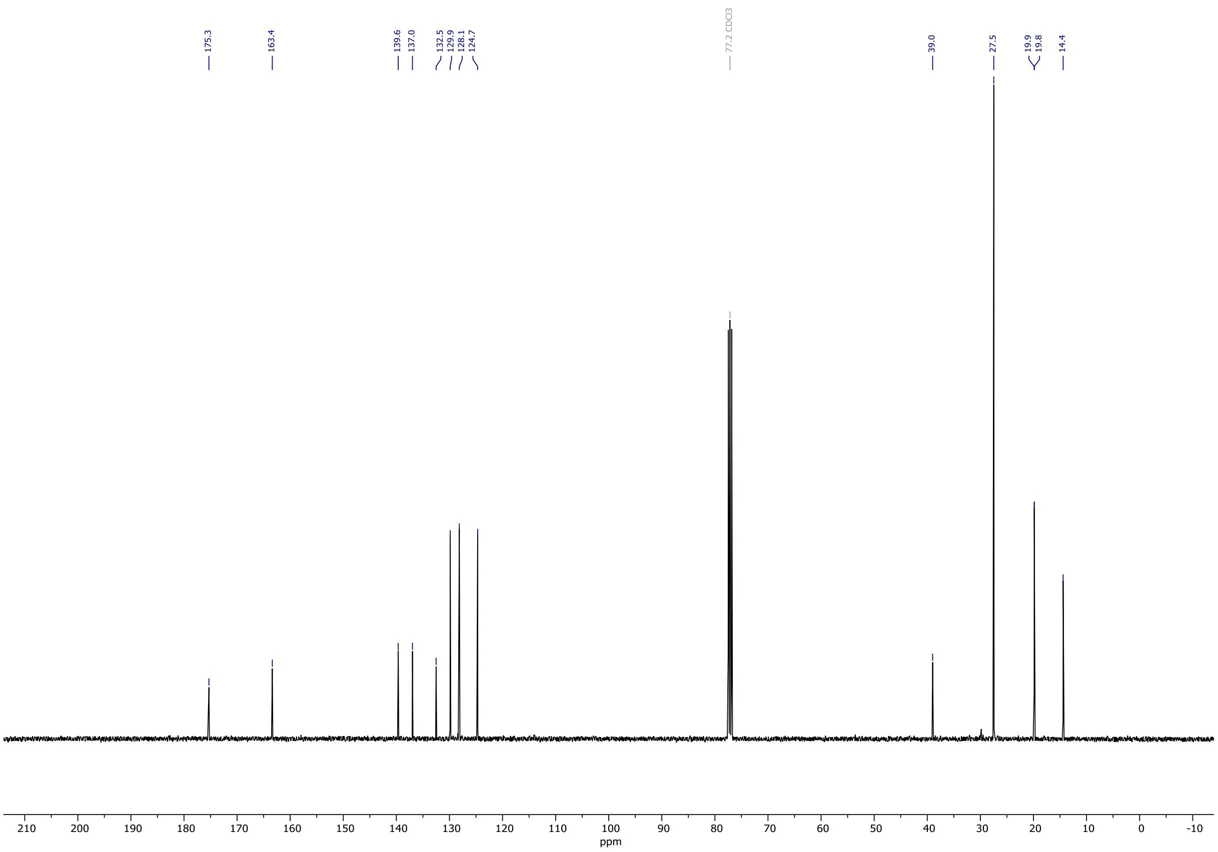

¹H NMR (CDCl$_3$, 400 MHz) for **2v**

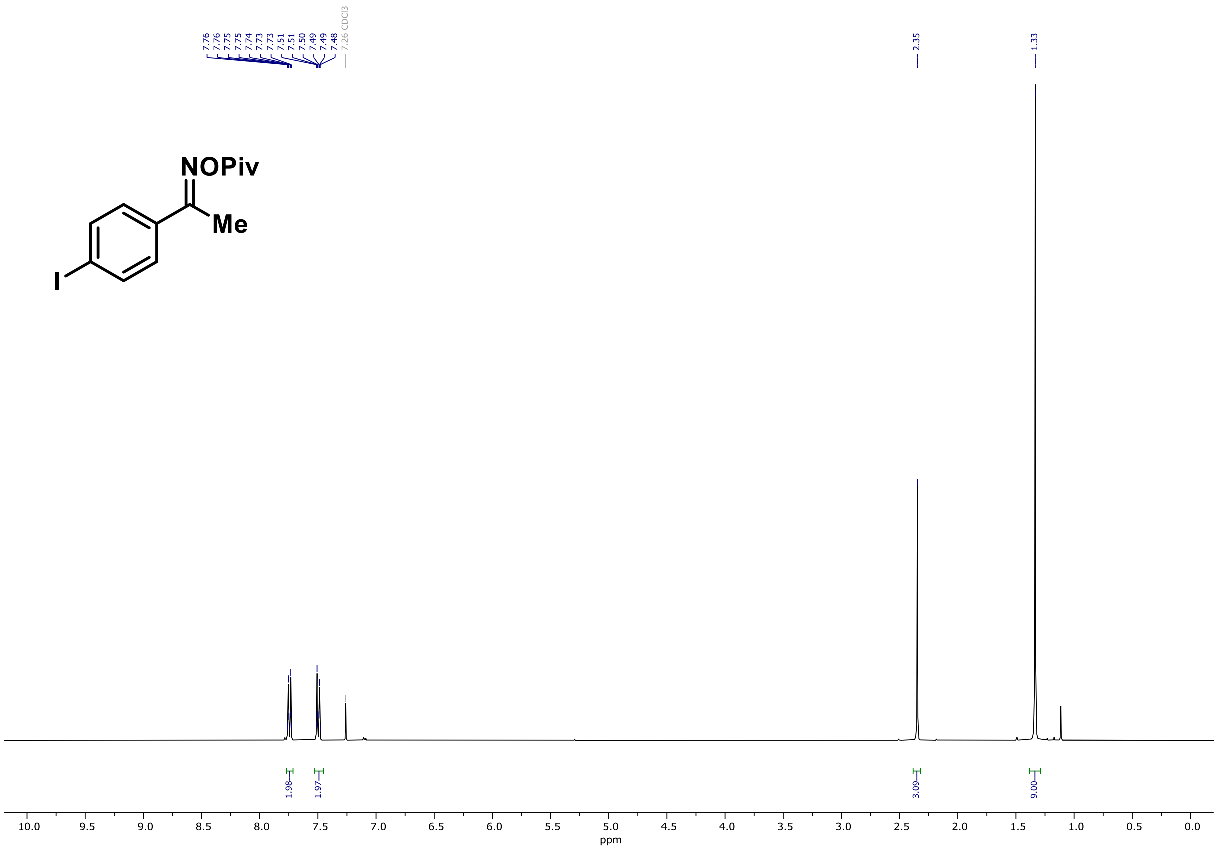

¹³C{¹H} NMR (CDCl$_3$, 101 MHz) for **2v**

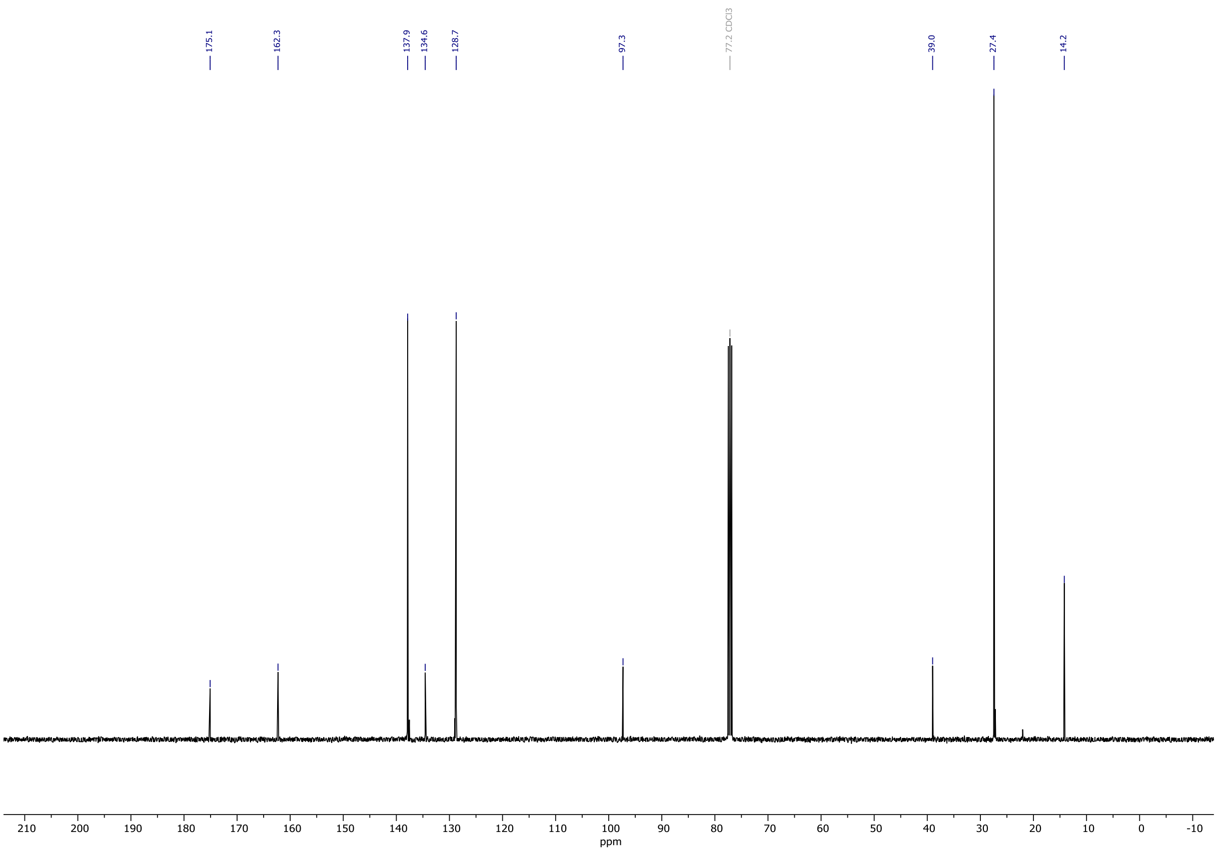

^{1}H NMR ($CDCl_3$, 400 MHz) for **2w**

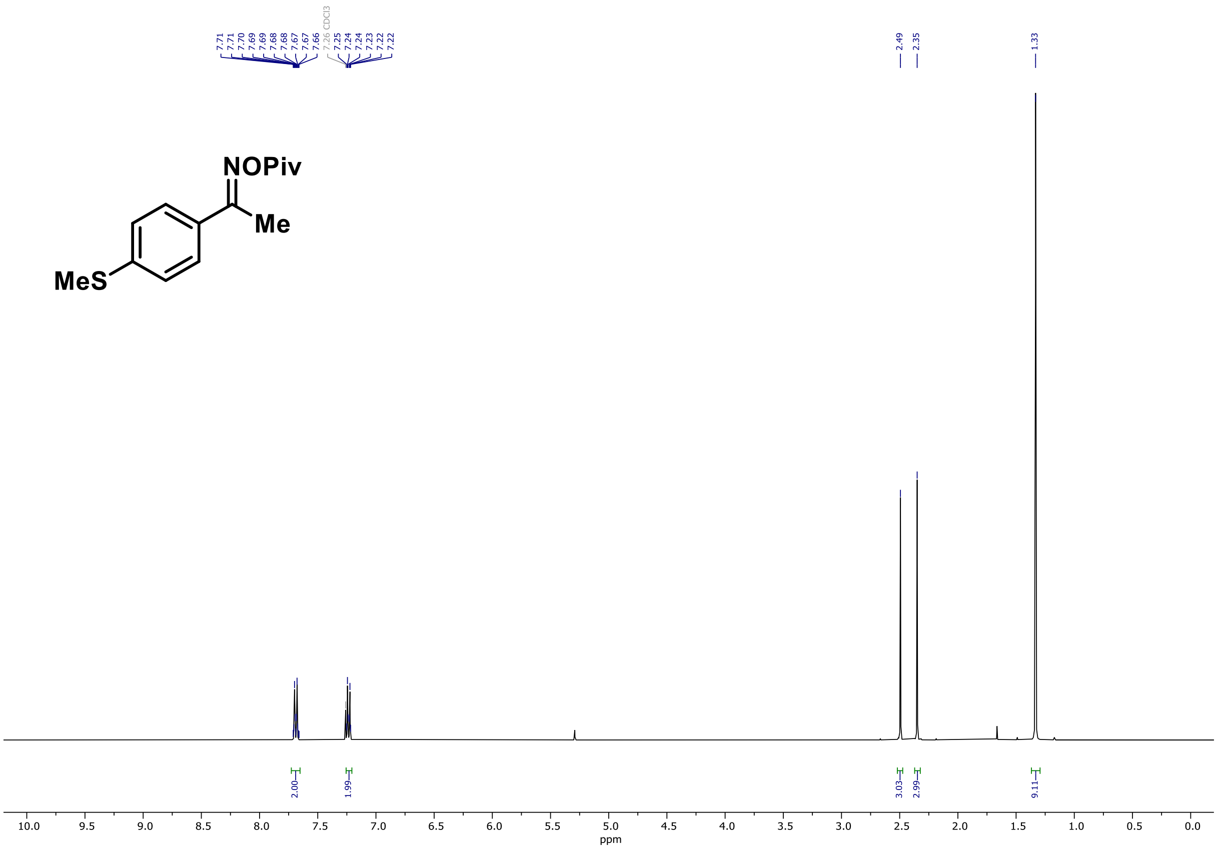

^{13}C{^{1}H} NMR ($CDCl_3$, 101 MHz) for **2w**

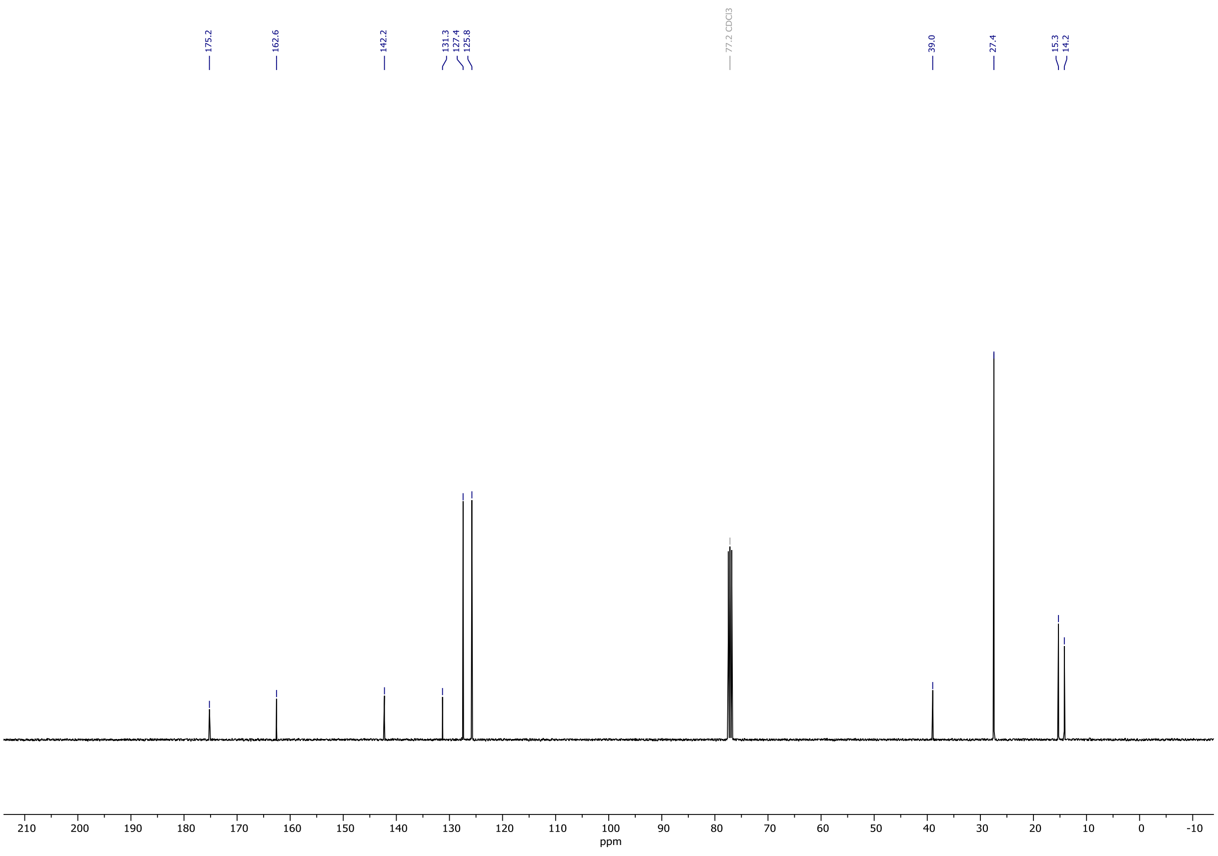

^{1}H NMR ($CDCl_3$, 400 MHz) for **2x**

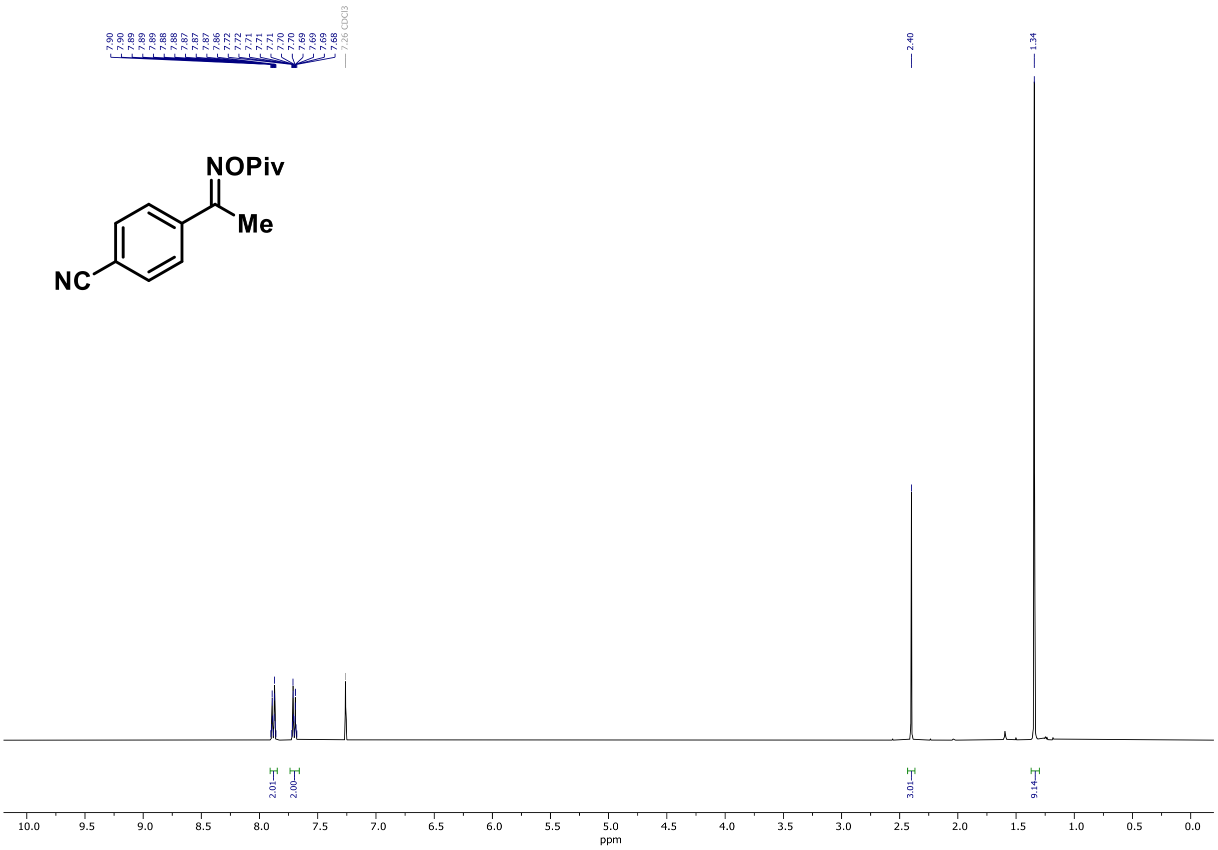

^{13}C{^{1}H} NMR ($CDCl_3$, 101 MHz) for **2x**

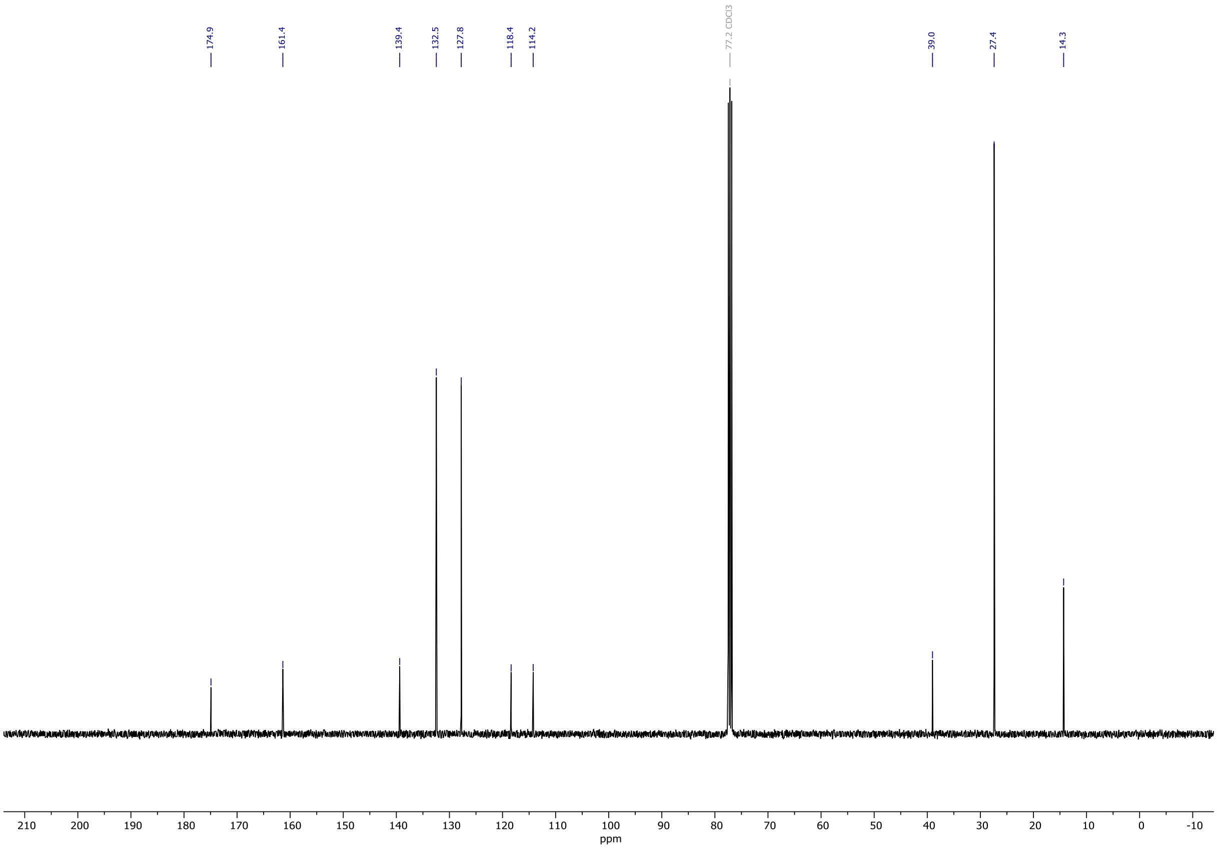

^{1}H NMR ($CDCl_3$, 400 MHz) for **2y**

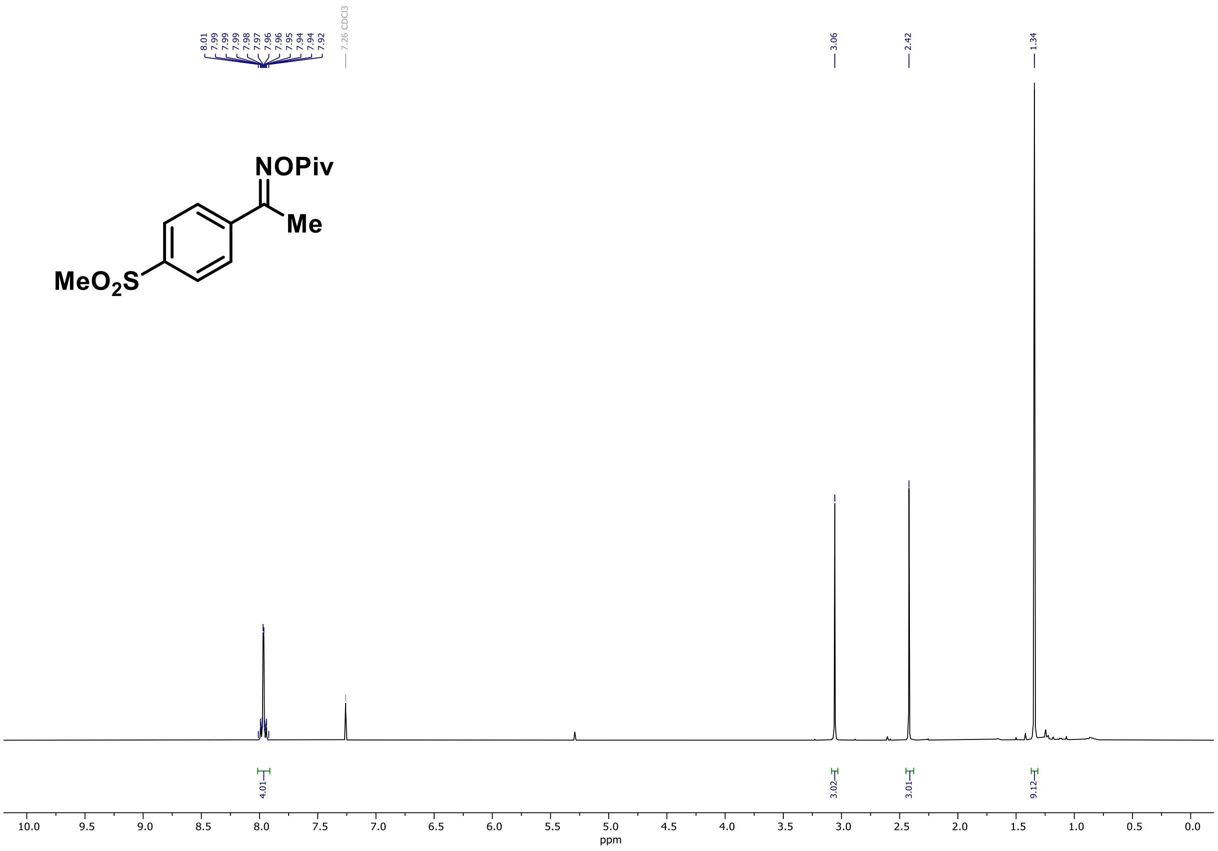

^{13}C{^{1}H} NMR ($CDCl_3$, 101 MHz) for **2y**

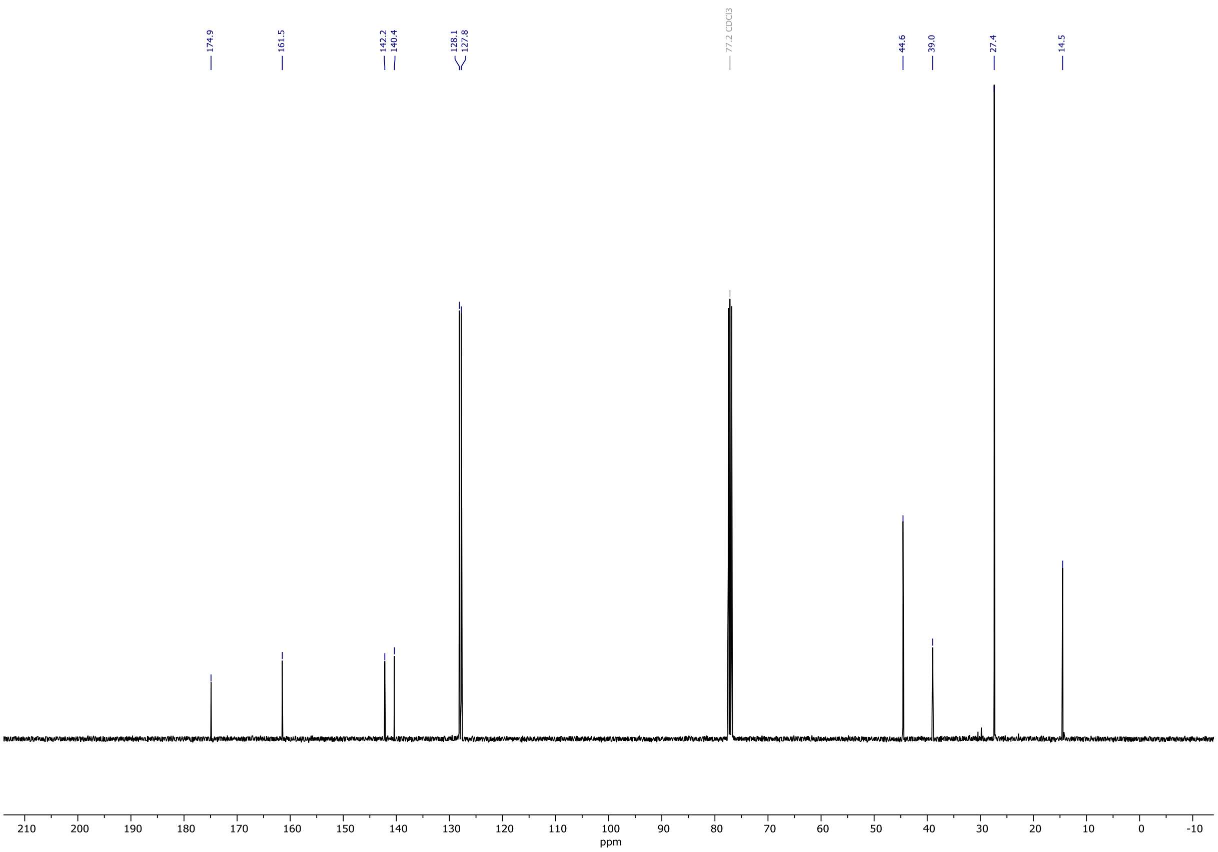

^{1}H NMR ($CDCl_3$, 400 MHz) for **2z**

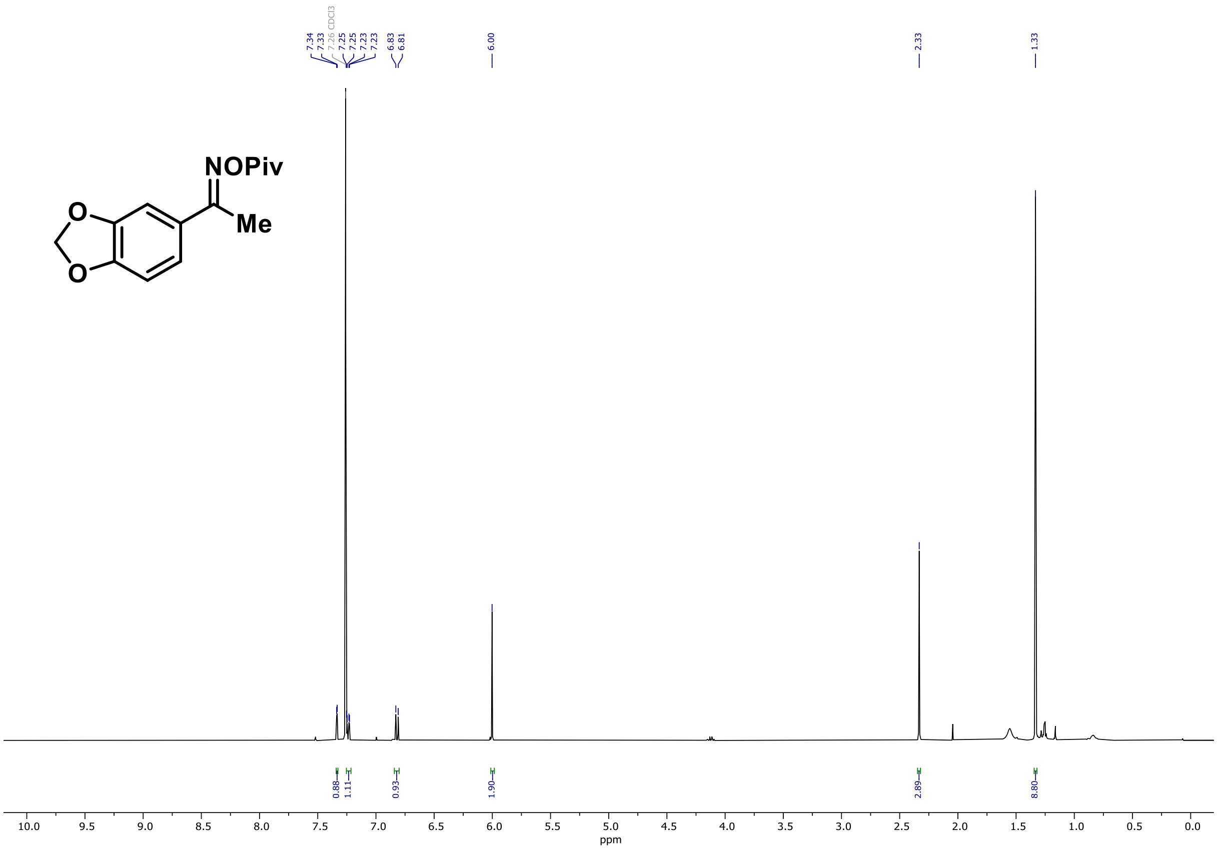

^{13}C{^{1}H} NMR ($CDCl_3$, 101 MHz) for **2z**

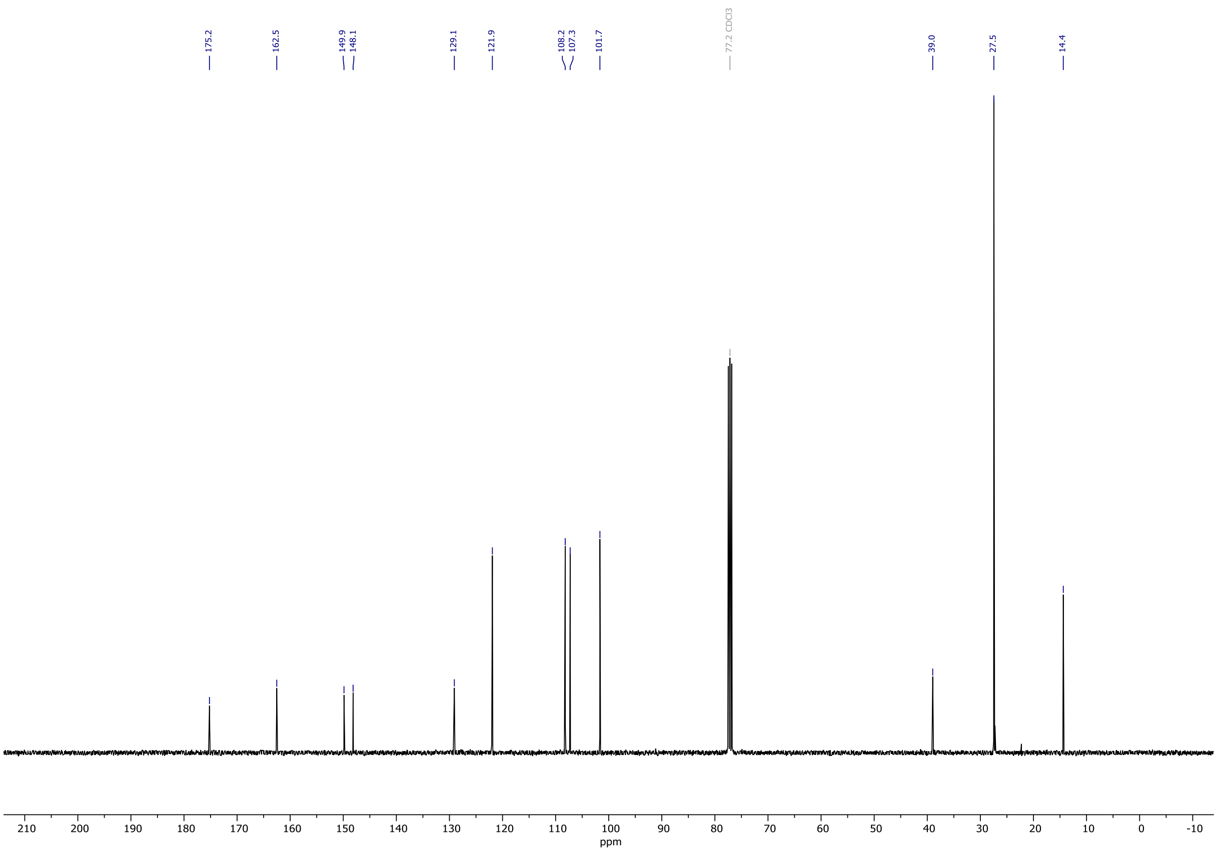

¹H NMR (CDCl$_3$, 400 MHz) for **2aa**

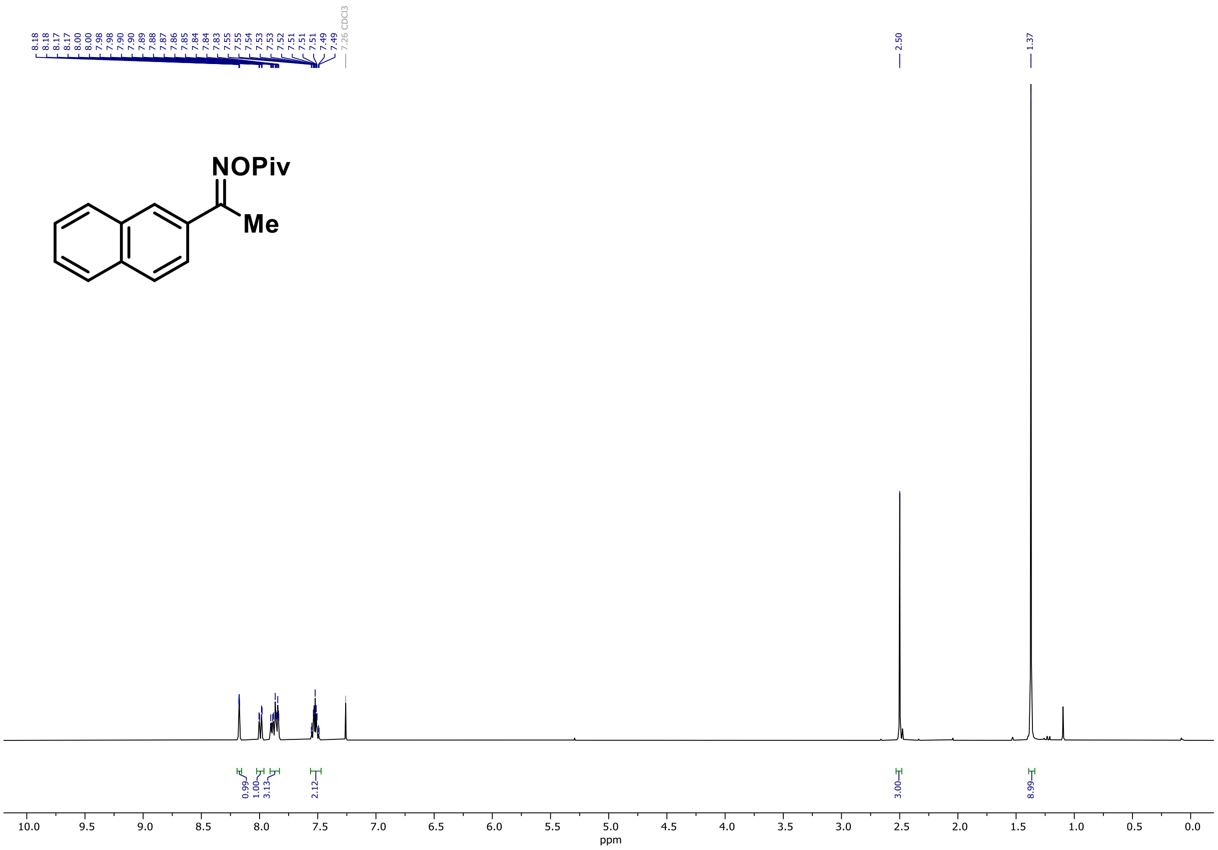

¹³C{¹H} NMR (CDCl$_3$, 101 MHz) for **2aa**

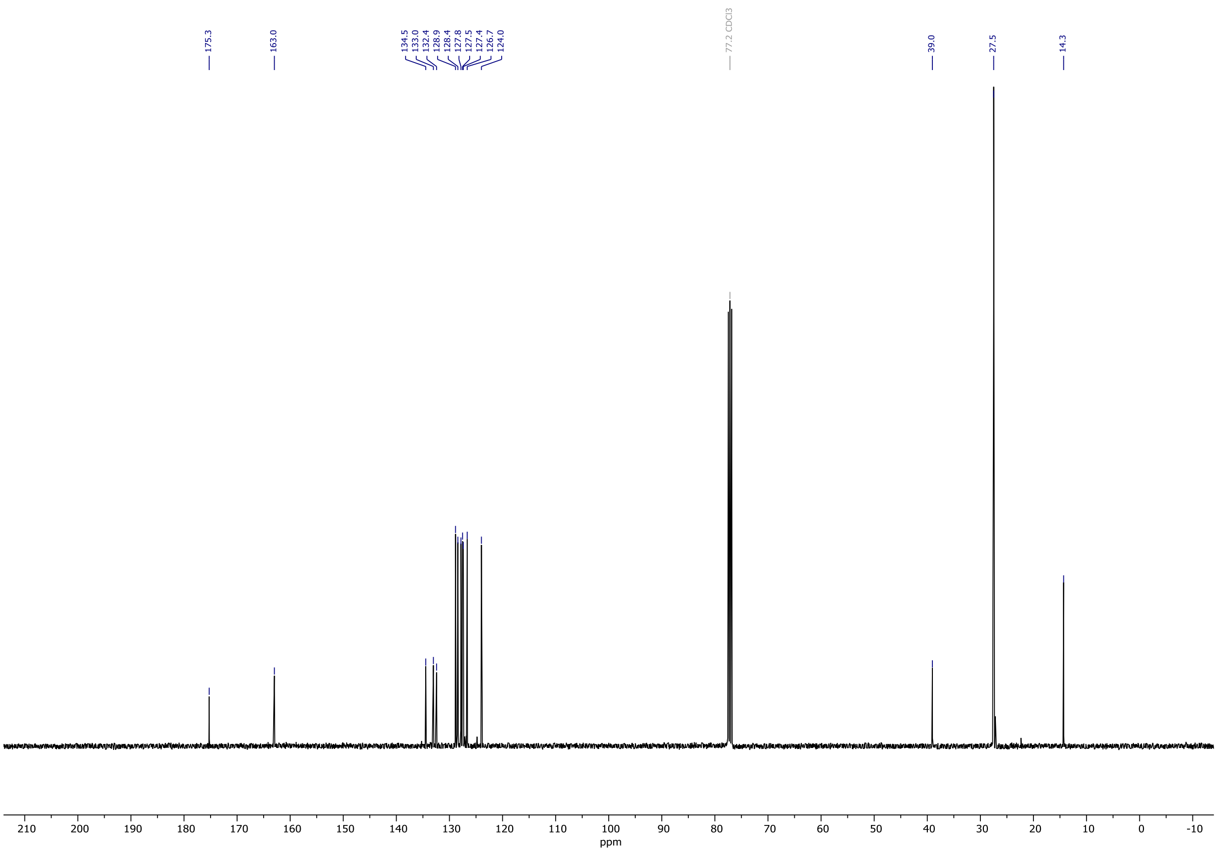

¹H NMR ($CDCl_3$, 400 MHz) for **2ab**

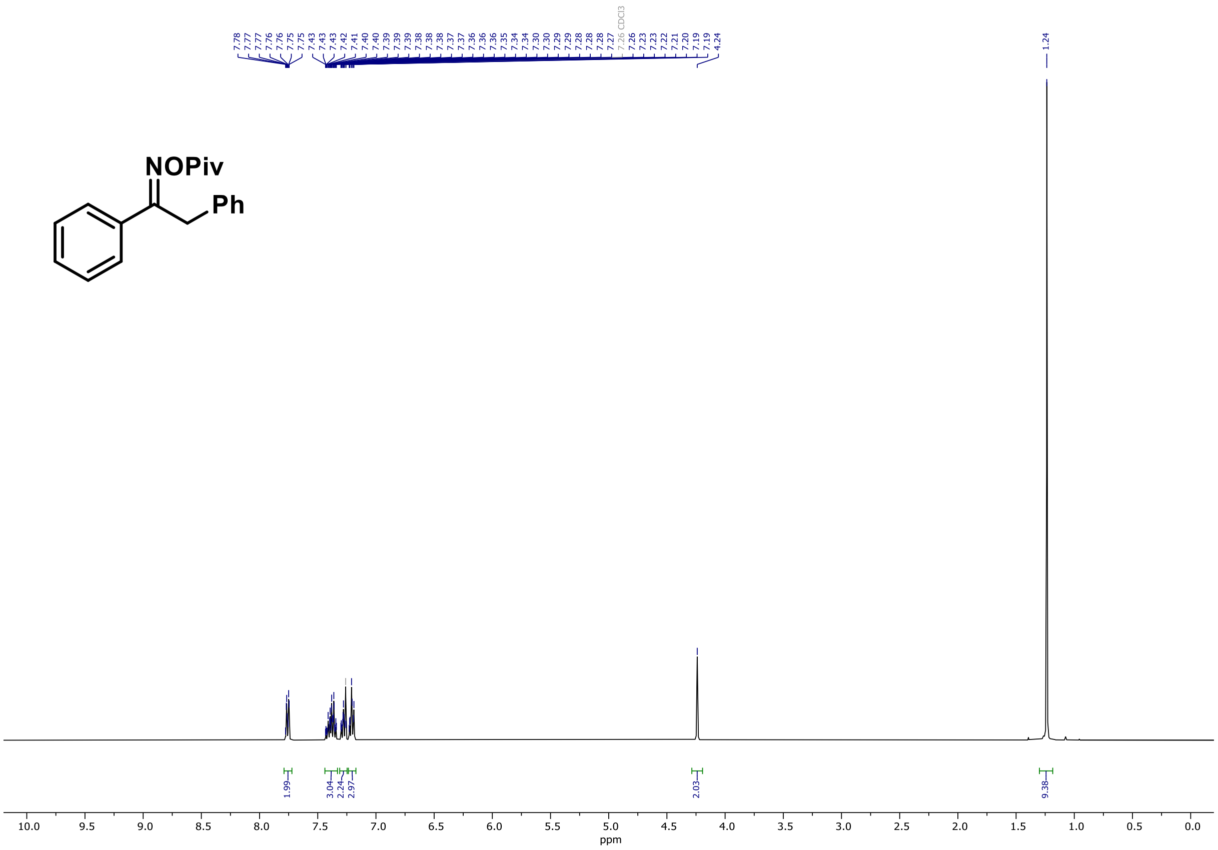

¹³C{¹H} NMR ($CDCl_3$, 101 MHz) for **2ab**

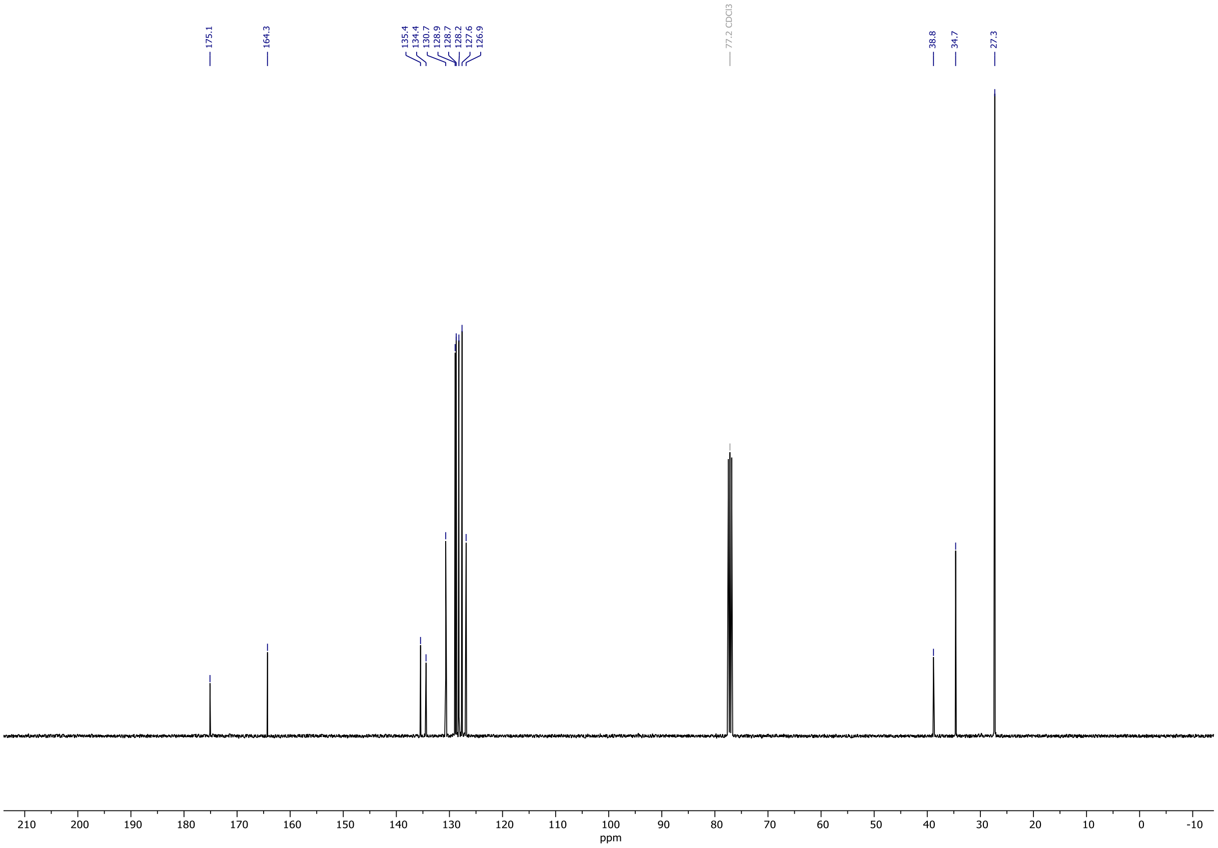

^{1}H NMR (CDCl$_3$, 599 MHz) for **2ac**

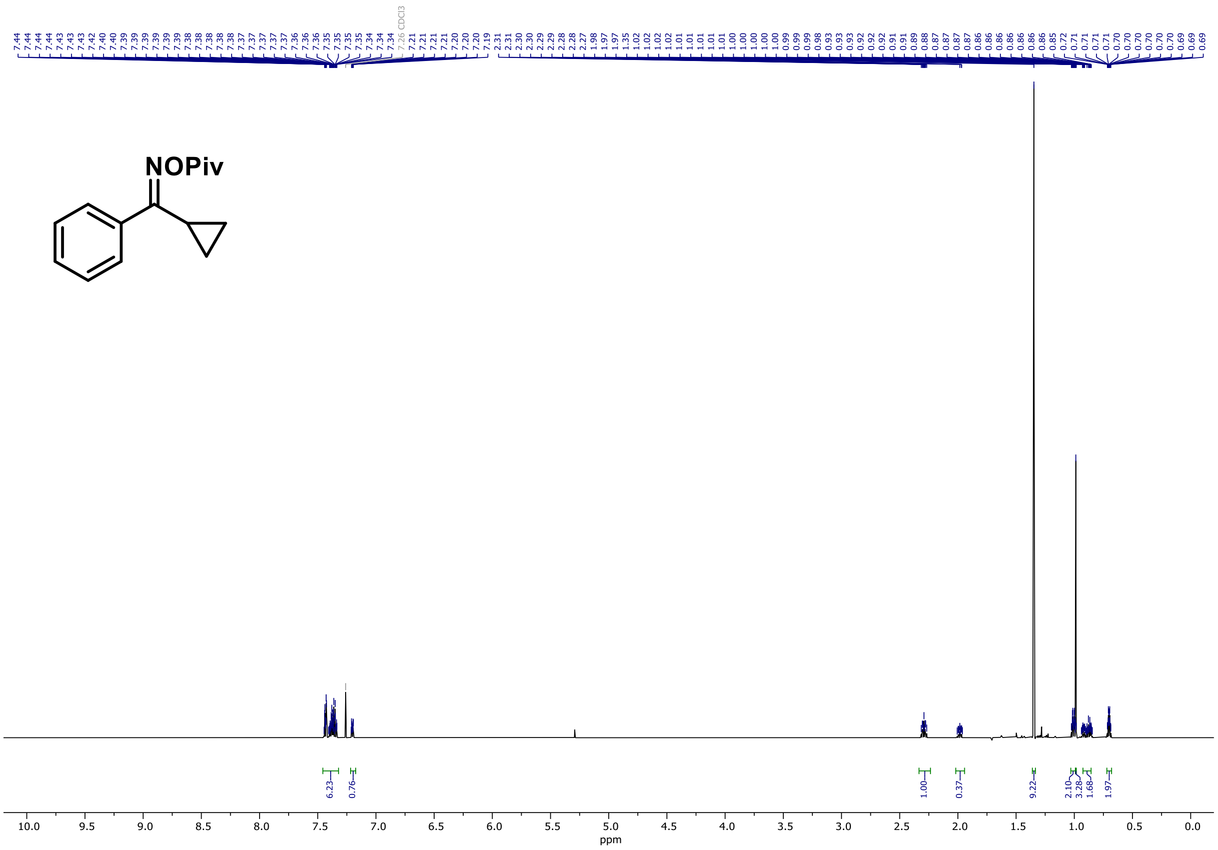

^{13}C{^{1}H} NMR (CDCl$_3$, 151 MHz) for **2ac**

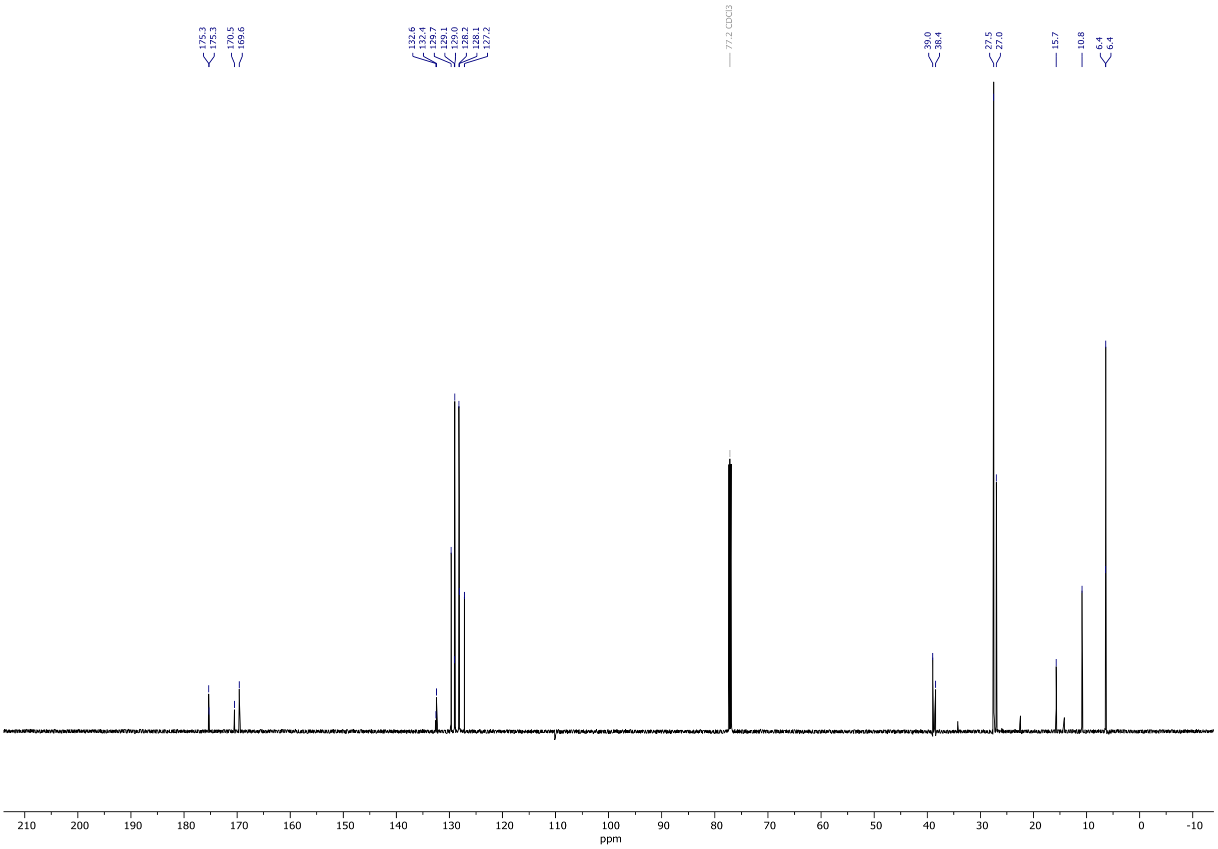

^{1}H NMR (CDCl$_3$, 400 MHz) for **2ad**

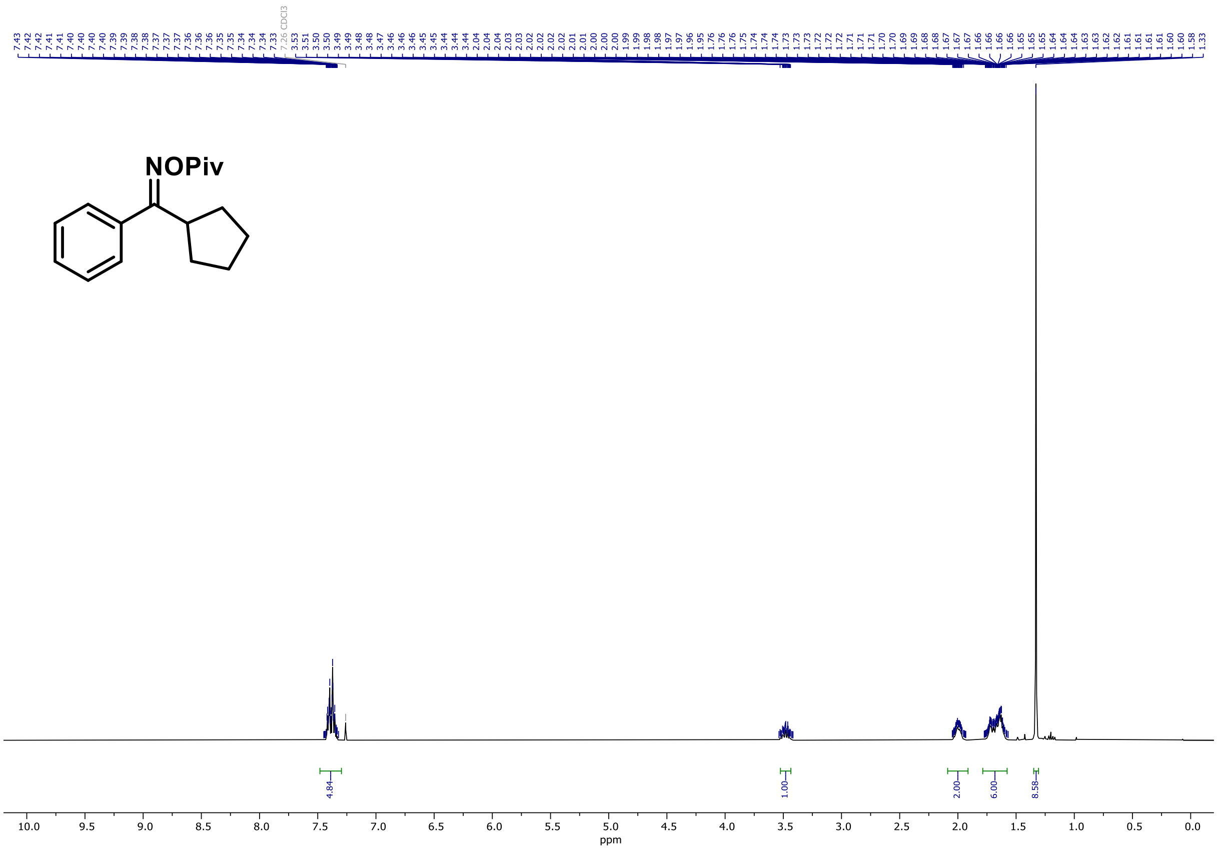

^{13}C{^{1}H} NMR (CDCl$_3$, 101 MHz) for **2ad**

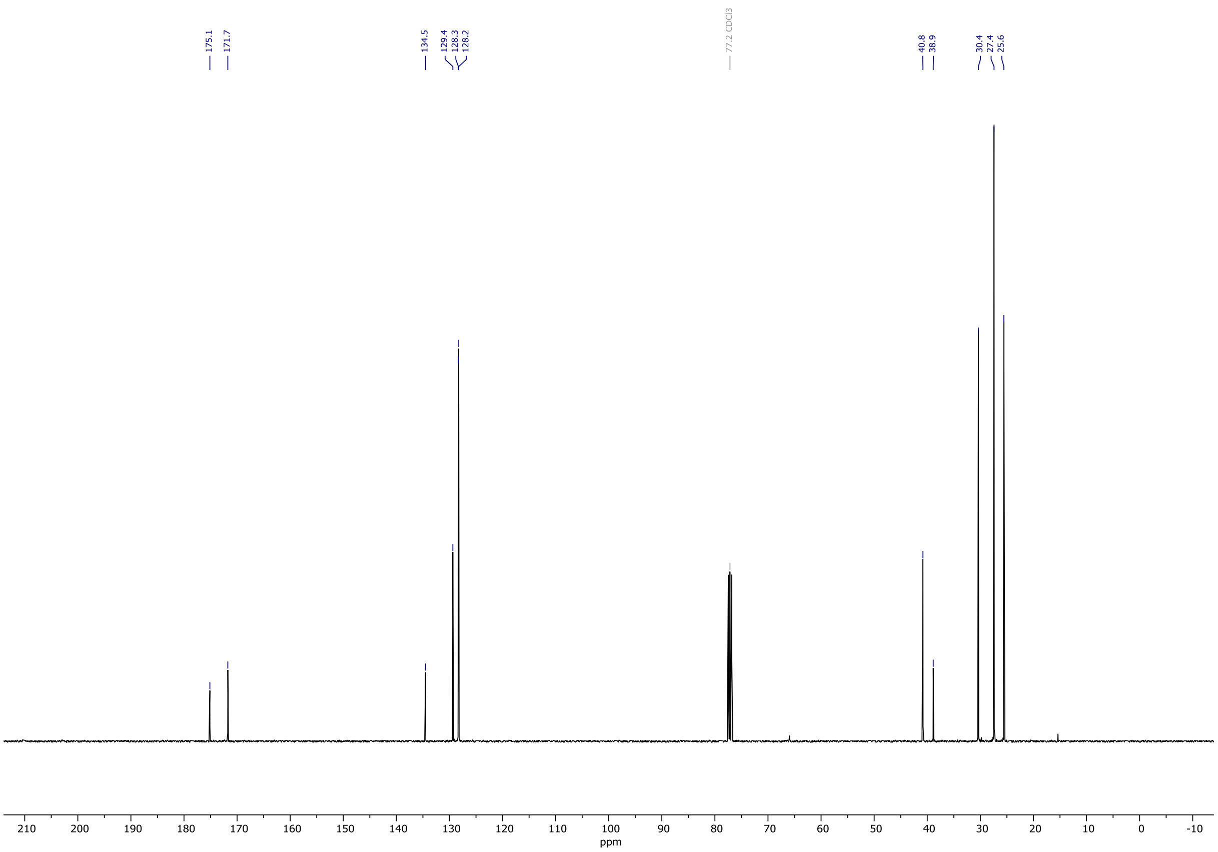

^{1}H NMR ($CDCl_3$, 400 MHz) for **2ae**

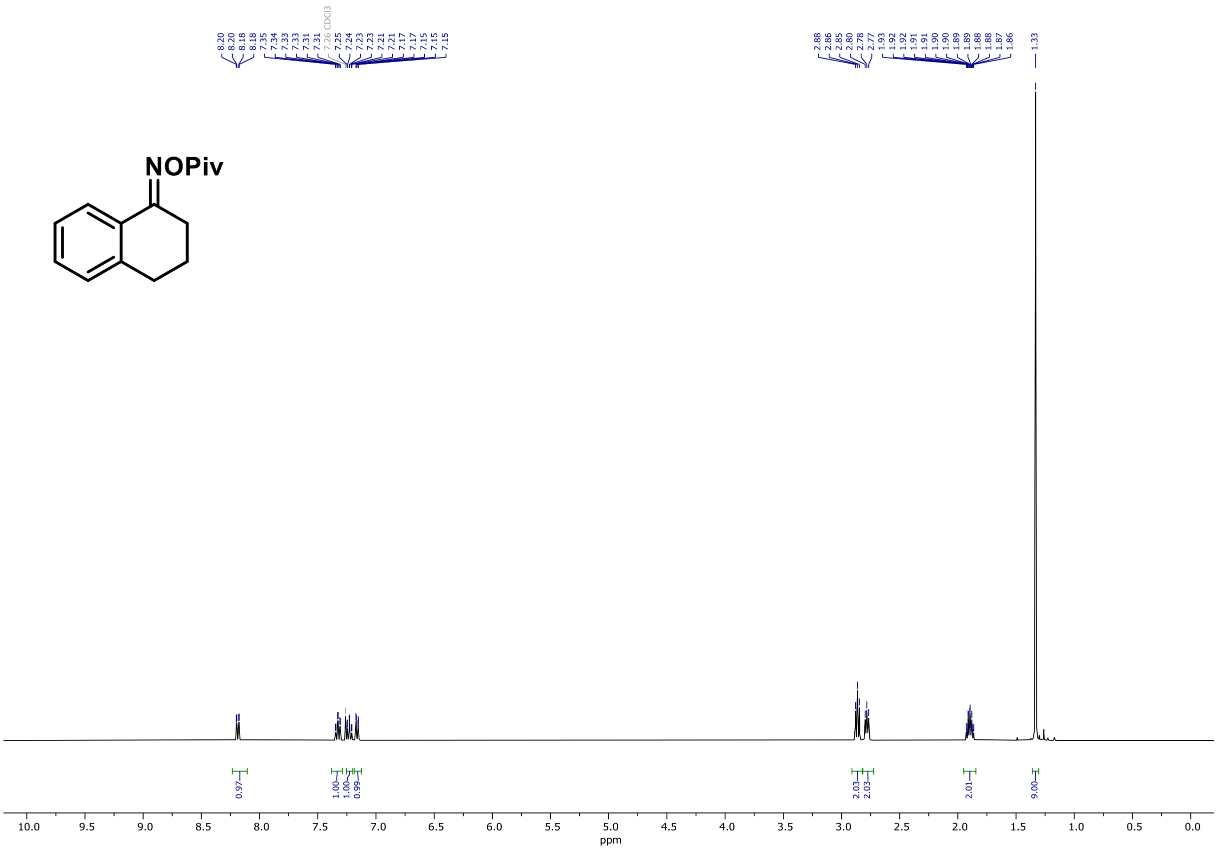

^{13}C{^{1}H} NMR ($CDCl_3$, 101 MHz) for **2ae**

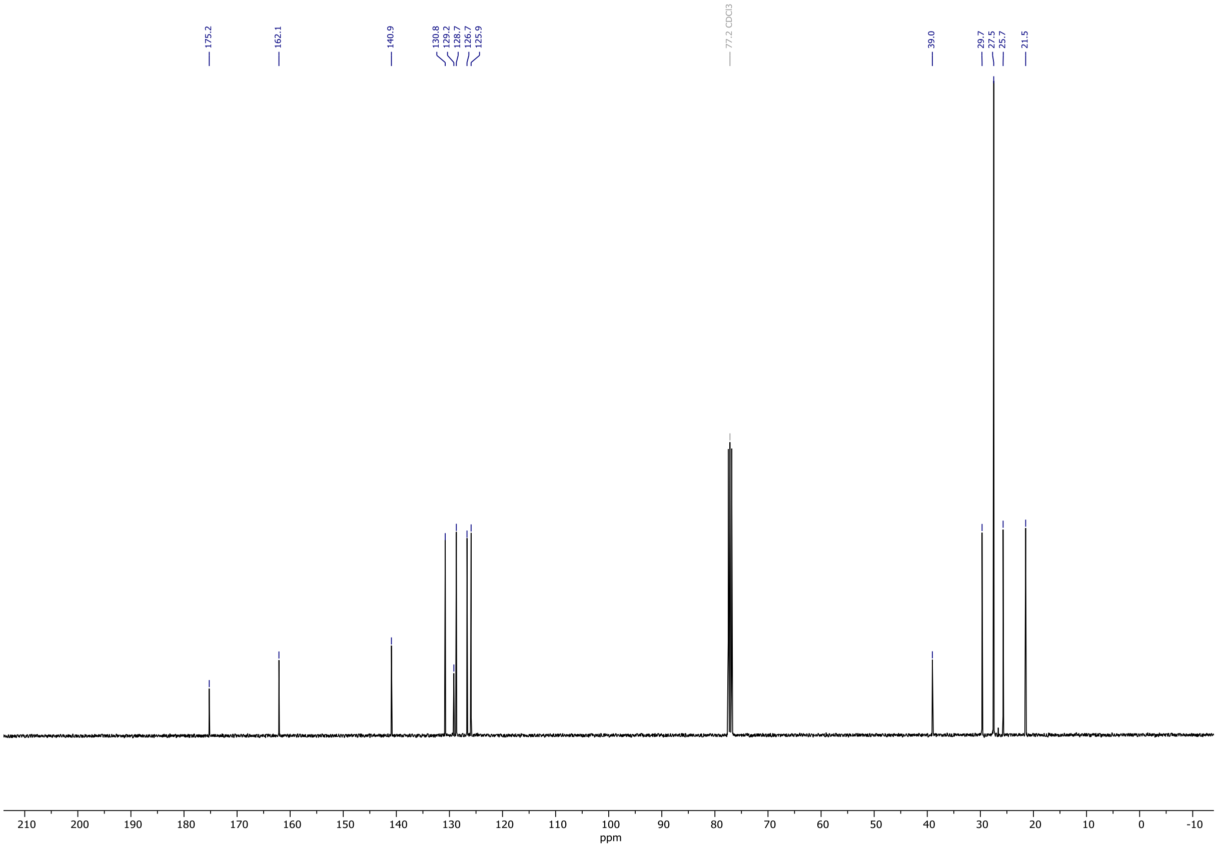

^{1}H NMR ($CDCl_3$, 400 MHz) for **2af**

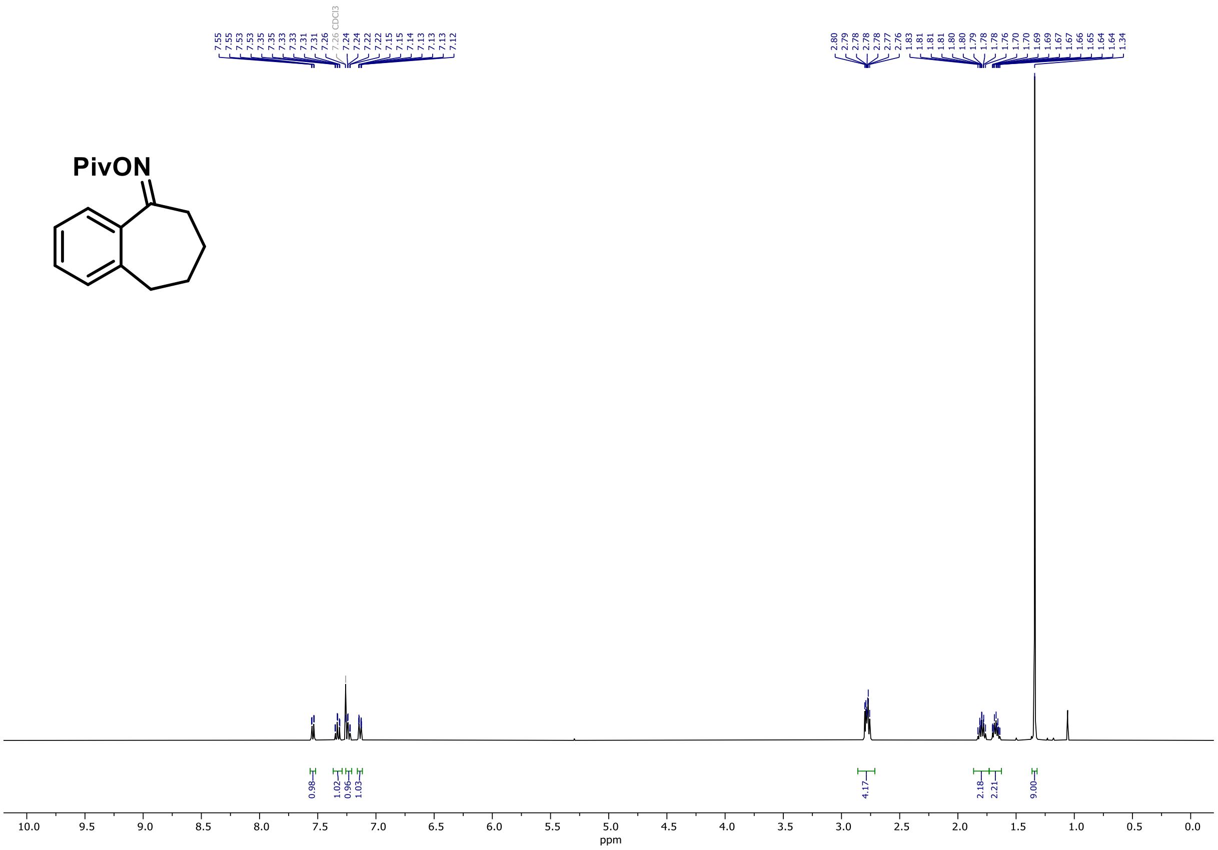

^{13}C{^{1}H} NMR ($CDCl_3$, 101 MHz) for **2af**

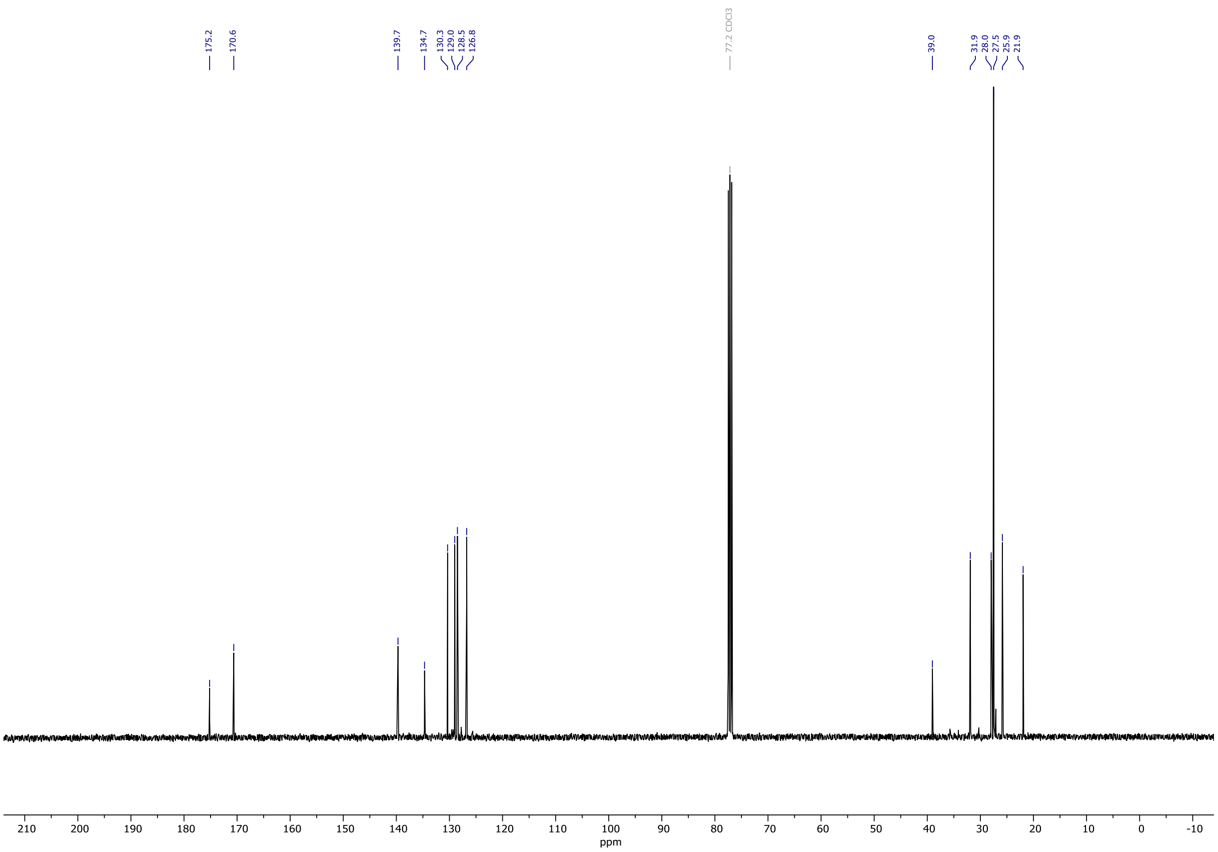

8.2 Substrate scope

¹H NMR ($CDCl_3$, 400 MHz) for **3a**

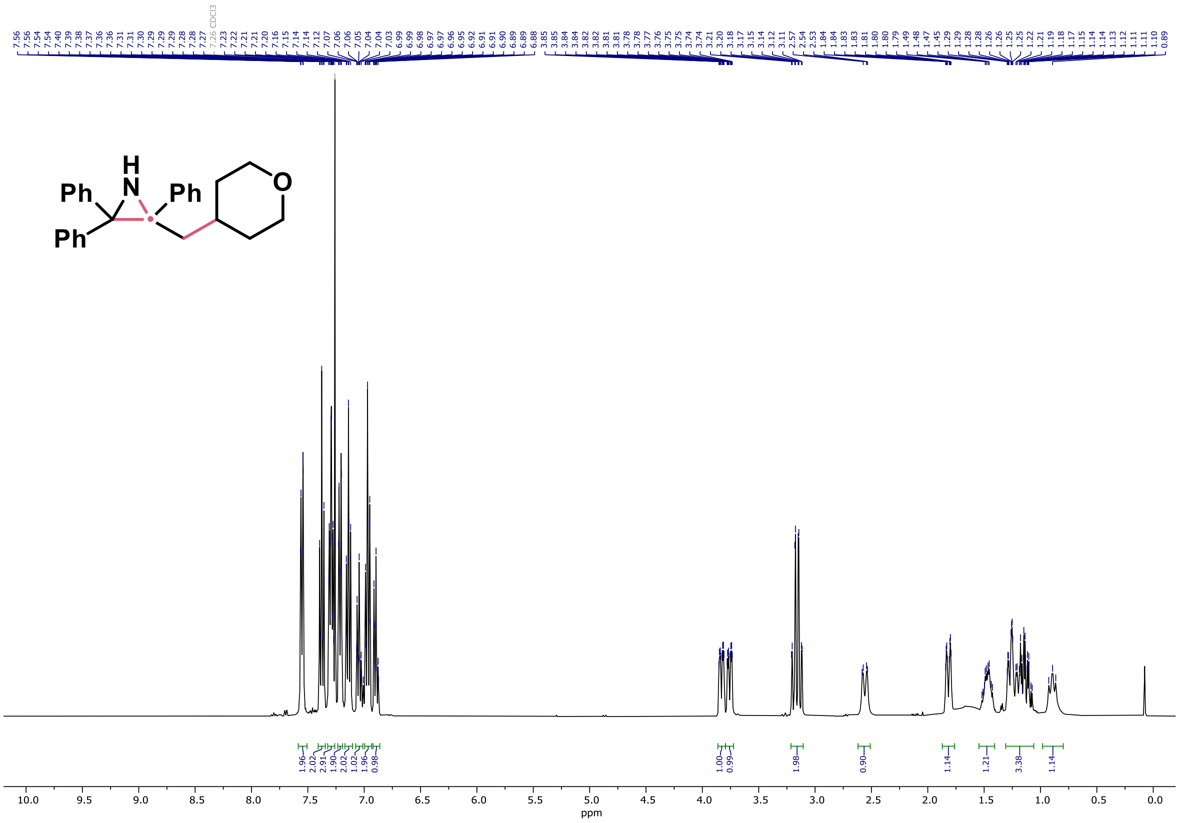

¹³C{¹H} NMR ($CDCl_3$, 101 MHz) for **3a**

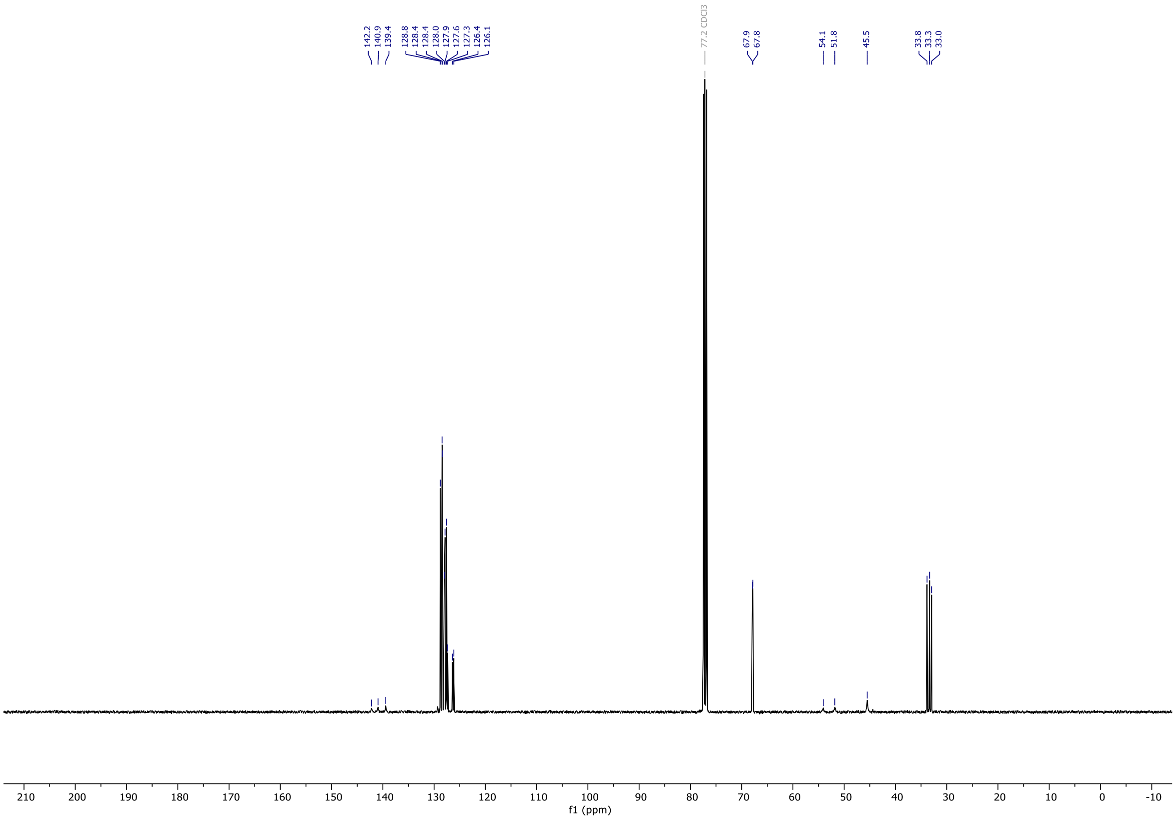

¹H NMR (CDCl$_3$, 400 MHz) for **3b**

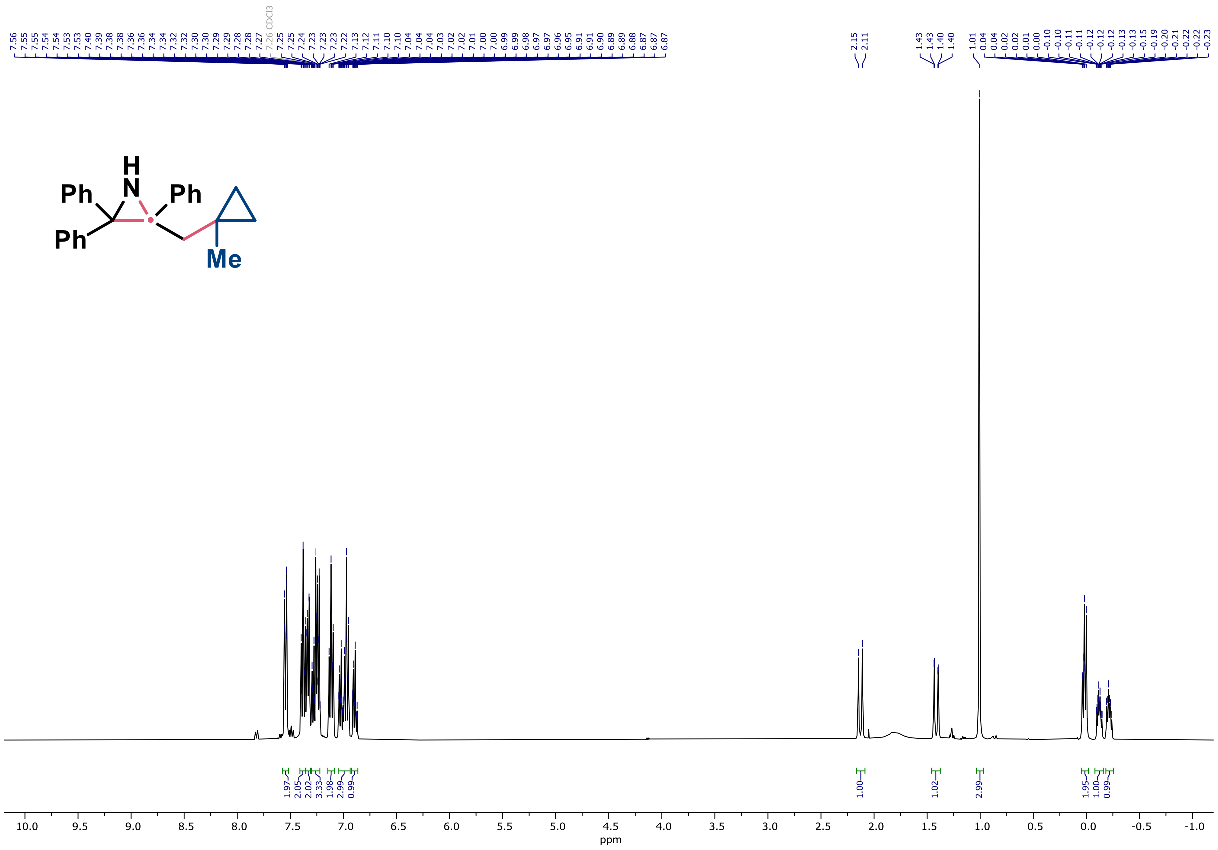

¹³C{¹H} NMR (CDCl$_3$, 151 MHz) for **3b**

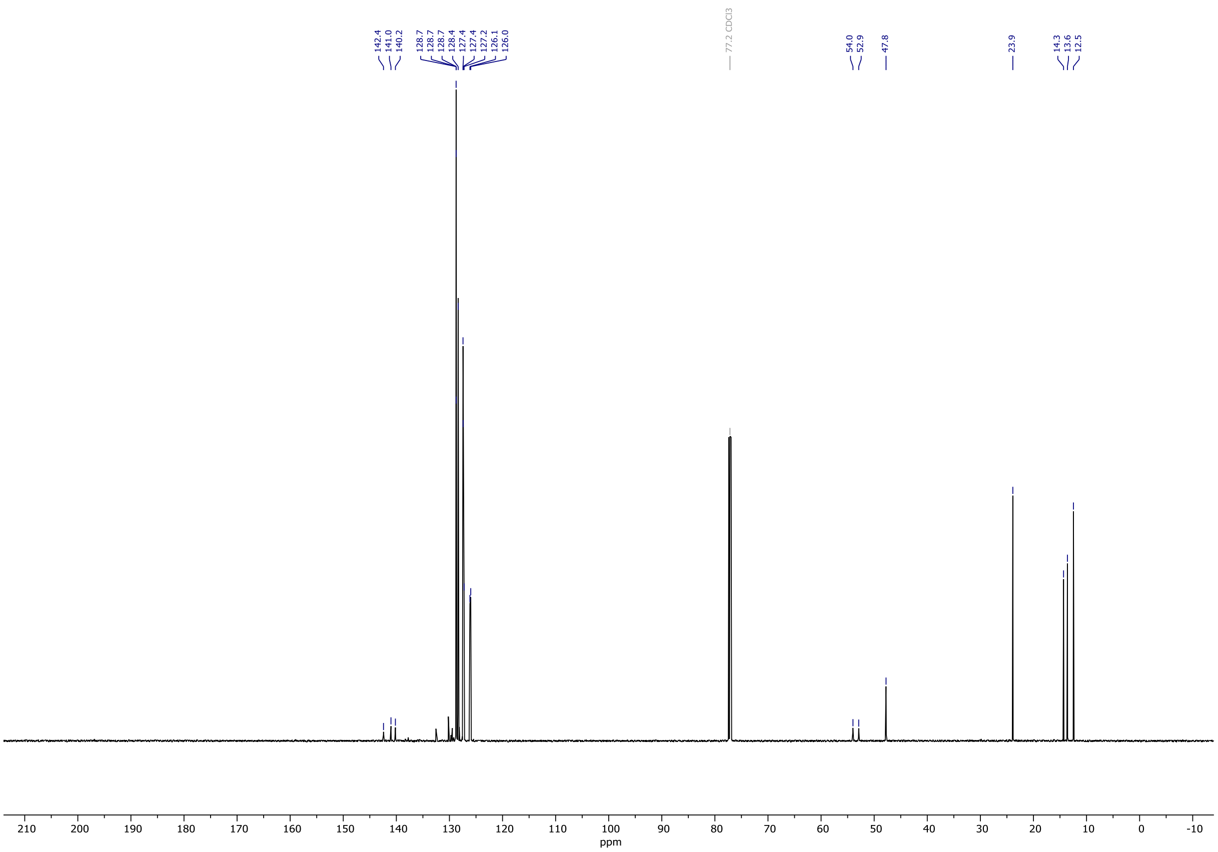

^{1}H NMR ($CDCl_3$, 400 MHz) for **3c**

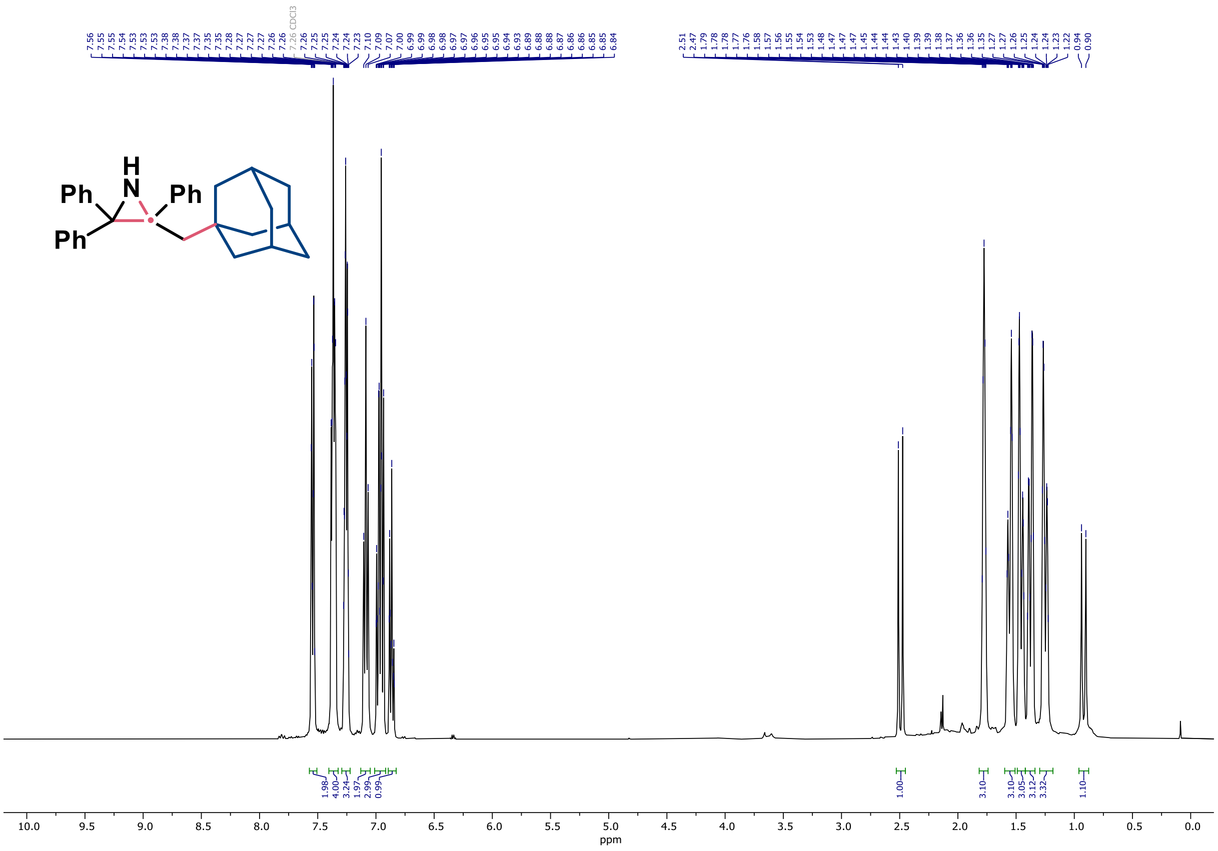

^{13}C{^{1}H} NMR ($CDCl_3$, 101 MHz) for **3c**

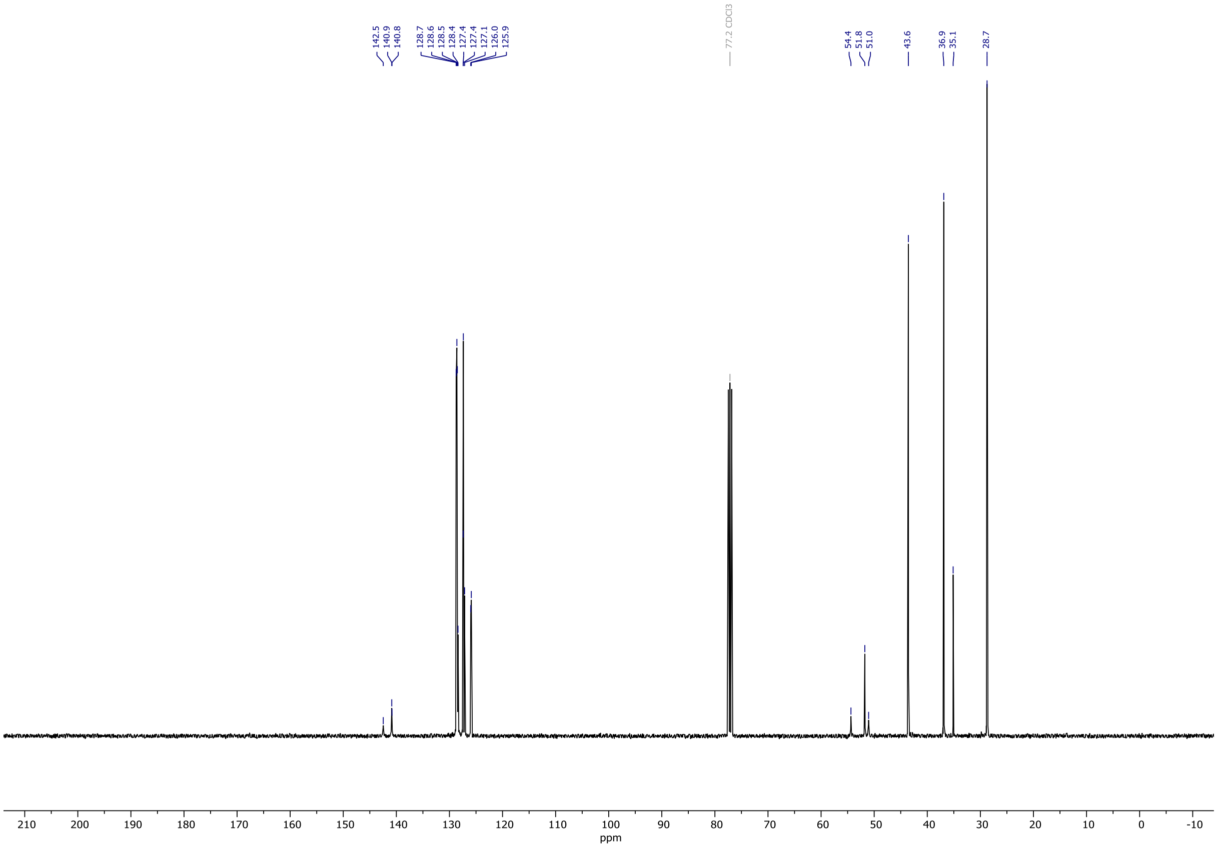

^{1}H NMR ($CDCl_3$, 400 MHz) for **3d**

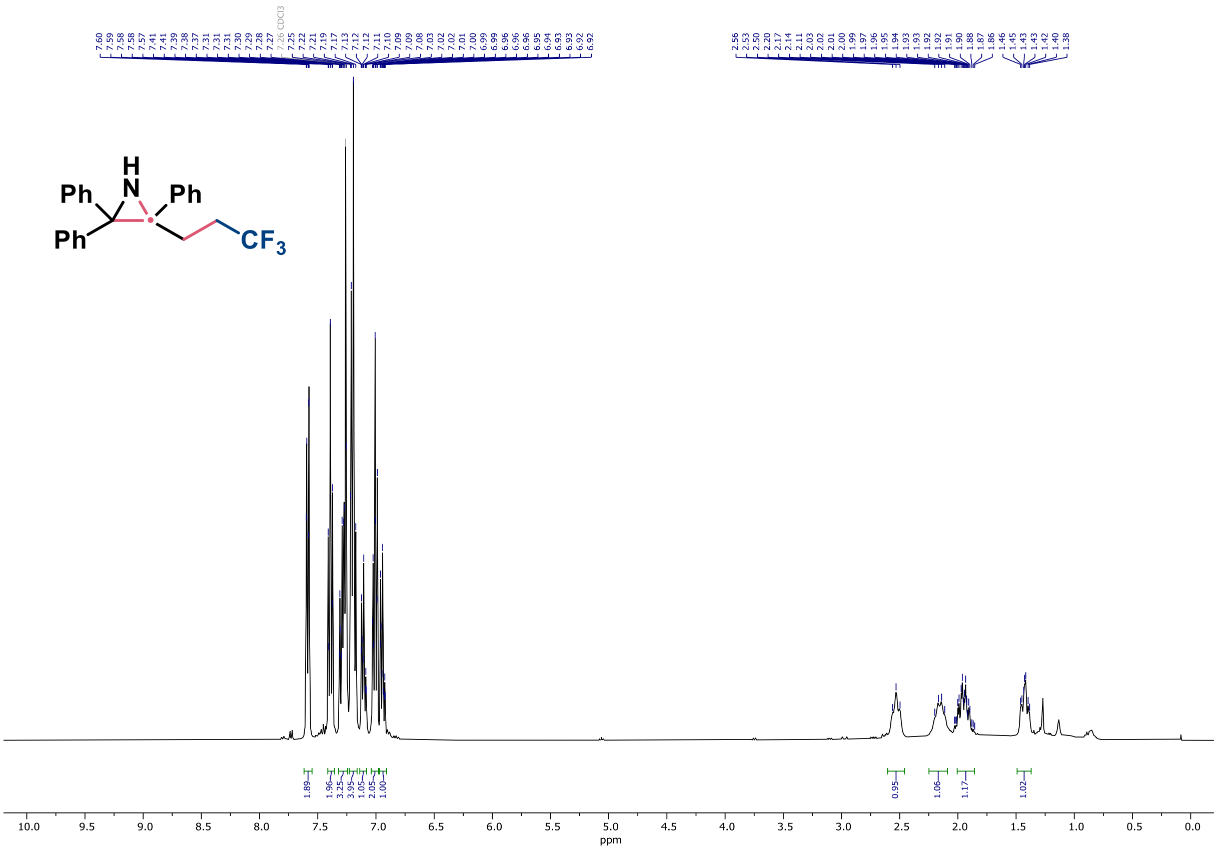

^{13}C{^{1}H} NMR ($CDCl_3$, 101 MHz) for **3d**

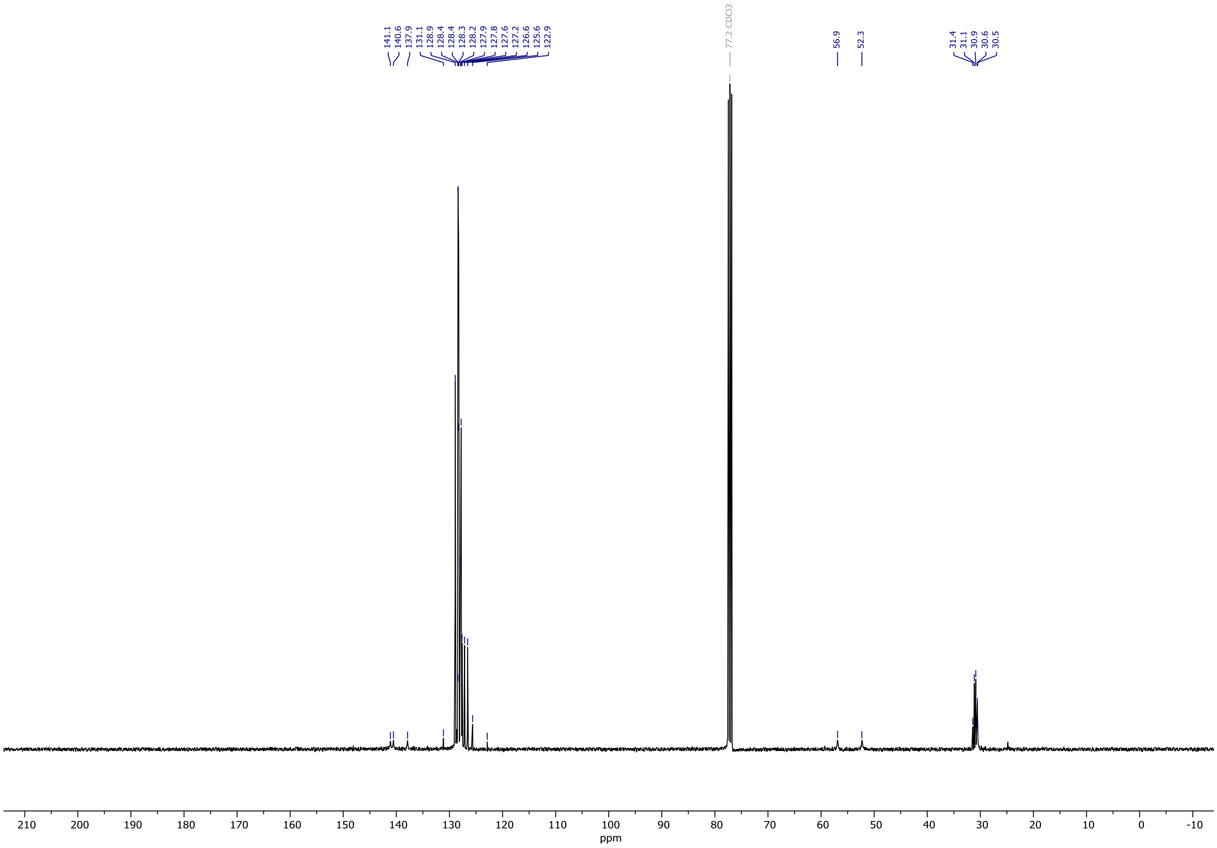

$^{19}F\{^{1}H\}$ NMR ($CDCl_3$, 376 MHz) for **3d**

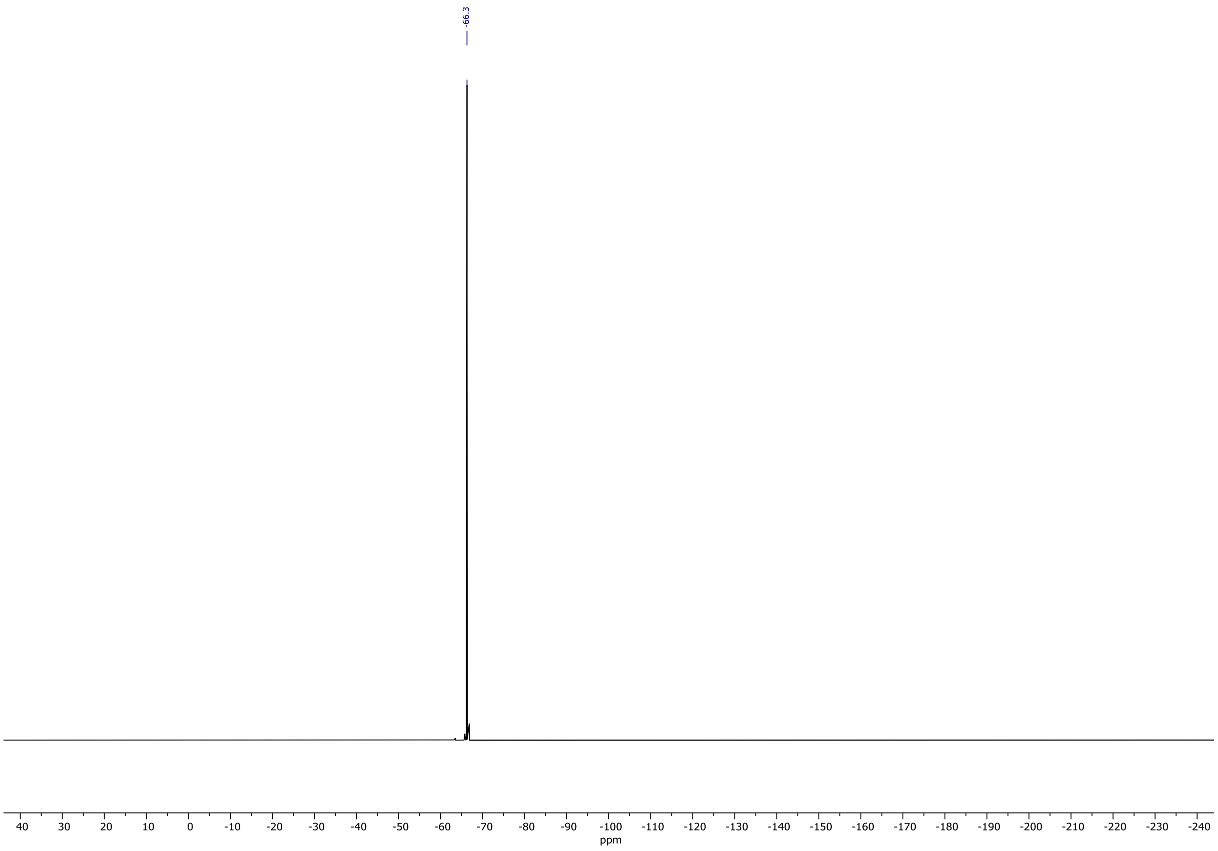

^{1}H NMR (CDCl$_3$, 400 MHz) for **3e**

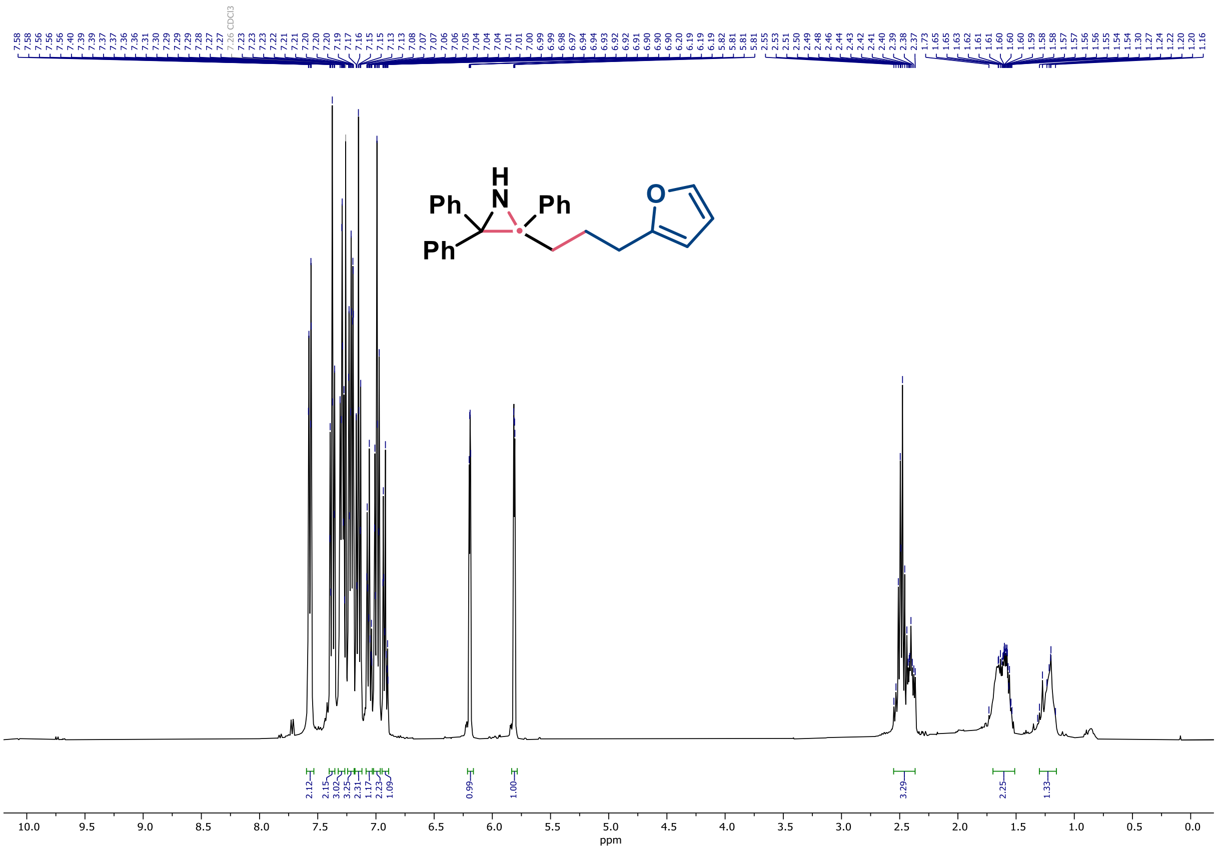

^{13}C{^{1}H} NMR (CDCl$_3$, 101 MHz) for **3e**

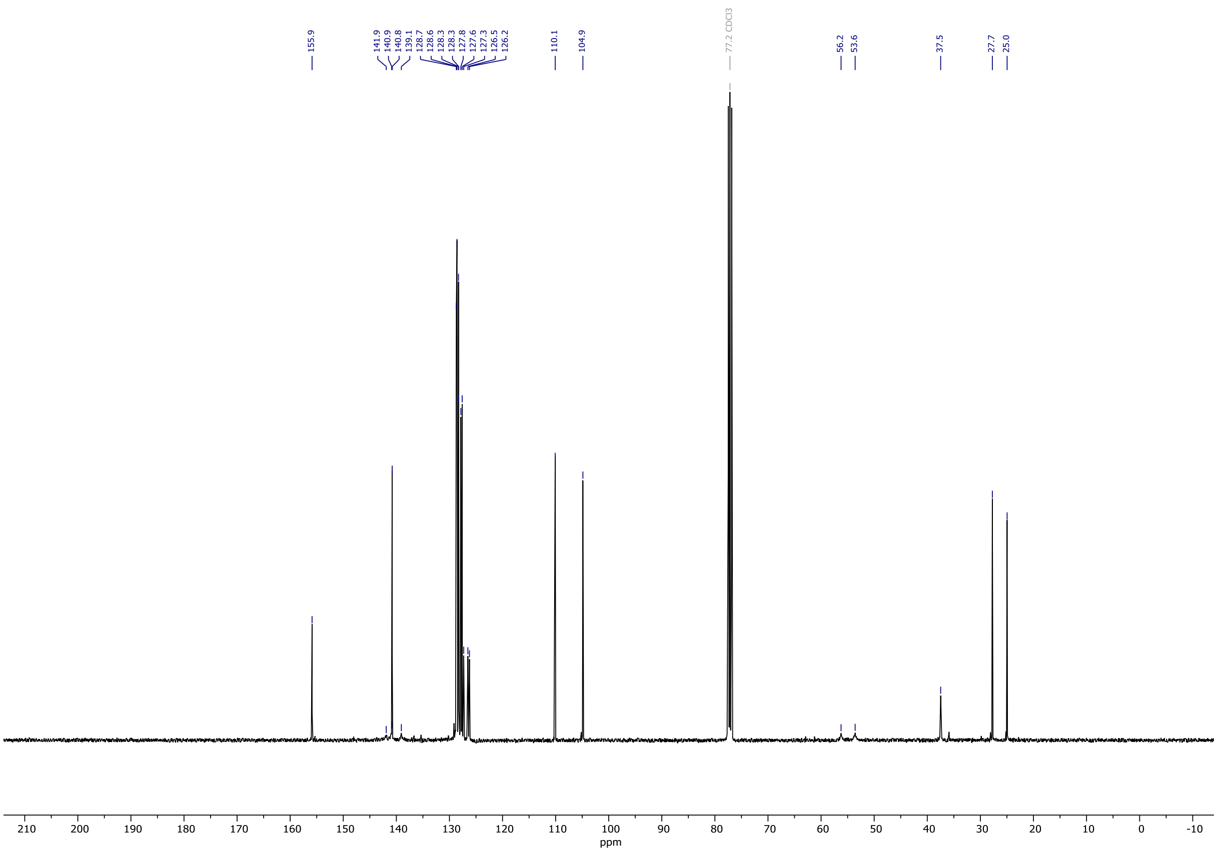

^{1}H NMR ($CDCl_3$, 400 MHz) for **3f**

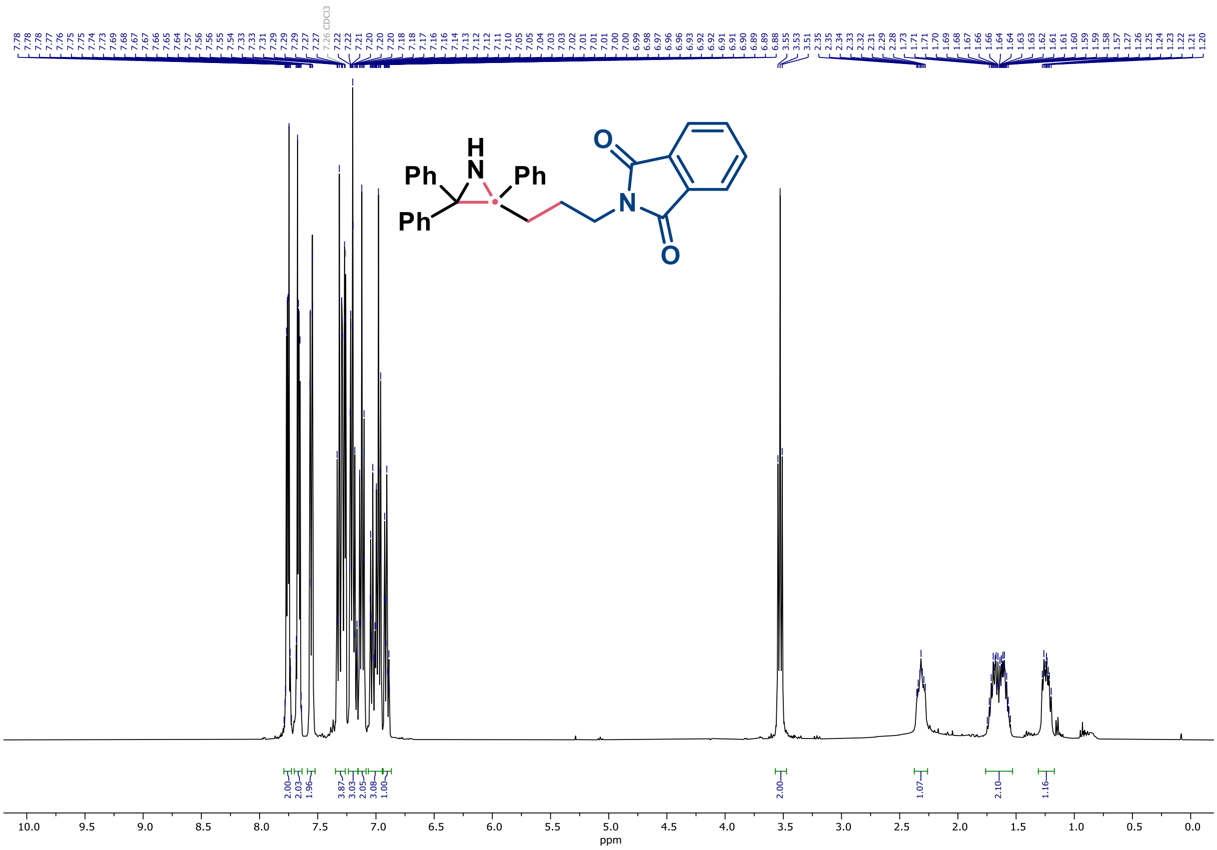

^{13}C{^{1}H} NMR ($CDCl_3$, 101 MHz) for **3f**

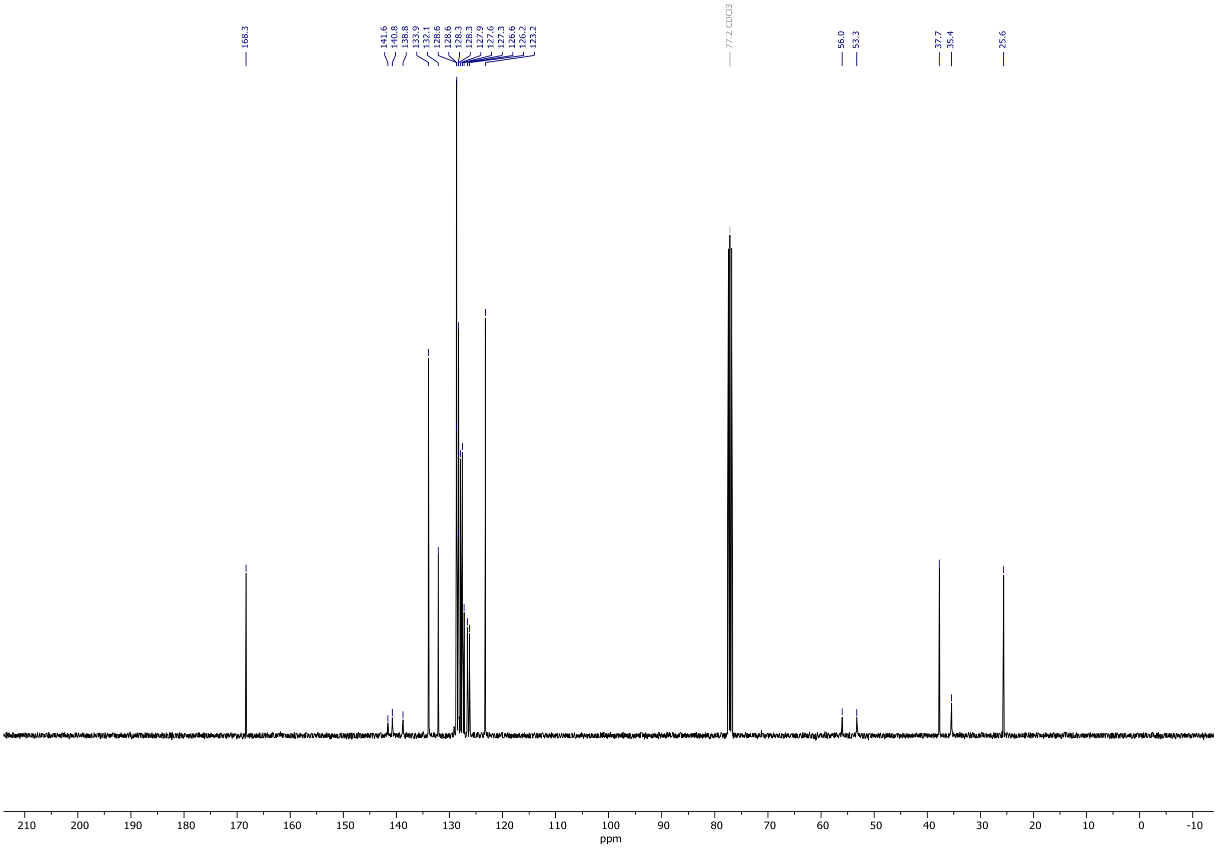

¹H NMR (CDCl$_3$, 599 MHz) for **3g**

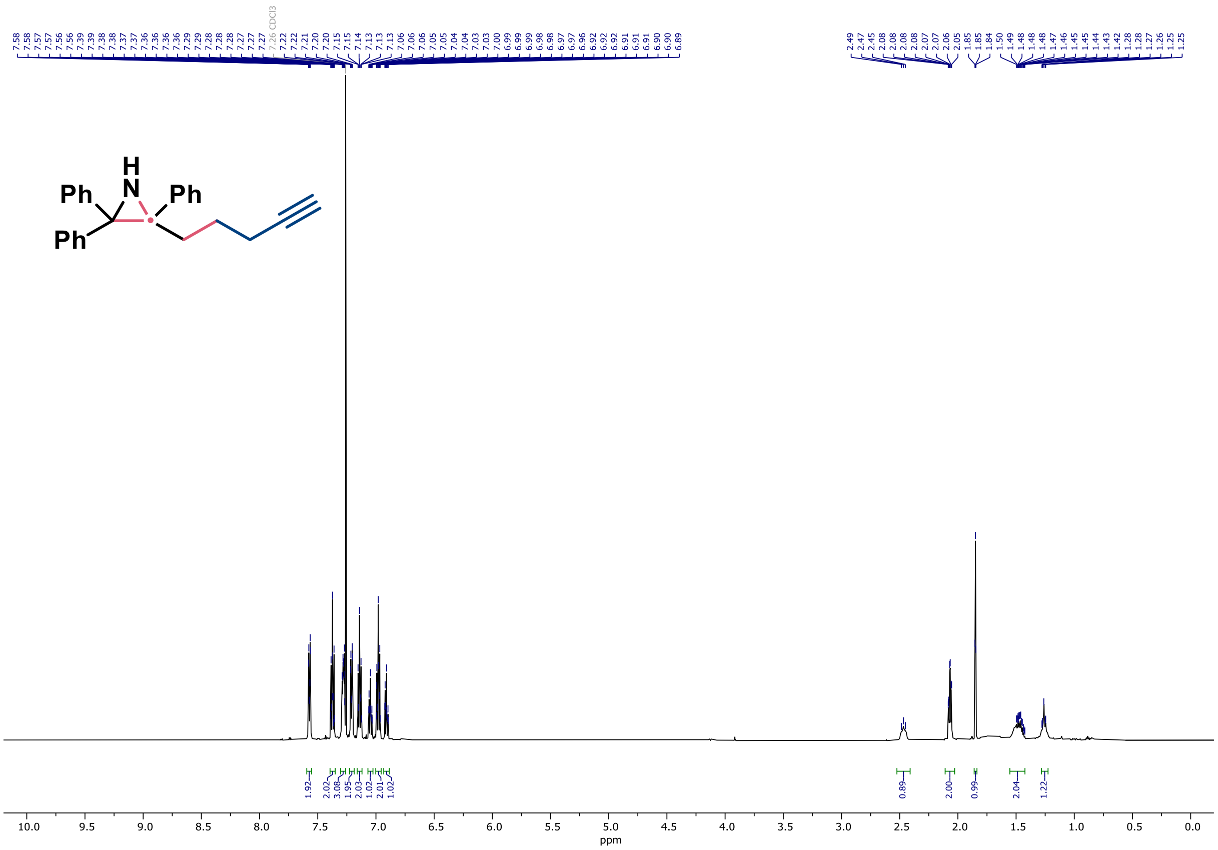

¹³C{¹H} NMR (CDCl$_3$, 151 MHz) for **3g**

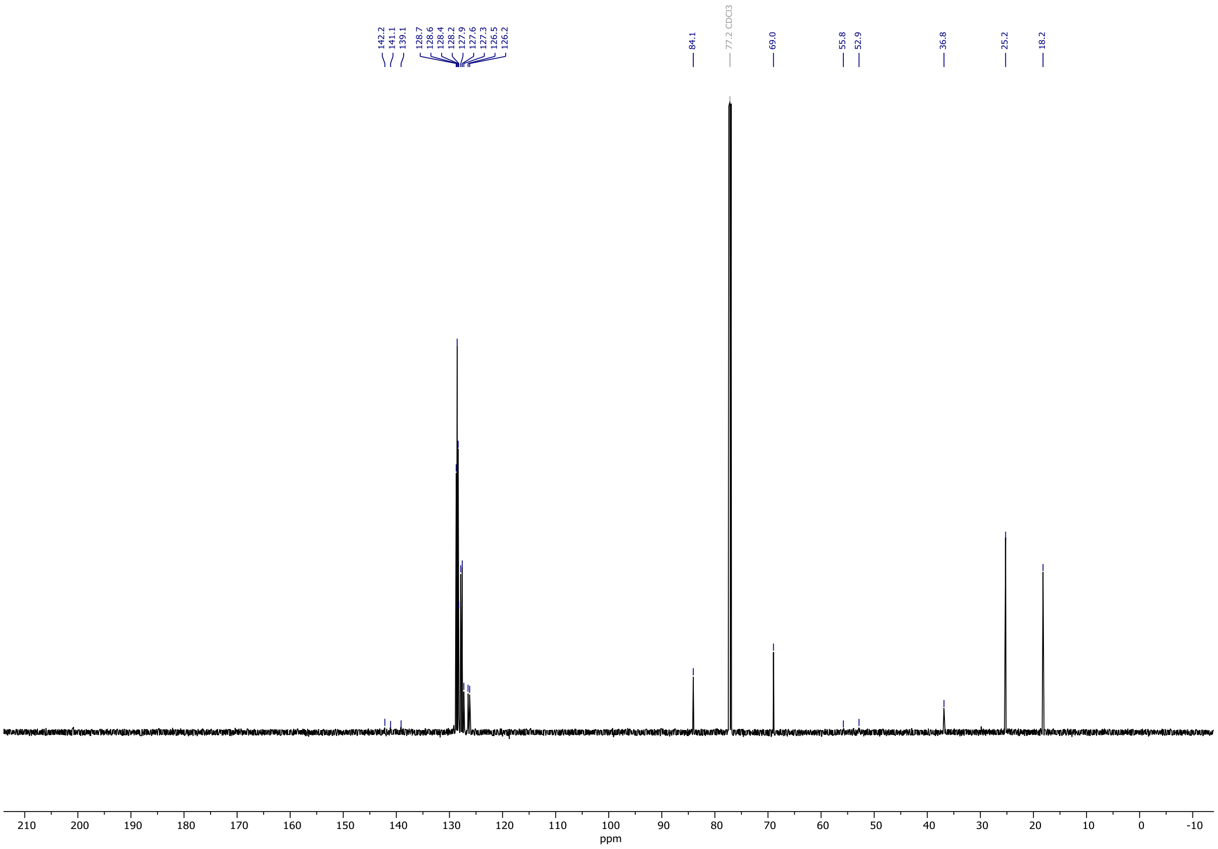

^{1}H NMR ($CDCl_3$, 500 MHz) for **3h**

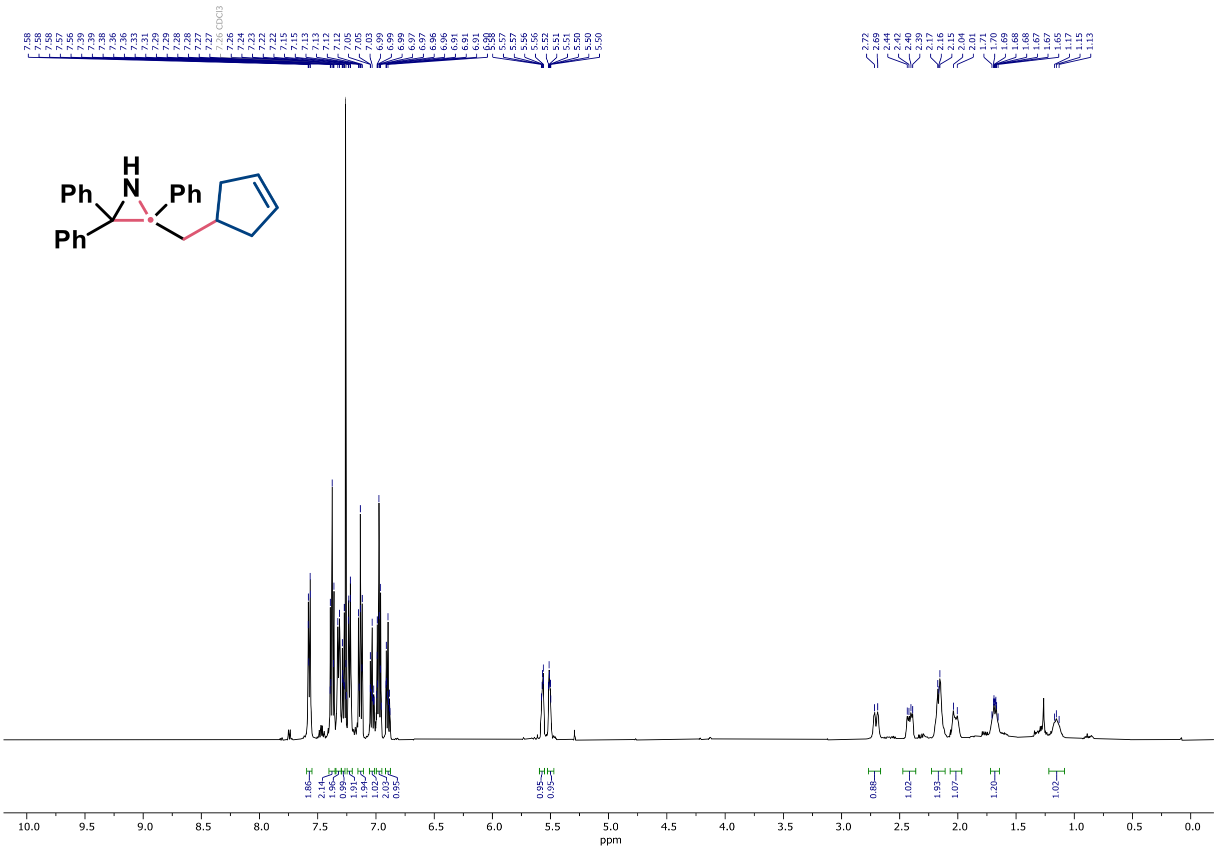

^{13}C{^{1}H} NMR ($CDCl_3$, 126 MHz) for **3h**

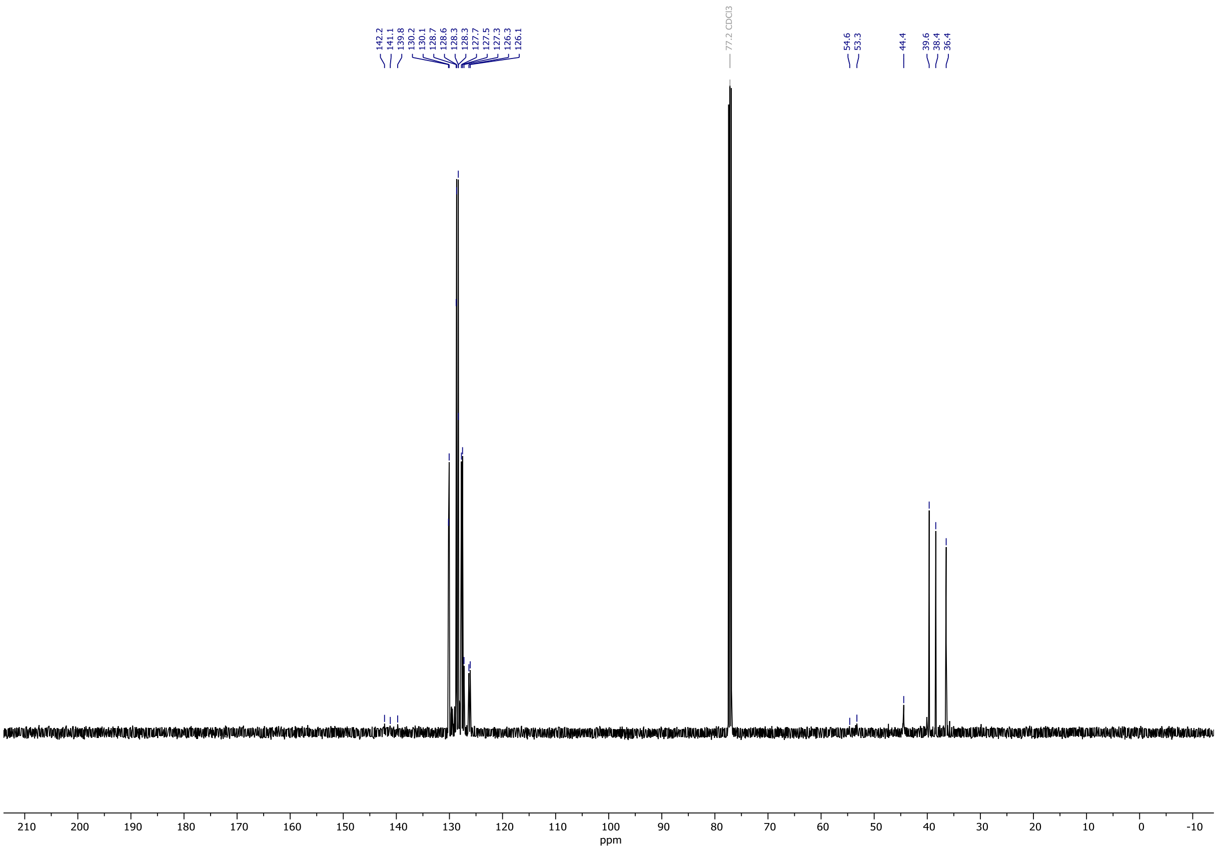

^{1}**H NMR** (CDCl$_3$, 400 MHz) for **3i**

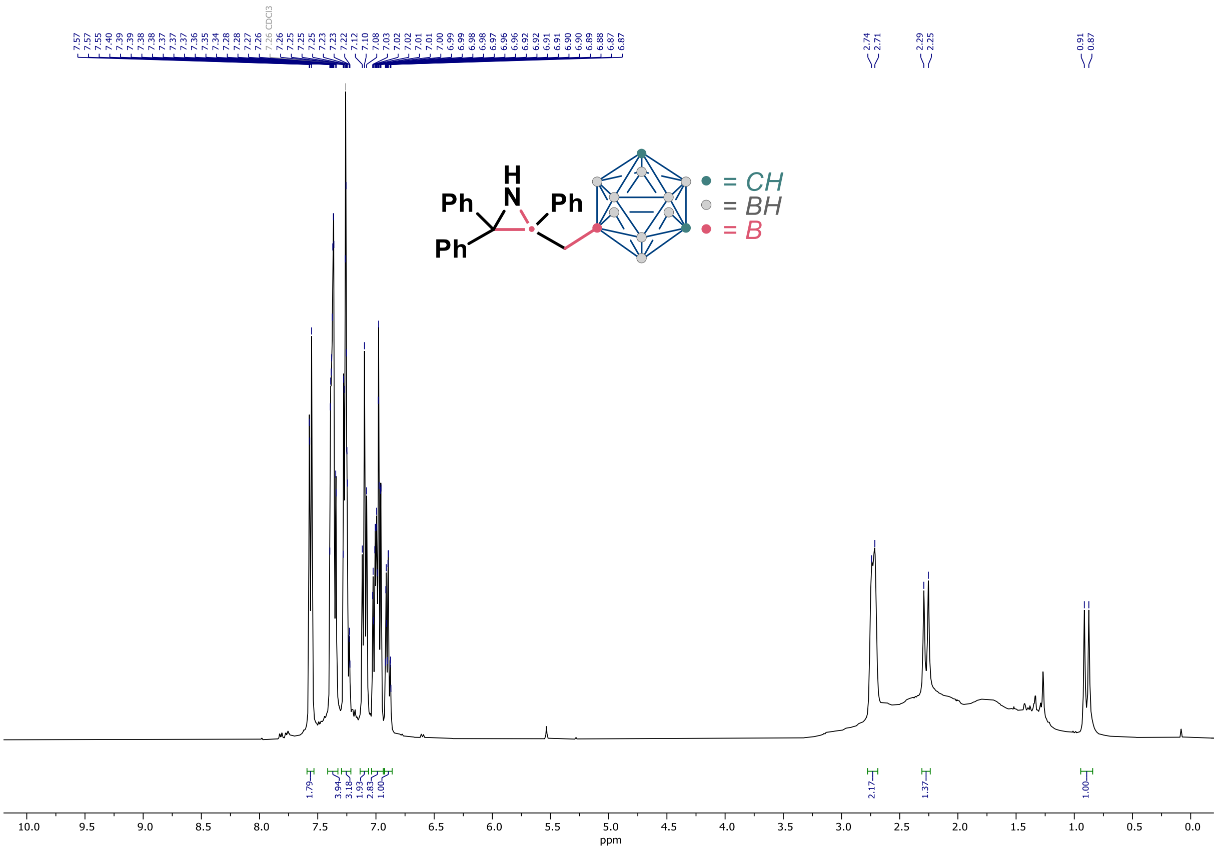

^{13}**C{^{1}H} NMR** (CDCl$_3$, 101 MHz) for **3i**

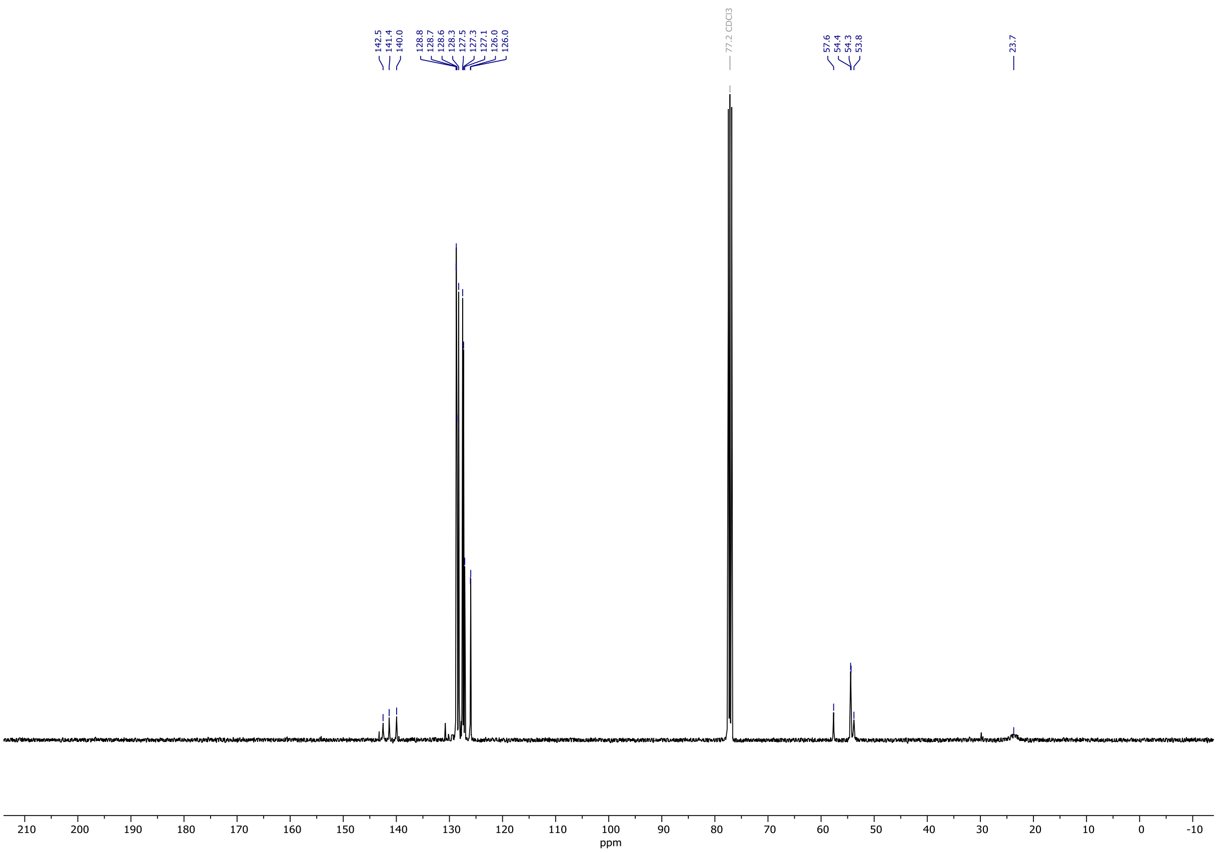

^{11}B{^{1}H} NMR ($CDCl_3$, 128 MHz) for **3i**

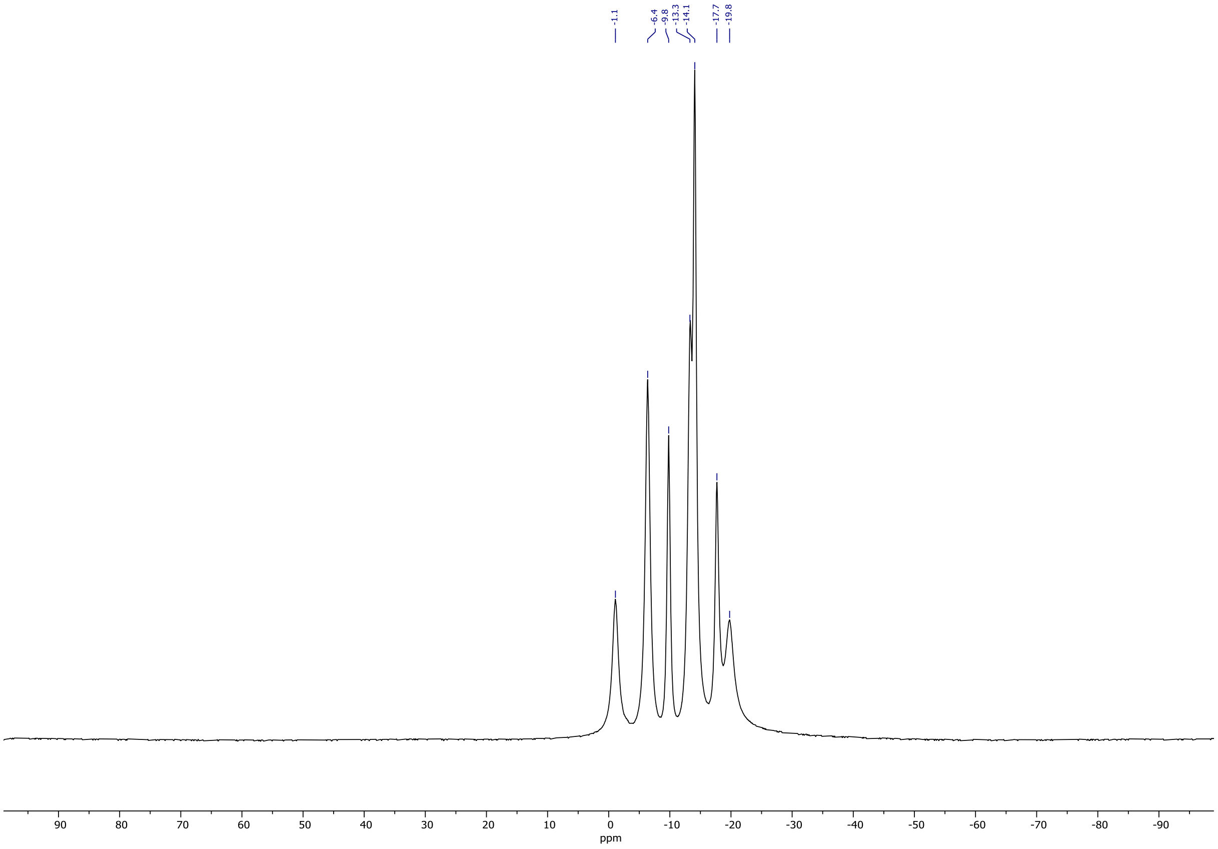

^{1}H NMR (CDCl$_3$, 400 MHz) for **3j**

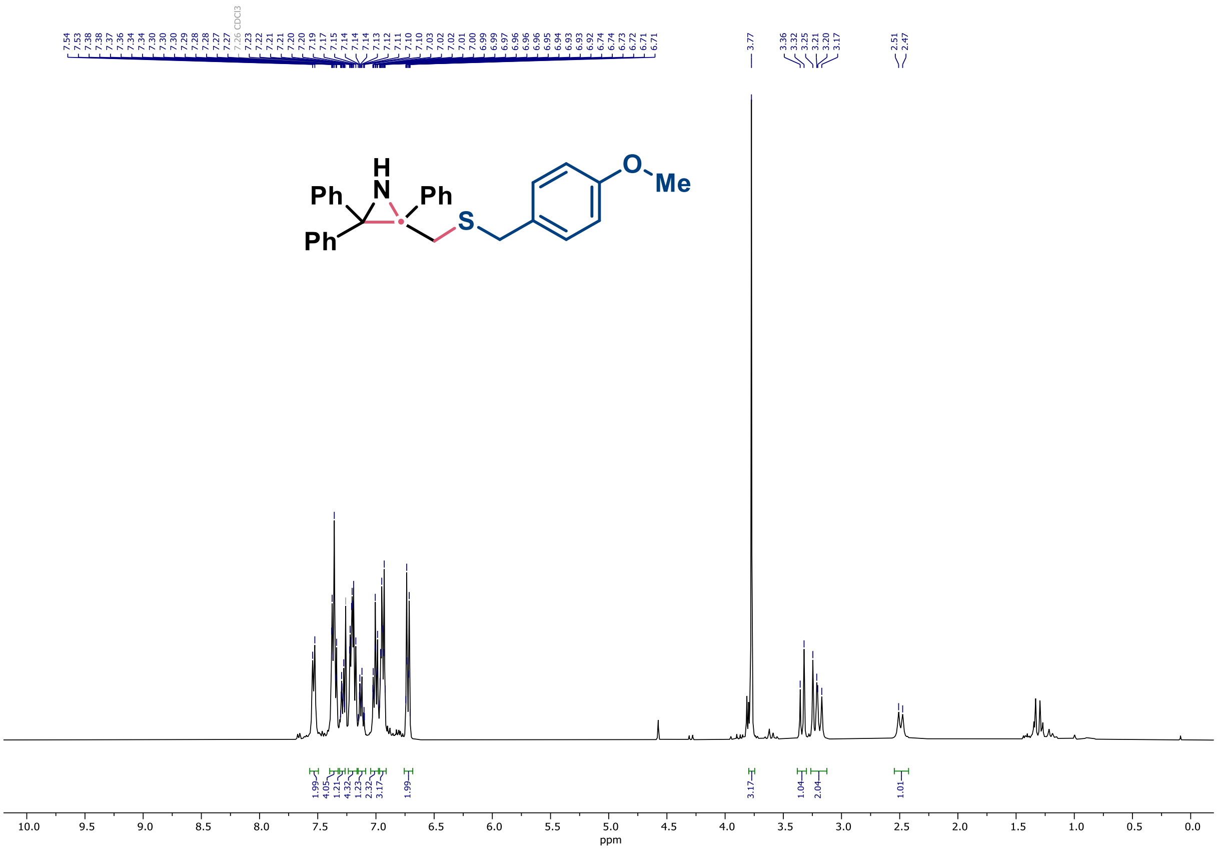

^{13}C{^{1}H} NMR (CDCl$_3$, 101 MHz) for **3j**

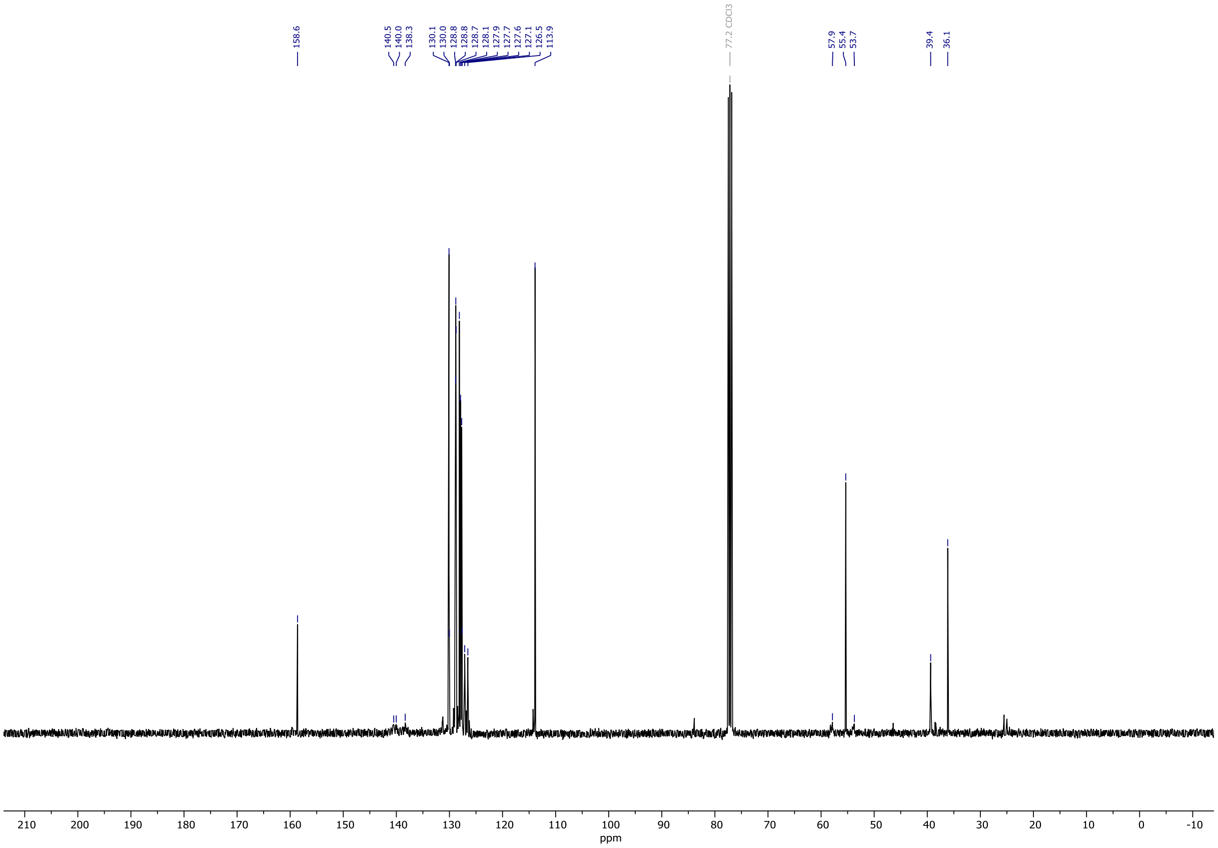

^{1}H NMR ($CDCl_3$, 400 MHz) for **3k**

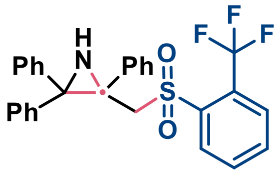

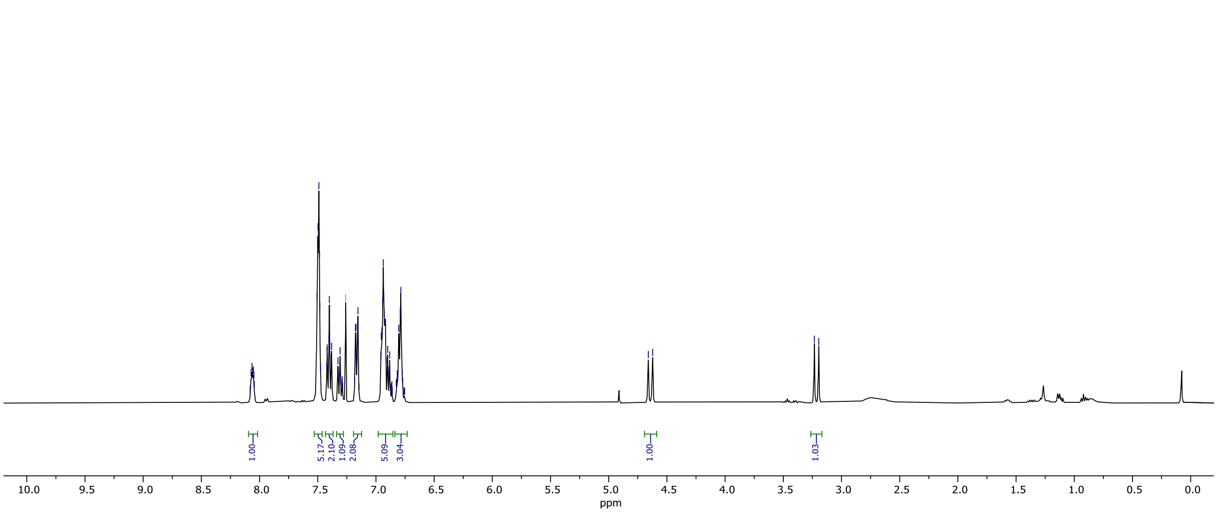

^{13}C{^{1}H} NMR ($CDCl_3$, 126 MHz) for **3k**

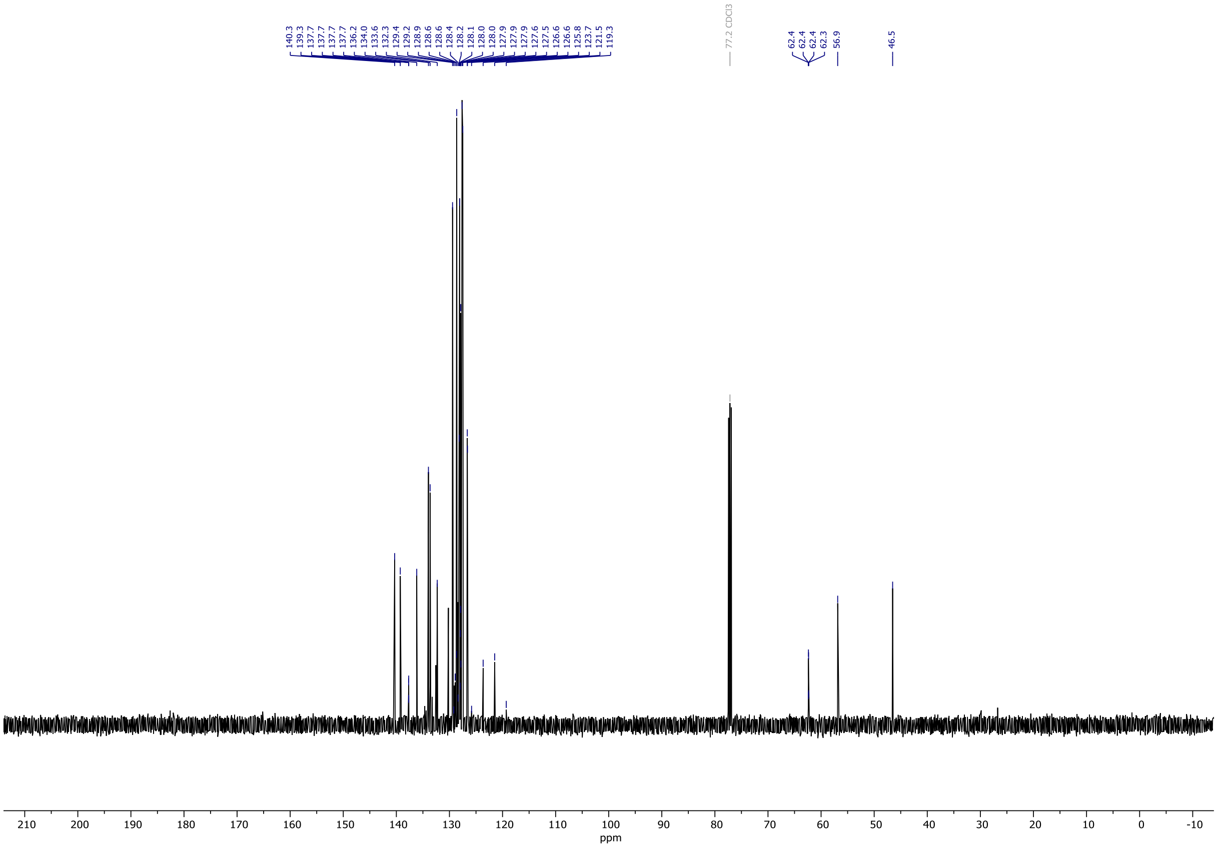

$^{19}F\{^{1}H\}$ NMR ($CDCl_3$, 470 MHz) for **3k**

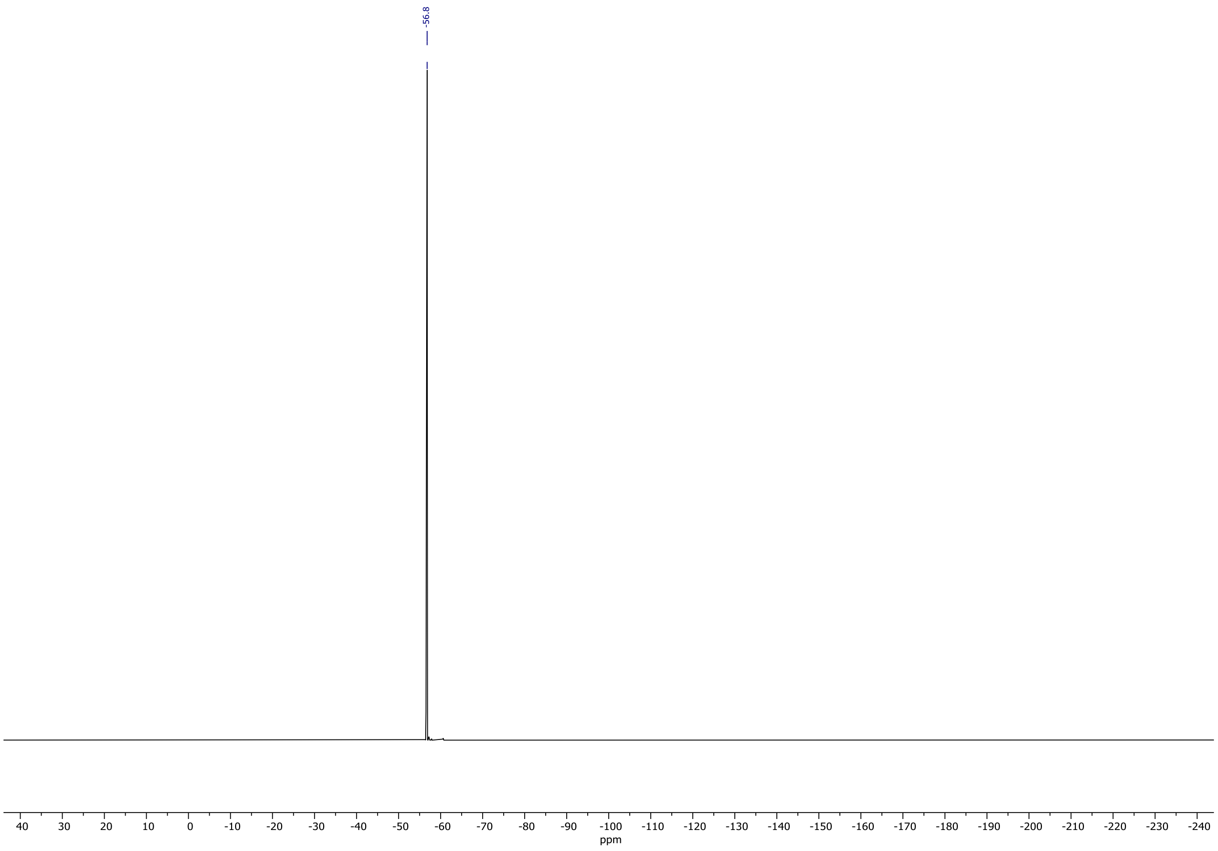

^{1}H NMR (CD_2Cl_2, 500 MHz) for **3l**

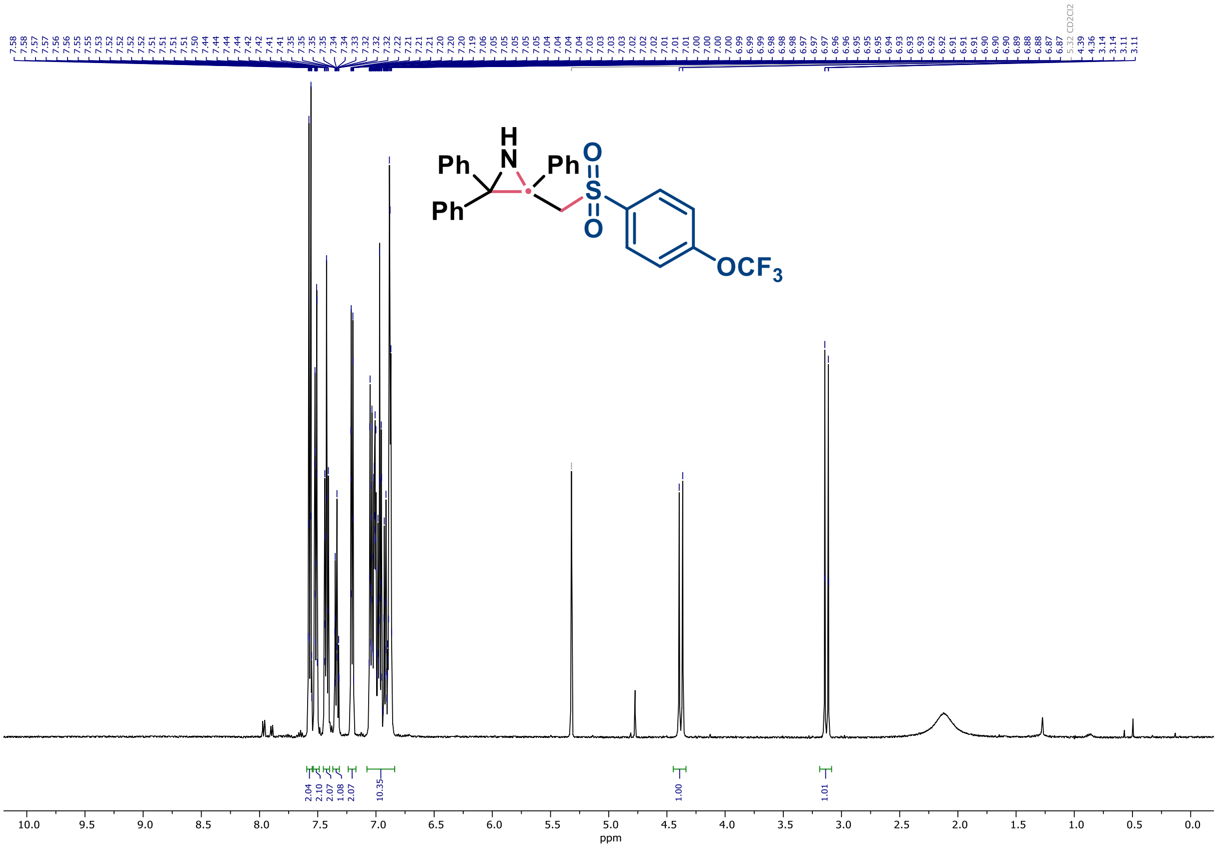

^{13}C{^{1}H} NMR (CD_2Cl_2, 126 MHz) for **3l**

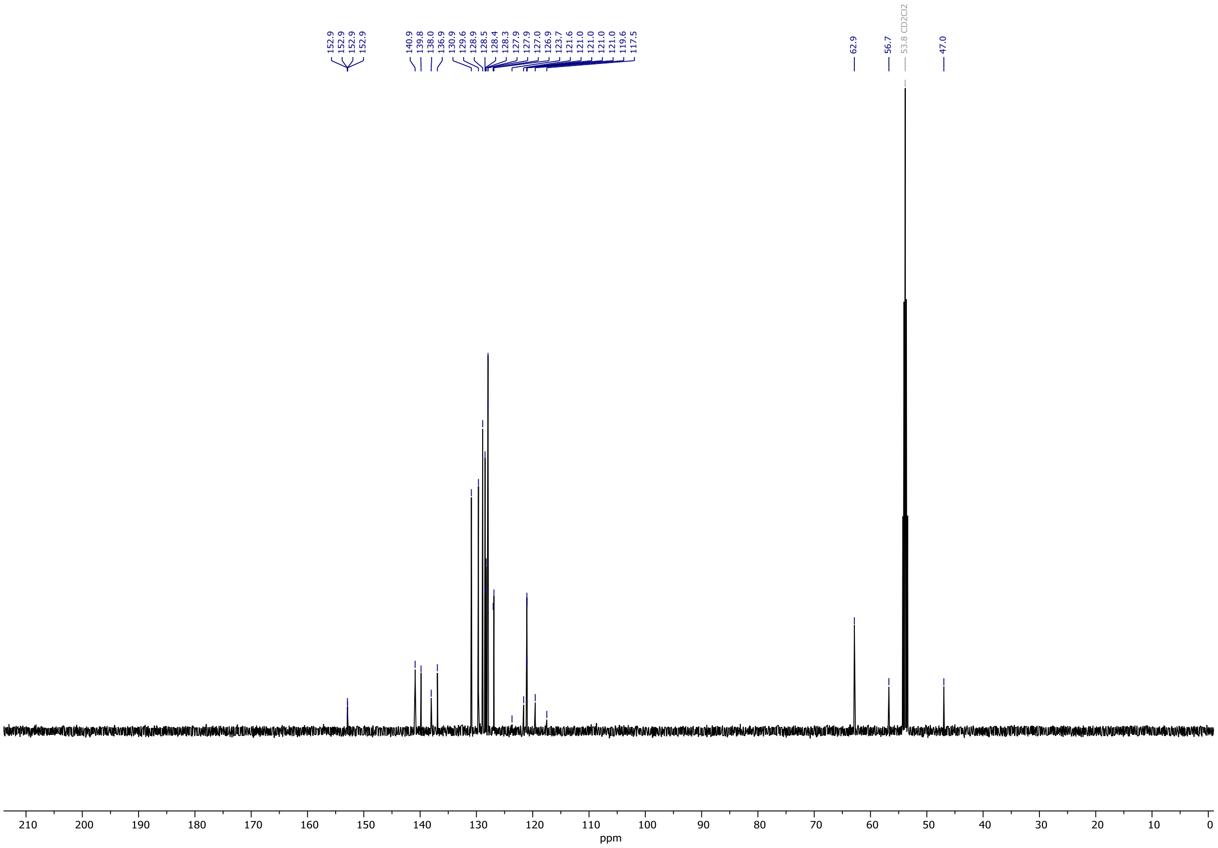

^{19}F{^{1}H} NMR (CD_2Cl_2, 377 MHz) for **3l**

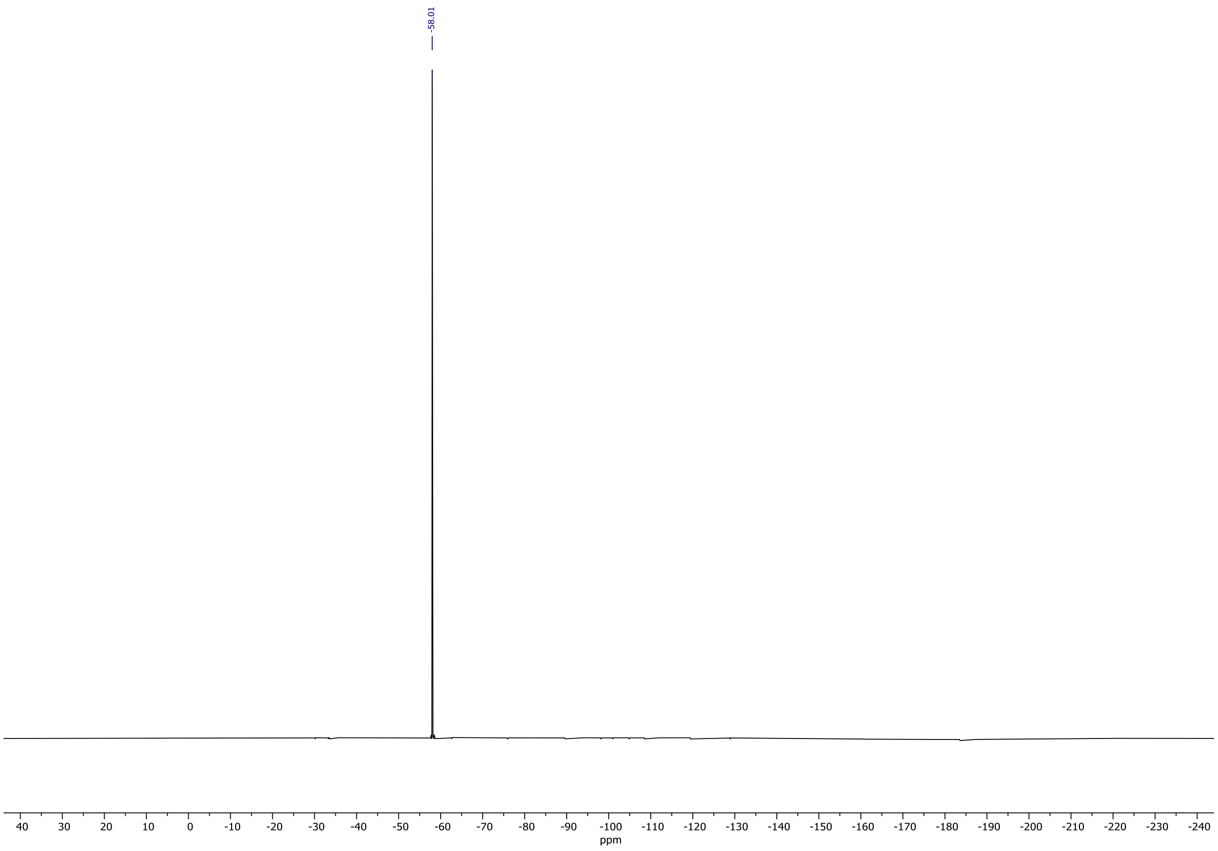

^{1}H NMR (CD_2Cl_2, 500 MHz) for **3m**

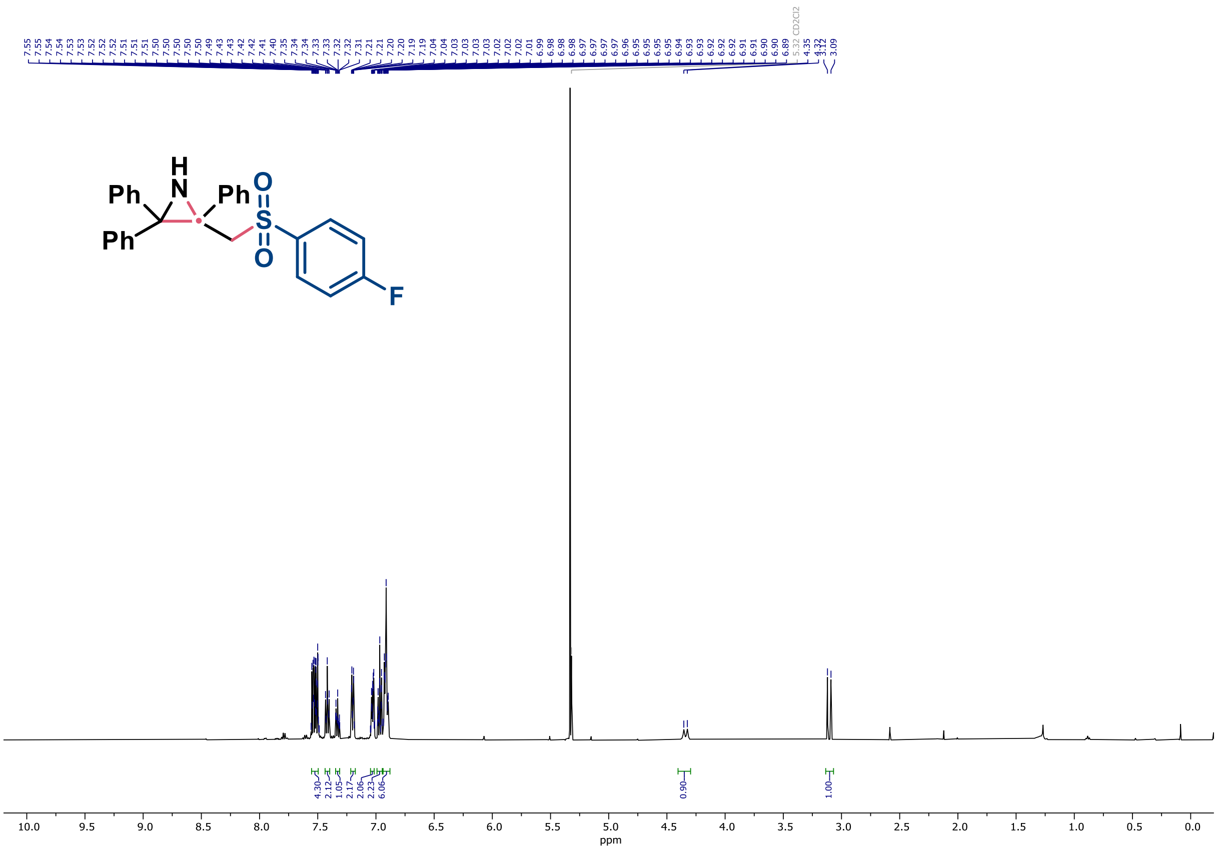

^{13}C{^{1}H} NMR (CD_2Cl_2, 126 MHz) for **3m**

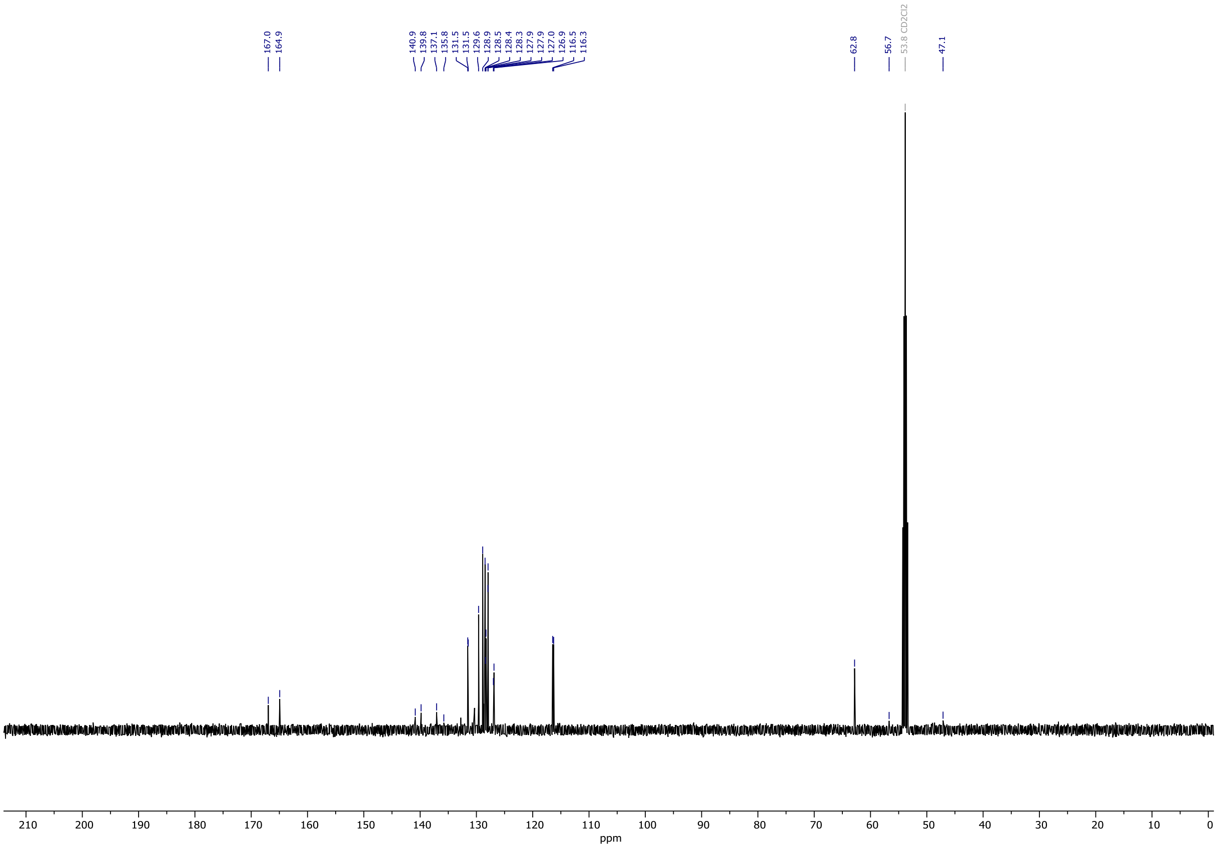

^{19}F{^{1}H} NMR (CD_2Cl_2, 470 MHz) for **3m**

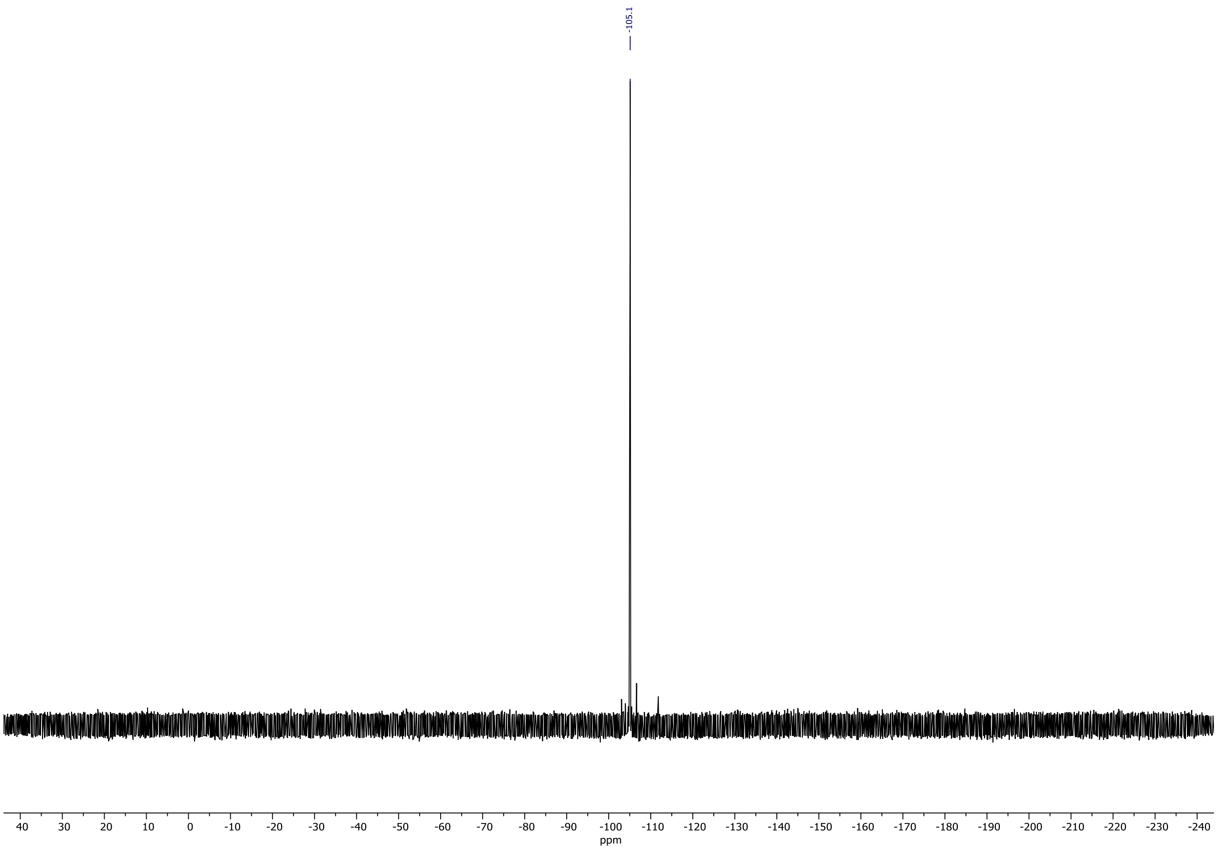

^{1}H NMR ($CDCl_3$, 400 MHz) for **3n**

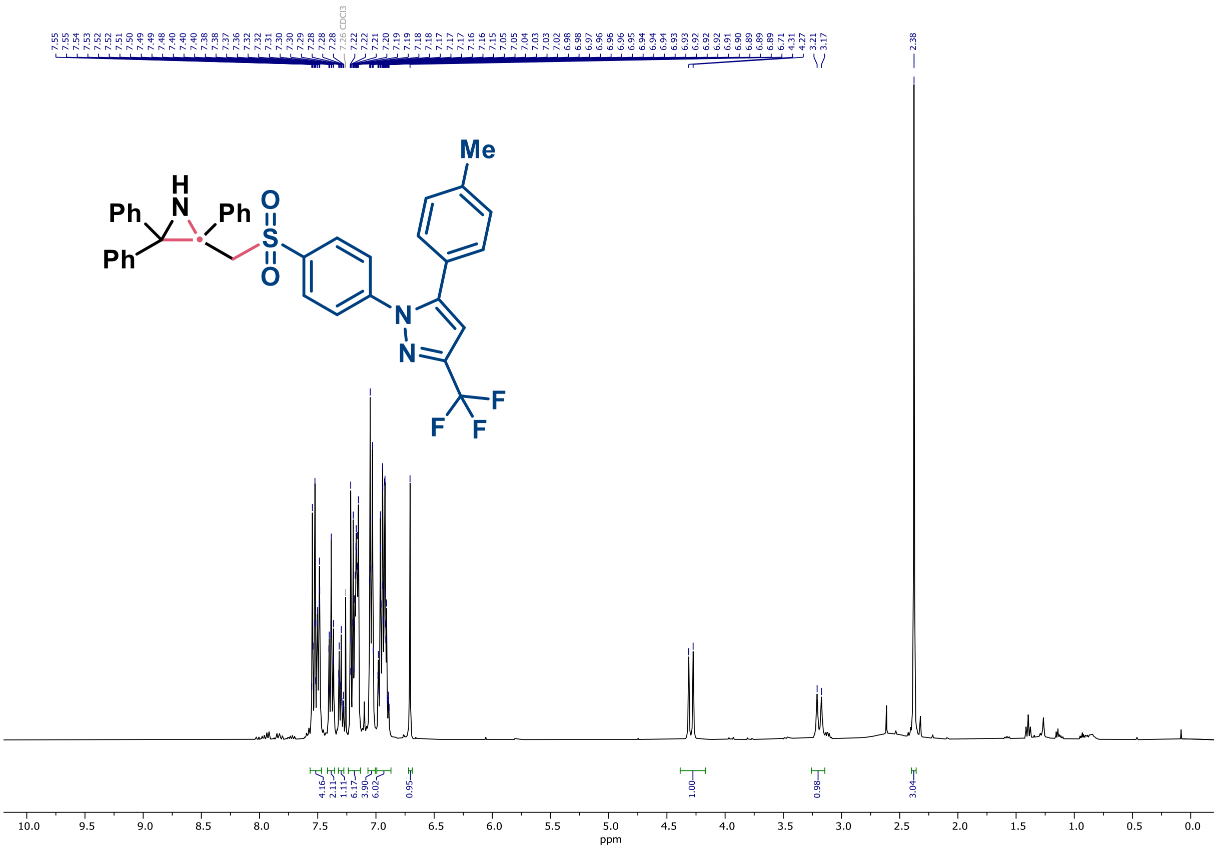

$^{13}C\{^{1}H\}$ NMR ($CDCl_3$, 101 MHz) for **3n**

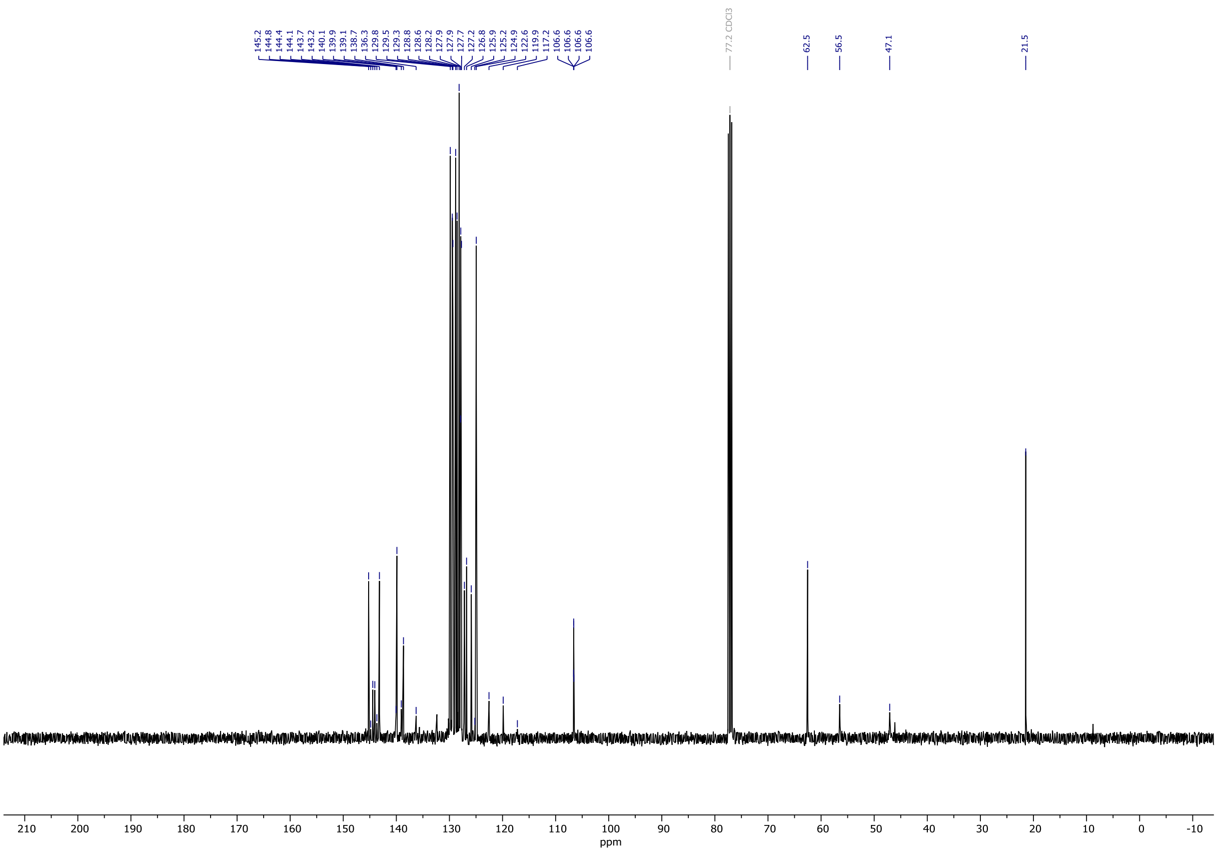

^{19}F{^{1}H} NMR ($CDCl_3$, 376 MHz) for **3n**

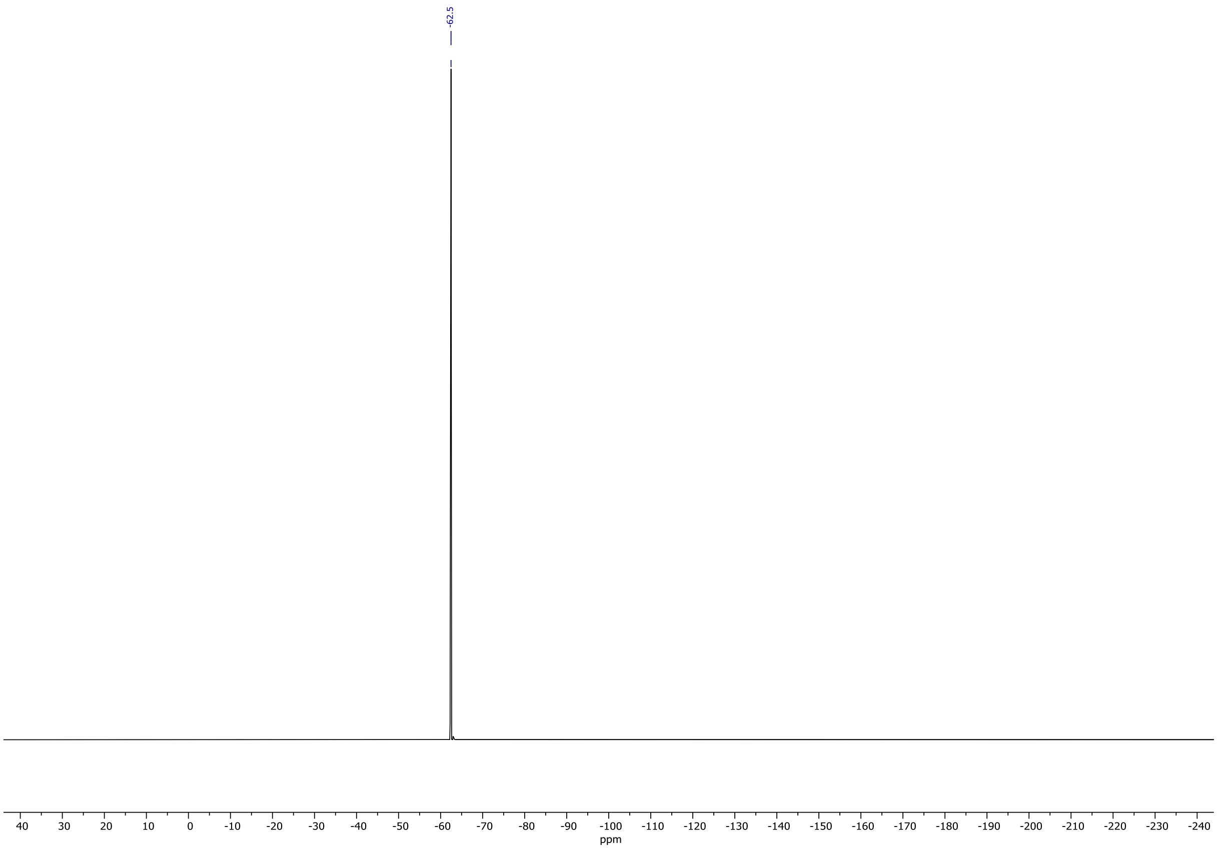

^{1}H NMR ($CDCl_3$, 400 MHz) for **3o**

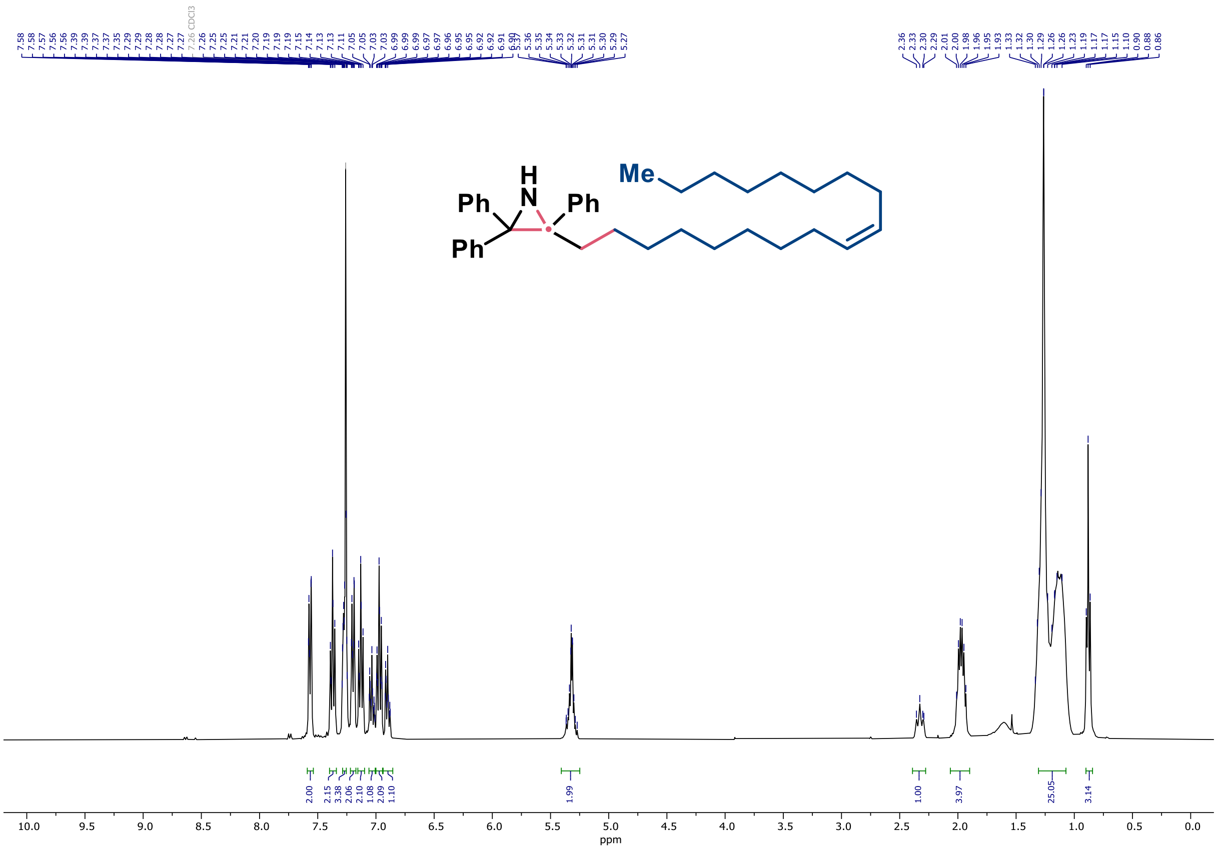

^{13}C{^{1}H} NMR ($CDCl_3$, 151 MHz) for **3o**

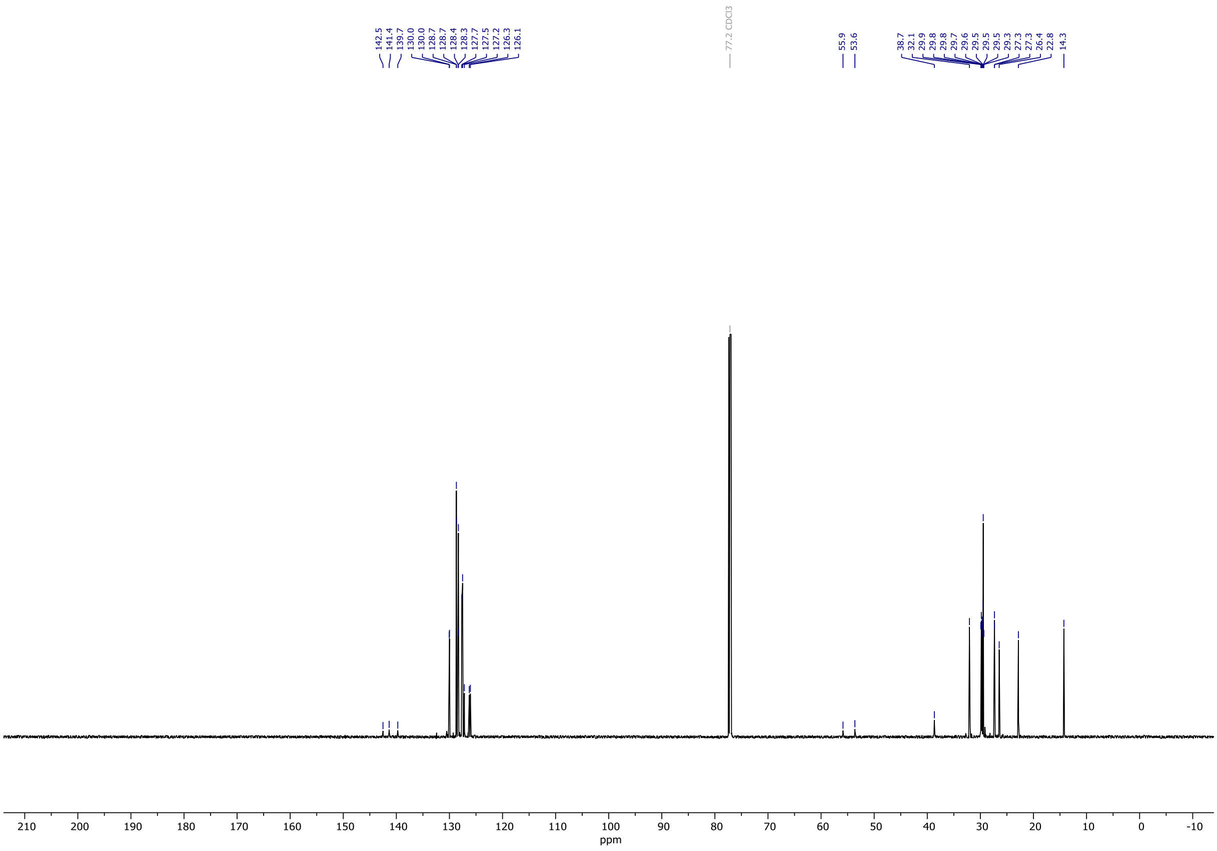

^{1}H NMR (CDCl$_3$, 599 MHz) for **3p**

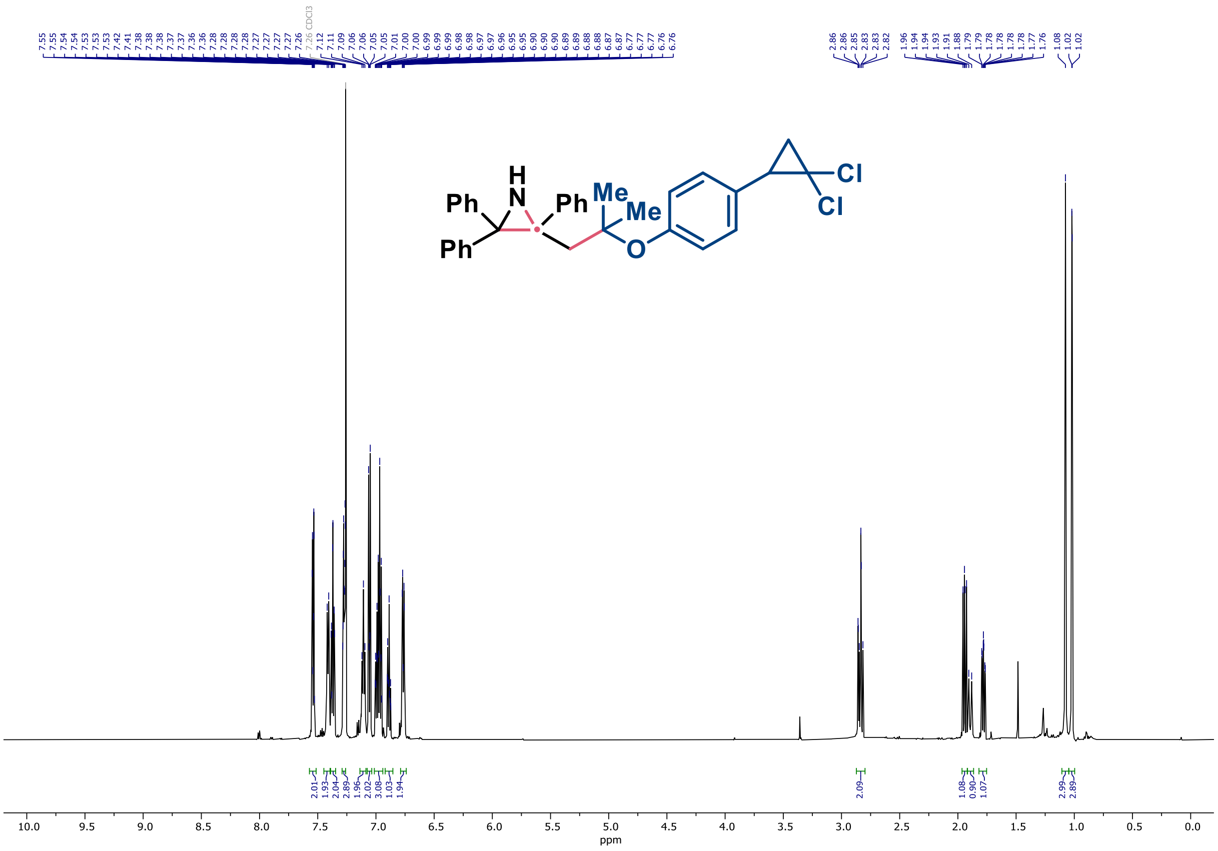

^{13}C{^{1}H} NMR (CDCl$_3$, 101 MHz) for **3p**

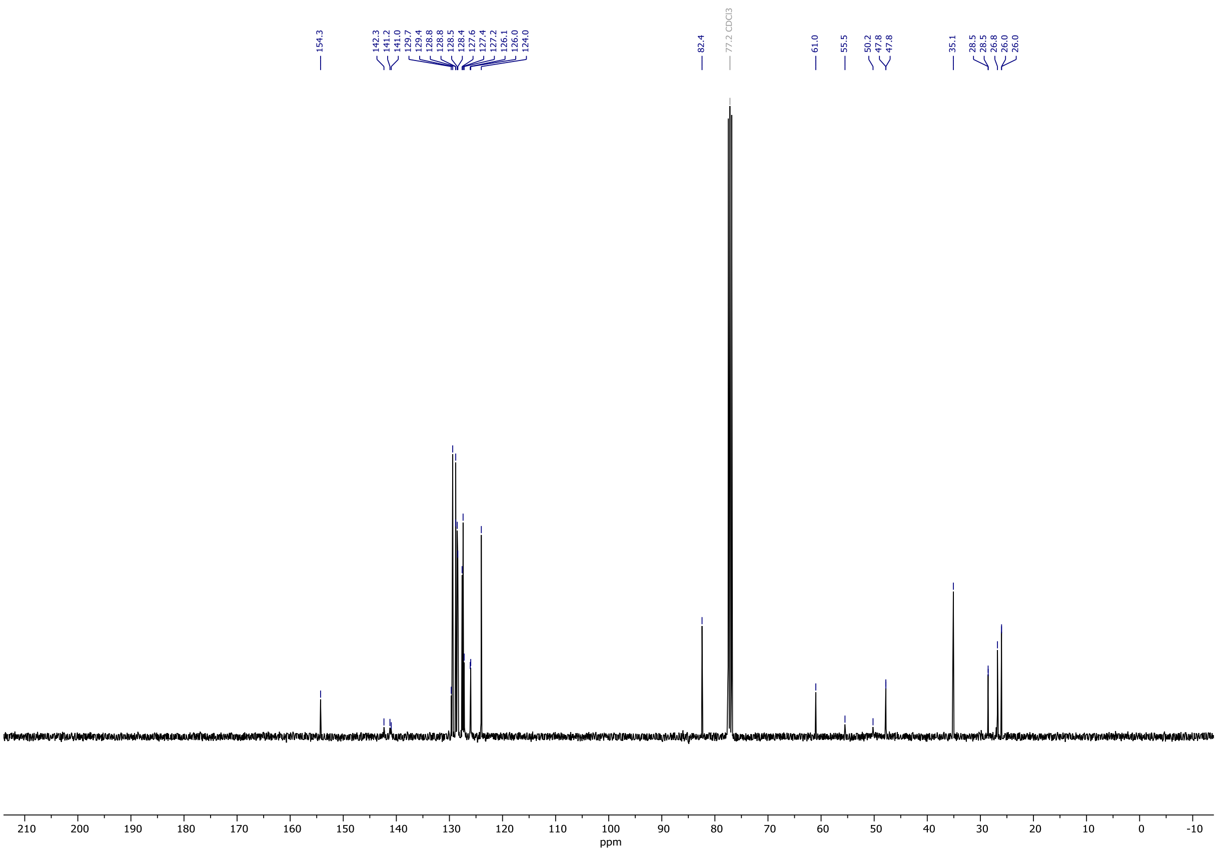

^{1}H NMR ($CDCl_3$, 400 MHz) for **3q**

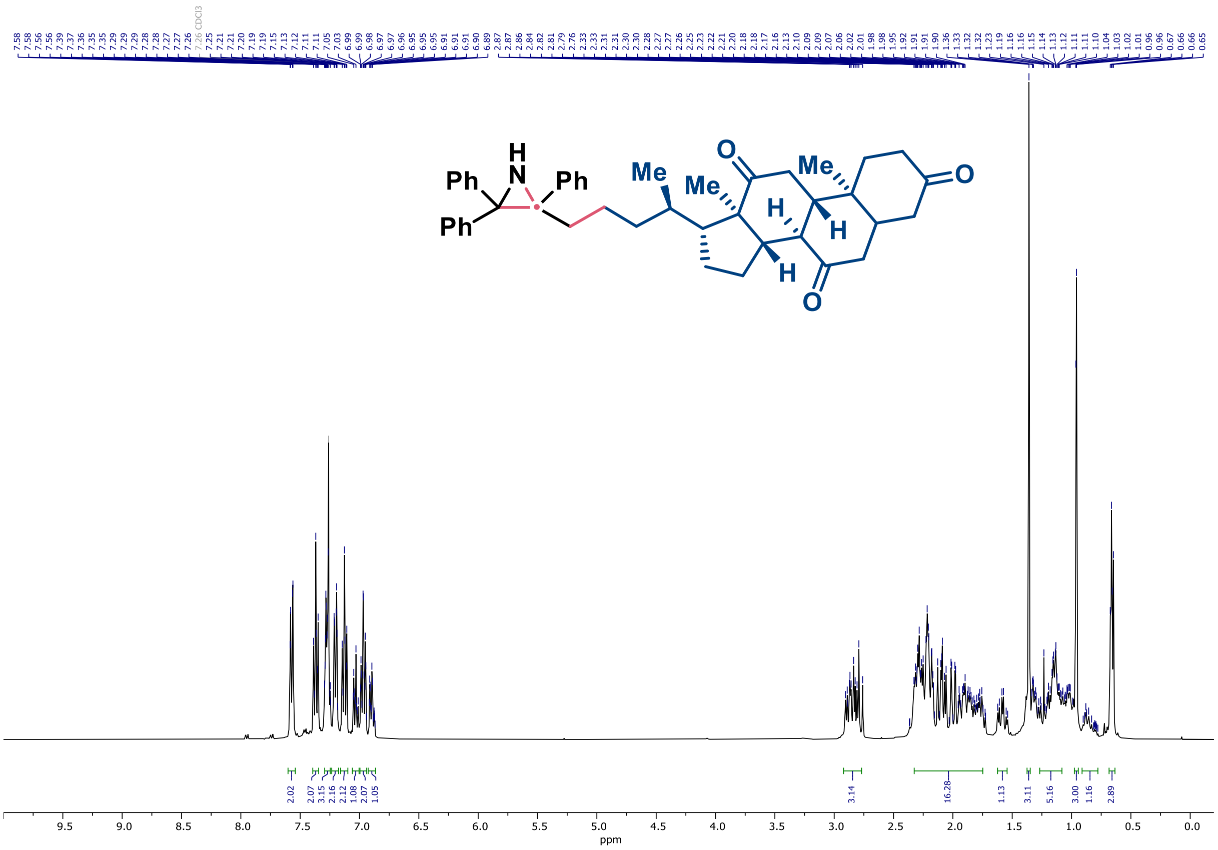

^{13}C{^{1}H} NMR ($CDCl_3$, 101 MHz) for **3q**

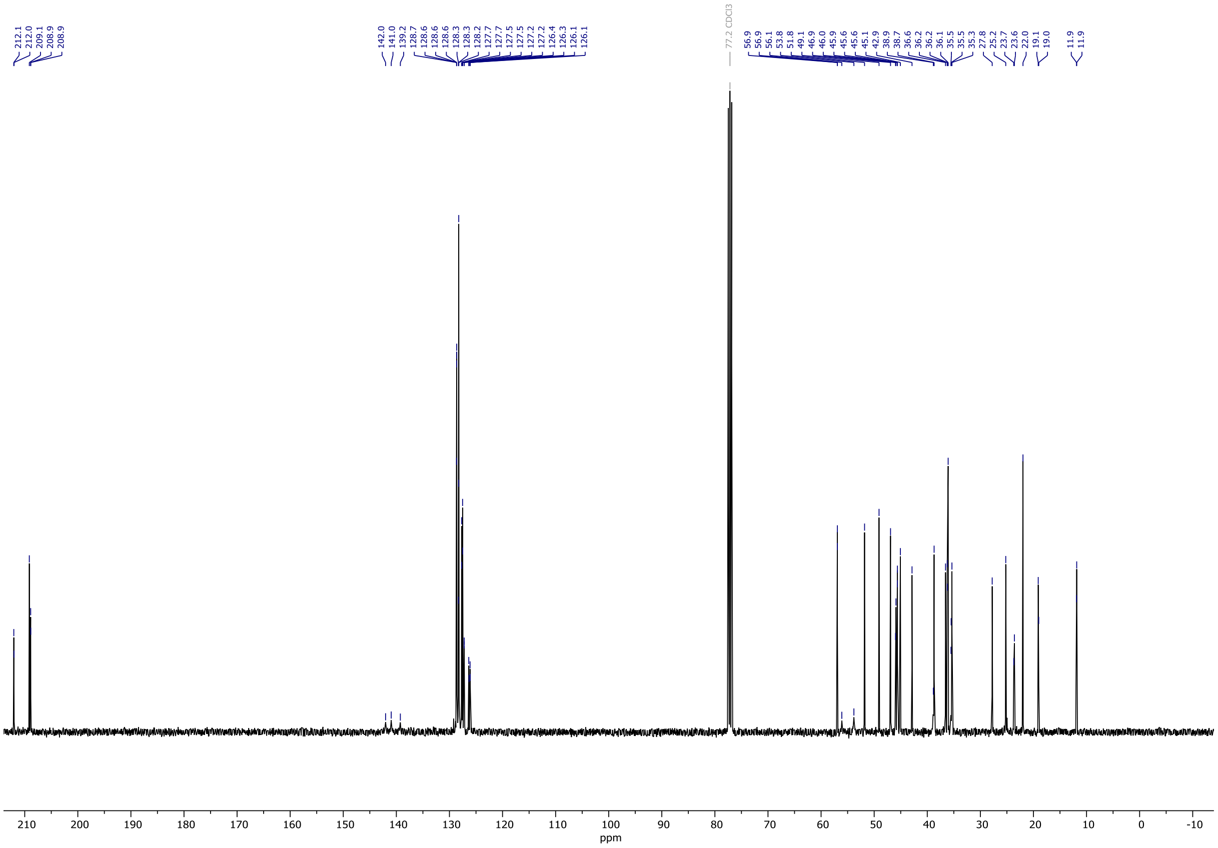

¹H NMR (CDCl3, 400 MHz) for **3r**

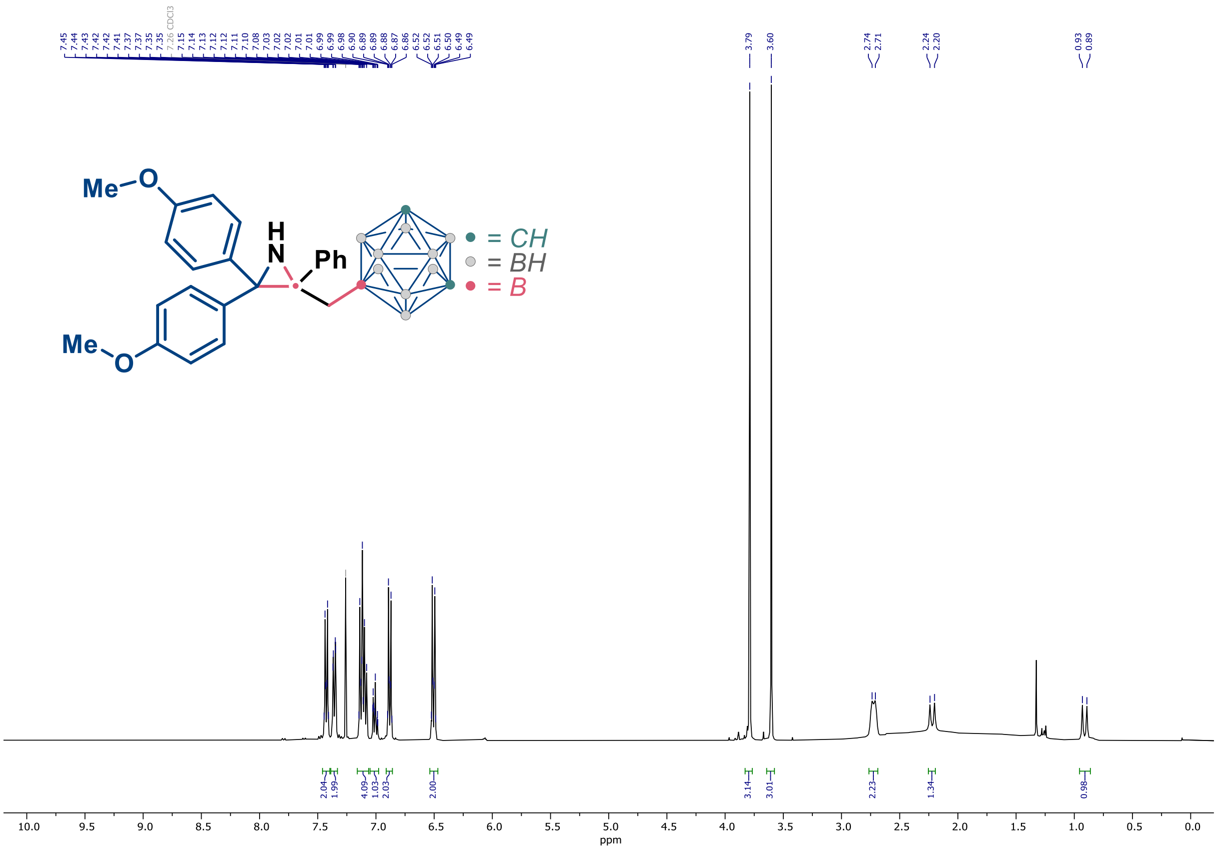

¹³C{¹H} NMR (CDCl3, 101 MHz) for **3r**

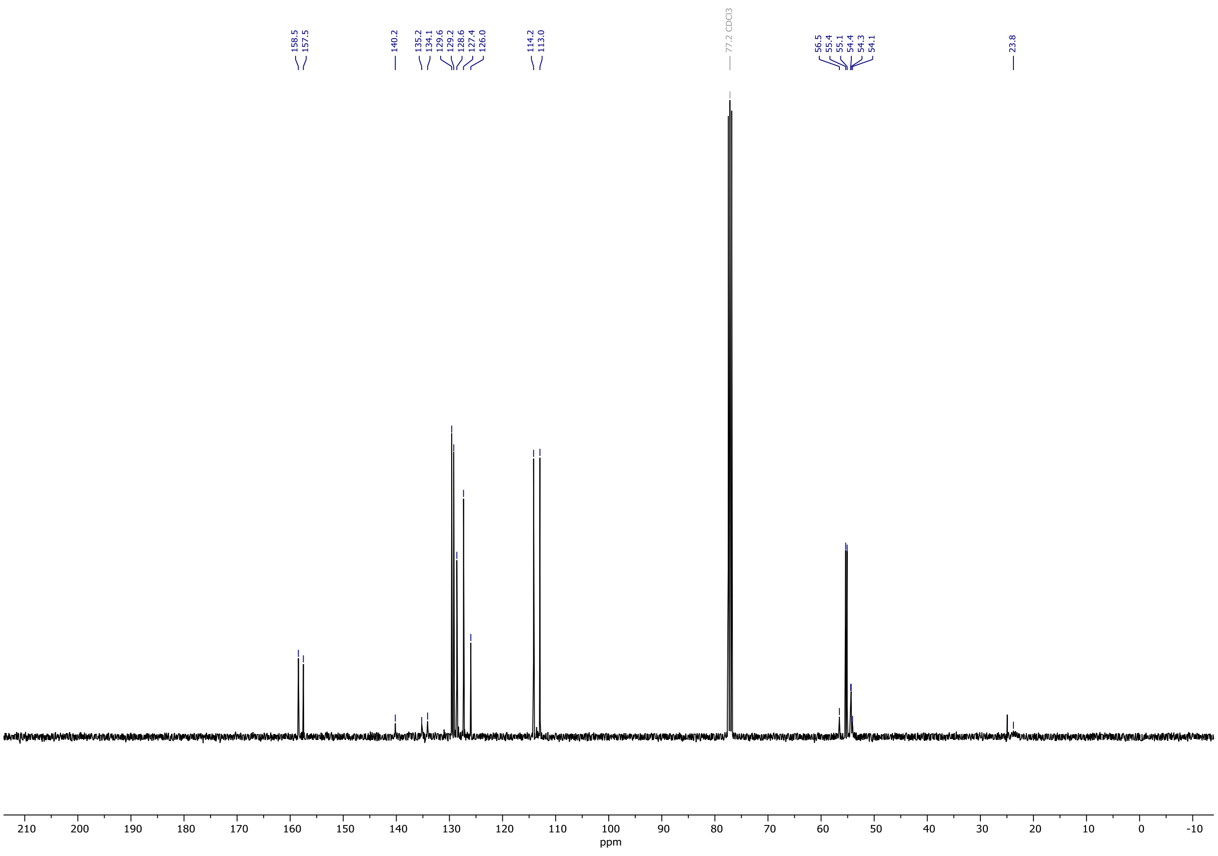

^{11}B{^{1}H} NMR ($CDCl_3$, 128 MHz) for **3r**

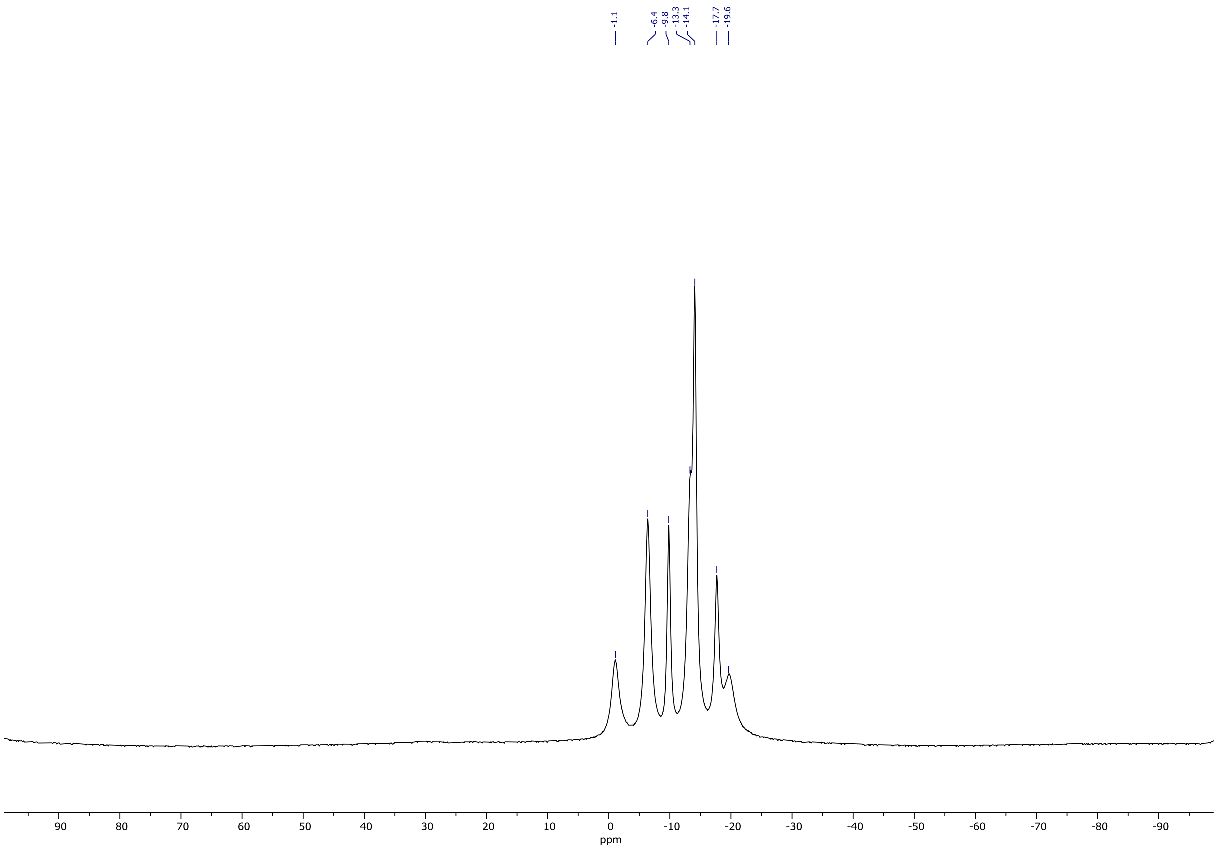

^{1}H NMR ($CDCl_3$, 400 MHz) for **3s**

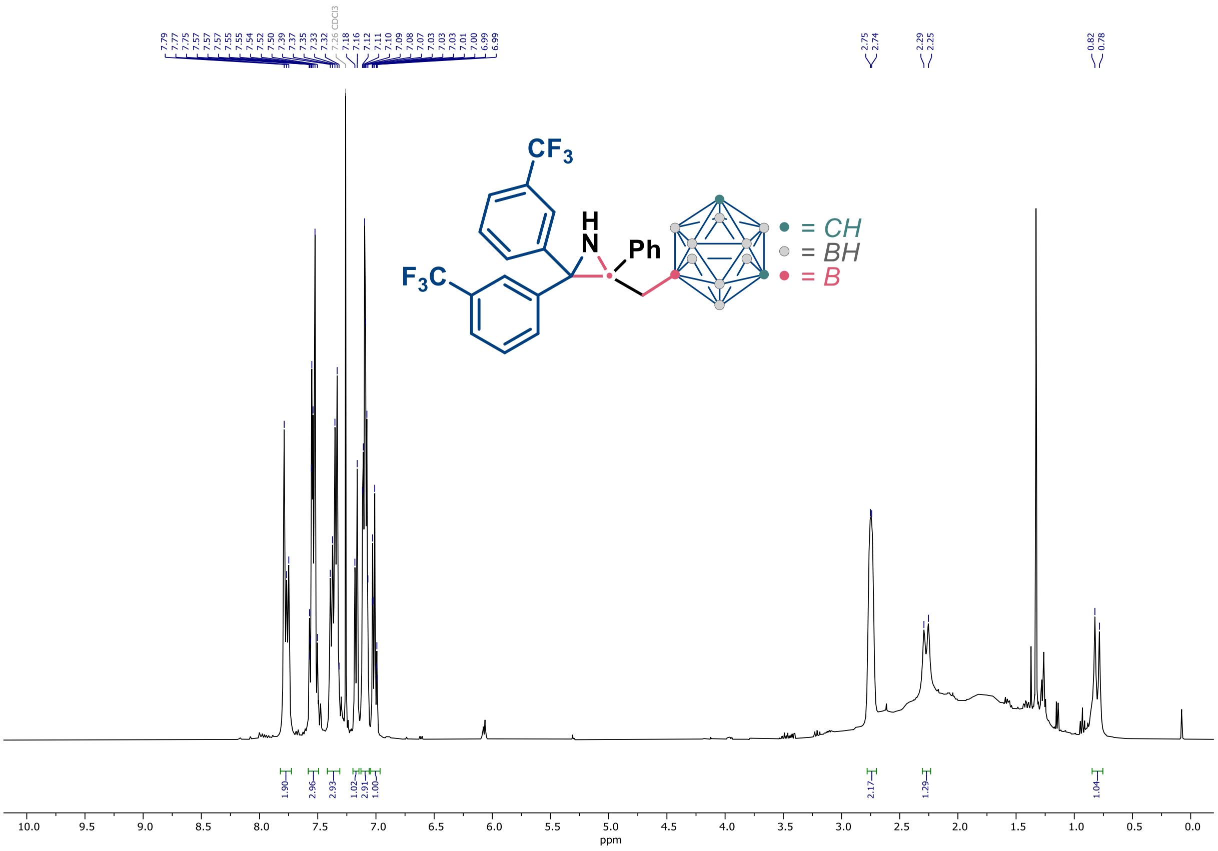

^{13}C{^{1}H} NMR ($CDCl_3$, 101 MHz) for **3s**

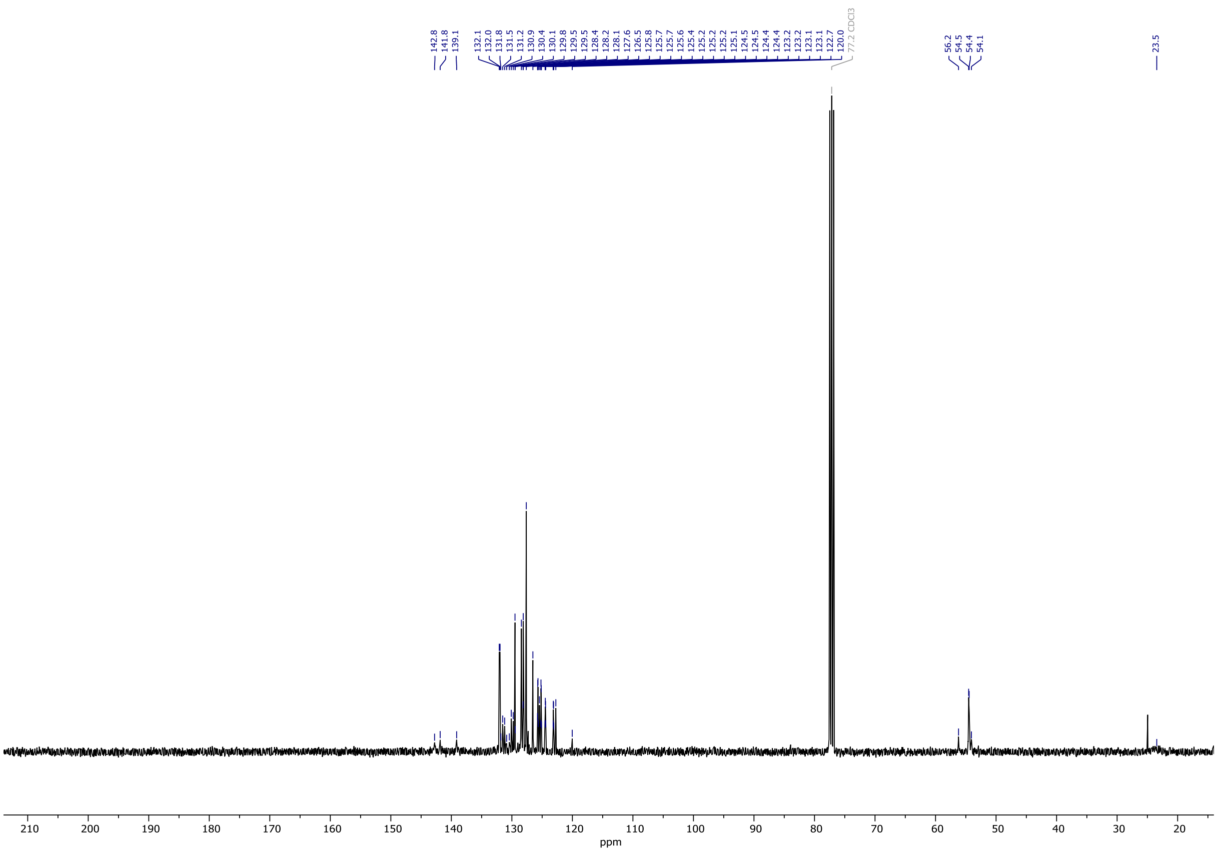

^{11}B{^{1}H} NMR (CDCl$_3$, 128 MHz) for **3s**

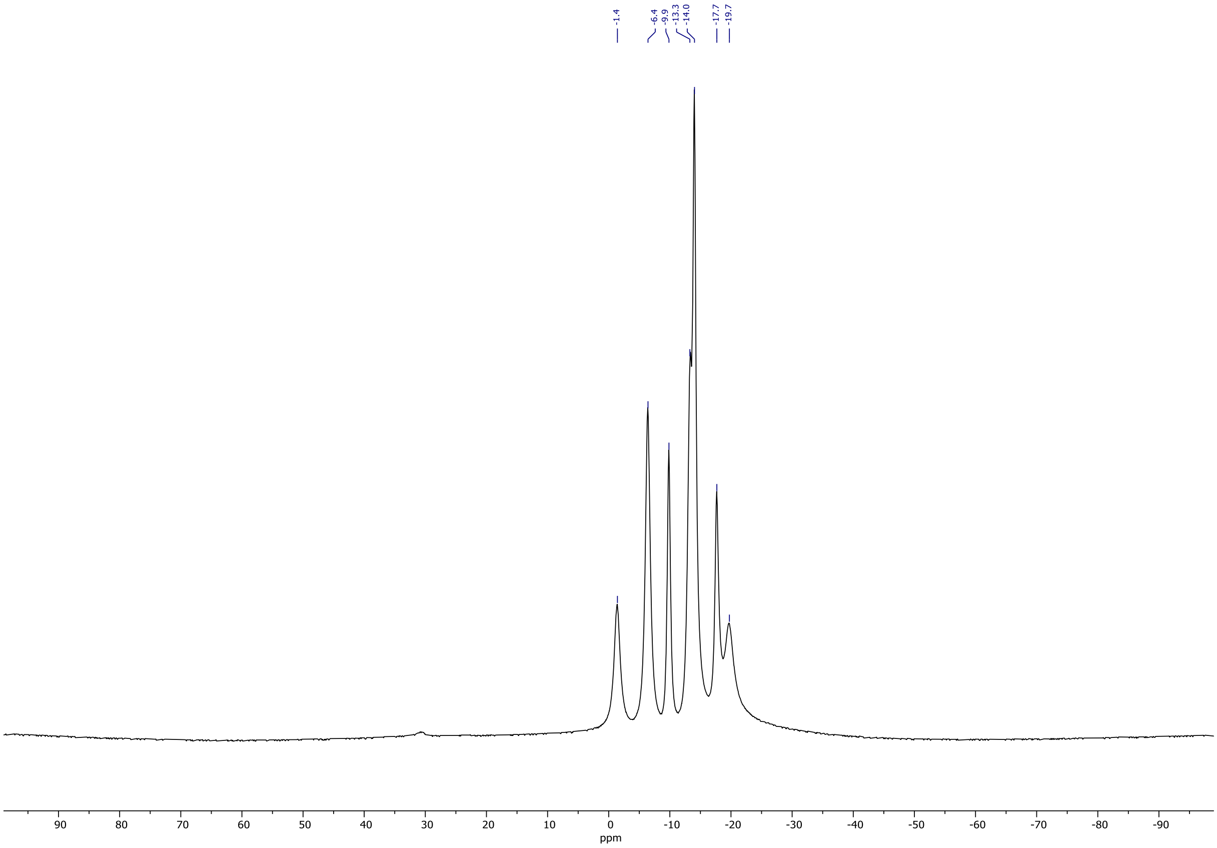

^{19}F{^{1}H} NMR (CDCl$_3$, 376 MHz) for **3s**

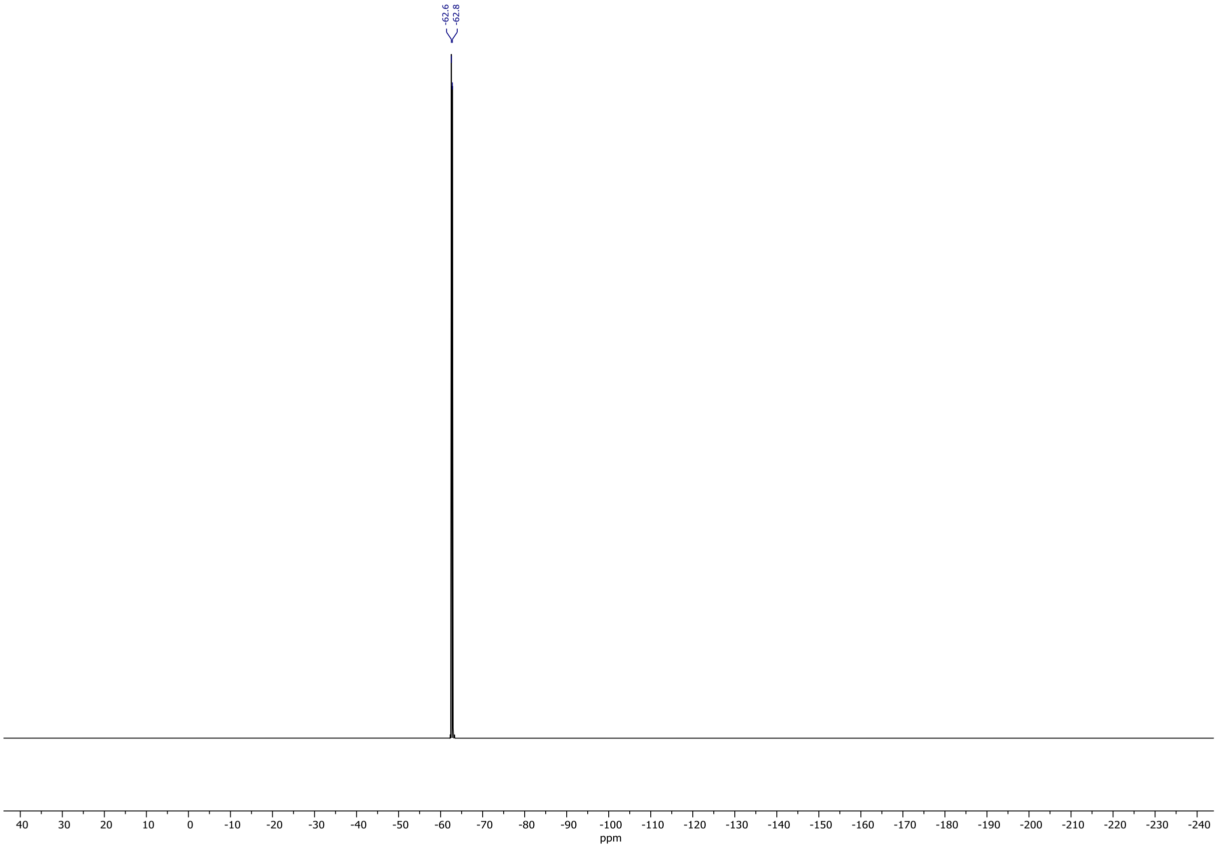

^{1}H NMR ($CDCl_3$, 400 MHz) for **3t**

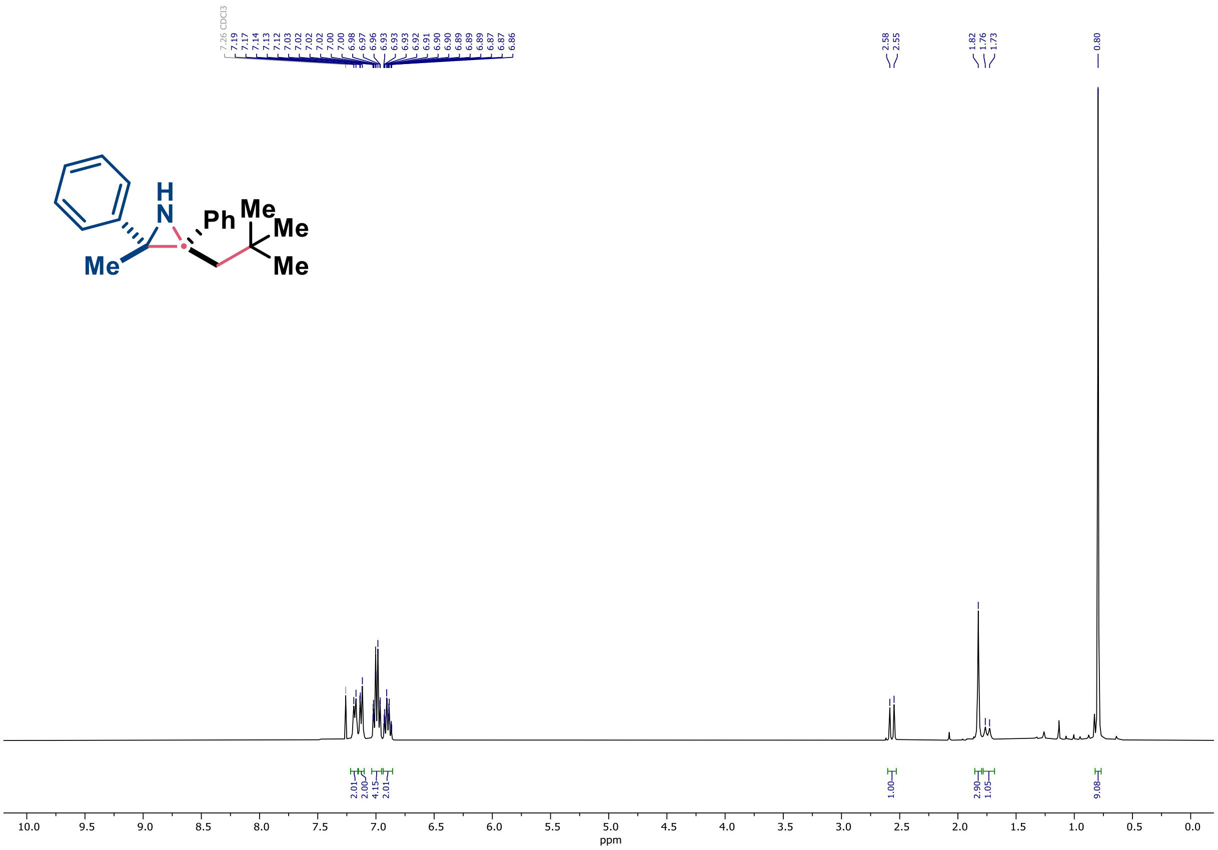

^{13}C{^{1}H} NMR ($CDCl_3$, 101 MHz) for **3t**

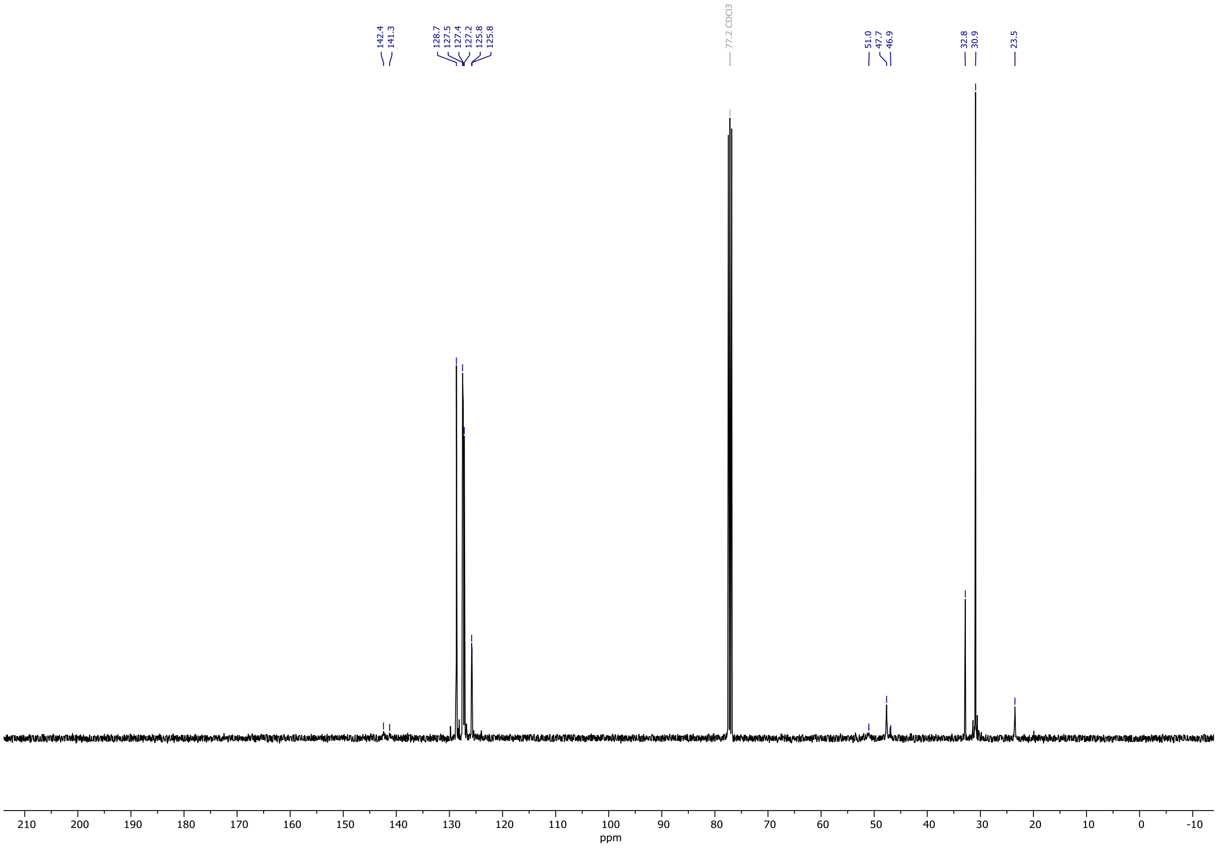

^{1}H NMR ($CDCl_3$, 400 MHz) for **3u (major diastereomer)**

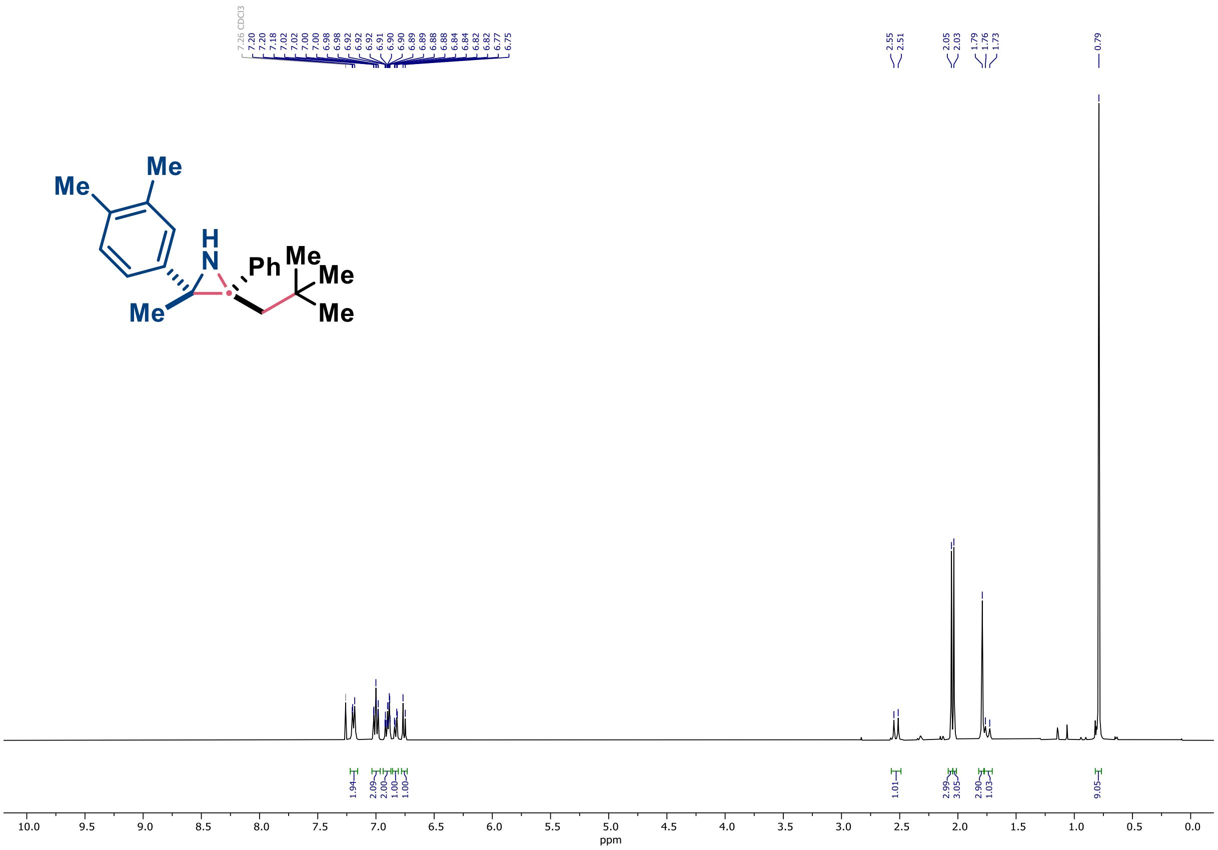

$^{13}C\{^{1}H\}$ NMR ($CDCl_3$, 101 MHz) for **3u (major diastereomer)**

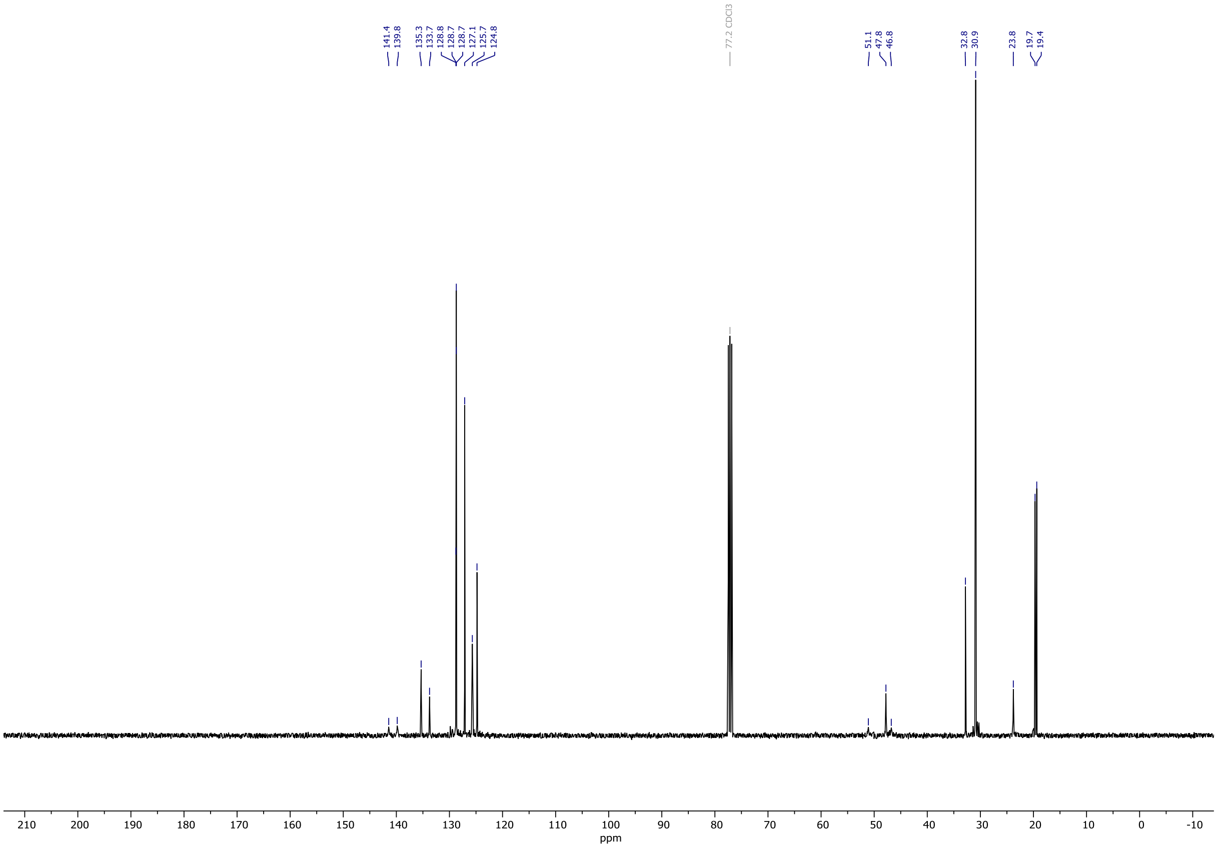

^{1}H NMR ($CDCl_3$, 400 MHz) for **3u (minor diastereomer)**

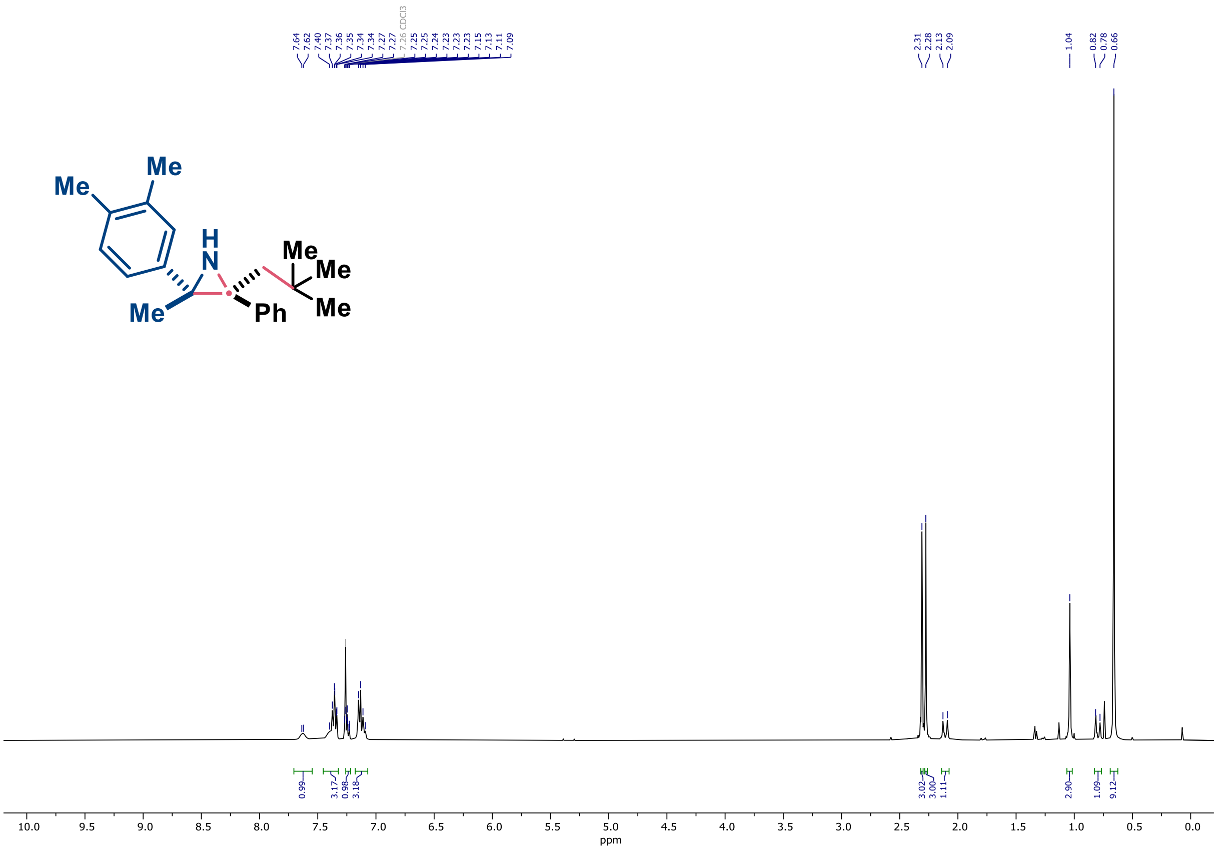

$^{13}C\{^{1}H\}$ NMR ($CDCl_3$, 101 MHz) for **3u (minor diastereomer)**

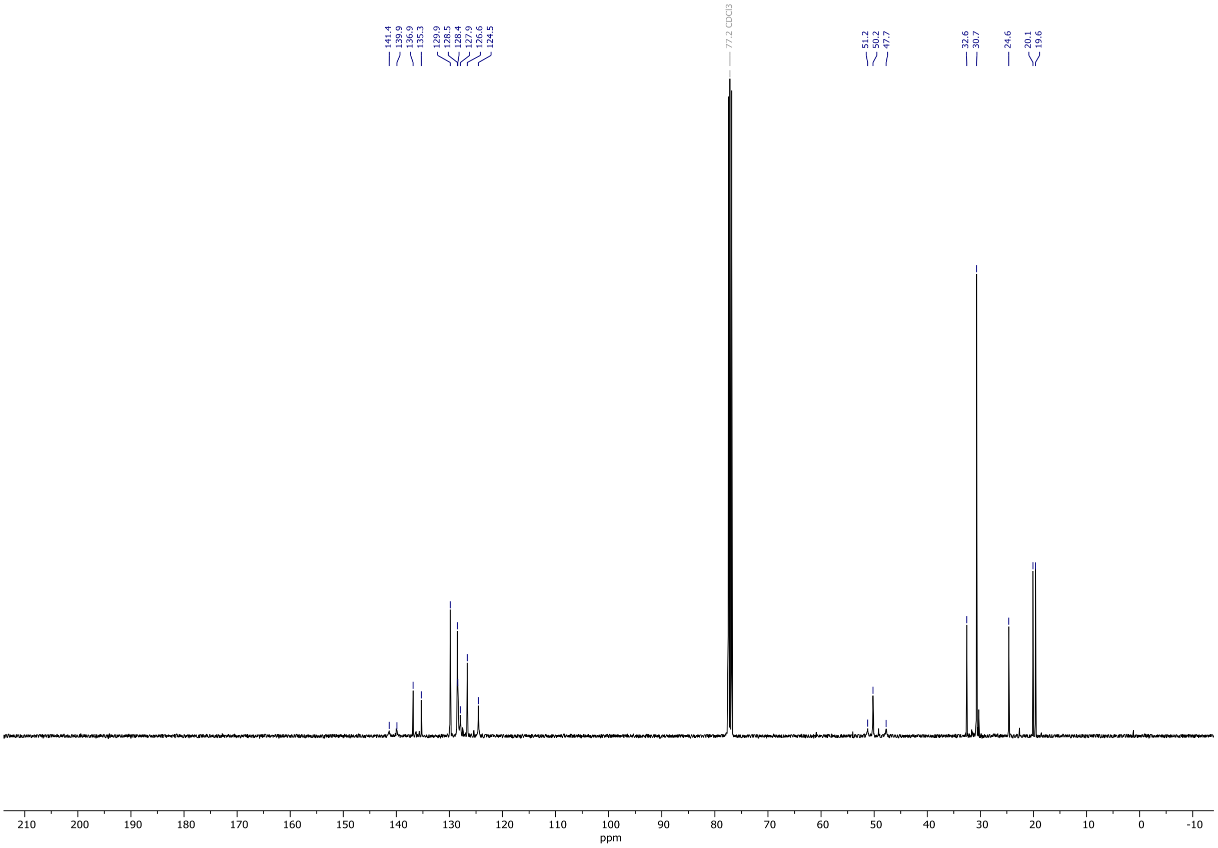

^{1}H NMR ($CDCl_3$, 500 MHz) for **3v**

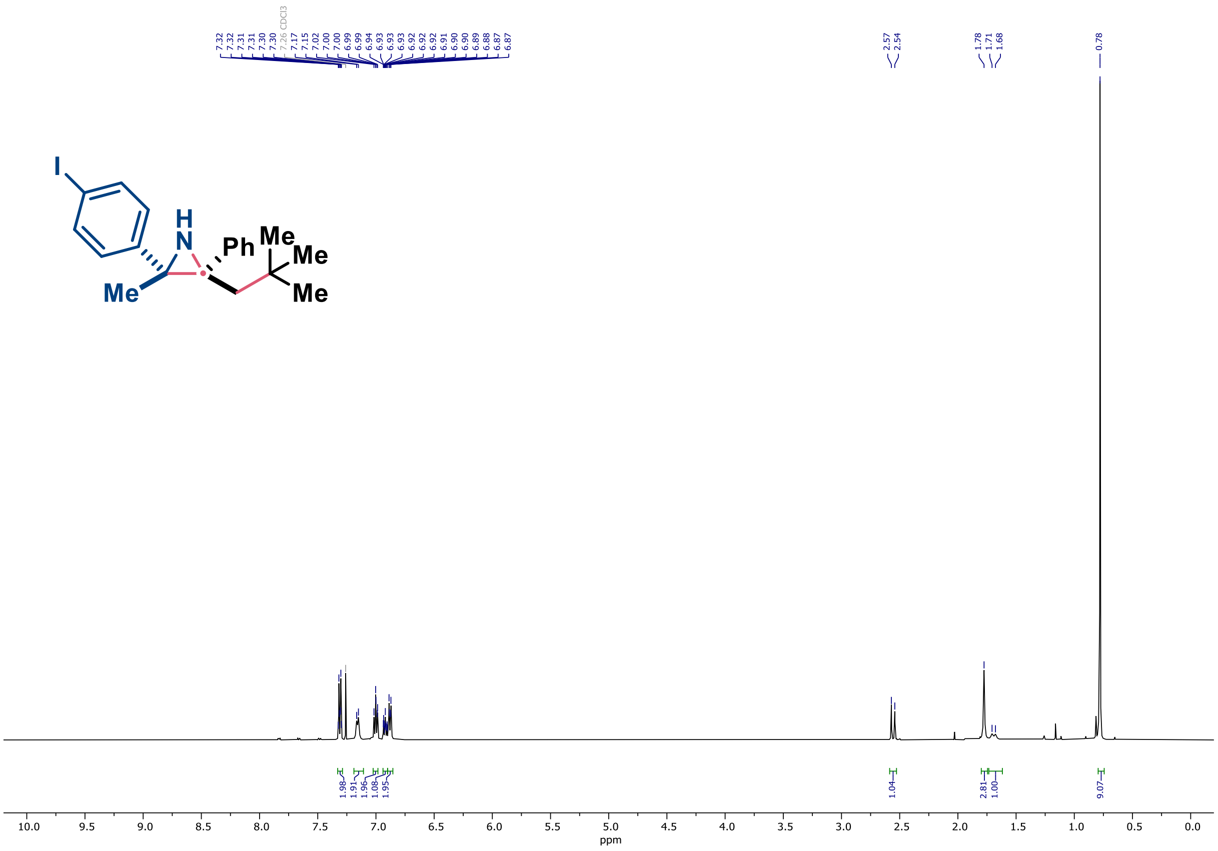

^{13}C{^{1}H} NMR ($CDCl_3$, 126 MHz) for **3v**

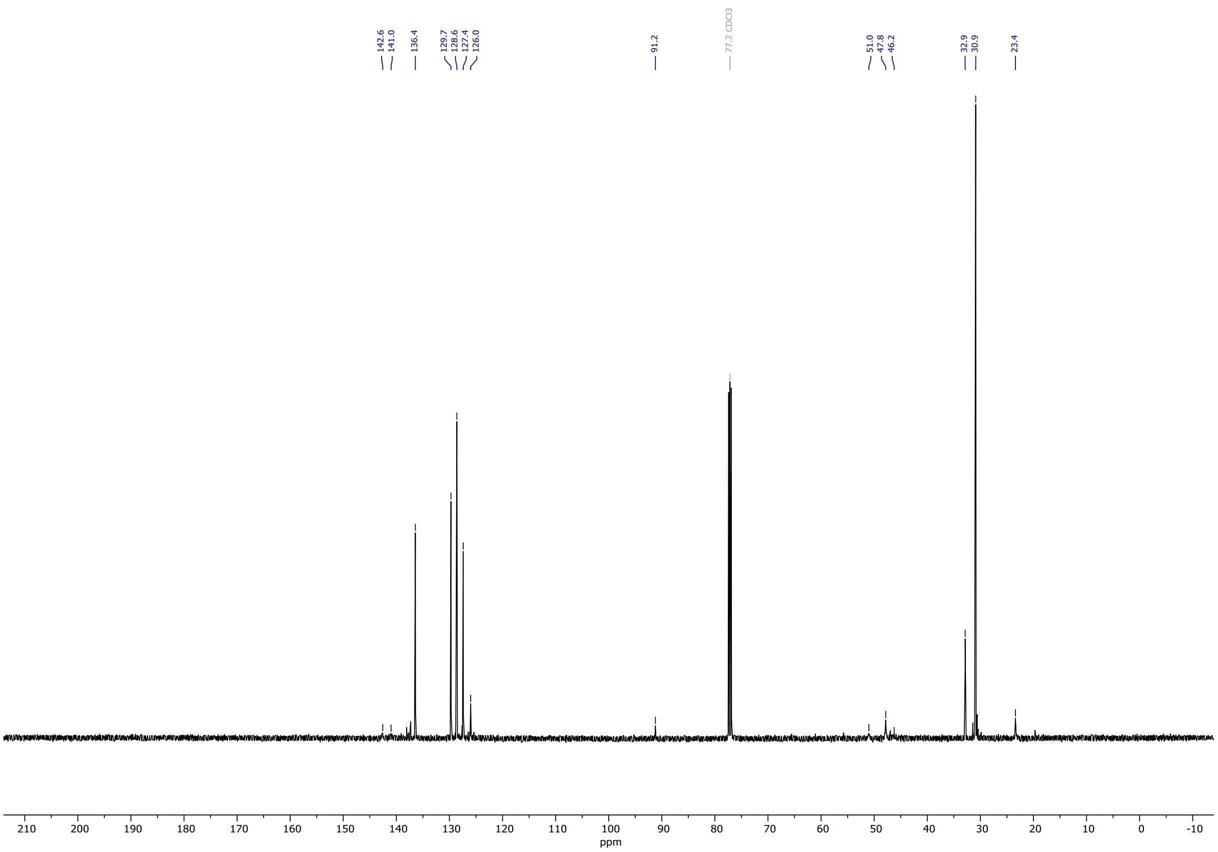

^{1}H NMR ($CDCl_3$, 400 MHz) for **3w (major diastereomer)**

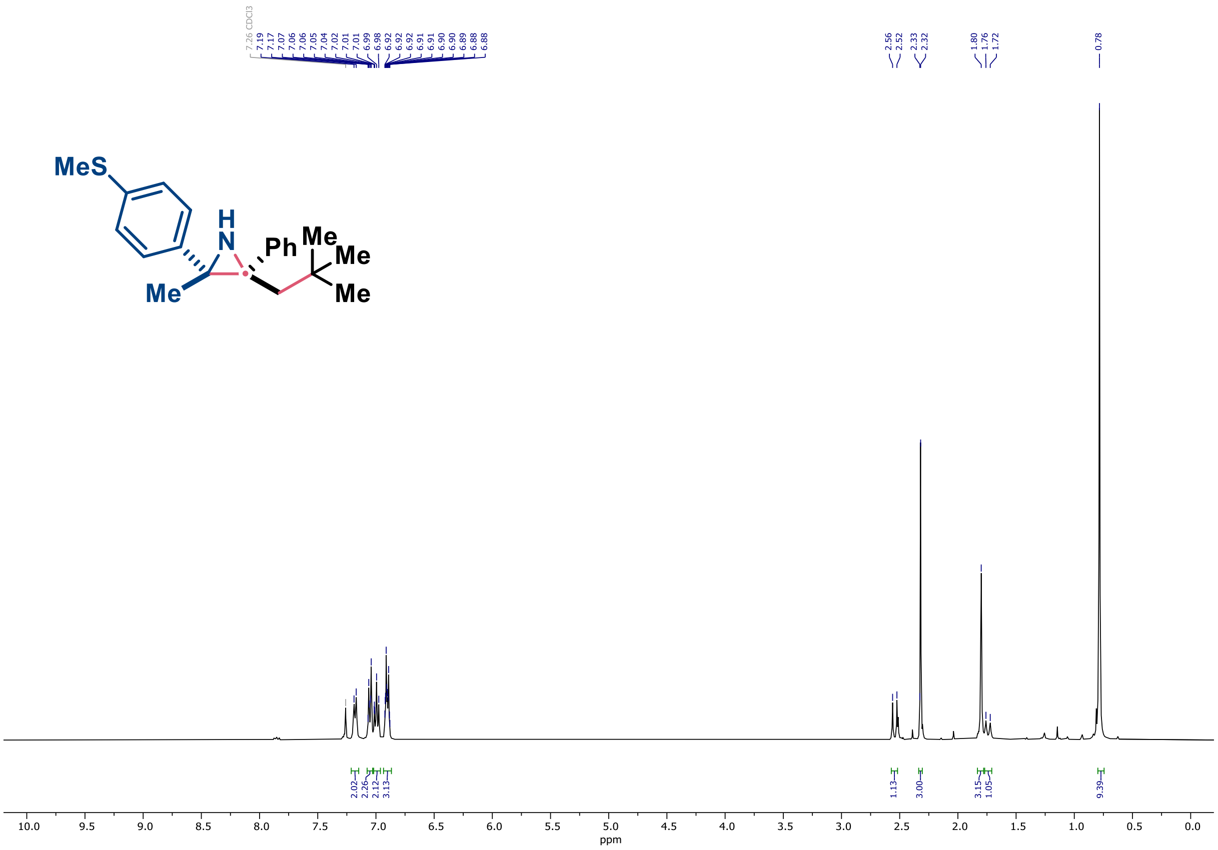

^{13}C{^{1}H} NMR ($CDCl_3$, 101 MHz) for **3w (major diastereomer)**

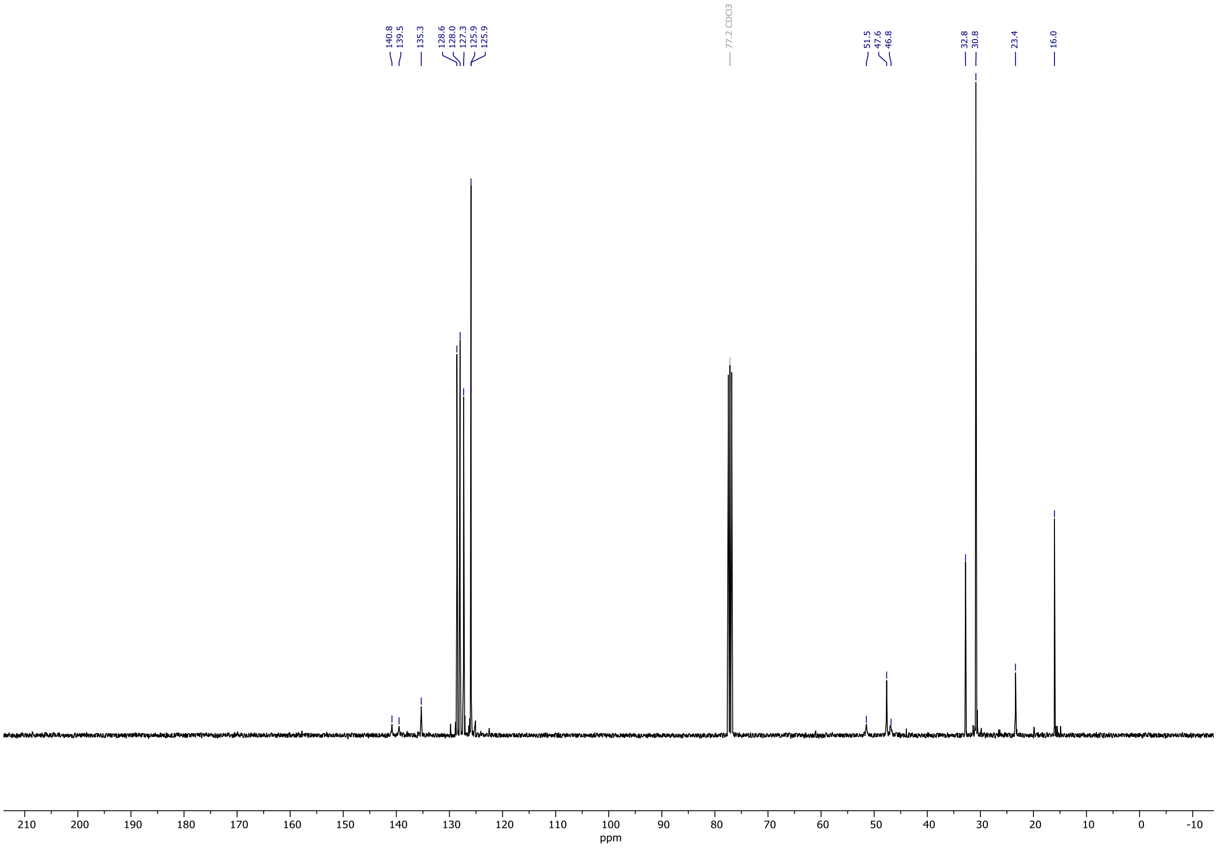

¹H NMR ($CDCl_3$, 400 MHz) for **3w (minor diastereomer)**

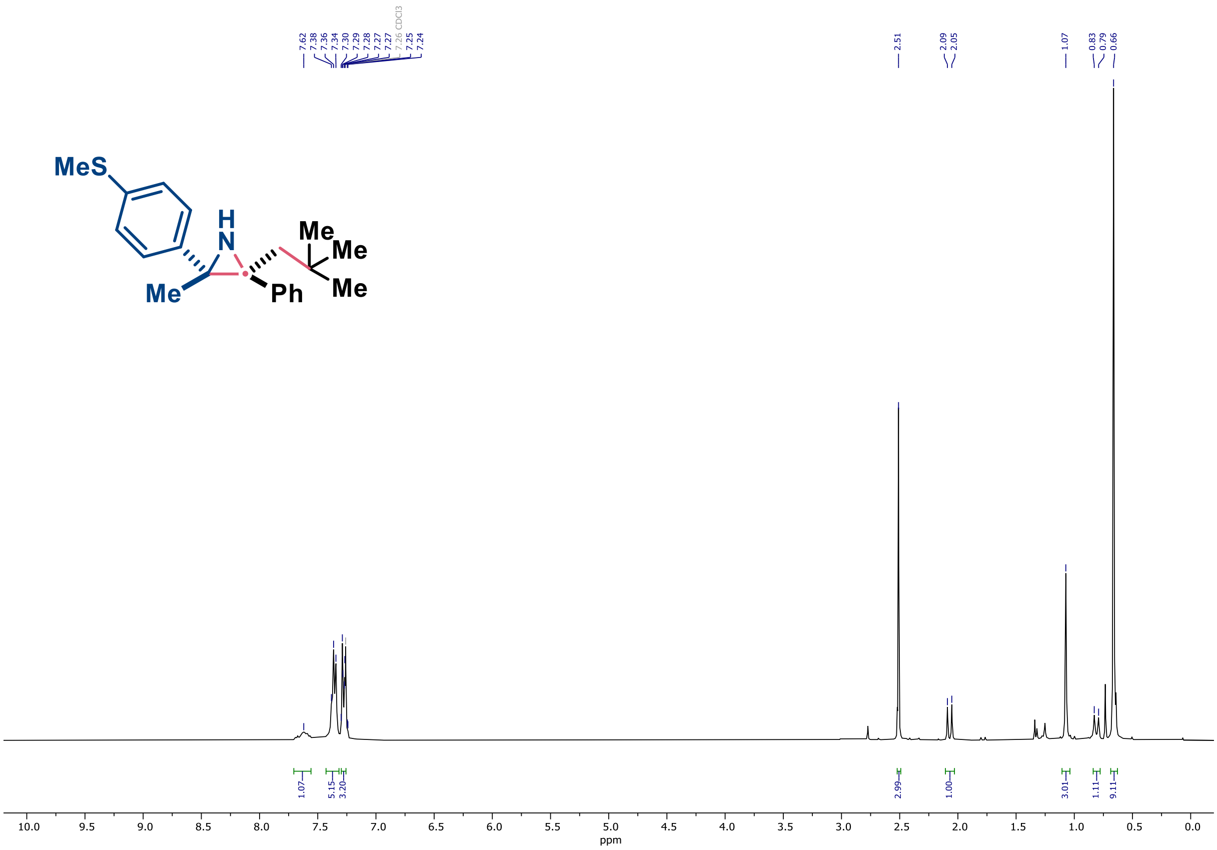

$^{13}C\{^1H\}$ NMR ($CDCl_3$, 101 MHz) for **3w (minor diastereomer)**

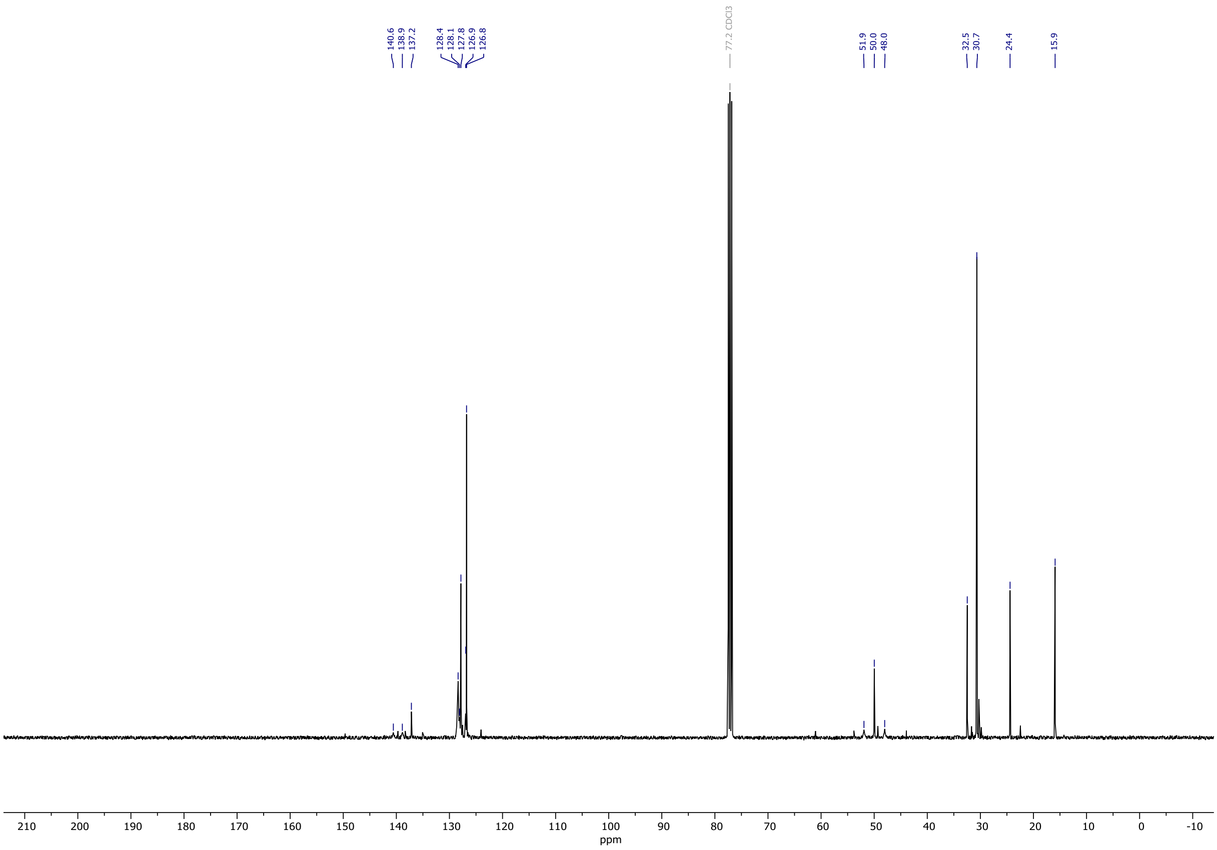

^{1}H NMR ($CDCl_3$, 400 MHz) for **3x (major diastereomer)**

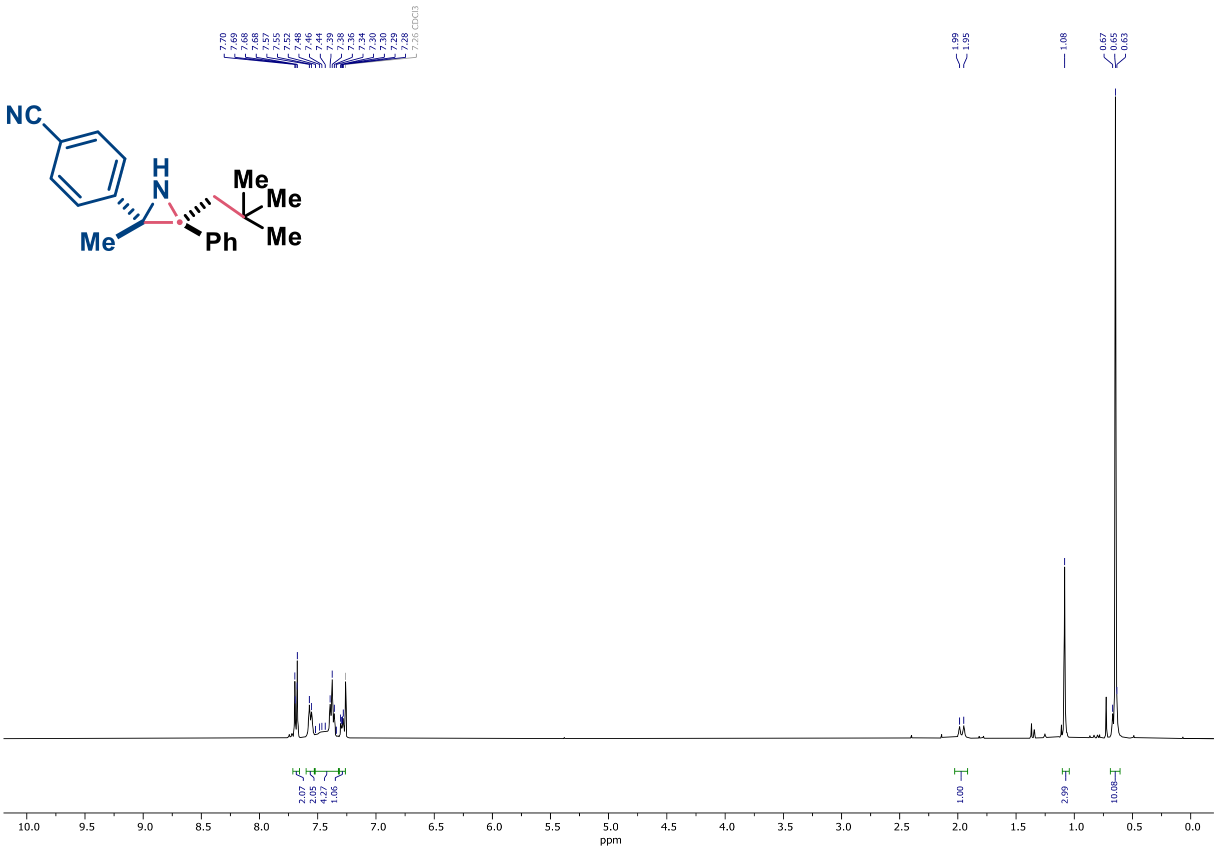

^{13}C{^{1}H} NMR ($CDCl_3$, 101 MHz) for **3x (major diastereomer)**

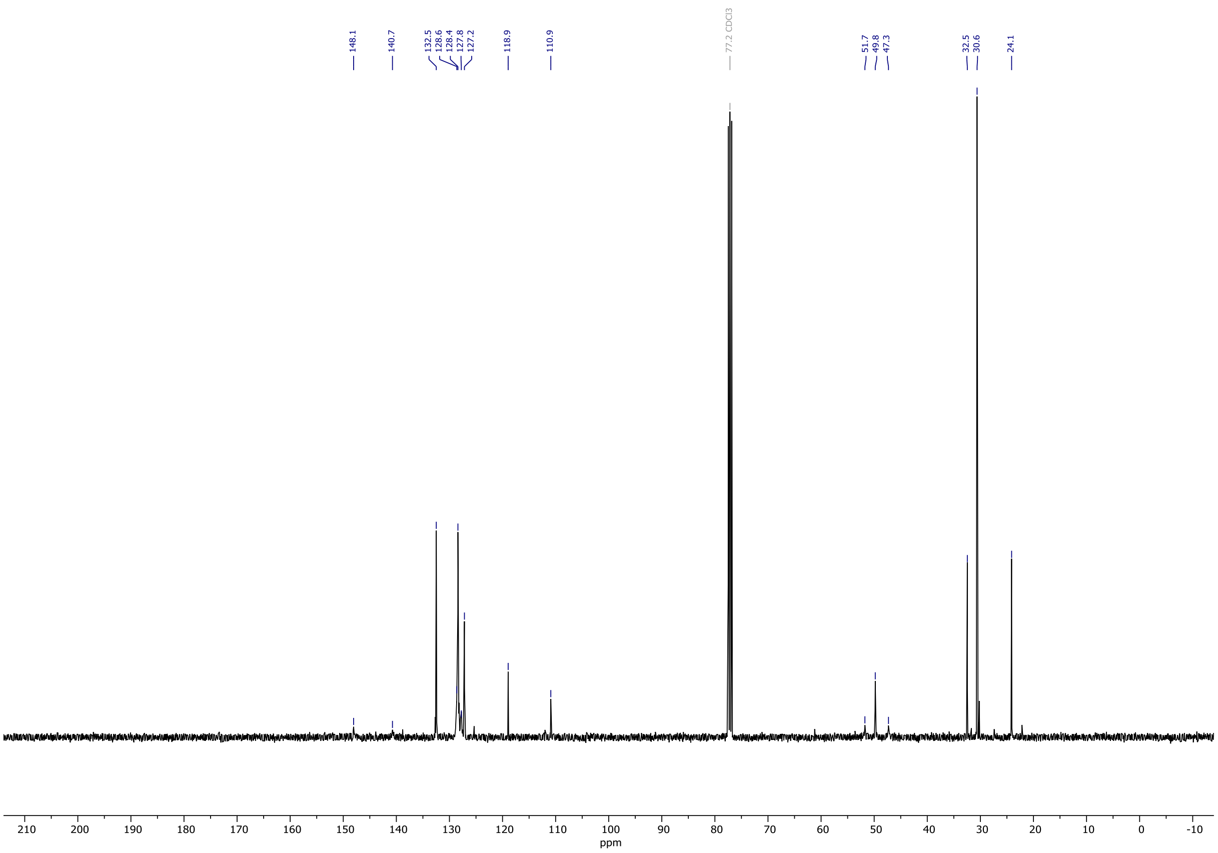

^{1}H NMR ($CDCl_3$, 400 MHz) for **3x (minor diastereomer)**

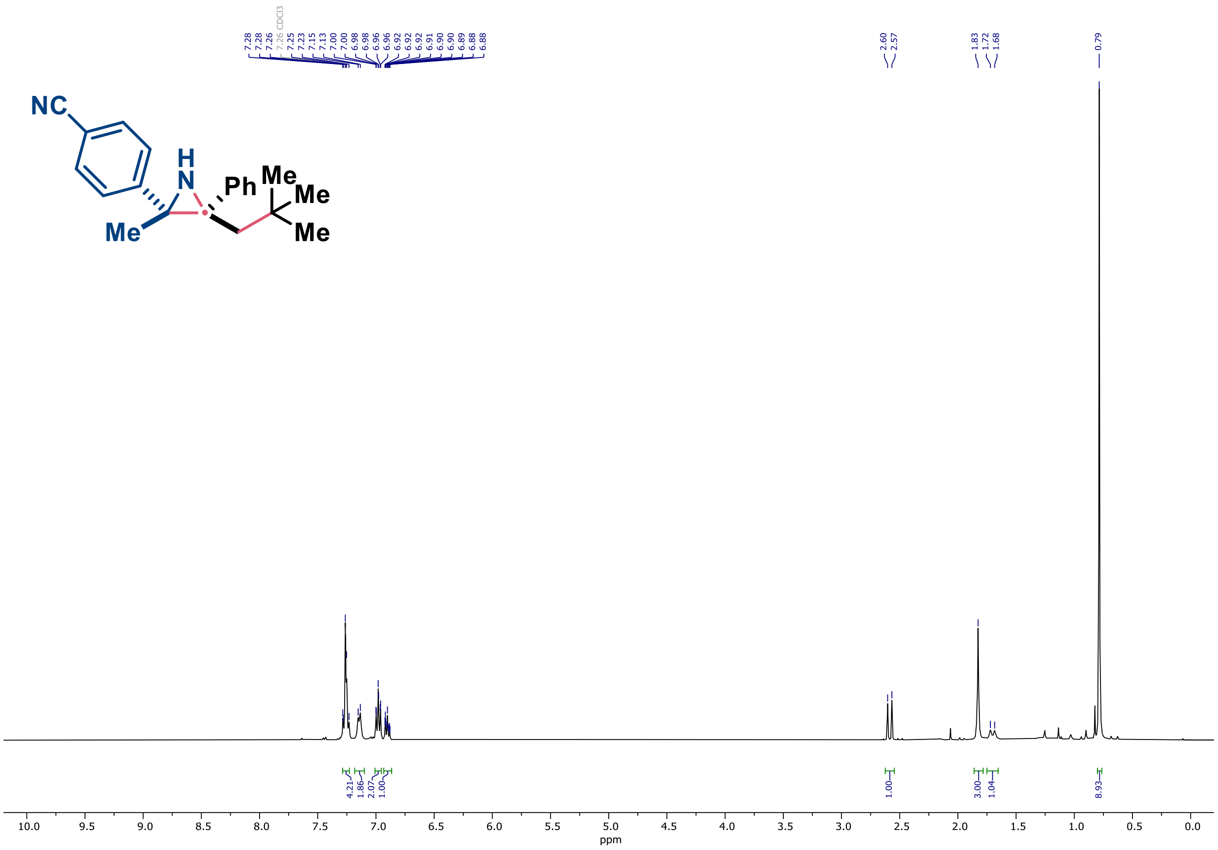

^{13}C{^{1}H} NMR ($CDCl_3$, 101 MHz) for **3x (minor diastereomer)**

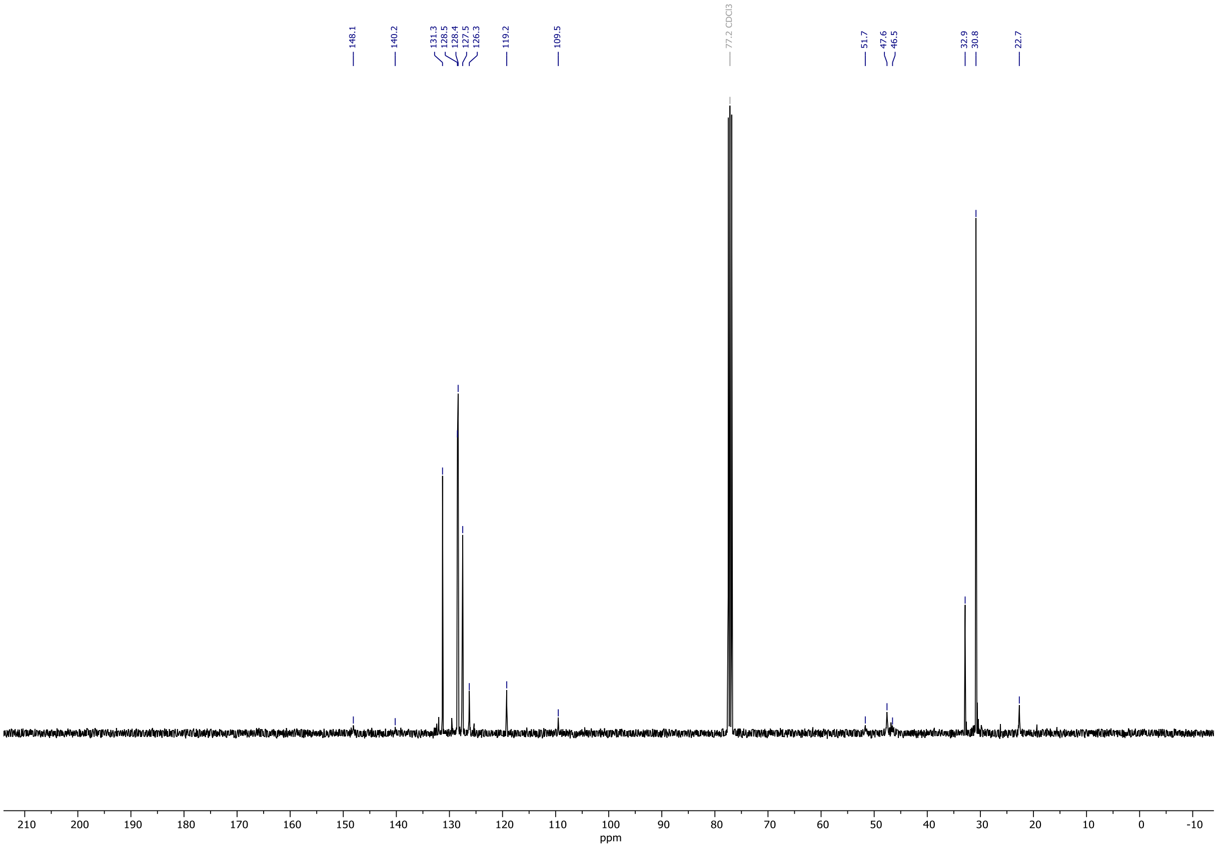

^{1}H NMR ($CDCl_3$, 400 MHz) for **3y (major diastereomer)**

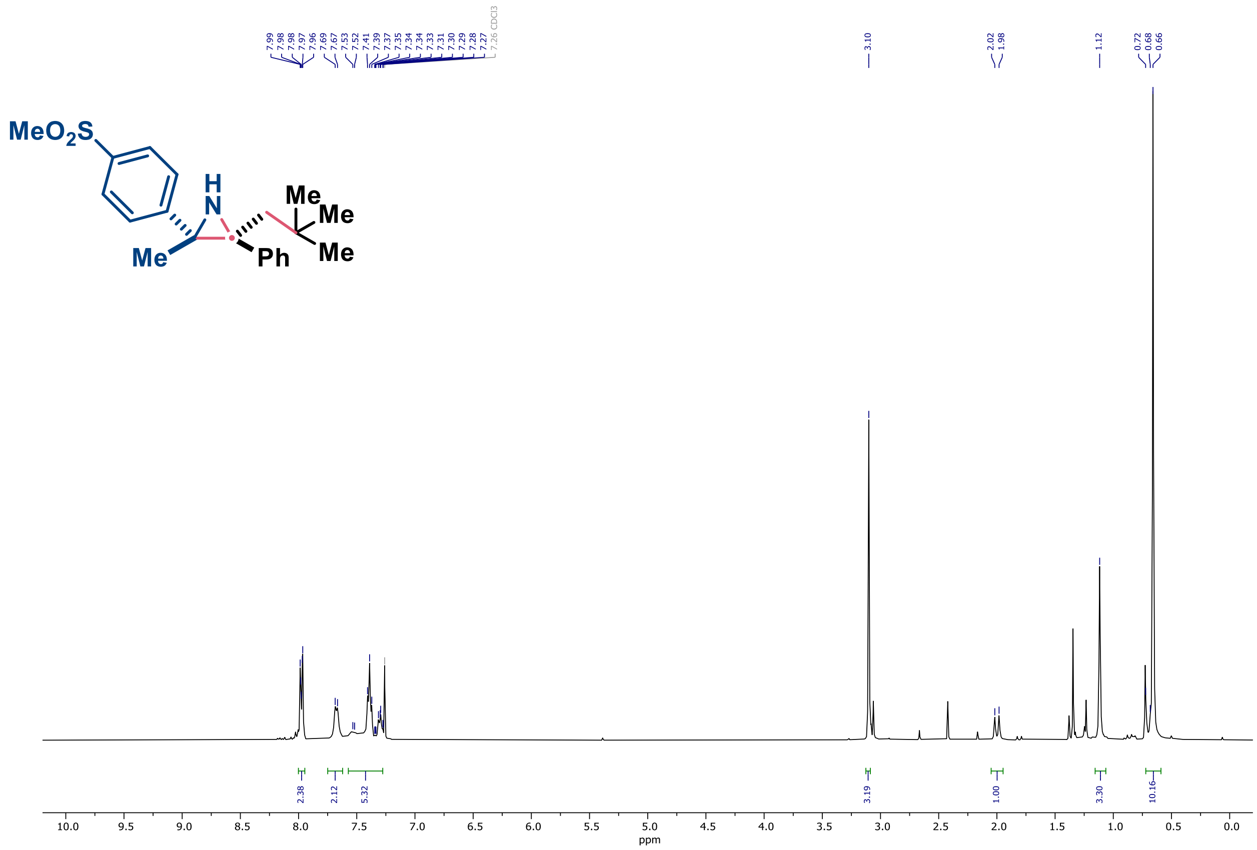

^{13}C{^{1}H} NMR ($CDCl_3$, 101 MHz) for **3y (major diastereomer)**

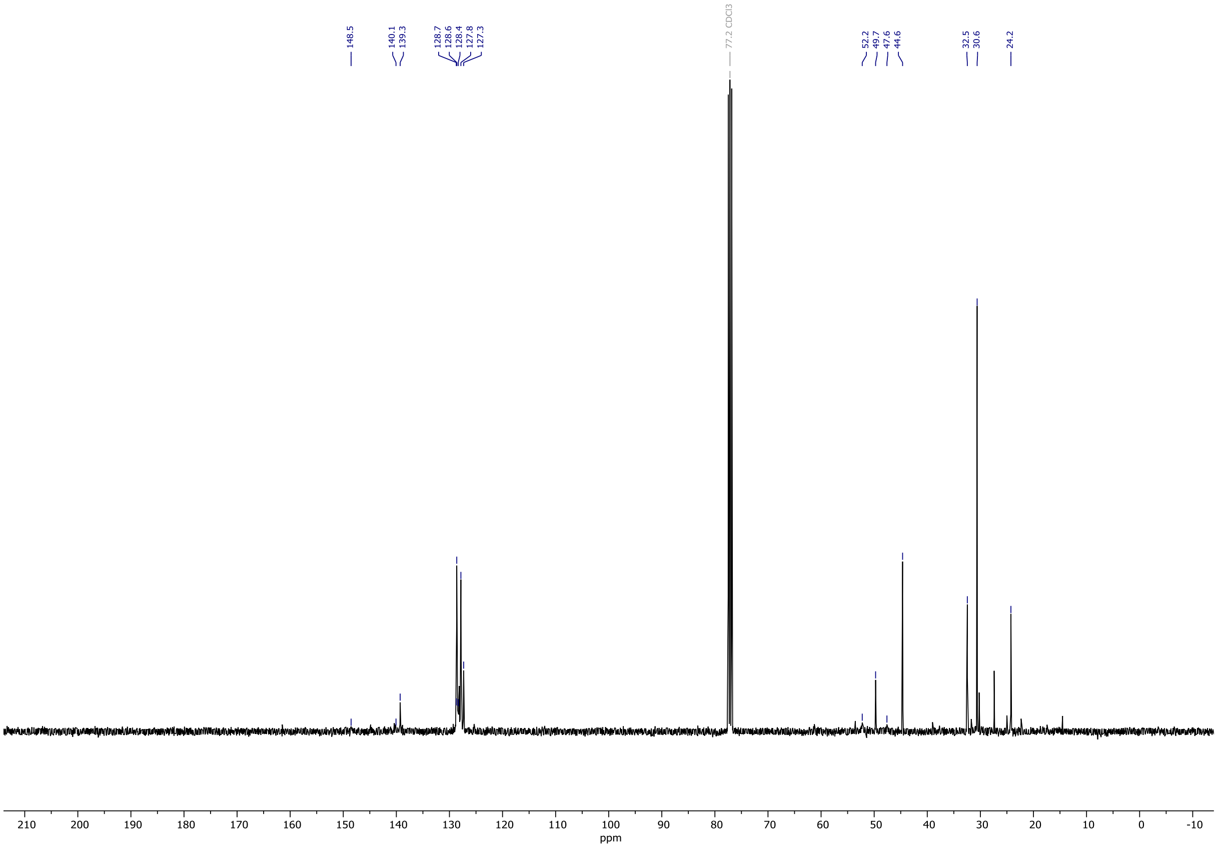

^{1}H NMR ($CDCl_3$, 400 MHz) for **3y (minor diastereomer)**

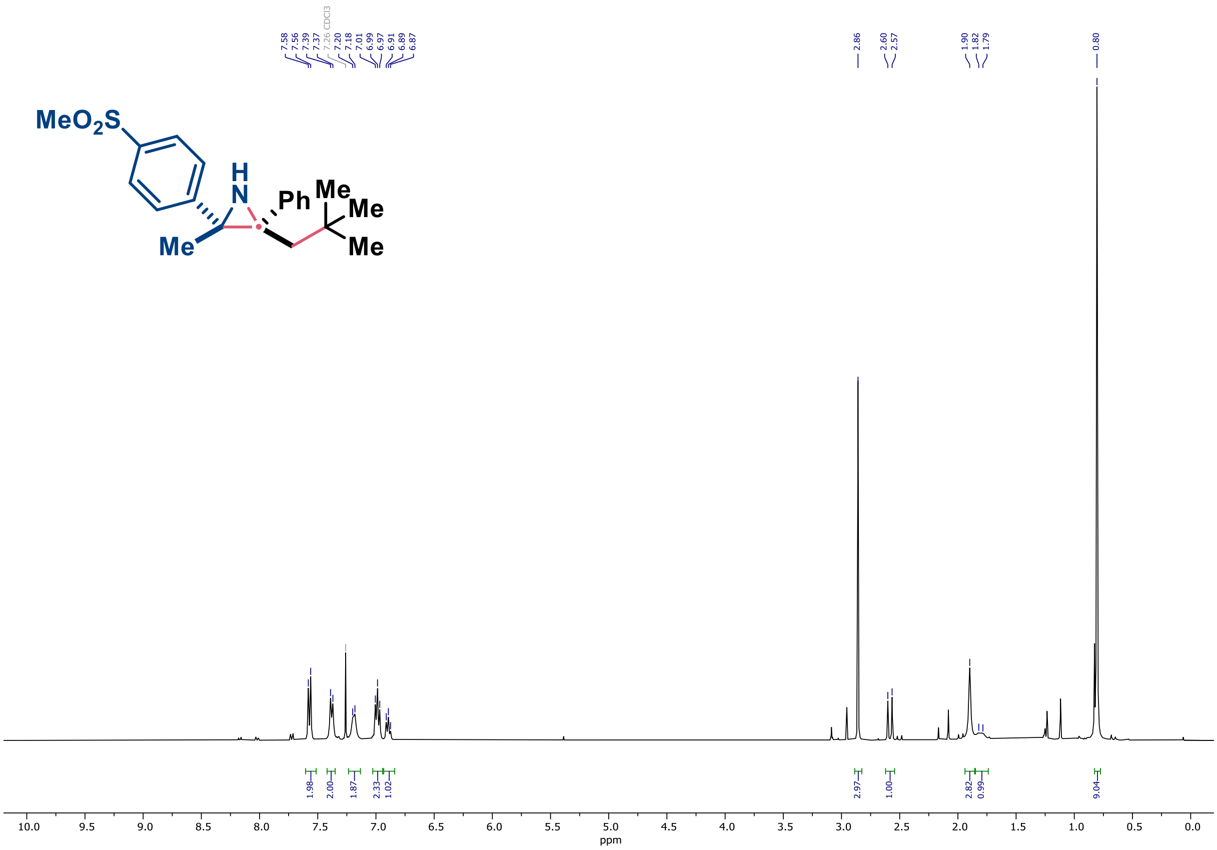

^{13}C{^{1}H} NMR ($CDCl_3$, 101 MHz) for **3y (minor diastereomer)**

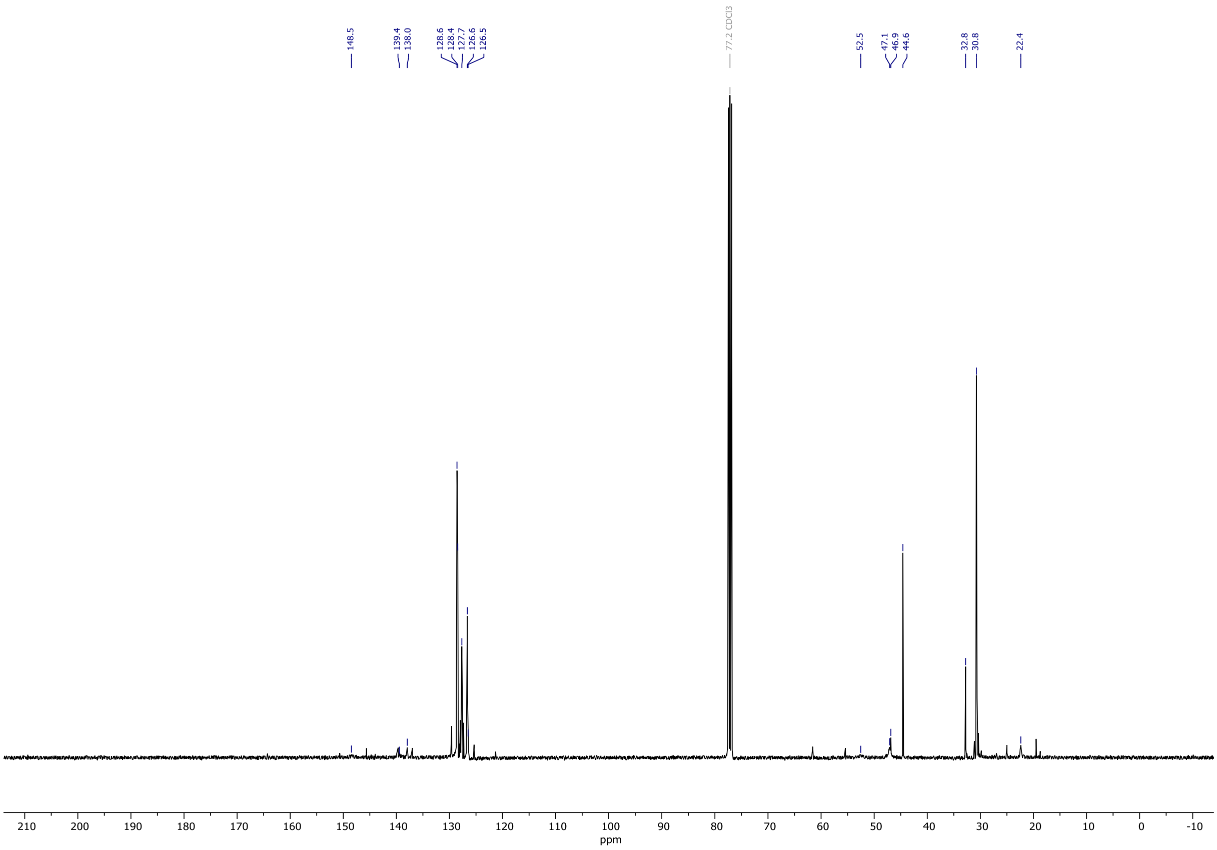

^{1}H NMR ($CDCl_3$, 500 MHz) for **3z**

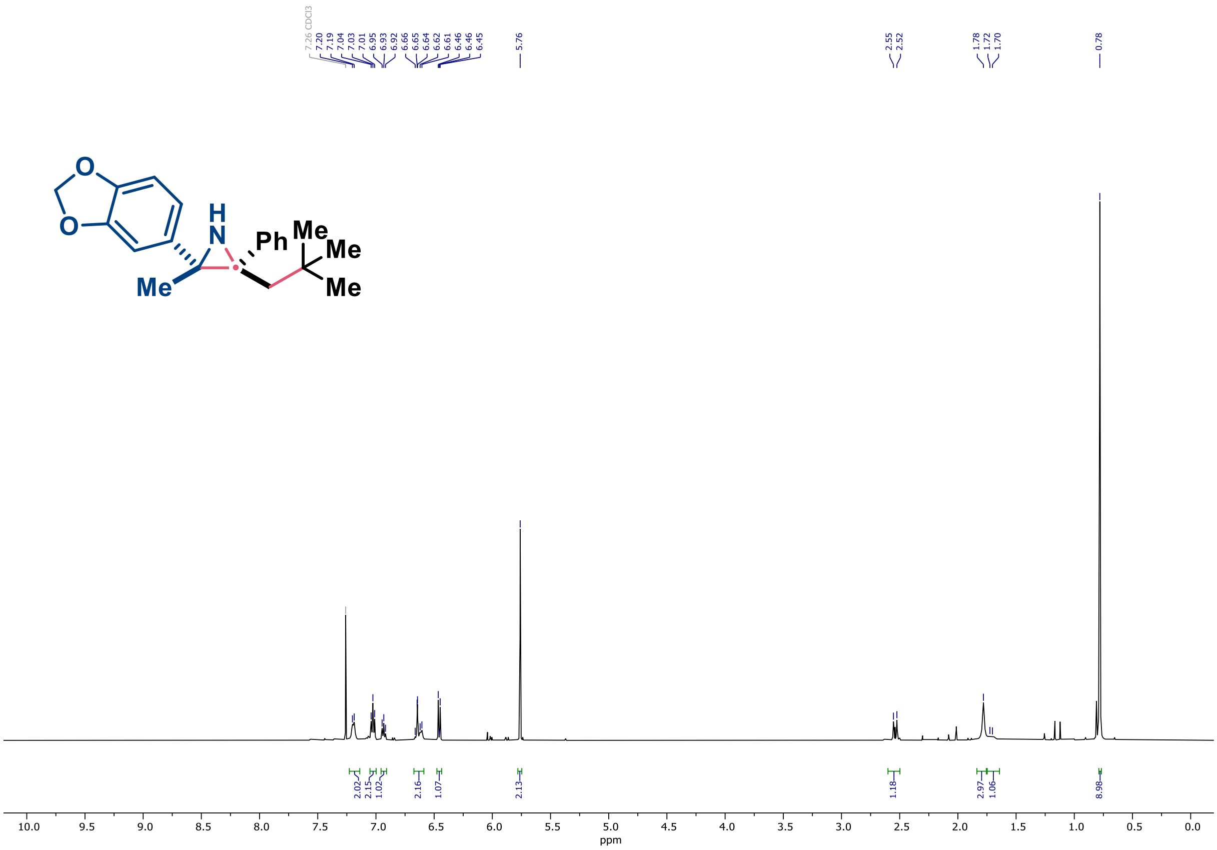

^{13}C{^{1}H} NMR ($CDCl_3$, 126 MHz) for **3z**

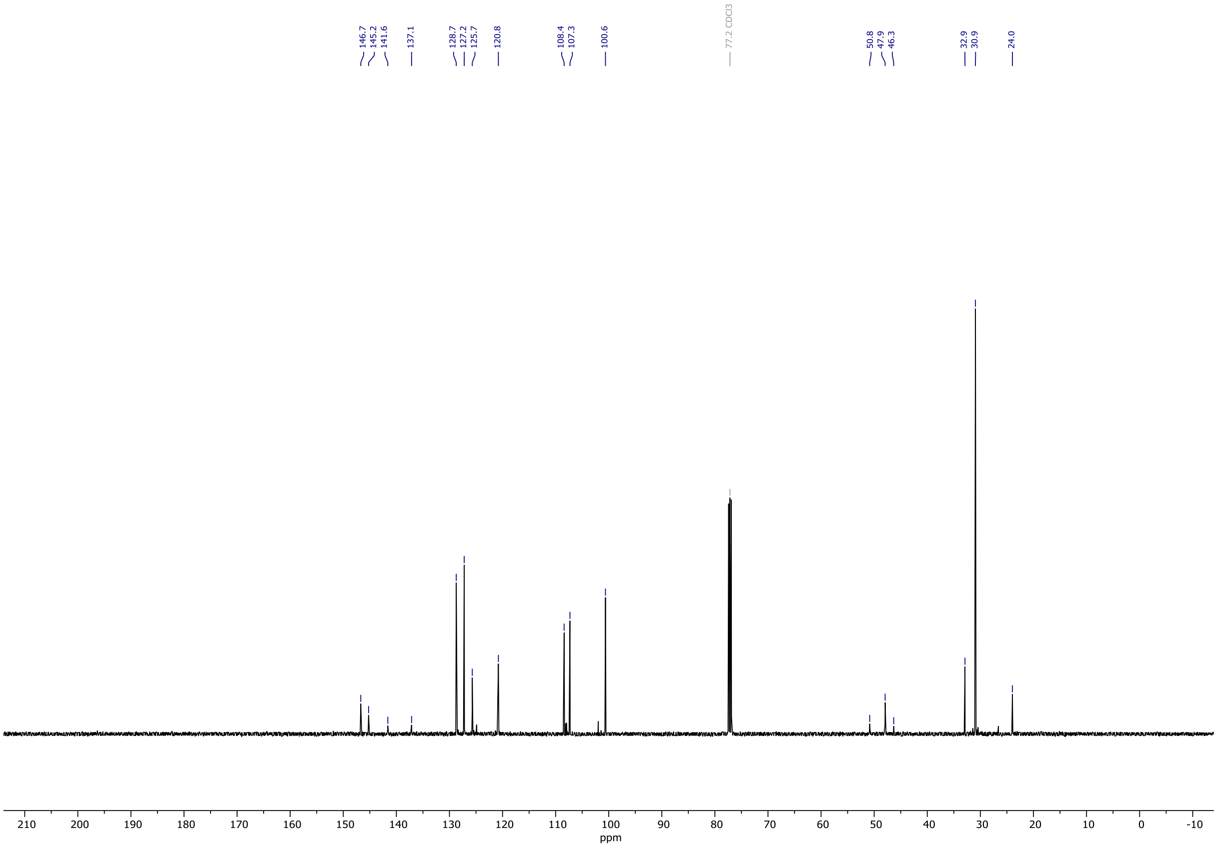

^{1}H NMR ($CDCl_3$, 400 MHz) for **3aa**

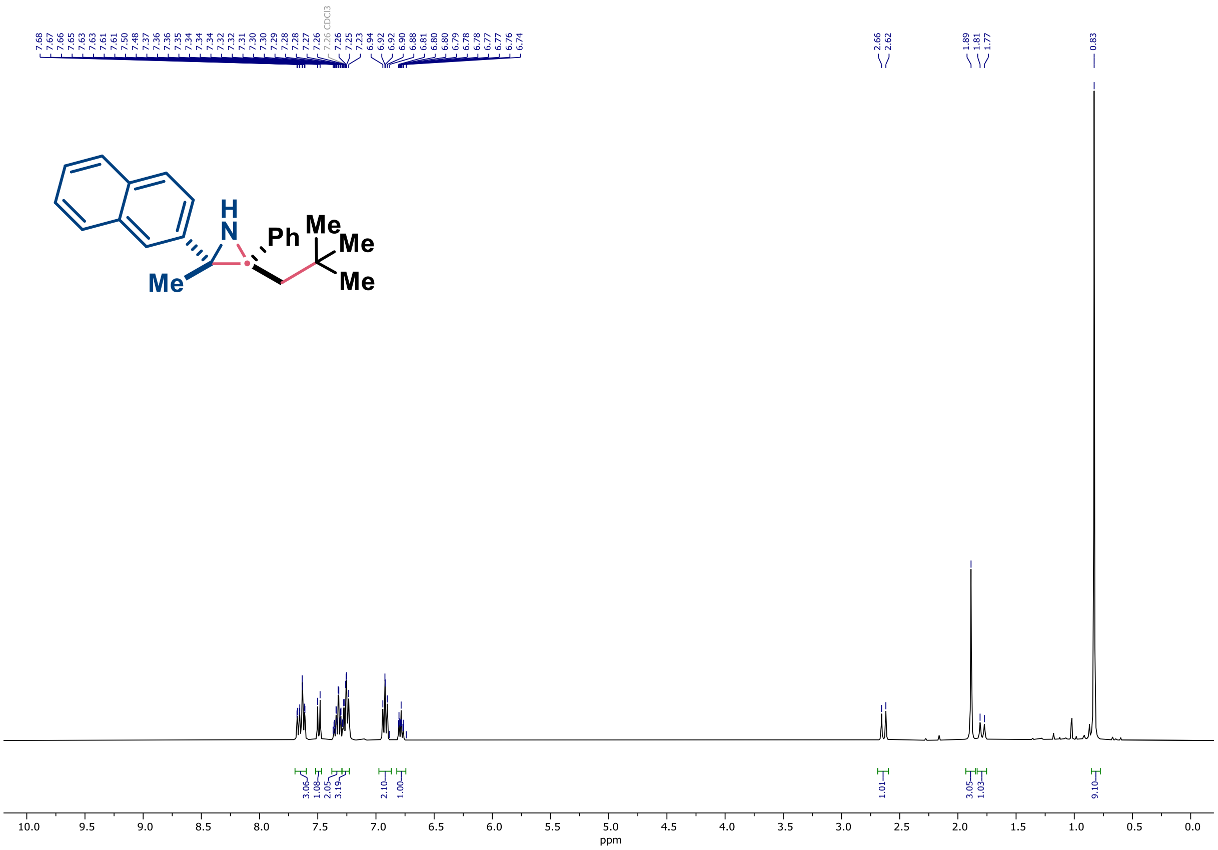

^{13}C{^{1}H} NMR ($CDCl_3$, 101 MHz) for **3aa**

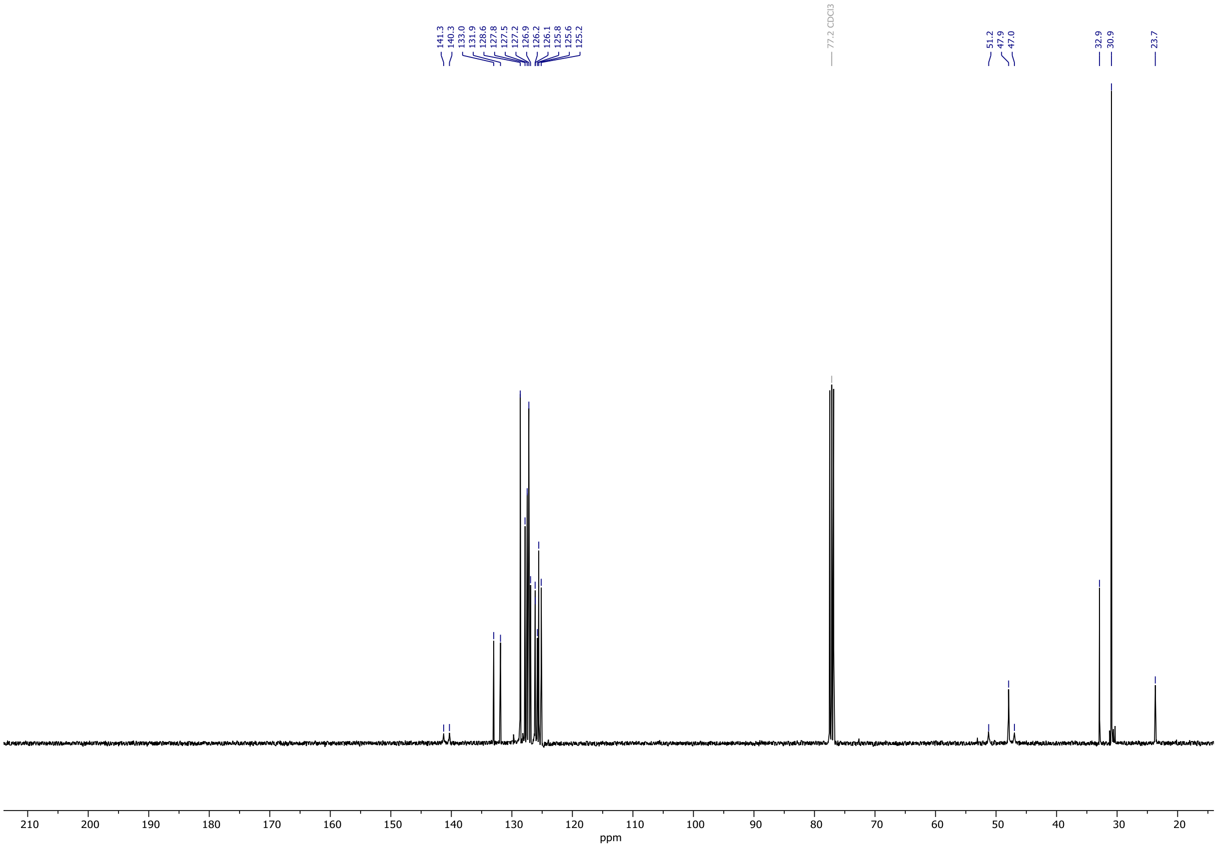

^{1}H NMR (CDCl$_3$, 400 MHz) for **3ab**

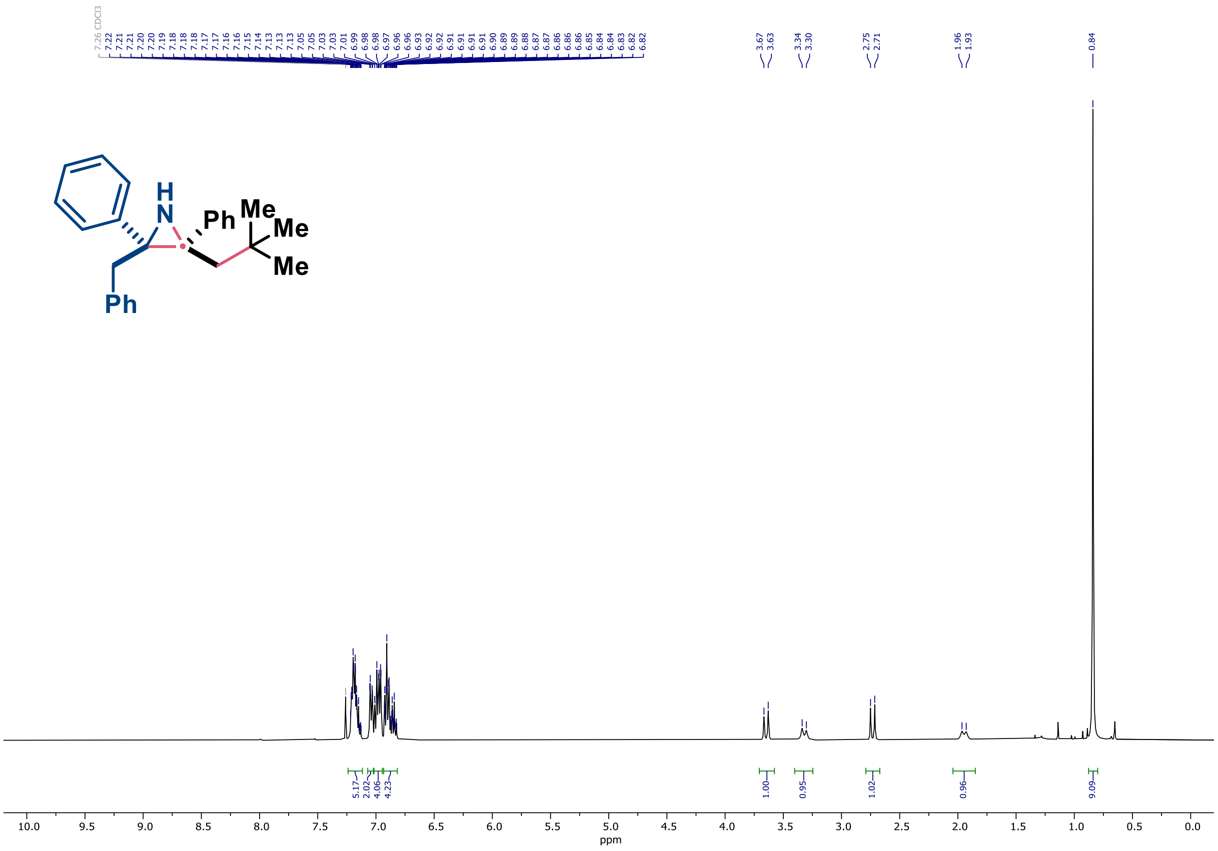

^{13}C{^{1}H} NMR (CDCl$_3$, 101 MHz) for **3ab**

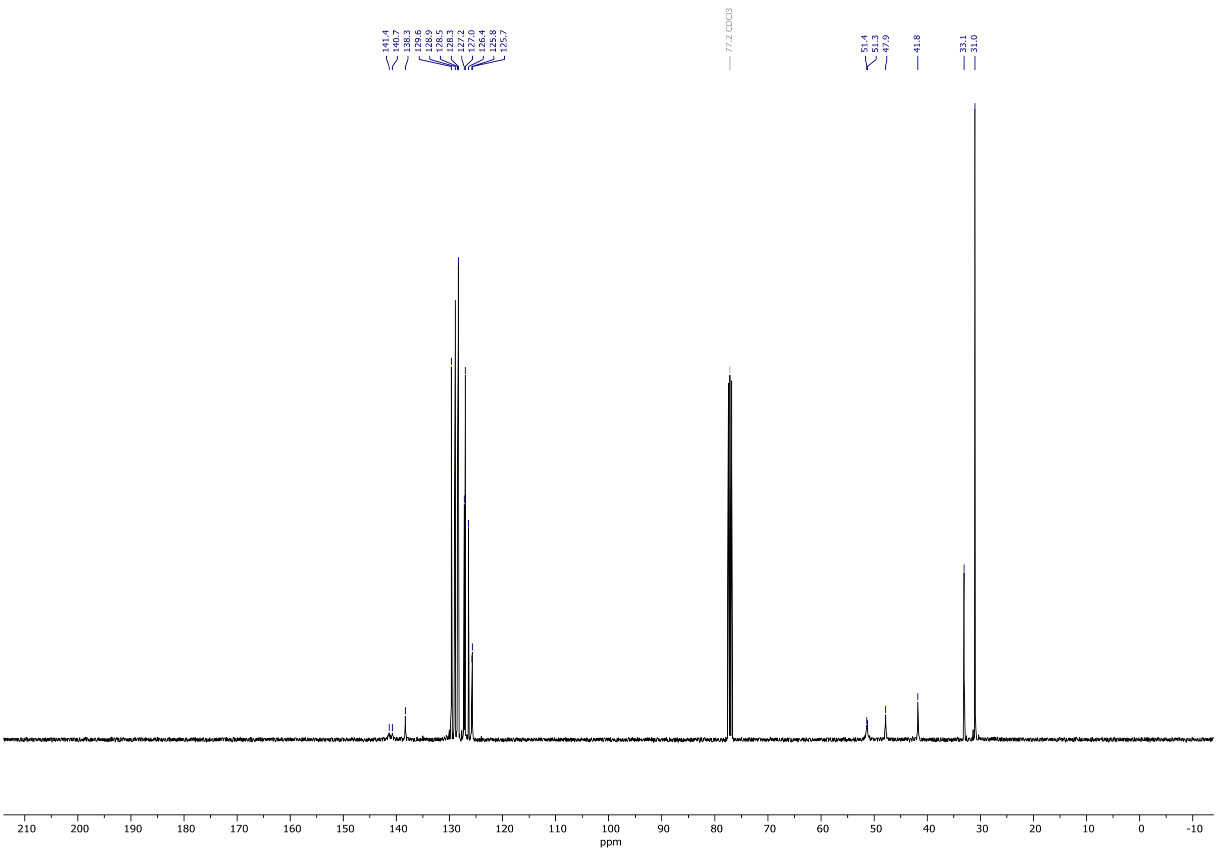

^{1}H NMR ($CDCl_3$, 400 MHz) for **3ac (major diastereomer)**

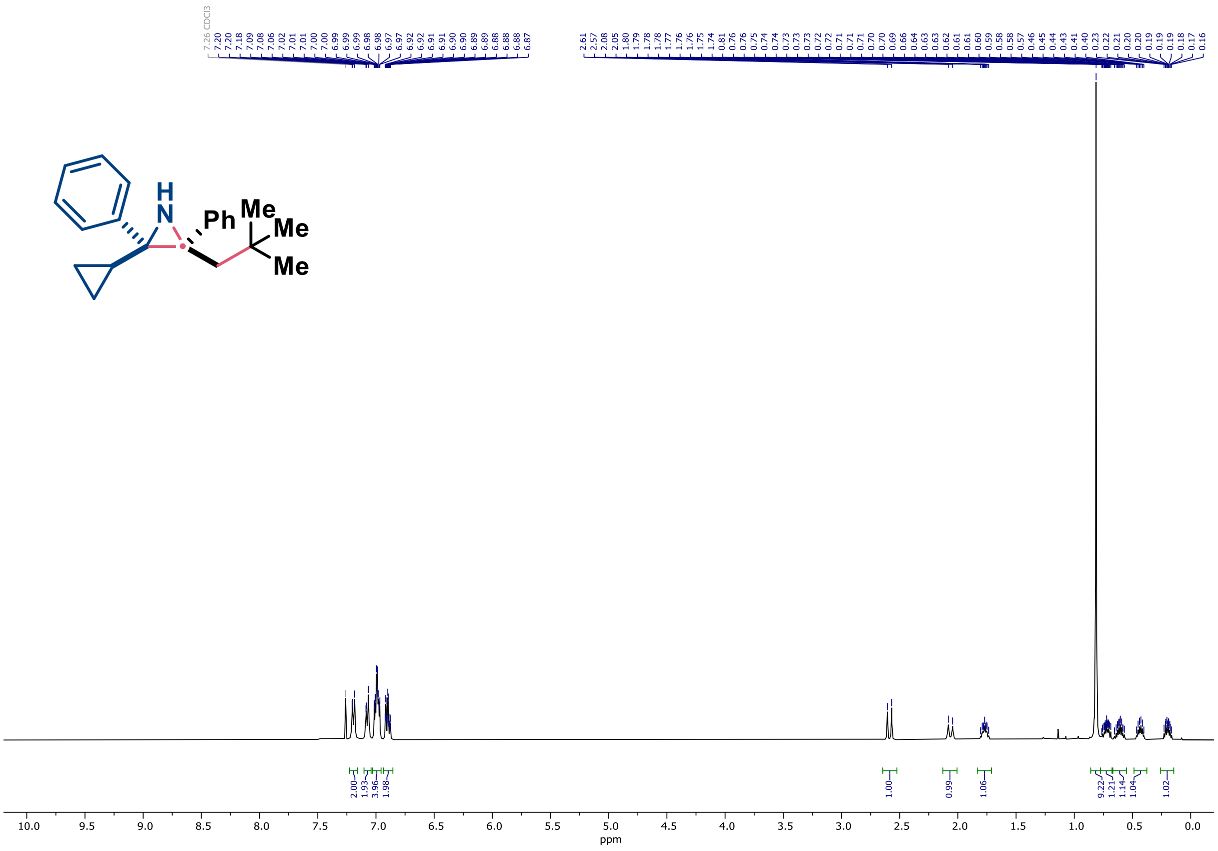

^{13}C{^{1}H} NMR ($CDCl_3$, 101 MHz) for **3ac (major diastereomer)**

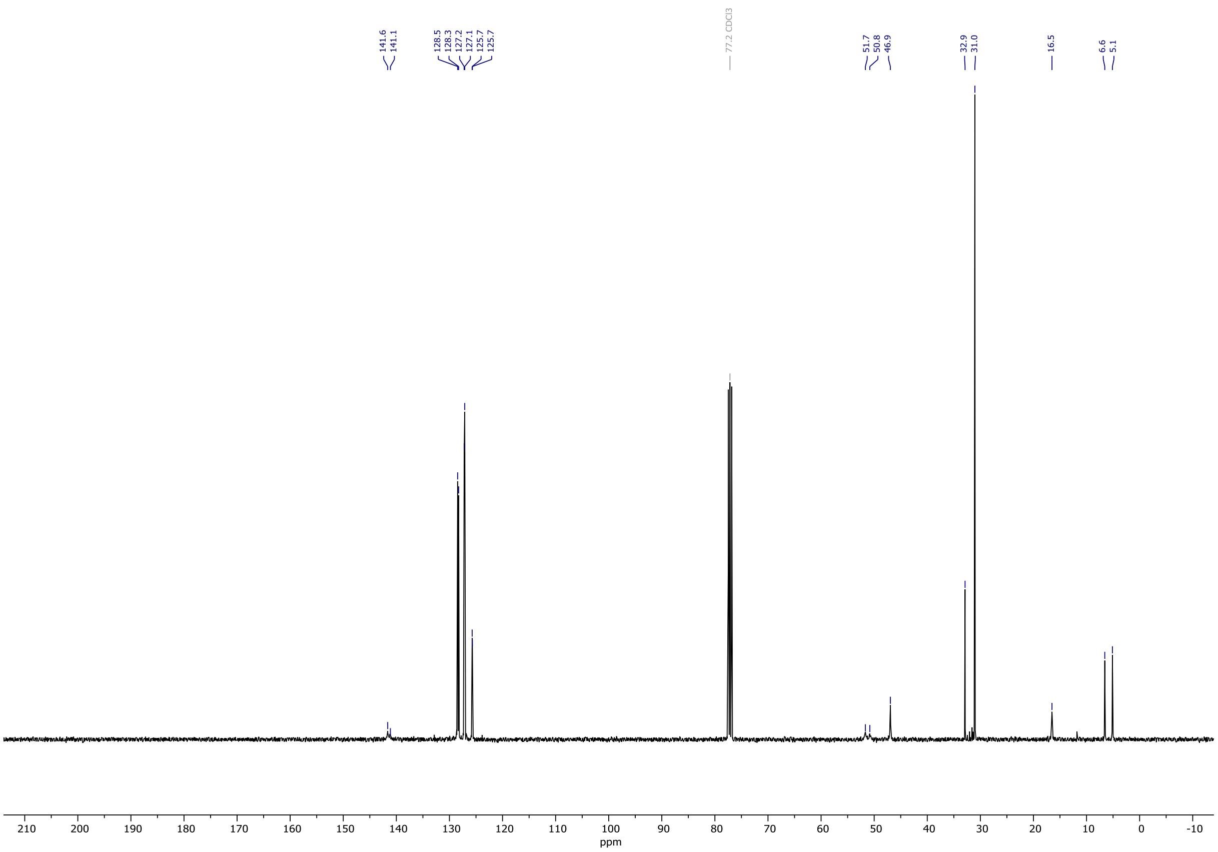

¹H NMR (CDCl$_3$, 400 MHz) for **3ac (minor diastereomer)**

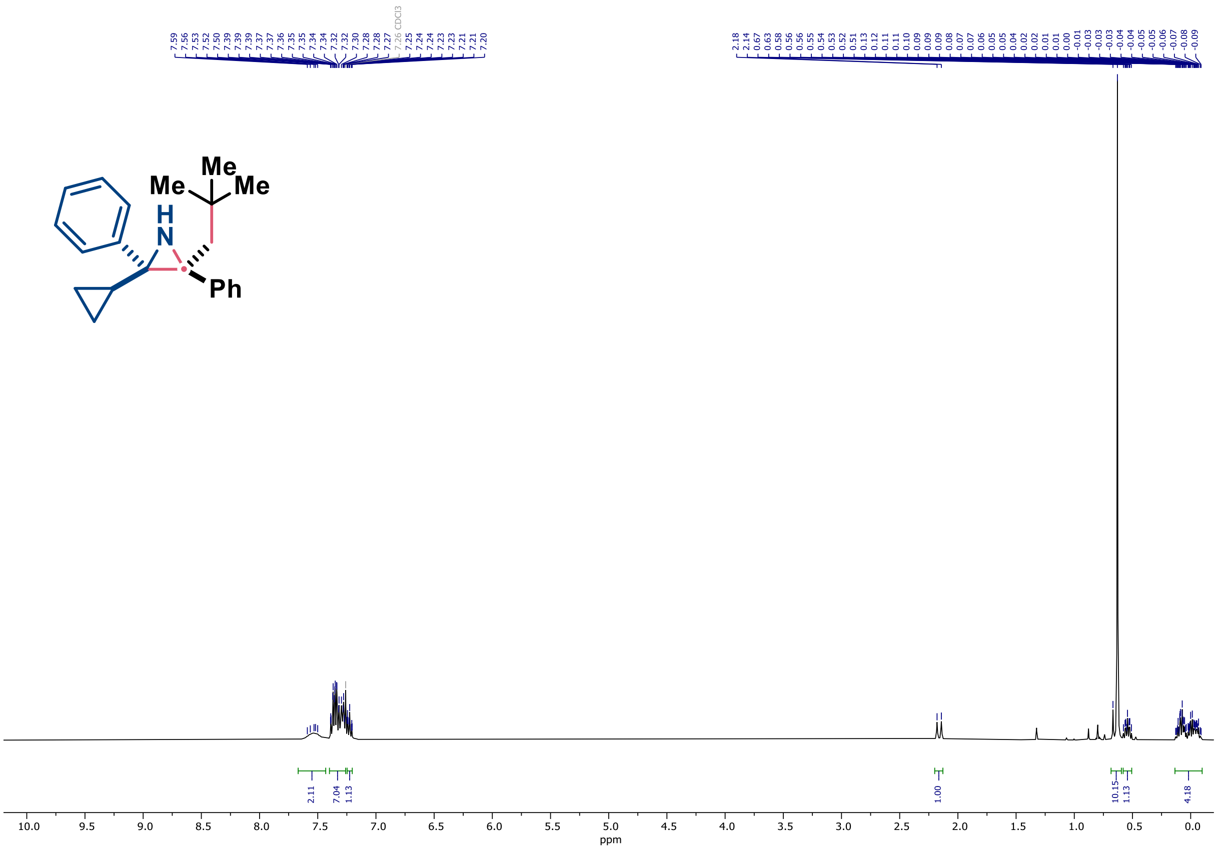

^{13}C{^{1}H} NMR (CDCl$_3$, 101 MHz) for **3ac (minor diastereomer)**

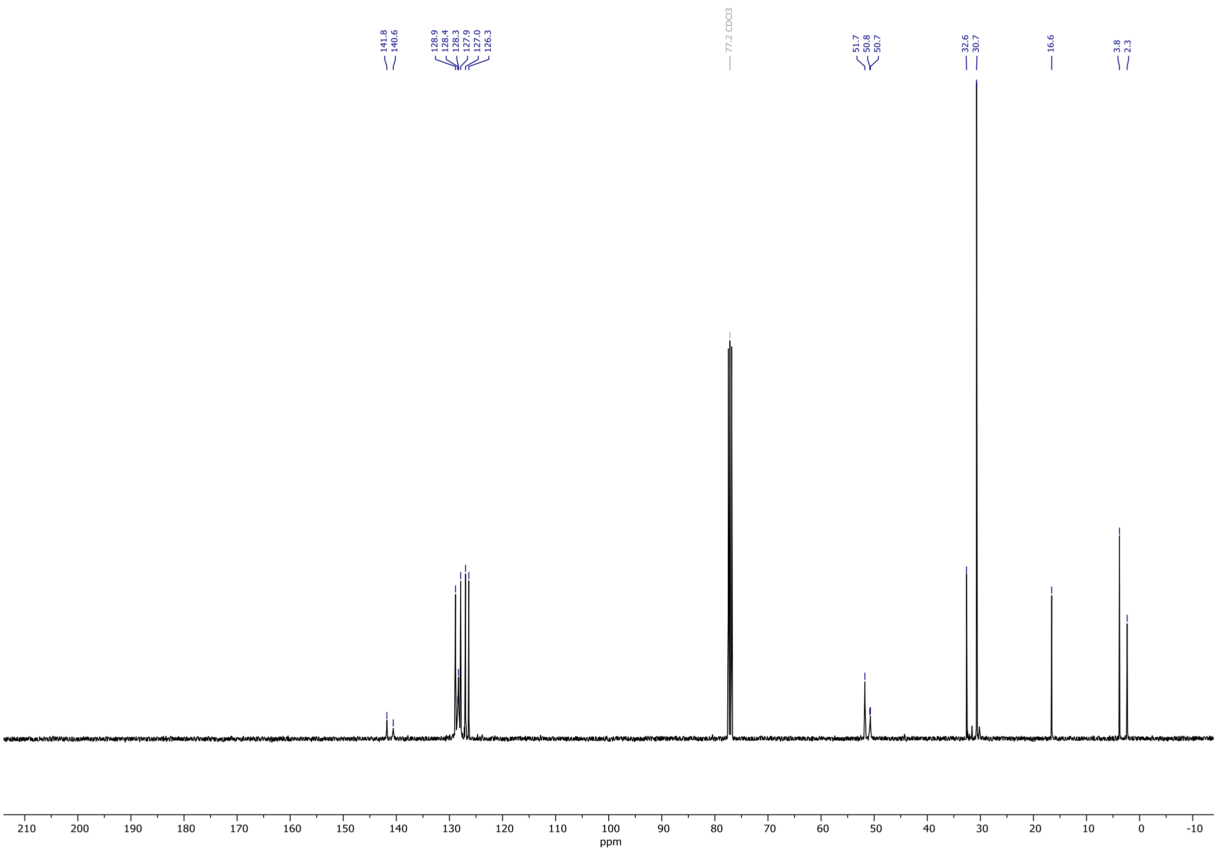

^{1}H NMR (CDCl$_3$, 400 MHz) for **3ad**

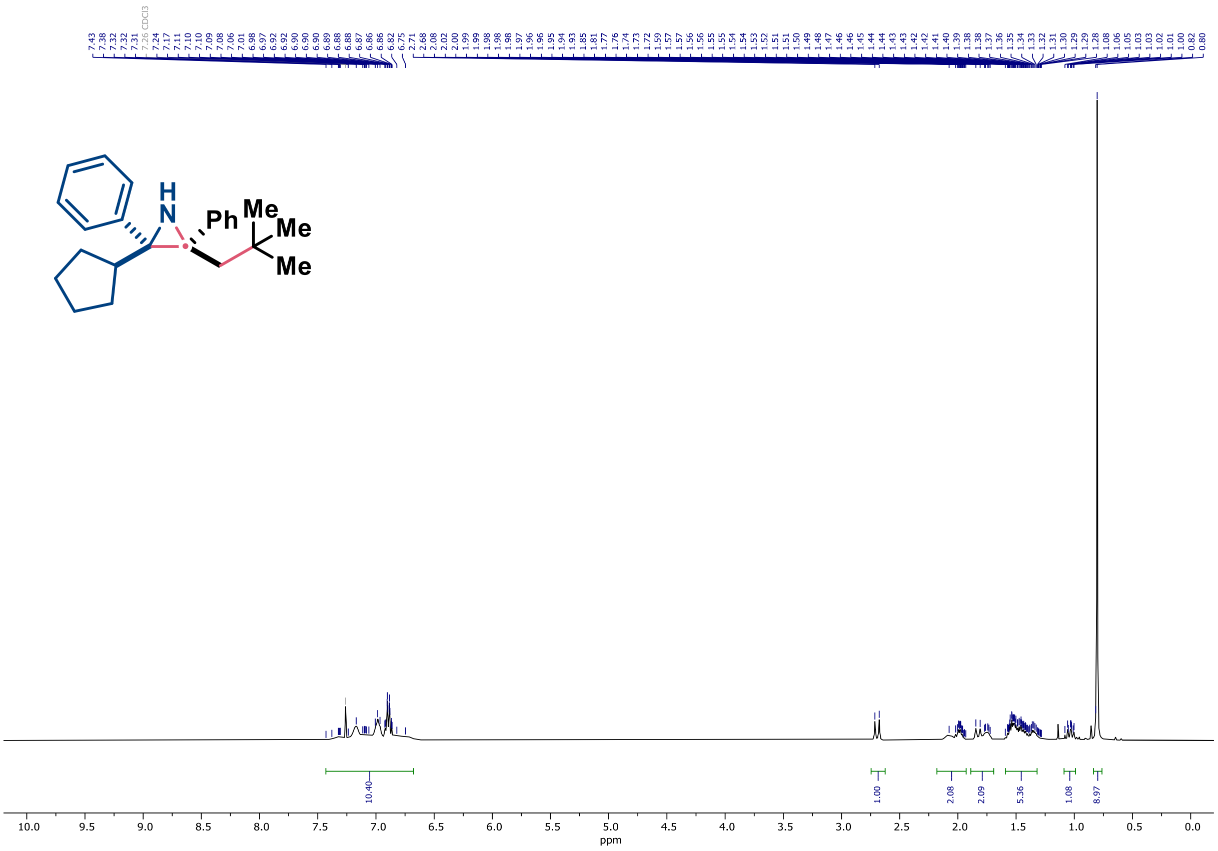

^{13}C{^{1}H} NMR (CDCl$_3$, 101 MHz) for **3ad**

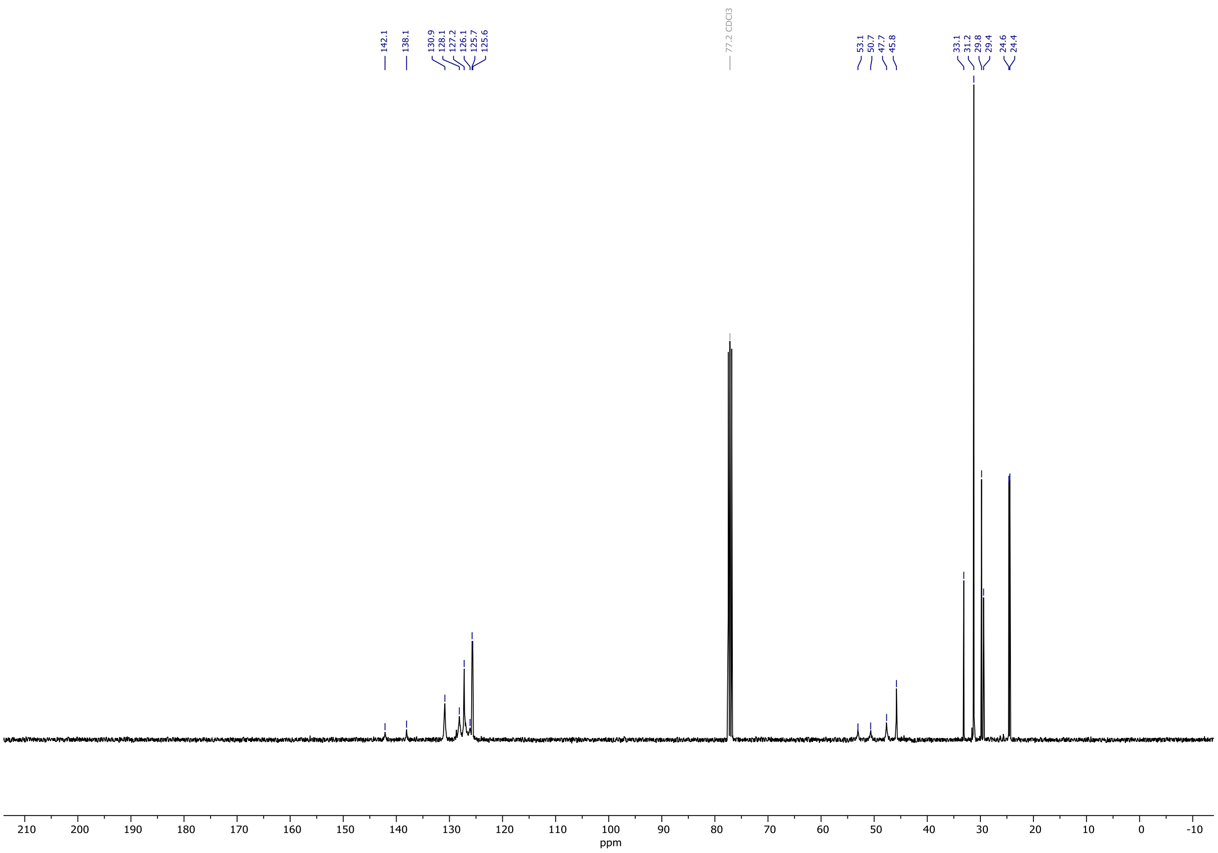

^{1}H NMR (CDCl$_3$, 400 MHz) for **3ae**

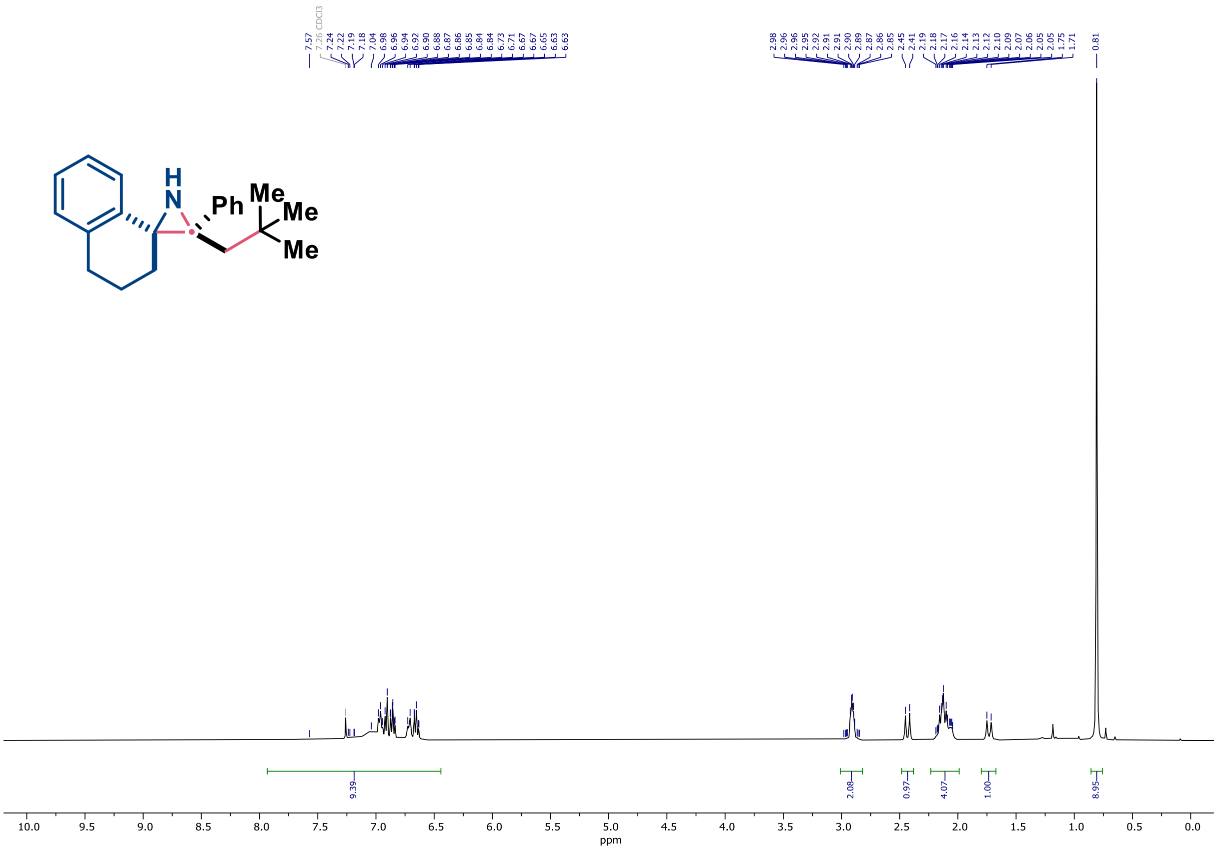

^{13}C{^{1}H} NMR (CDCl$_3$, 101 MHz) for **3ae**

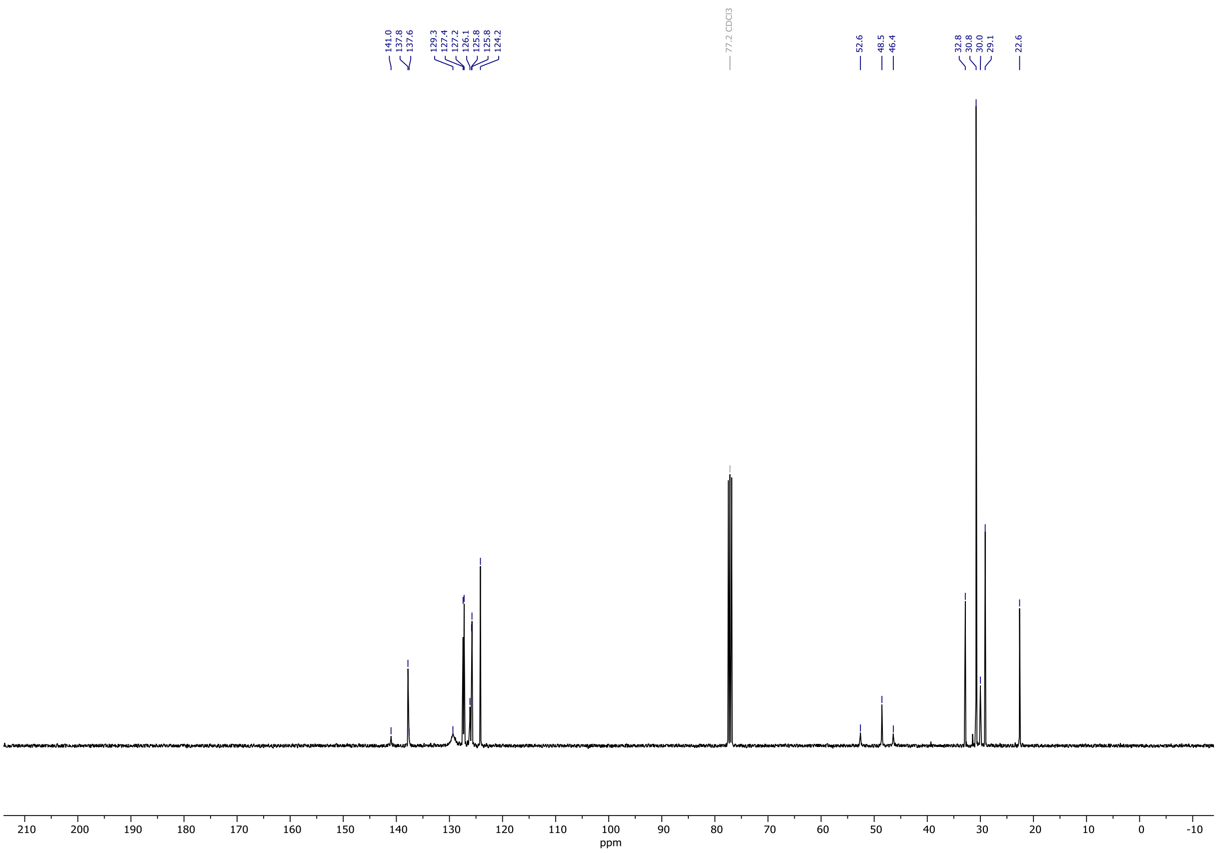

^{1}H NMR ($CDCl_3$, 400 MHz) for **3af**

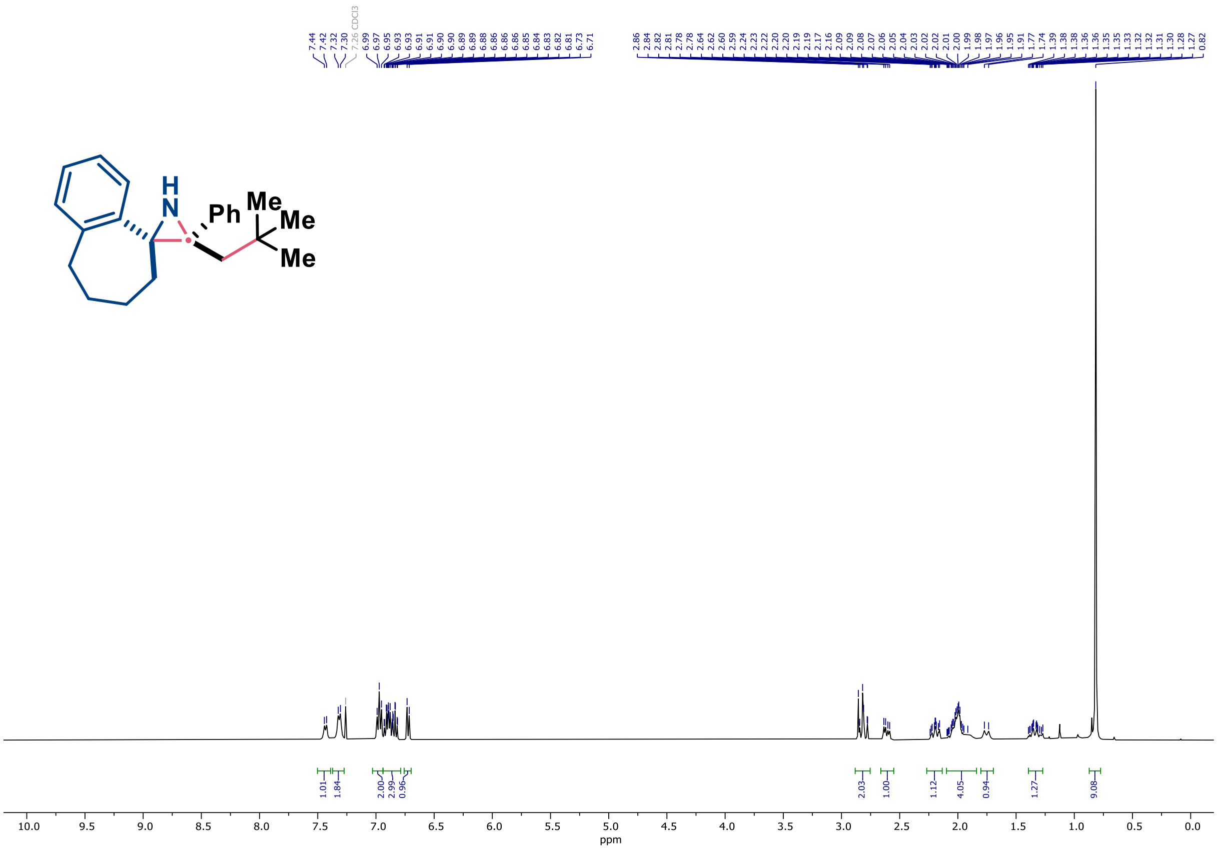

^{13}C{^{1}H} NMR ($CDCl_3$, 101 MHz) for **3af**

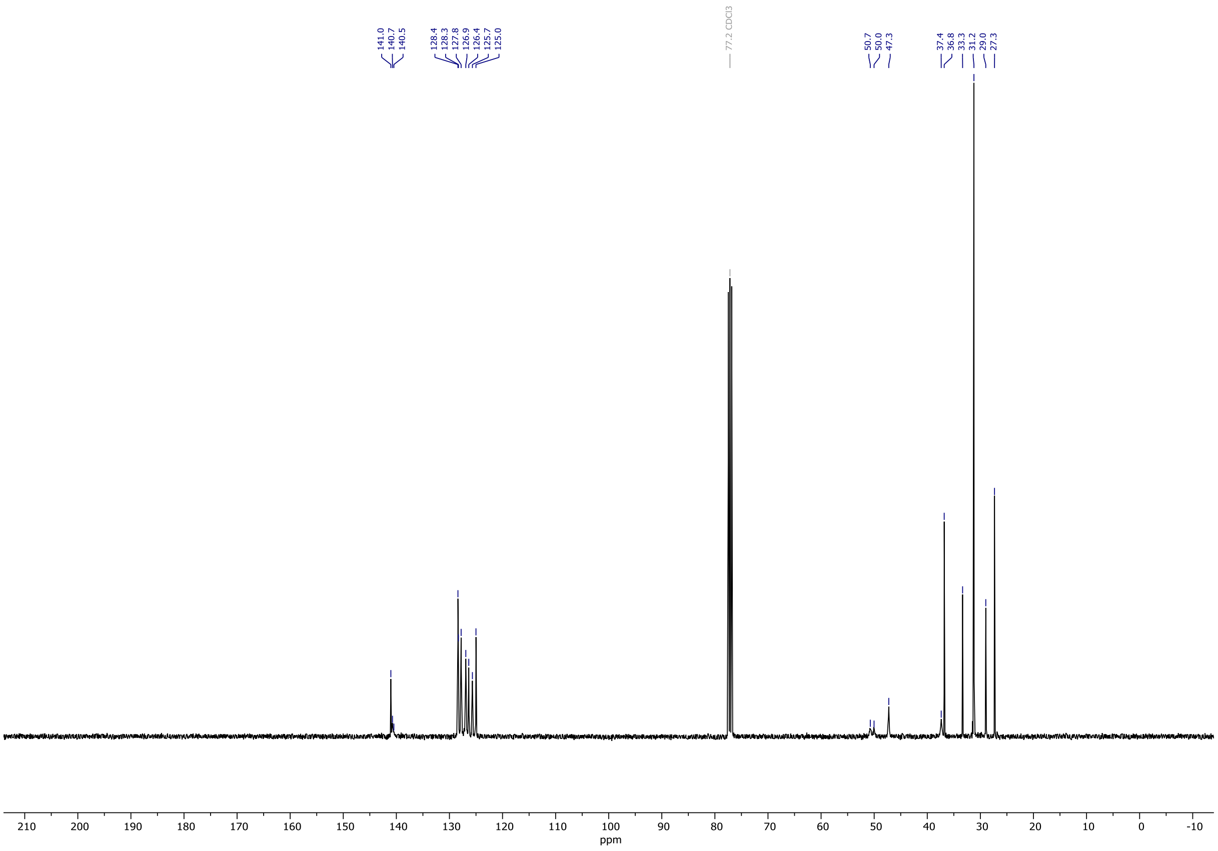

^{1}H NMR (CDCl$_3$, 599 MHz) for **3ag**

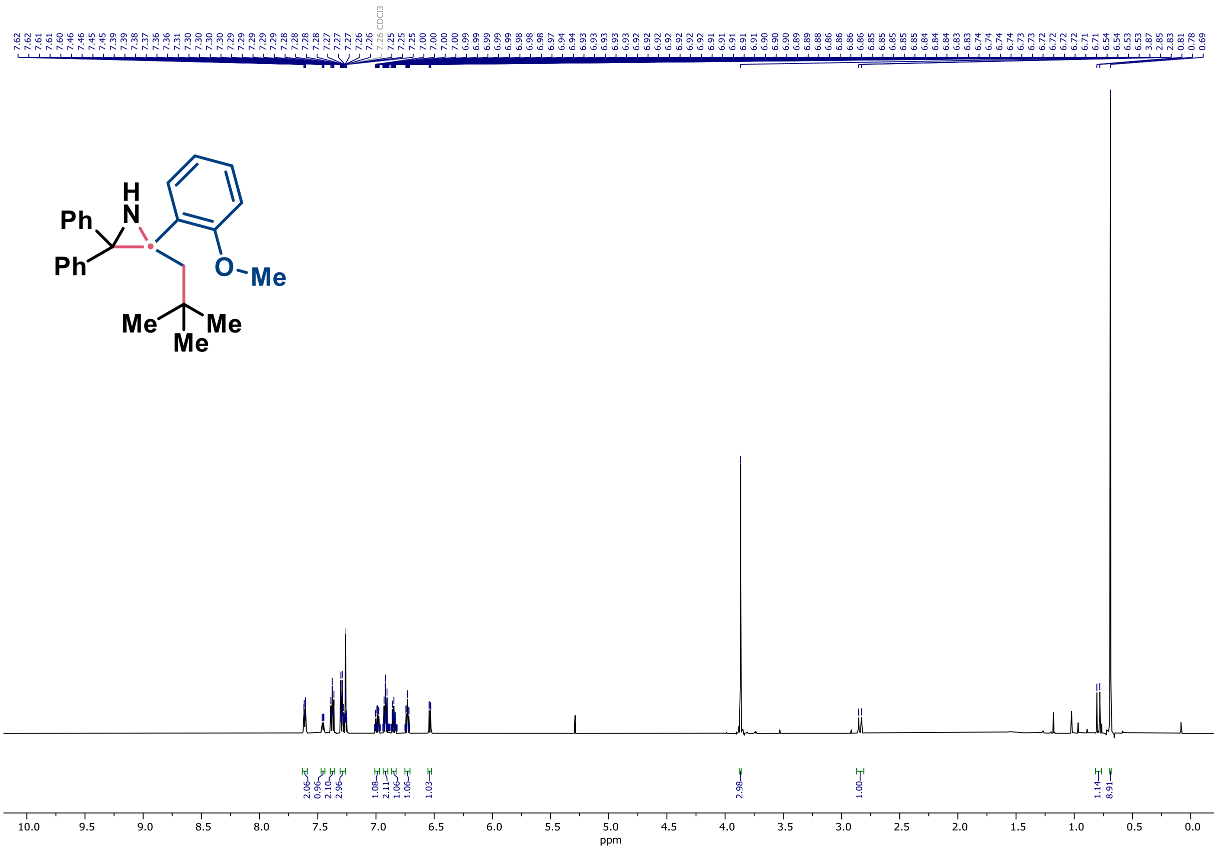

^{13}C{^{1}H} NMR (CDCl$_3$, 151 MHz) for **3ag**

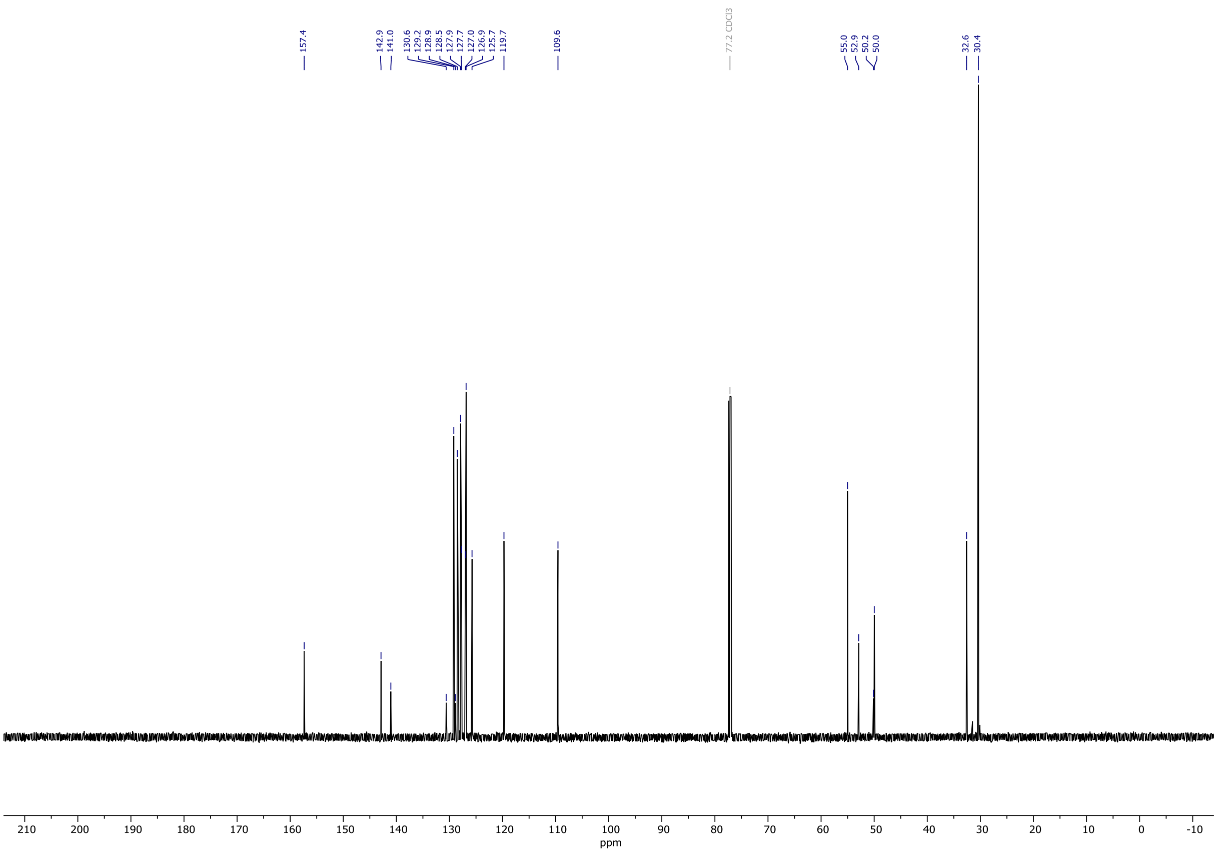

^{1}H NMR ($CDCl_3$, 599 MHz) for **3ah**

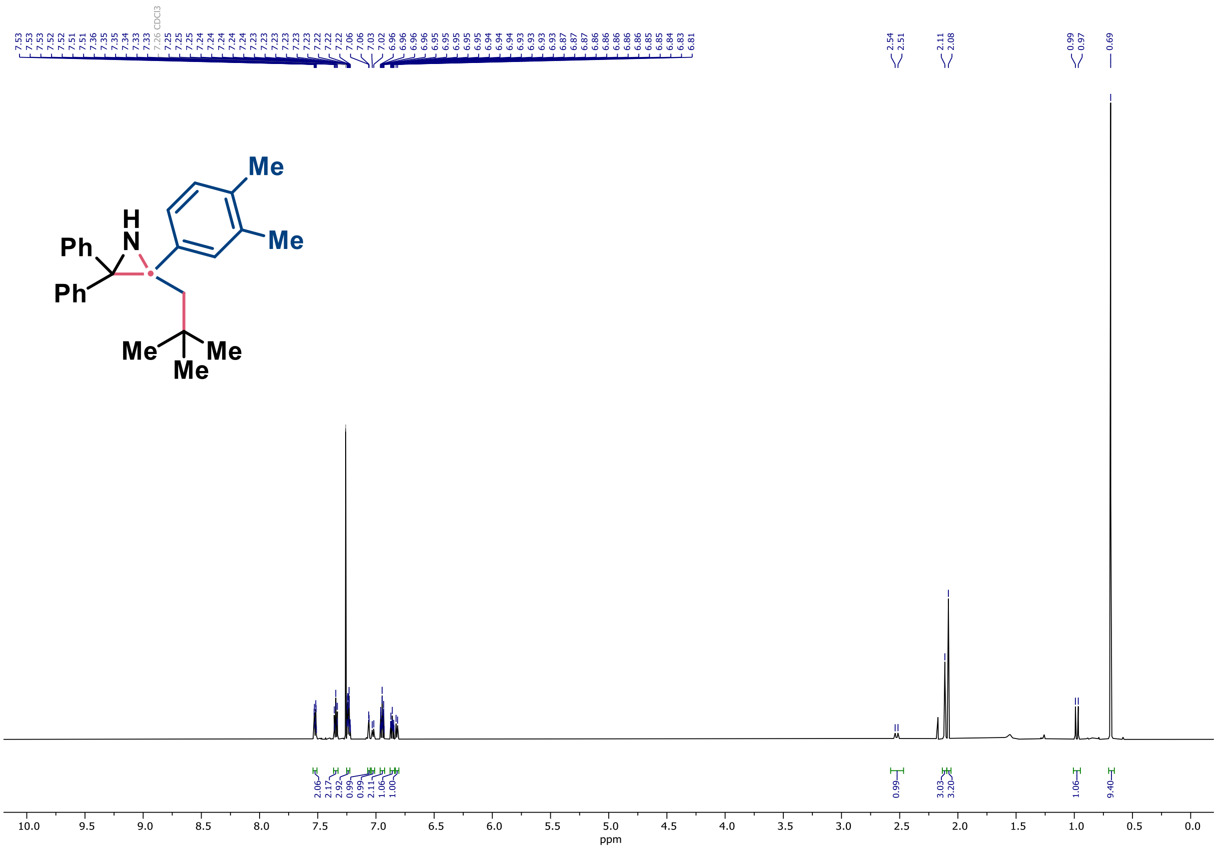

^{13}C{^{1}H} NMR ($CDCl_3$, 101 MHz) for **3ah**

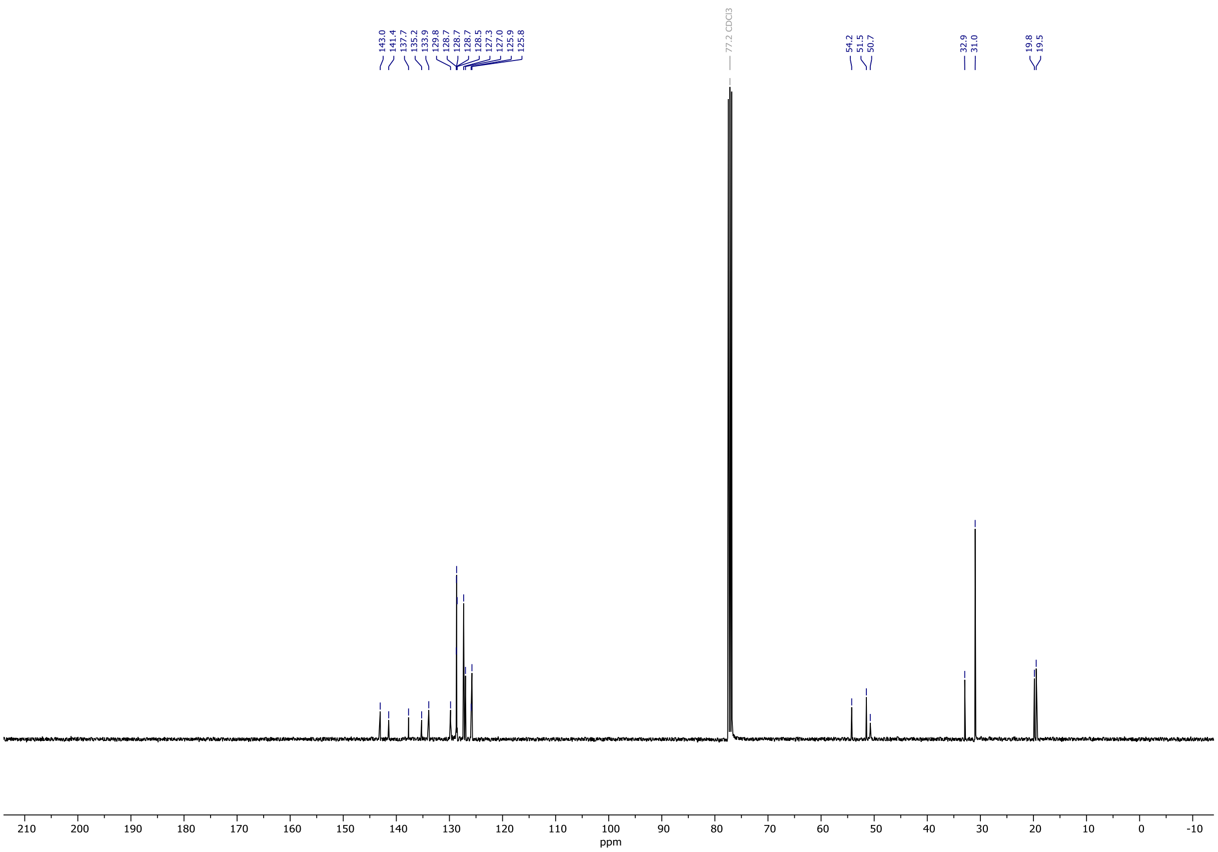

^{1}H NMR (CDCl$_3$, 400 MHz) for **3ai**

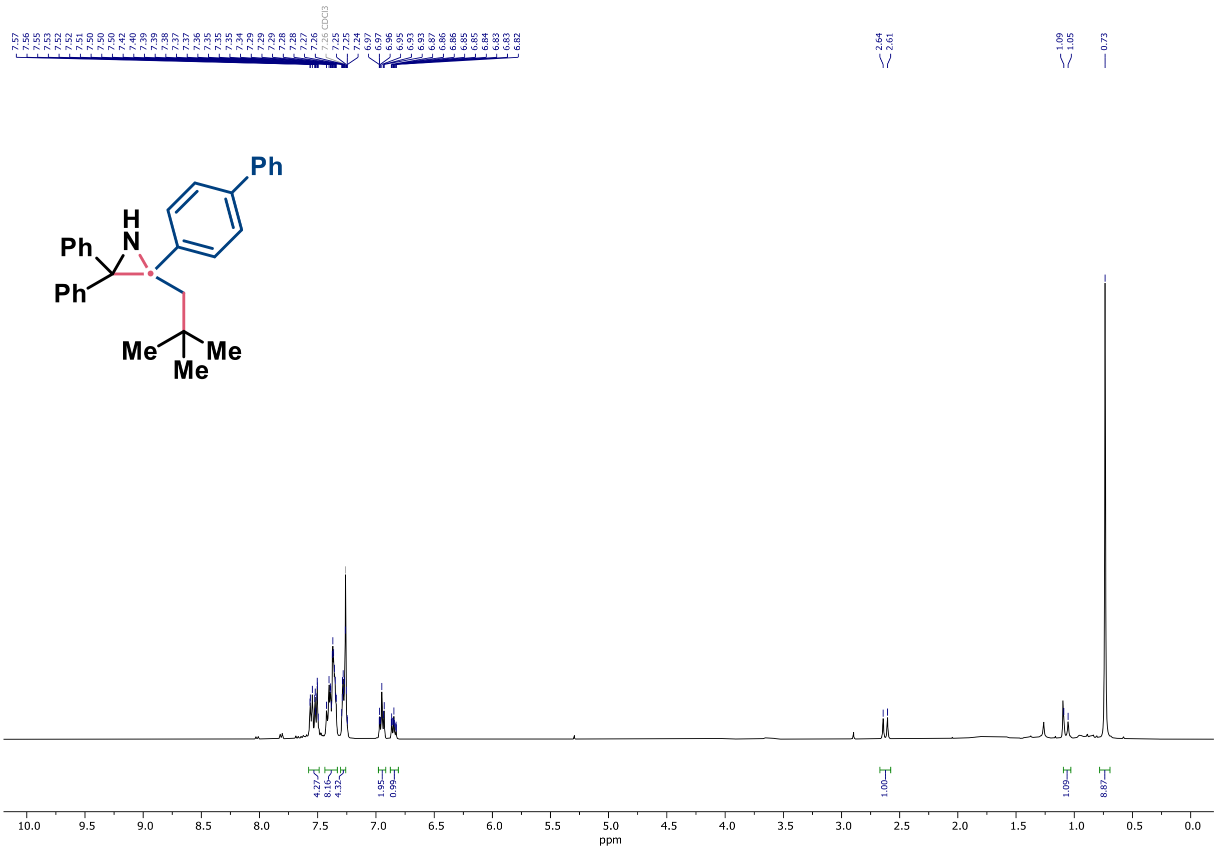

^{13}C{^{1}H} NMR (CDCl$_3$, 101 MHz) for **3ai**

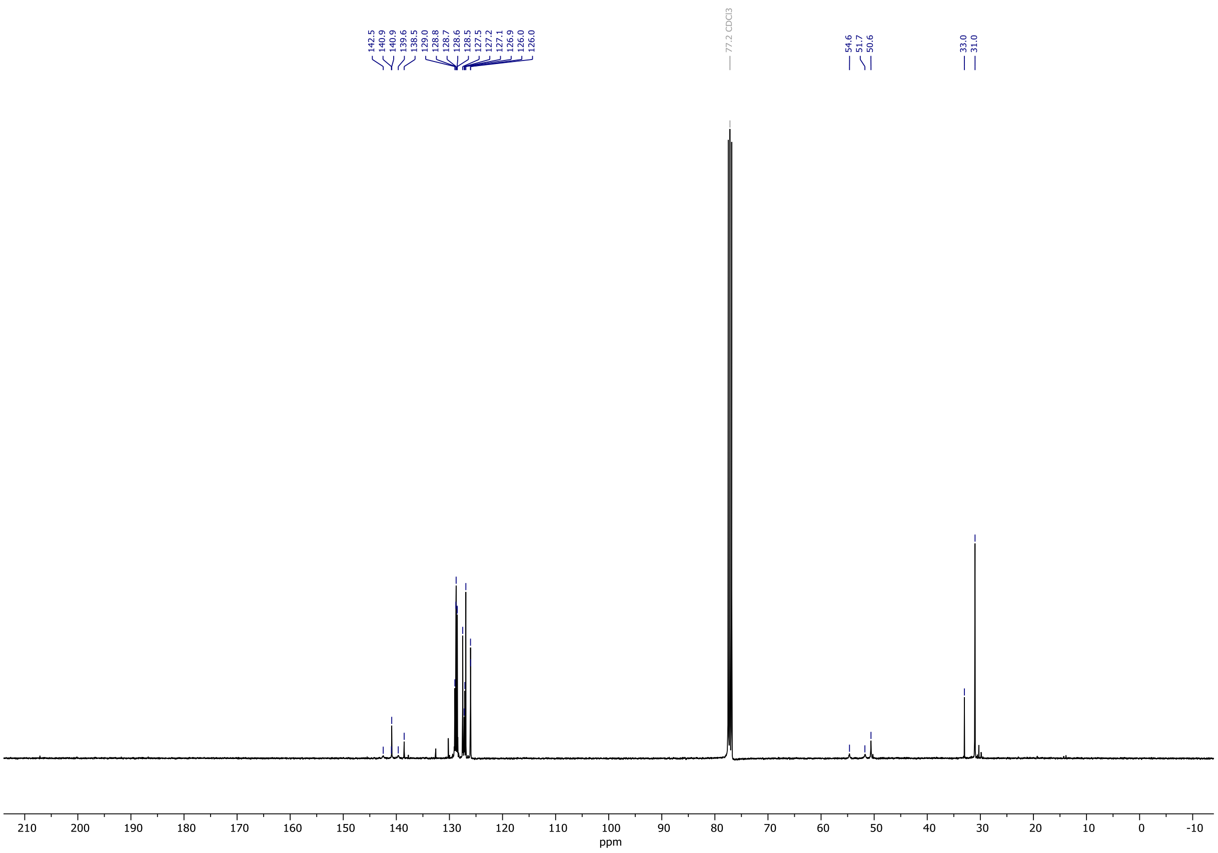

^{1}H NMR ($CDCl_3$, 599 MHz) for **3aj**

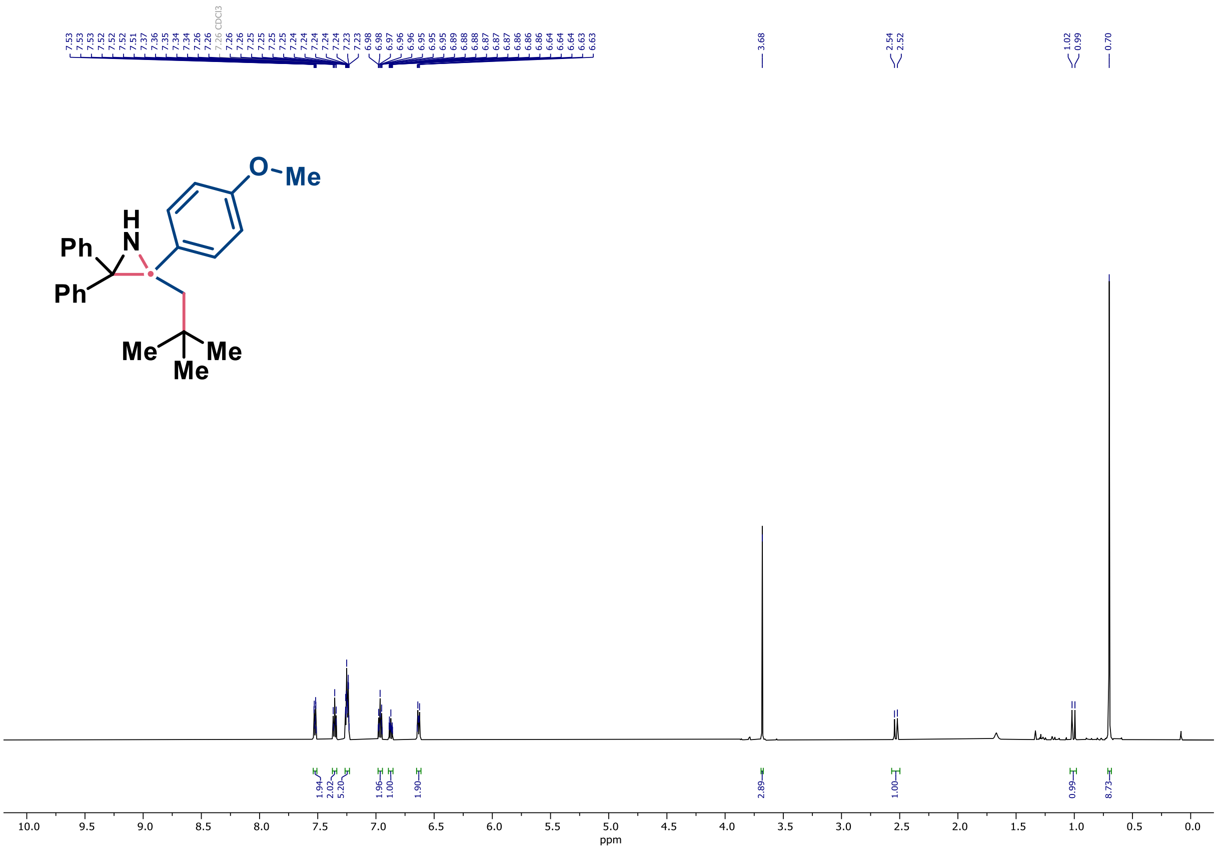

^{13}C{^{1}H} NMR ($CDCl_3$, 151 MHz) for **3aj**

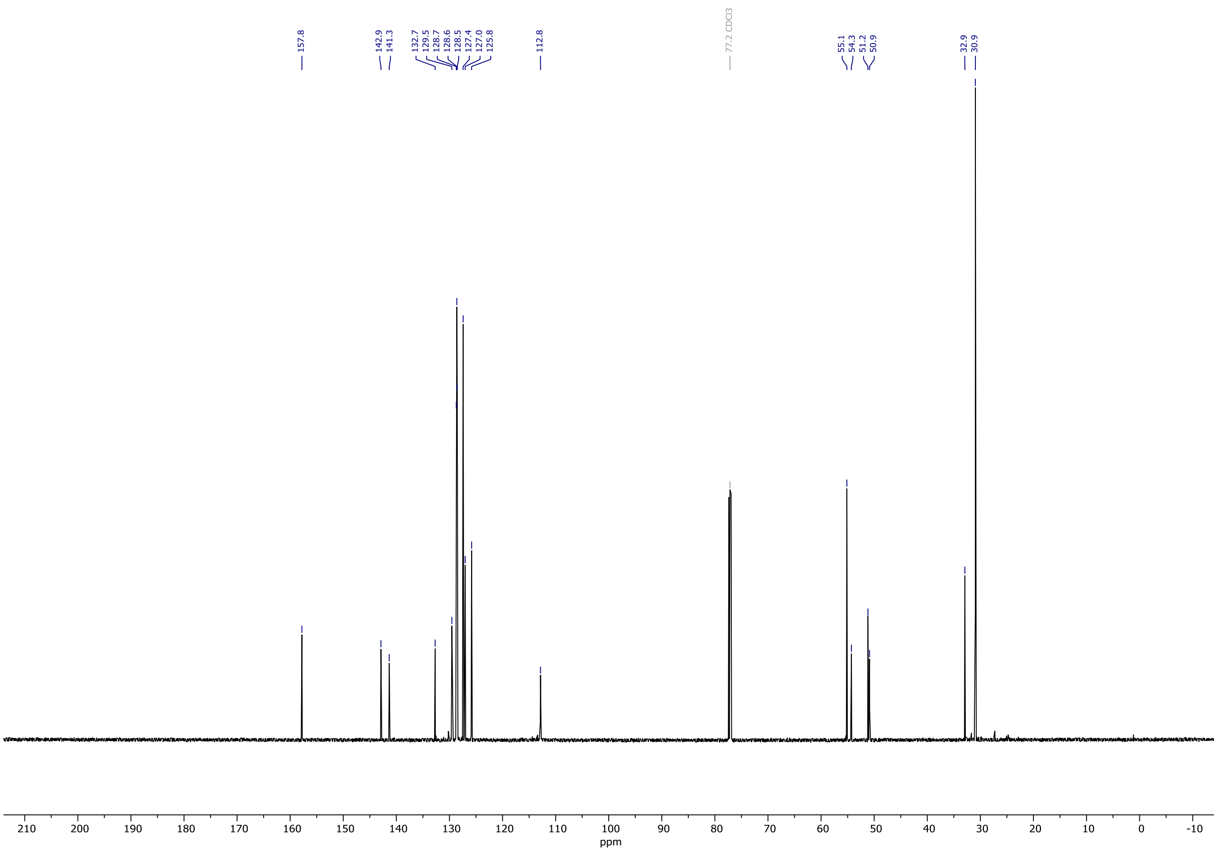

^{1}H NMR ($CDCl_3$, 500 MHz) for **3ak**

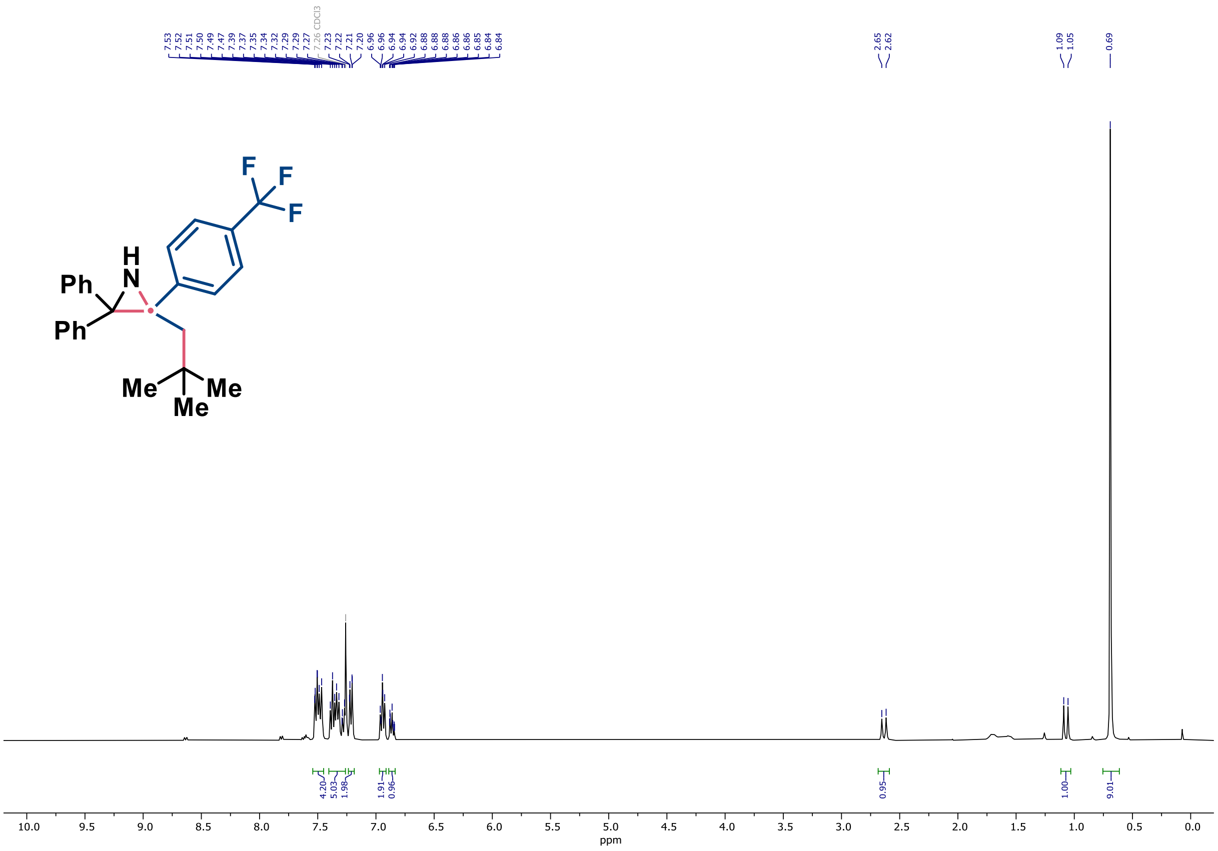

^{13}C{^{1}H} NMR ($CDCl_3$, 126 MHz) for **3ak**

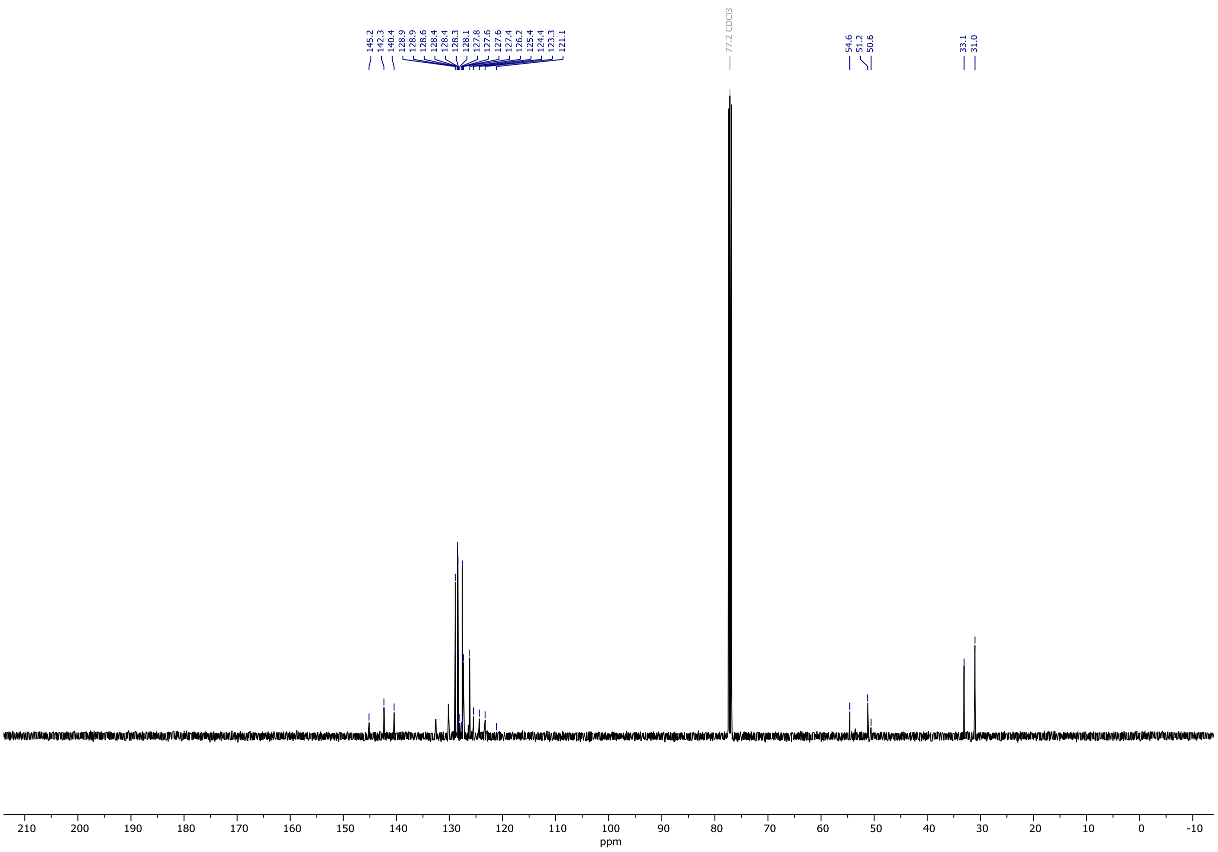

^{19}F{^{1}H} NMR ($CDCl_3$, 470 MHz) for **3ak**

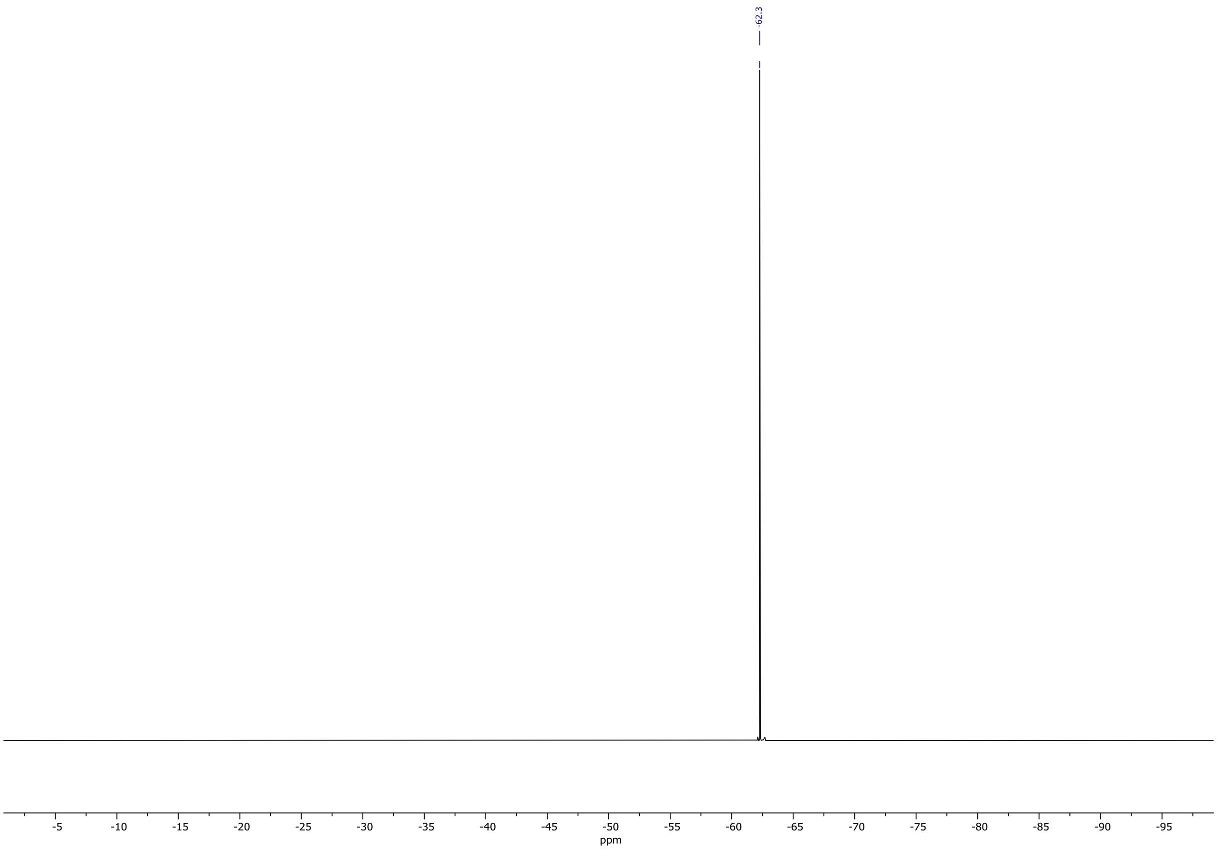

^{1}H NMR (CDCl$_3$, 500 MHz) for **3al**

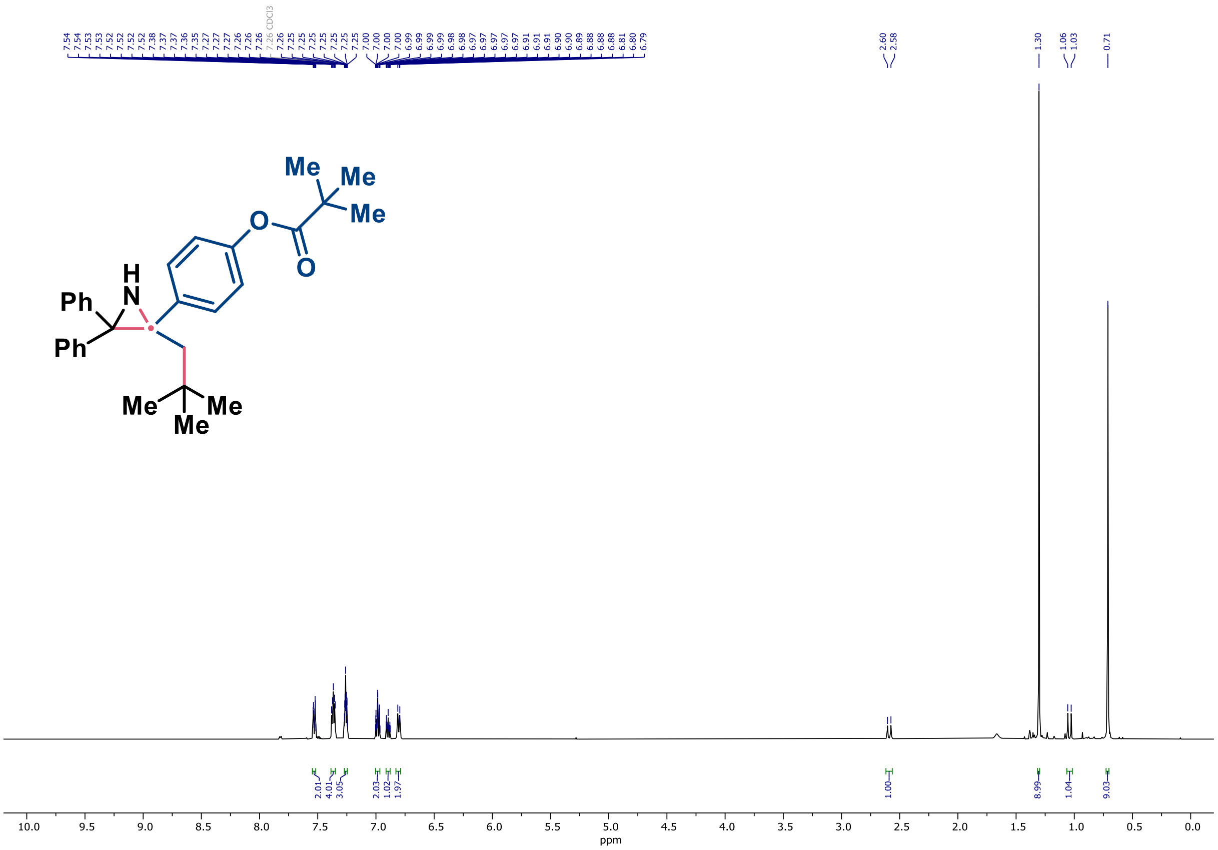

^{13}C{^{1}H} NMR (CDCl$_3$, 126 MHz) for **3al**

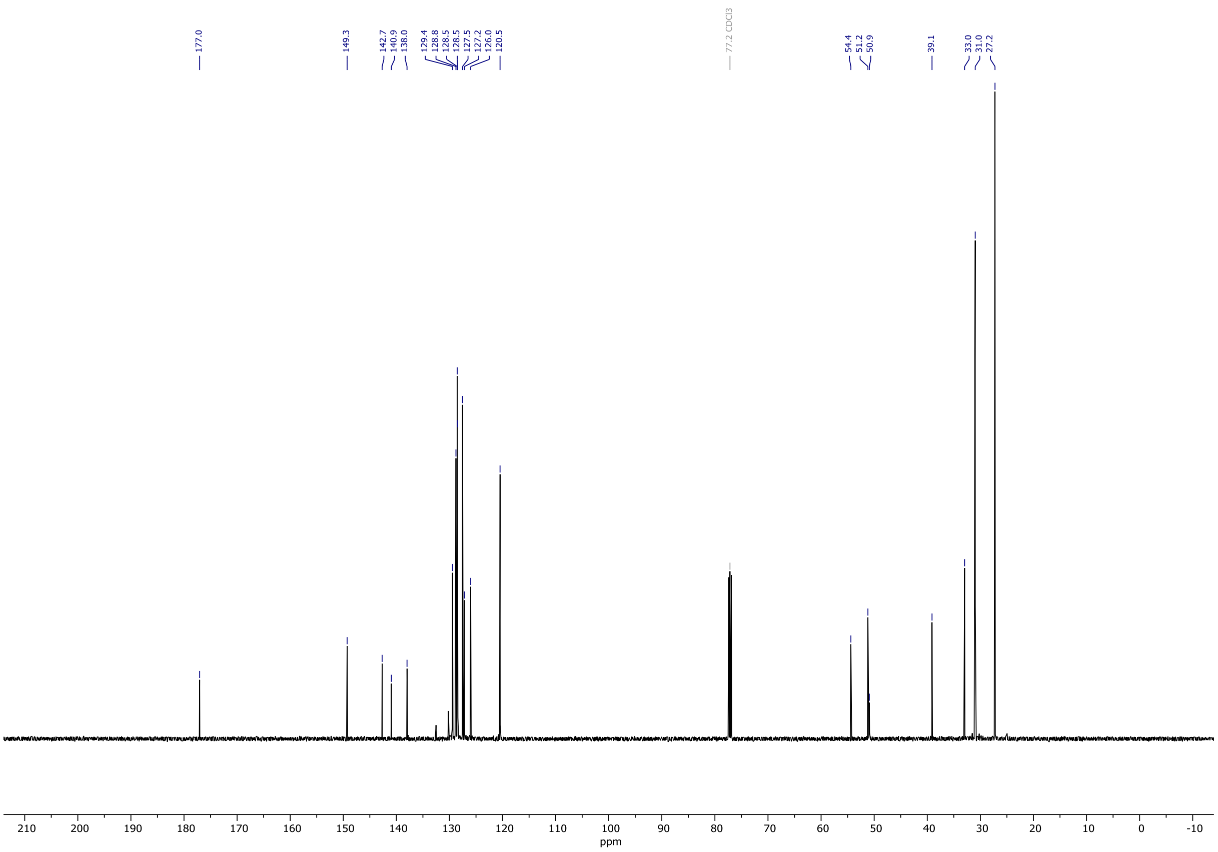

^{1}H NMR (CDCl$_3$, 599 MHz) for **3am**

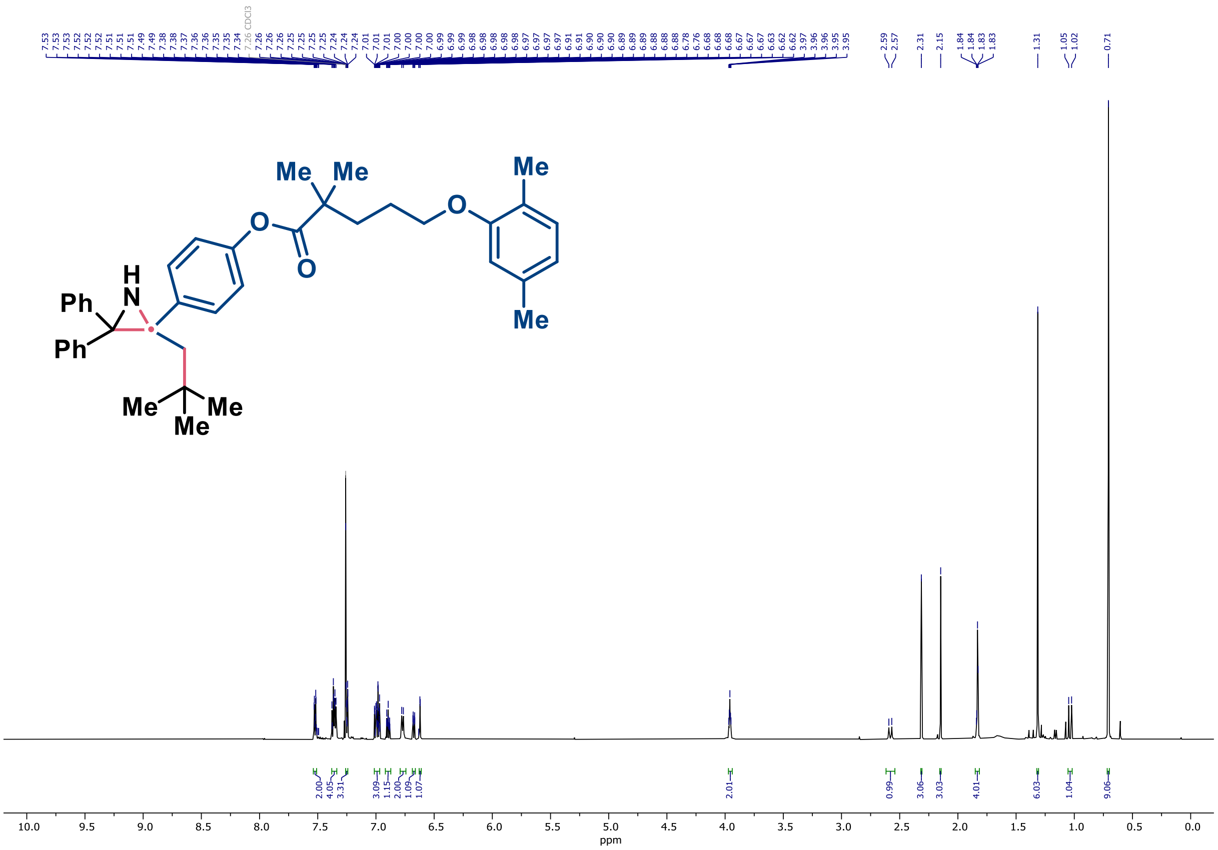

^{13}C{^{1}H} NMR (CDCl$_3$, 151 MHz) for **3am**

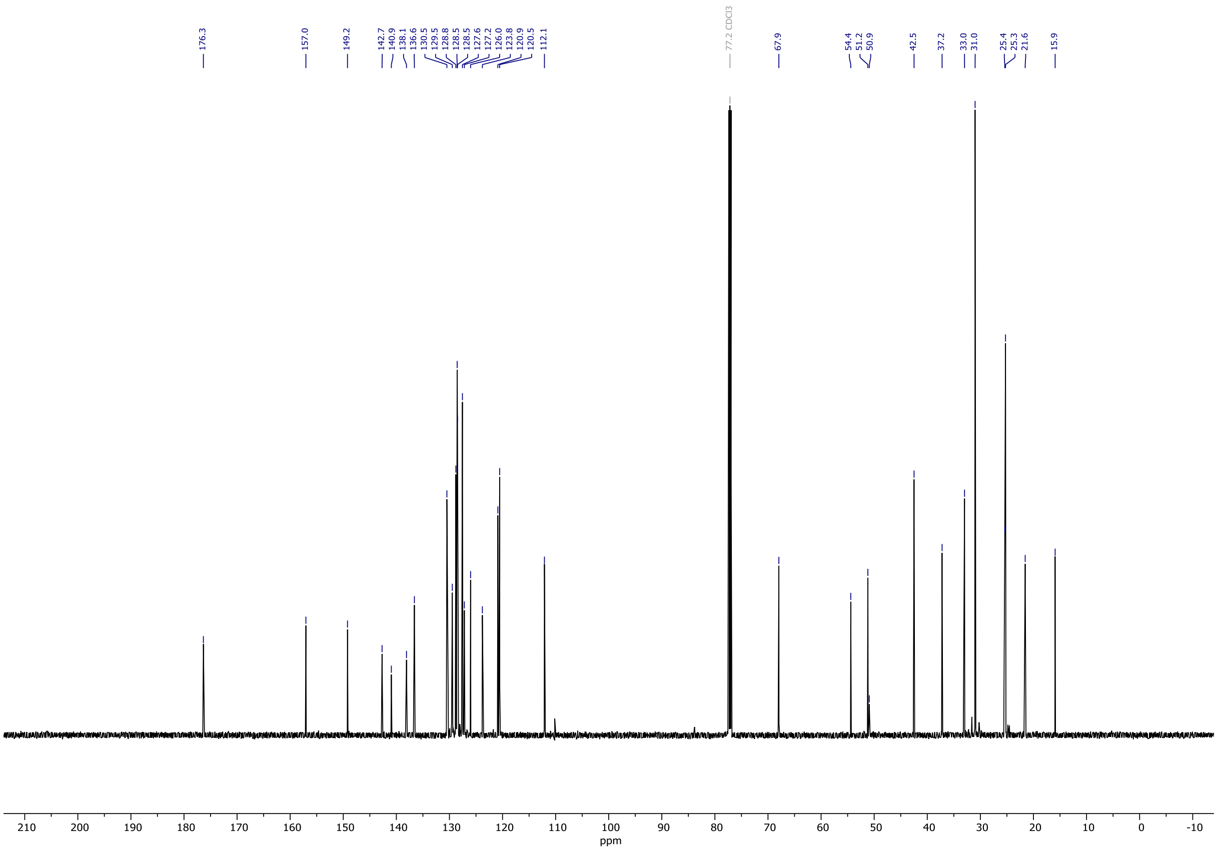

¹H NMR (CDCl$_3$, 500 MHz) for **3an**

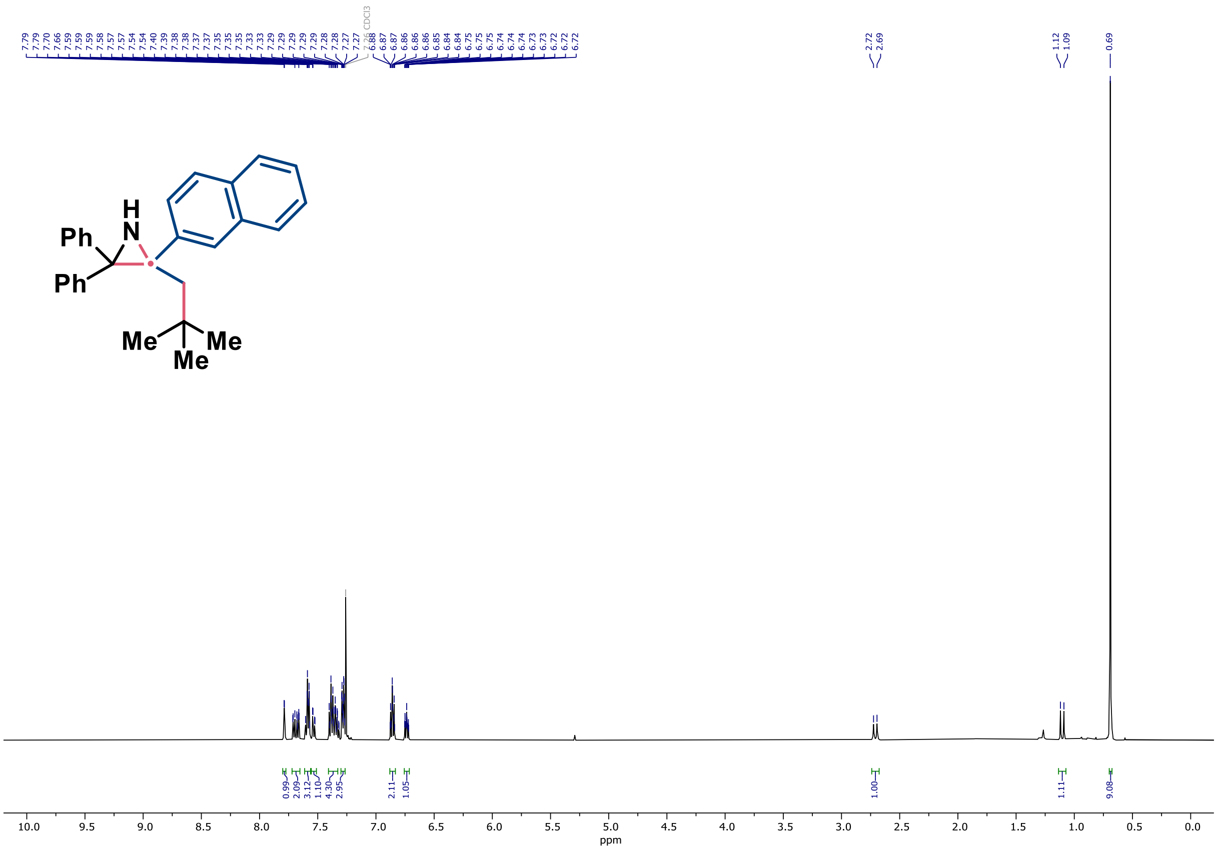

¹³C{¹H} NMR (CDCl$_3$, 101 MHz) for **3an**

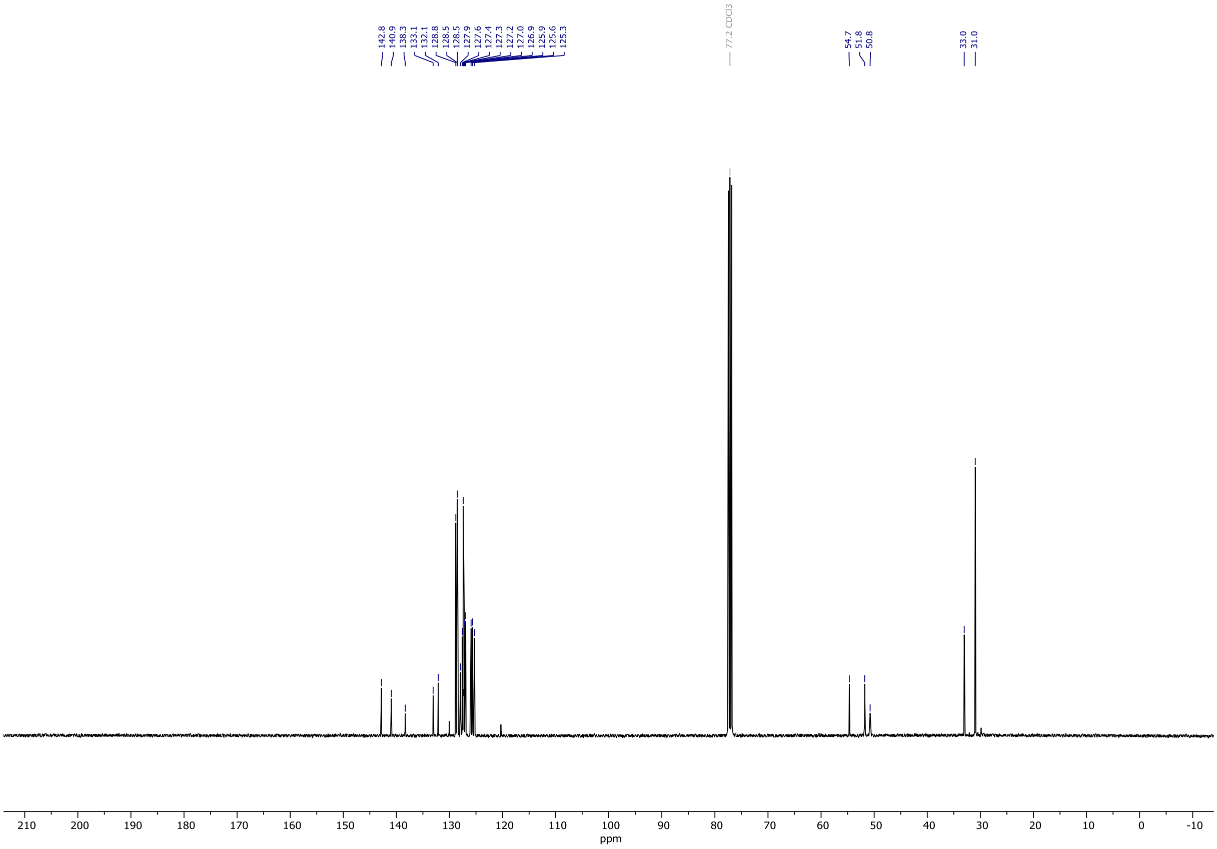

¹H NMR ($CDCl_3$, 500 MHz) for **3ao**

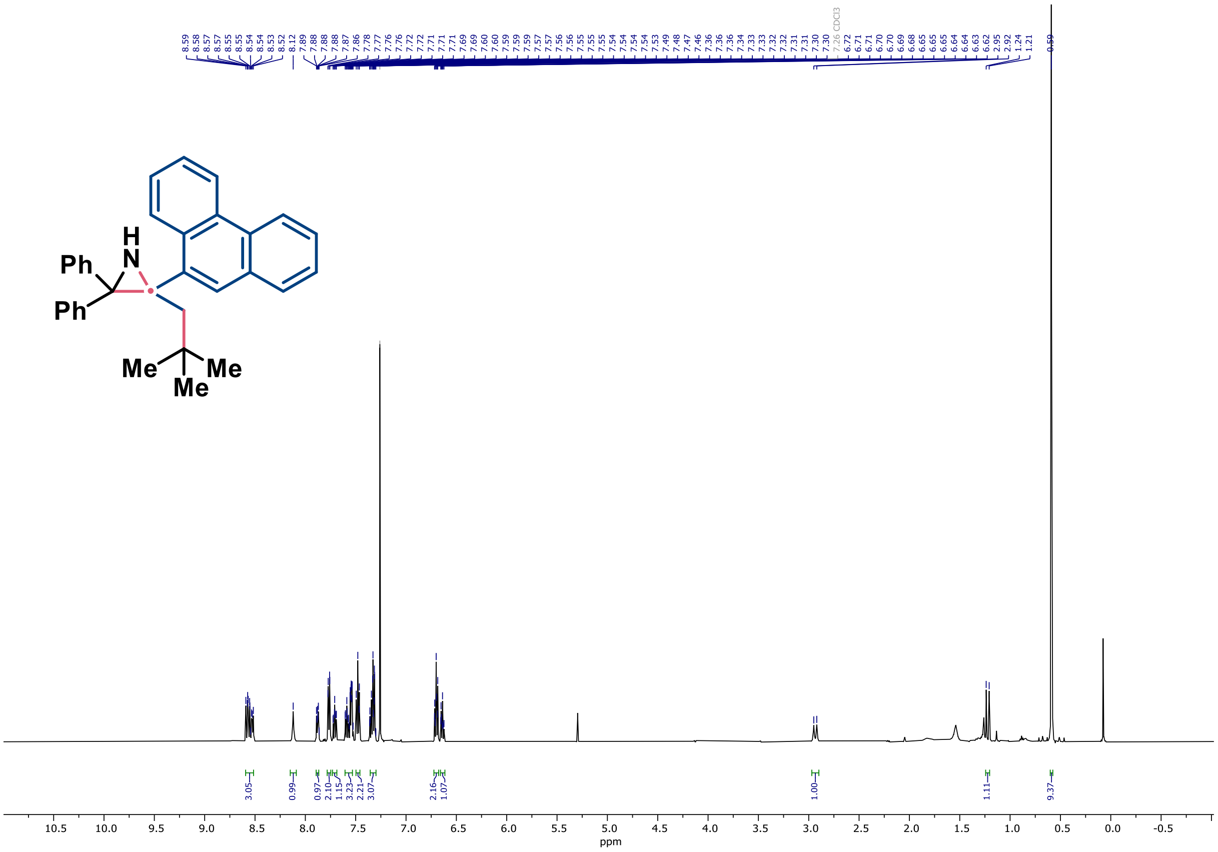

¹³C{¹H} NMR ($CDCl_3$, 101 MHz) for **3ao**

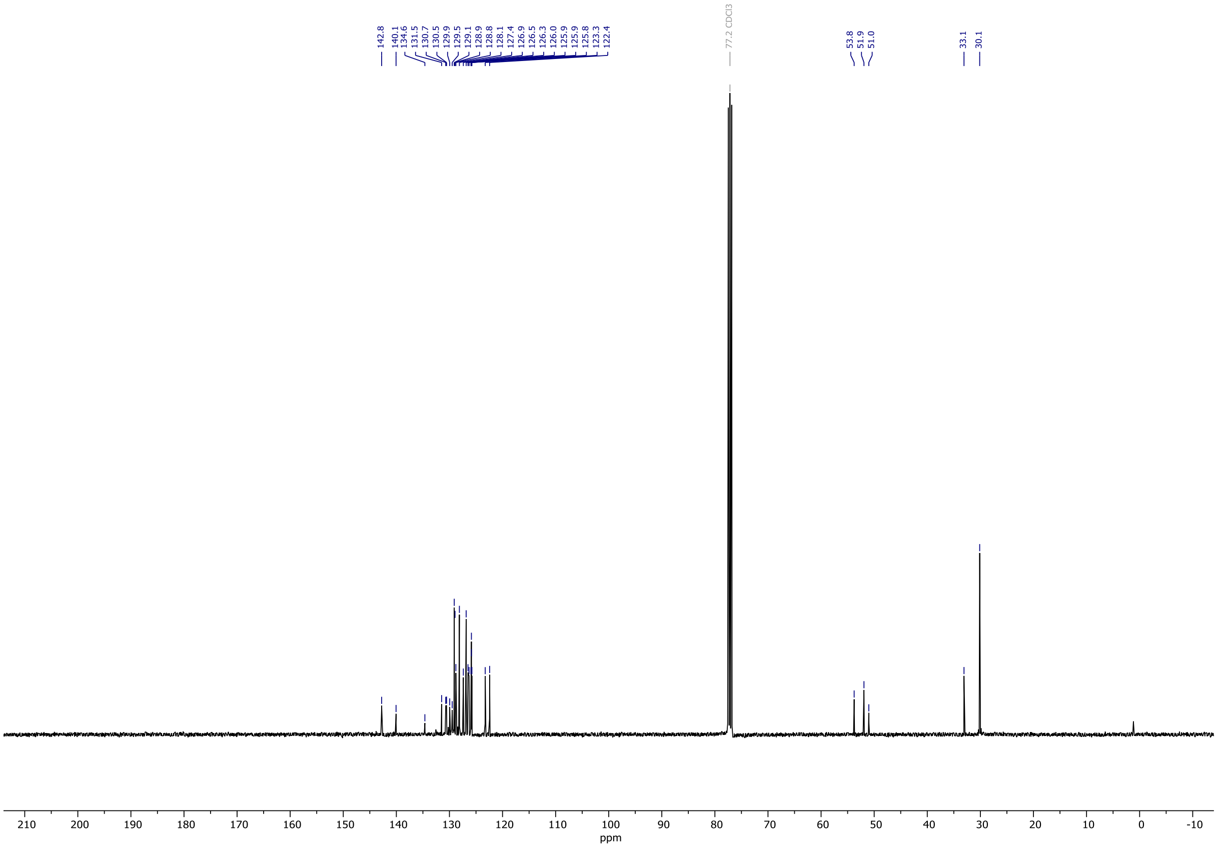

8.3 Other

^{1}**H NMR** (CDCl$_3$, 400 MHz) for **6**

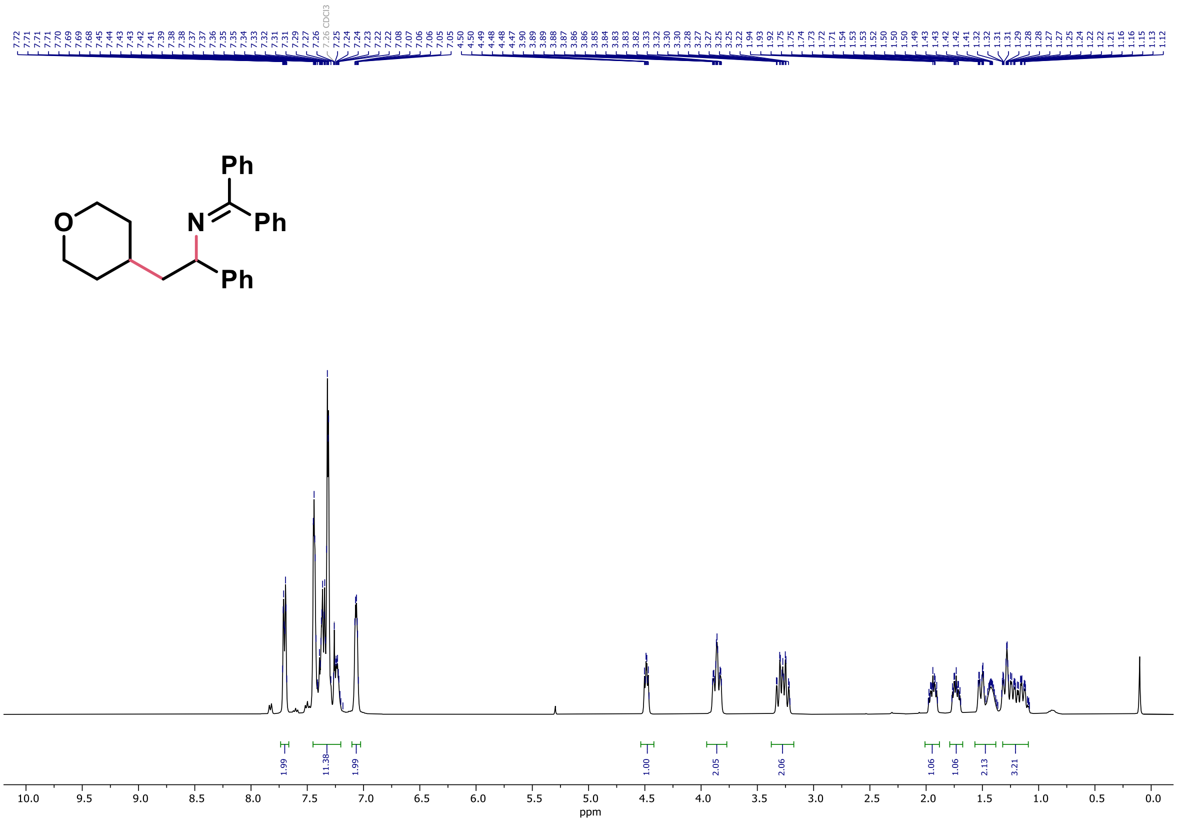

^{13}**C{^{1}H} NMR** (CDCl$_3$, 101 MHz) for **6**

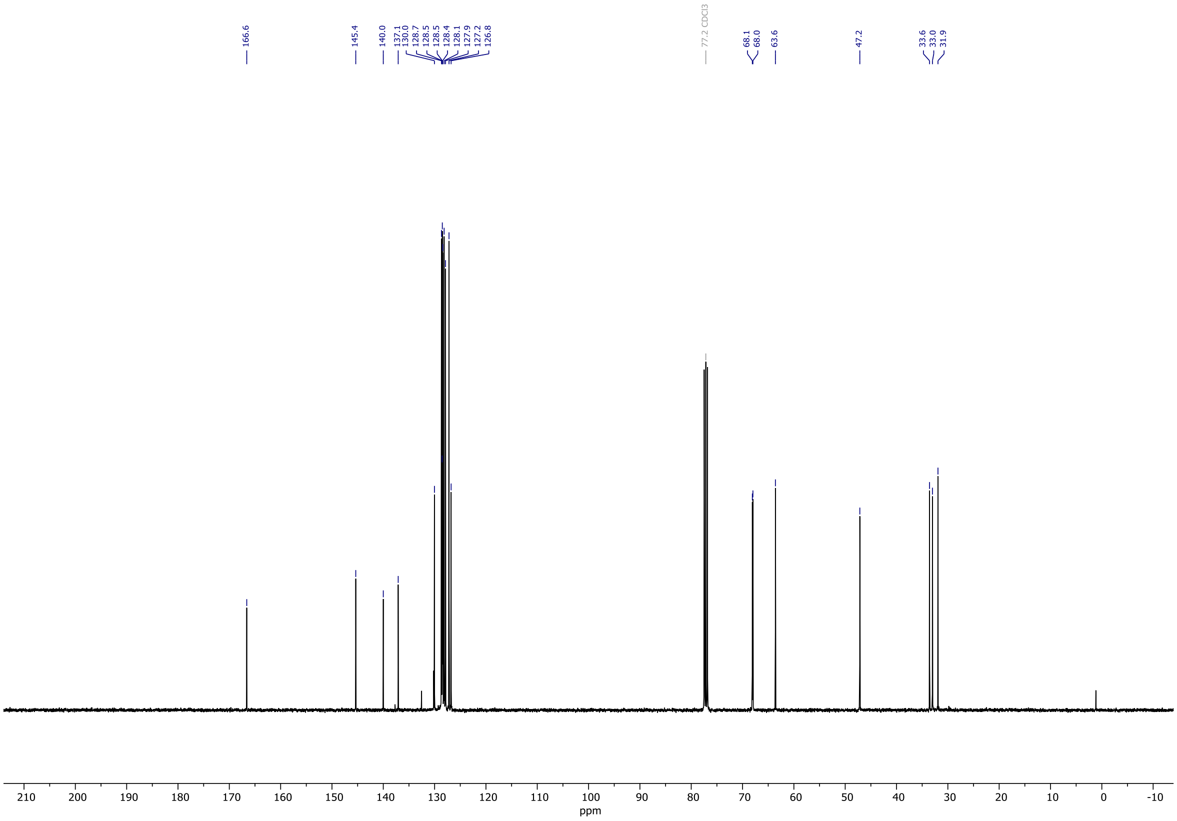

¹H NMR (CDCl$_3$, 500 MHz) for **10**

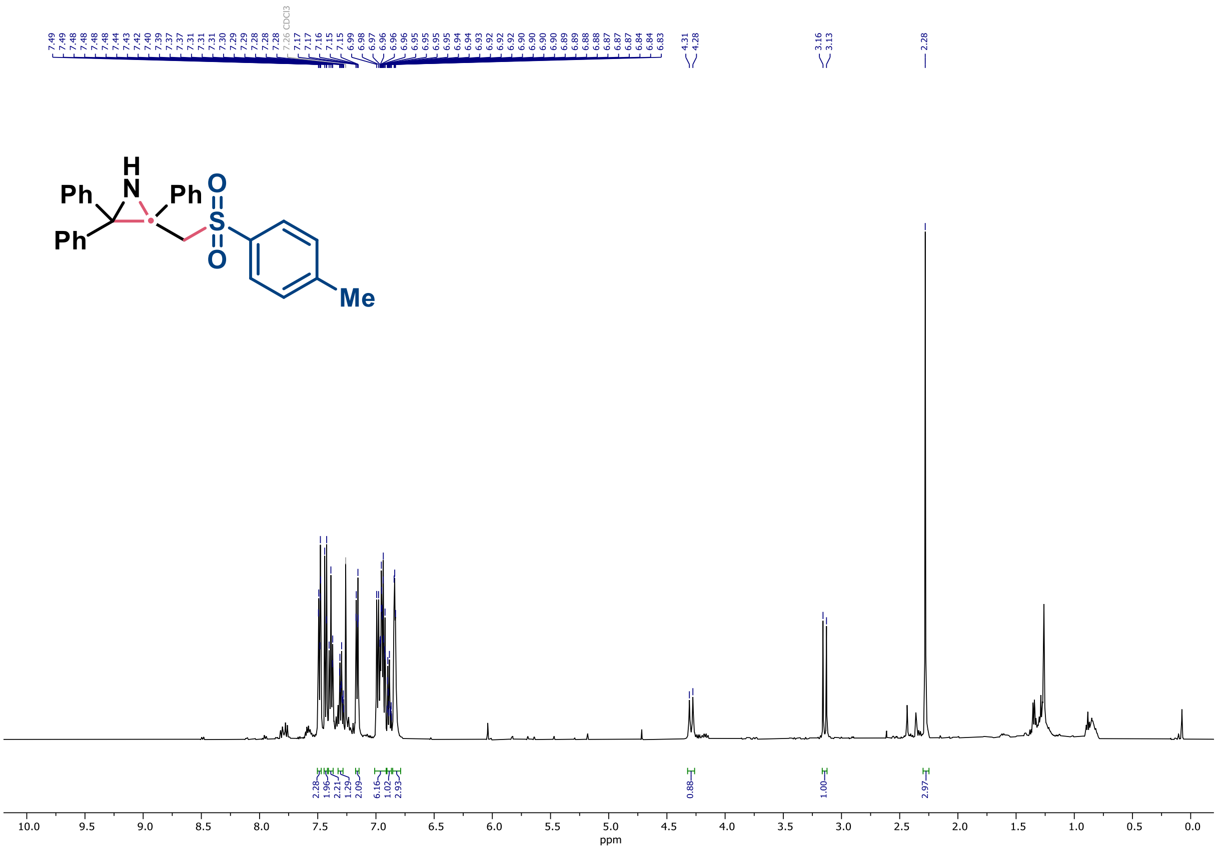

¹³C{¹H} NMR (CDCl$_3$, 126 MHz) for **10**

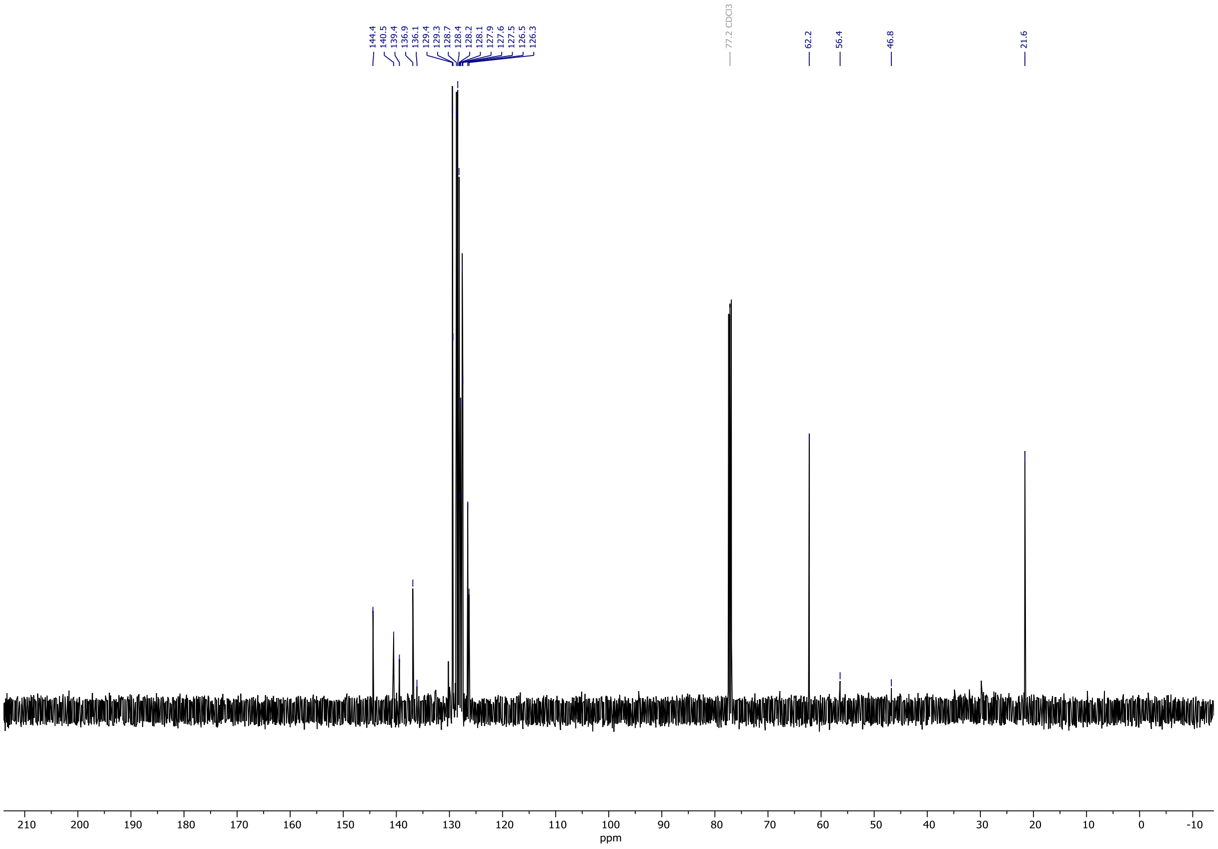

^{1}H NMR ($CDCl_3$, 400 MHz) for **11**

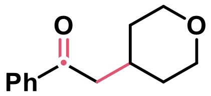

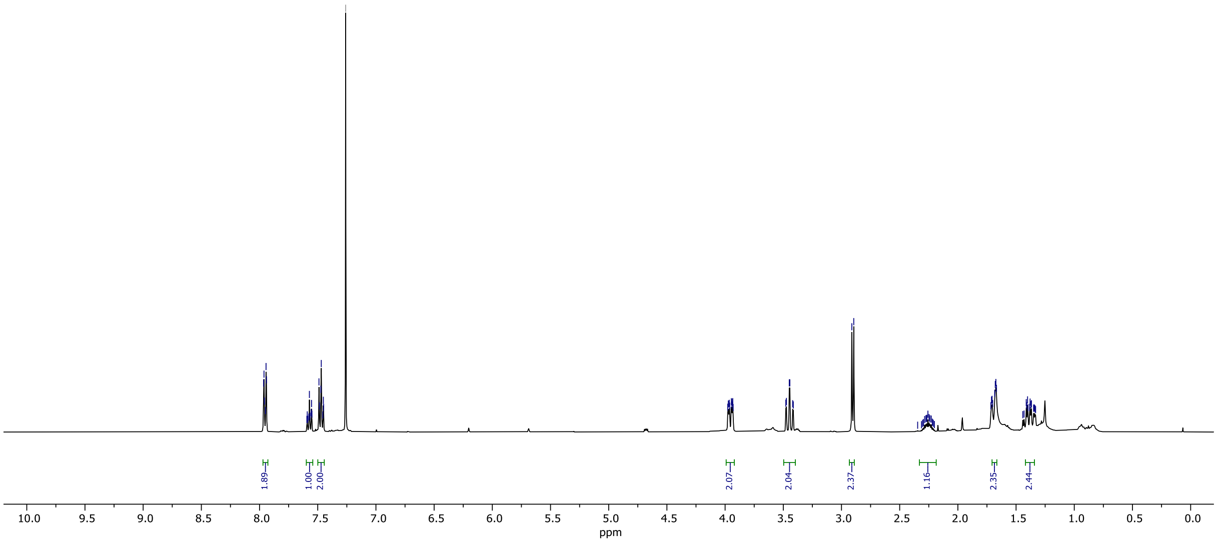

^{13}C{^{1}H} NMR ($CDCl_3$, 101 MHz) for **11**

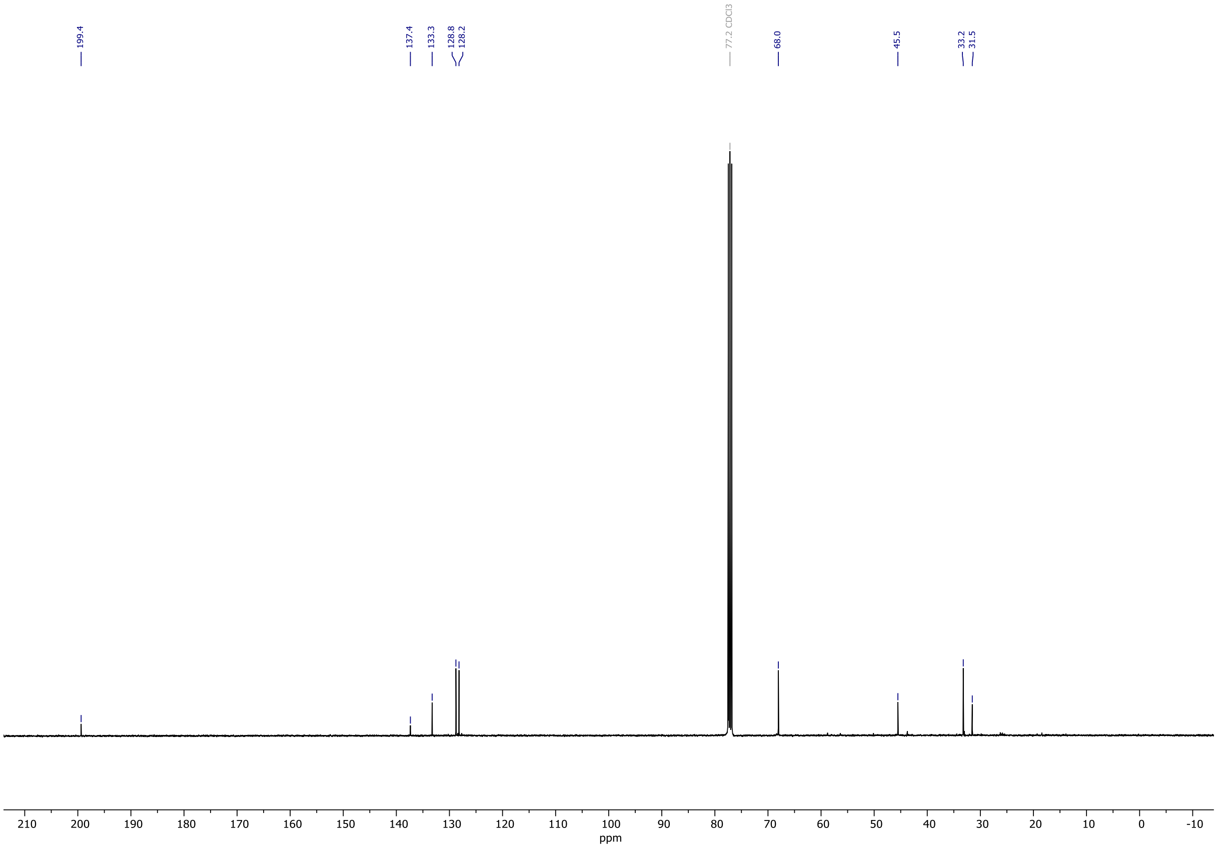

¹H NMR (CDCl$_3$, 599 MHz) for **12**

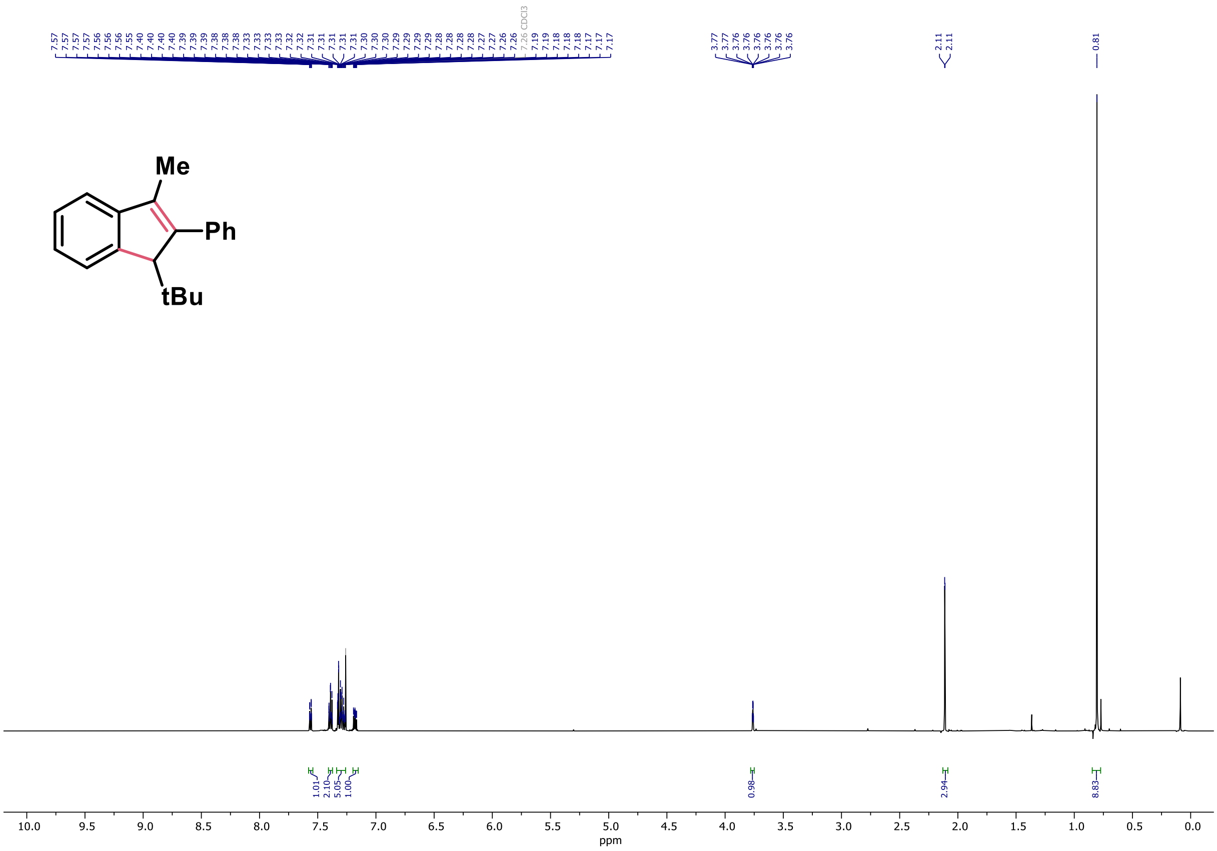

¹³C{¹H} NMR (CDCl$_3$, 151 MHz) for **12**

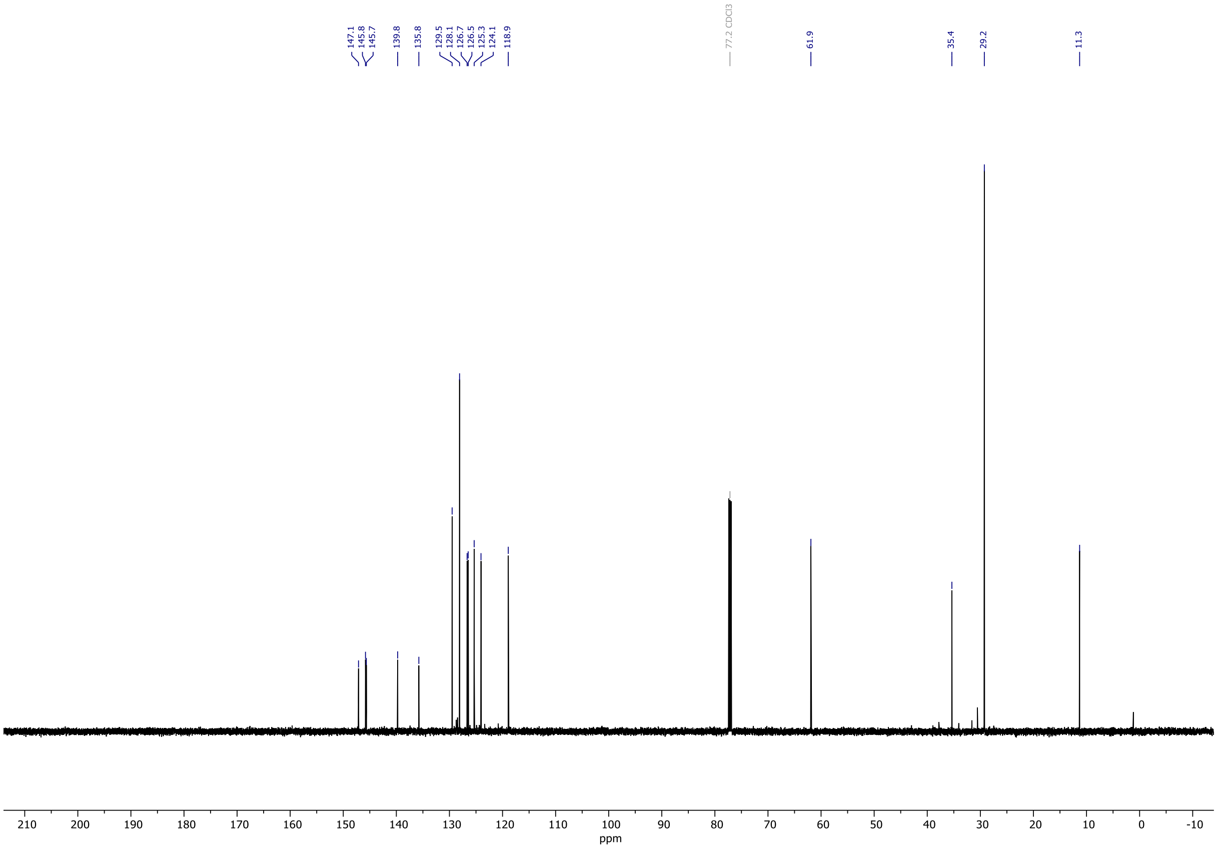

^{1}H NMR (CDCl$_3$, 400 MHz) for **13**

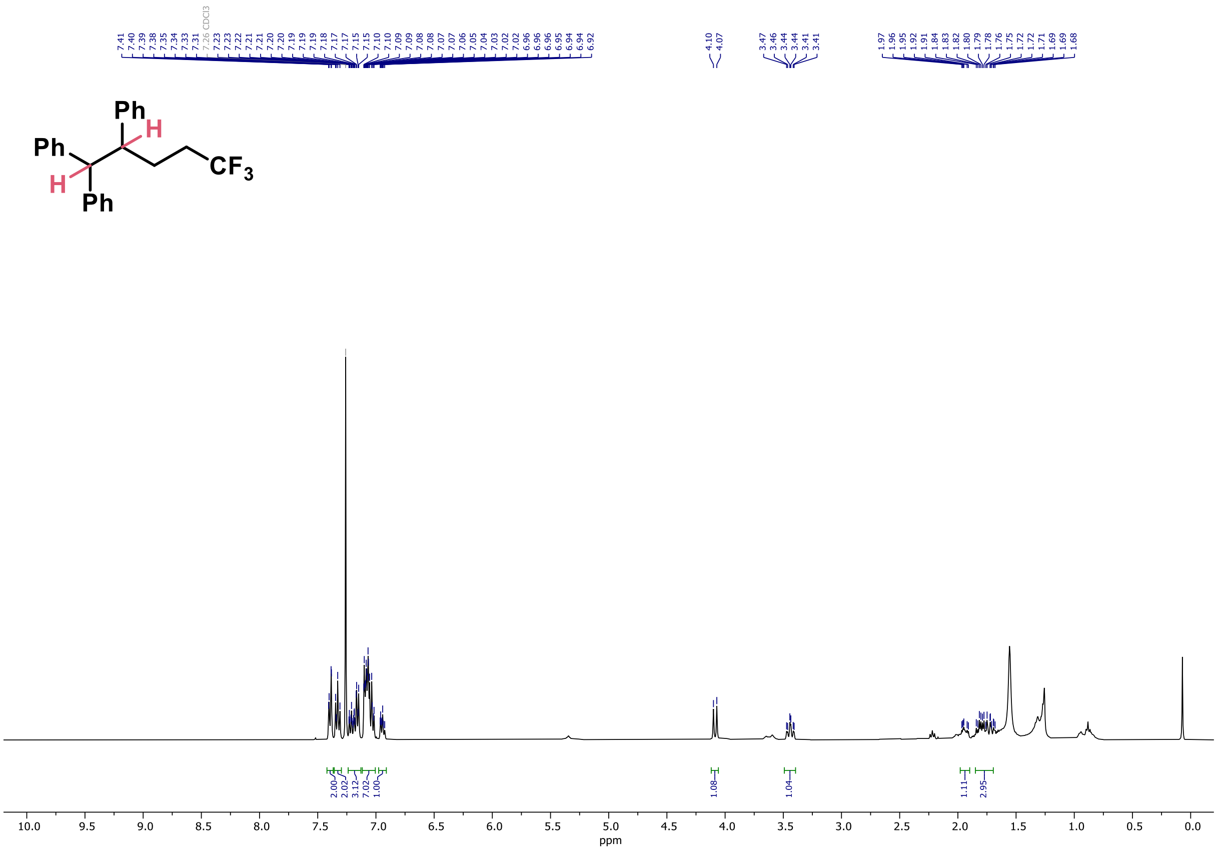

^{13}C{^{1}H} NMR (CDCl$_3$, 126 MHz) for **13**

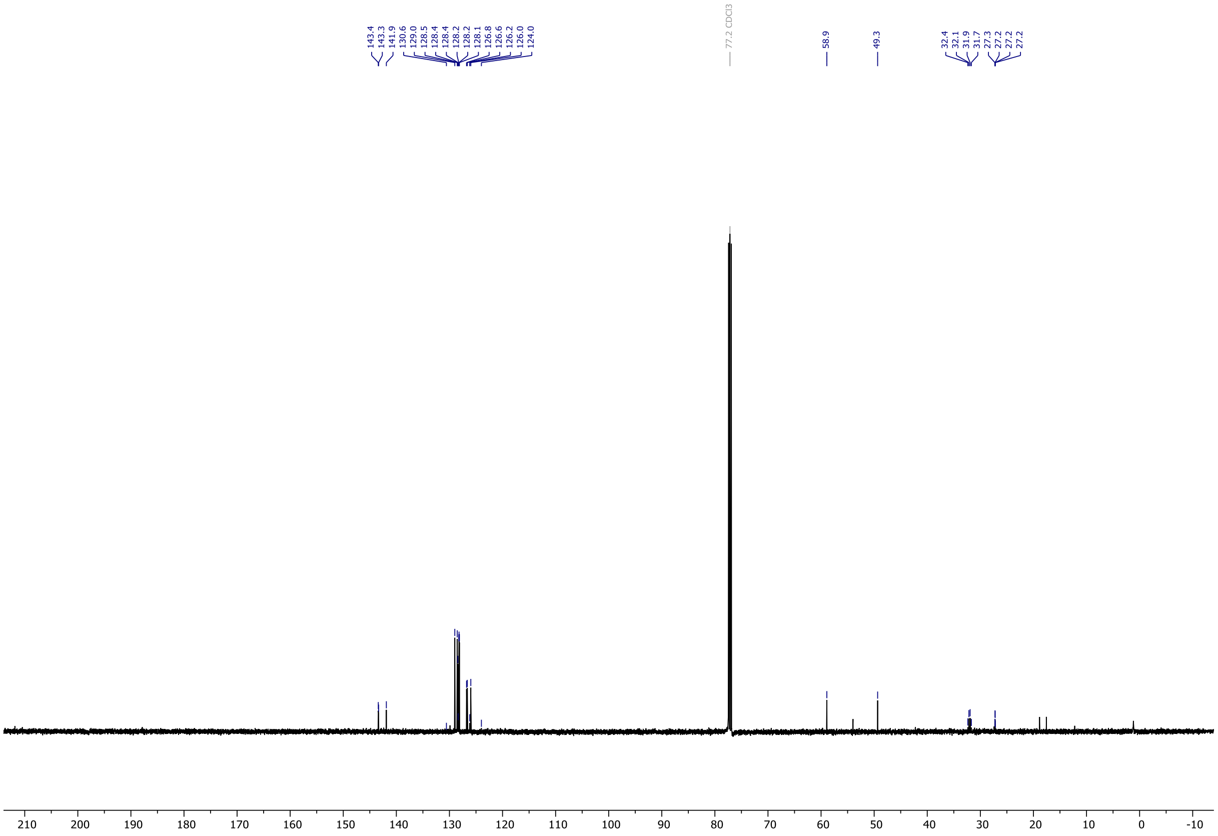

$^{19}F\{^1H\}$ NMR ($CDCl_3$, 377 MHz) for **13**

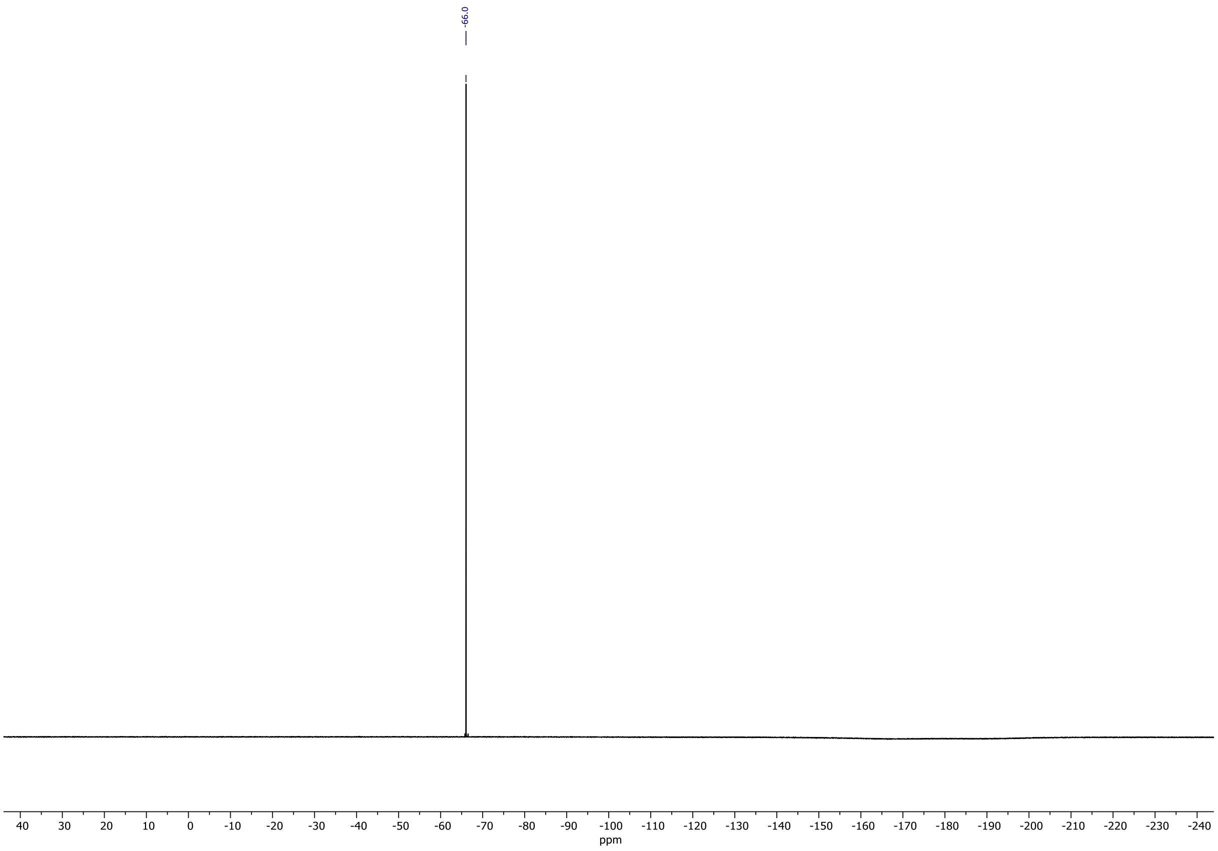

9. References

[1] Y. M. Ivon, Z. V. Voitenko, O. O. Grygorenko, *Synthesis* **2018**, *50*, 1857.

[2] A. Spaggiari, D. Vaccari, P. Davoli, G. Torre, F. Prati, *J. Org. Chem.* **2007**, *72*, 2216.

[3] C. R. Davis, Y. Fu, P. Liu, J. M. Ready, *J. Am. Chem. Soc.* **2022**, *144*, 16118.

[4] X. Tian, L. Wu, *Chem. Sci.* **2025**, *16*, 6515.

[5] X.-Y. Dong, T.-Y. Zhan, S.-P. Jiang, X.-D. Liu, L. Ye, Z.-L. Li, Q.-S. Gu, X.-Y. Liu, *Angew. Chem. Int. Ed.* **2021**, *60*, 2160.

[6] T. von Geldern, B. Backes, B. He, J. Harris, WO2021108549 (A1), **2021**.

[7] X. Su, H. Huang, Y. Yuan, Y. Li, *Angew. Chem. Int. Ed.* **2017**, *56*, 1338.

[8] P. Zhang, J. Meijide Suárez, T. Driant, E. Derat, Y. Zhang, M. Ménand, S. Roland, M. Sollogoub, *Angew. Chem. Int. Ed.* **2017**, *56*, 10821.

[9] D. Shen, D. Cao, R. Zhang, P. Bai, Z. Liu, *Org. Biomol. Chem.* **2024**, *22*, 4062.

[10] G. Tan, F. Paulus, A. Petti, M.-A. Wiethoff, A. Lauer, C. Daniliuc, F. Glorius, *Chem. Sci.* **2023**, *14*, 2447.

[11] F. Paulus, C. Heusel, M. Jaspers, L. M. Amrehn, F. Schreiner, D. Rana, C. G. Daniliuc, M. R. Hansen, F. Glorius, *Angew. Chem. Int. Ed.* **2025**, *64*, e202504793.

[12] R. Laskar, R. E. Thielemann, J. Knüppe, N. Hölter, C. G. Daniliuc, F. Glorius, *ACS Catal.* **2025**, *15*, 6731.

[13] F. Paulus, C. Stein, C. Heusel, T. J. Stoffels, C. G. Daniliuc, F. Glorius, *J. Am. Chem. Soc.* **2023**, *145*, 23814.

[14] L. Pitzer, F. Schäfers, F. Glorius, *Angew. Chem. Int. Ed.* **2019**, *58*, 8572.

[15] M. A. Cismesia, T. P. Yoon, *Chem. Sci.* **2015**, *6*, 5426.

[16] C. G. Hatchard, Parker C. A., *Proc. R. Soc. London. Ser. A. Math. Phys. Sci.* **1956**, *235*, 518.

[17] J. Chen, A. S. Dvornikov, P. M. Rentzepis, *J. Phys. Chem. A* **2009**, *113*, 8818.

[18] E. E. Wegner, A. W. Adamson, *J. Am. Chem. Soc.* **1966**, *88*, 394.

[19] H. Zhang, X. Zhao, R. Yan, W. Lin, *Org. Lett.* **2024**, *26*, 4251.

[20] Y. Sun, Y. Wang, Z. Zuo, *Org. Chem. Front.* **2024**, *11*, 6510.

[21] S. Liu, W. Zhao, J. Li, N. Wu, C. Liu, X. Wang, S. Li, Y. Zhu, Y. Liang, X. Cheng, *CCS Chem.* **2022**, *4*, 693.
